# Dendrochemical Challenge in Climate Science: Whether Chemical Elements in Wood Reflect the Fluctuations in Weather Parameters

**DOI:** 10.3390/plants11233240

**Published:** 2022-11-25

**Authors:** Vladimir Gavrikov, Alexey Fertikov, Ruslan Sharafutdinov, Anton Pyzhev, Eugene Vaganov

**Affiliations:** 1 School Ecology and Geography, Siberian Federal University, Krasnoyarsk 660041, Russia; 2 Institute for Forest SB RAS, Krasnoyarsk 660036, Russia

**Keywords:** elemental content, tree rings, conifers, X-ray fluorescence analysis, weather parameters

## Abstract

The aim of this study was to find consistent correlations between weather parameters and elemental content of tree rings in four widely distributed Siberian conifers: Siberian spruce (*Picea obovata* Ledeb.), Scots pine (*Pinus sylvestris* L.), Siberian larch (*Larix sibirica* Ledeb.), and Siberian pine (*Pinus sibirica* Du Tour). Slices from the wood cores were subjected to chemical treatment by extraction in alcohol and HCl. The slices were scanned using an Itrax Multiscanner (COX Analytical Systems) to obtain the count rates of Al, P, S, K, Ca, Ti, Mn, Fe, Cu, Zn, and Sr. Every slice was scanned three times, in the original form, after alcohol extraction, and after HCl treatment. Altogether, 21 weather parameters were used to search for consistent correlations between the elemental contents. Weather parameters as well as elemental contents were averaged for successive triplets of calendar years. The statistical treatment of the data included the calculations of non-parametrical Spearman rho and Kendall tau coefficients. We defined consistent correlation as a correlation that is stronger than +/–0.3, observed in all the trees studied, and is significant at least in one tree. The main result of the study is that no consistent correlation was found that we could observe in all the species involved in the study. Nevertheless, there are several consistent correlations within the species. This means that the right choice of species for a dendrochemical study is of paramount importance. In some species, e.g., the larch and spruce, we found no correlations unless the chemical treatment was applied. Thus, a chemical treatment may reveal the reactions of tree rings’ elemental content to some weather parameters.

## 1. Introduction

For the last several decades, dendrochemistry has been developing as a promising area that might provide science with new proxies of important natural phenomena. Binda et al. [1] provided a critical review of the dendrochemical literature, paying special attention to the reliability of used proxies. According to their estimations, 46% of reviewed papers were in environmental contamination (e.g., [2,3,4,5,6]). Another, smaller but remarkable, area was paleoclimate reconstruction with 12% of reviewed papers [7,8].

An inference that one could make from the review by Binda et al. [1] is that there are some gaps in paleoclimate and climate reconstructions. Most research uses only stable isotopes, d^18^O and d^13^C, as dendrochemical proxies for climatic parameters. In a very rare case, some heavier elements, such as Ca, were reported to relate to temperature and rainfall [9]. Thus, the range of explored elements is currently rather narrow. Stable isotope ratios may be a compelling method that provides abundant records on relative humidity and soil water status, summer irradiance and temperature, as well as on temperature signals and leaf transpiration [10]. But the use of the method requires mass-spectroscopy techniques that involve thorough sample pretreatment and hence do not allow for obtaining a massive amount of data. On the contrary, direct methods such as X-ray fluorescence, though not without their own problems, provide rich data on a wide spectrum of elements in tree stems.

Another issue regarding dendrochemical proxies is the species choice. The choice problem is infrequently present in the literature, notwithstanding that researchers recognized its value decades ago. In part, an effective choice can appear with direct methods of elements’ detection, allowing one to quickly receive a lot of data from many trees/species. Cutter and Guyette [11] considered possible criteria for tree species suitable for dendrochemistry research. Among the criteria, species with extended geographical distribution, low heartwood moisture, narrow sapwood, and low xylem permeability are beneficial to dendrochemistry. Moreover, the choice of species partly implies the choice of elements because not all the elements are equally mobile in the xylem of different species. Lageard et al. [4] demonstrated that Scots pine is a suitable species to study the scale and timing of past lead pollution in industrial areas of England. Gavrikov et al. [12,13] studied the distribution trends of elements in stems of four widespread Siberian conifers. A few elements (Ca, P, and Co) had consistent trends in the stems irrespective of species. Distribution parameters of other elements (Mn, Pb, Cl, Cr, Ni, Sr, and W) are stably clustered within species.

Lastly, there is a gap in the methods used to treat the cores extracted from tree stems. Normally, thin slices are sawn out of the cores and those slices go through scanning with an X-ray facility ‘as is’, i.e., no additional treatment is applied to the slices. Meanwhile, xylem is an extremely complicated medium. The forms in which elements exist in xylem are largely unknown. Thus, some chemical methods might be helpful in developing a hidden structure of the elements in the wood. Practically, precious little research has used treatment to obtain additional information from the scanned cores. Hietz et al. [14] studied the distribution of elements in the wood of six monsoon forest species. The X-ray images revealed some entities of high density that were more or less concentrated near tree ring boundaries. The energy-dispersive X-ray microanalysis showed that the supposed crystals were mostly of Ca and sometimes of Si content. Moreover, depending on species, the authors found the enrichments in the entities with Fe, K, P, Cu, and Zn. To get more information on the entities, the authors used an HCl extraction technique. In all the cases studied, an application of HCl removed the high-density entities.

In this study, we aim to explore several chemical elements in tree rings of four Siberian conifers, known to be biologically active, regarding their possible reflection of weather parameters. The main hypothesis is two-fold. First, the weather-specific elemental content of annual tree increments (rings) may exist—because weather exerts an obvious impact on the tree growth, while the tree growth occurs at the expense of soil solutions. Second, a chemical extractive treatment of wood may change the correlations (reveal, increase, alter)—because wood is a very complicated natural compound, and its different layers may show different relationships.

## 2. Results

Tables S1–S24 (see electronic Supplementary Material) present the results of all the primary calculations where significant (*p* < 0.05) Spearman and Kendall correlation coefficients are highlighted by the bold font. A noteworthy detail is that the tables in the Supplementary Material are shared with the purpose of providing the reader with the reference data alone, which may be used to perform an alternative analysis.

As can be seen from the tables, the correlation coefficients are highly variable. Sometimes the coefficients from different trees of the same species may have opposite signs. Nevertheless, a visible proportion of the coefficients are significant and have the same sign across the trees. We found no case in which a pair of ‘weather parameter–element counts’ had significant coefficients in all the three trees of the same species. Therefore, a consistent correlation was defined as follows: it was the case when (i) all the correspondent coefficients within a species had the same sign, (ii) all the three coefficients were stronger than +/–0.3, and (iii) at least one of them was significant. Using this rule, we performed a comparison between Spearman and Kendall coefficient matrices and species. An example of the approach is given below.

It should be noted that the value +/–0.3 is to a large extent an arbitrary choice presenting a trade-off between having an unobservable number of weak correlations and no correlations at all. The value 0.3 denotes a weak but visible correlation and the goal was to find enough correlations that could be discussed.

Table 1 summarizes the within-species comparison of the Spearman coefficients. In the table, the species’ name in a table cell means that a consistent correlation is found for the particular pair of ‘weather parameter–element counts’. For Spruce, two consistent correlations were found, which are both related to July weather (humidity and wind speed).

Siberian pine showed the maximum consistent correlations among the species studied. Moreover, three of five correlations in Siberian pine relate to Ti and all those correlations relate to June weather parameters (humidity index, both detrended and not, and the precipitation amount). The other two correlations both relate to August weather, humidity and temperature. Another feature of Siberian pine is that it is the only species that has consistent correlations with the humidity index.

To give an example of a consistent correlation for a Siberian pine’s core that was extracted in alcohol (see Table 1), the consideration was as follows. The Spearman correlations for alcohol-extracted Mn and August mean temperature were −0.57 (Siberian pine #1), −0.41 (#2), and −0.33 (#3), see Tables S7, S9, and S11. All the coefficients are negative and the first one is significant at *p* < 0.05. Thus, according to the adopted definition, the correlation for Mn (after extraction in alcohol) vs. August mean temperature was considered a consistent one.

**Figure 1:**
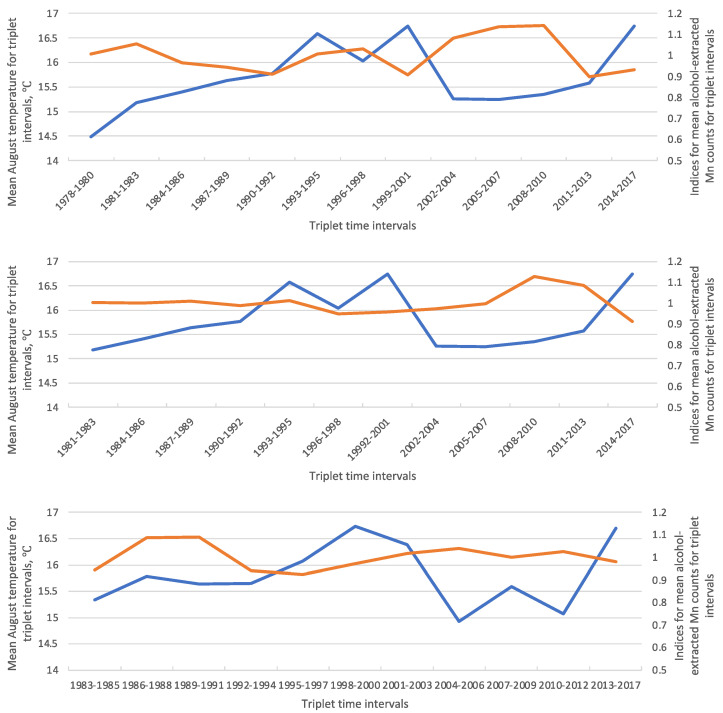
Example time series for August mean temperature and indices for alcohol-extracted Mn counts. Legend: blue lines are the course of the August mean temperatures (means for triplet year intervals), brown lines are the course of alcohol-extracted Mn indices for the triplet year intervals for correspondent Siberian pine trees.

A graphical representation of time series of this example correlation is given in Figure 1. As follows from the figure, the August mean temperatures and the counts for Mn after alcohol extraction evolve more or less contrarily to each other, which is most obviously seen in Siberian pine #1 and corresponds to the negative sign of the correlation.

In the Larch species, four consistent correlations were found, three of which relate to early summer weather, May and June humidity, and June precipitations. Like in Spruce, Larch correlations relate only to those elements’ counts that were received after chemical treatment. This means that no correlations can be recorded in these species until the chemical treatment, alcohol and/or HCl, is applied. Of course, the elements’ spectrum studied in this research limits the inference.

Last, Scots pine had three consistent correlations, two of which relate to August weather, humidity, and temperature.

Table 2 provides the consistent correlations for the species based on the Kendall tau correlation coefficient. Kendall tau looks more demanding to the data because many pairs of ‘weather parameter–element counts’ do not show consistent correlations. This led to only two species, Siberian pine and Scots pine, remaining on the list of consistent correlations. Nevertheless, the results of Table 2 correspond completely to those of Table 1. For example, Scots pine shows a consistent correlation for the pair ‘August humidity–original Sr content’ in both tables. The same is true for the consistent correlations in Siberian pine (cf. Table 1 and Table 2). A feature of the Kendall tau coefficient is that only the original elemental contents show consistent correlations.

In the tables, the same species are highlighted by the same color.

In Appendix A, the tables summarize data on elemental composition of all the trees under study. The summarized data are mean elemental counts in cores subjected to no treatment (Table A1), after extraction in alcohol (Table A2), and after extraction in HCl (Table A3). The data provide an idea of the variability of the elemental counts within and between species under the treatment conditions.

## 3. Discussion

Referring to the first part of our hypothesis, we can see that correlations connecting the elemental counts in tree-rings and weather parameters may really exist—although a great number of correlations present only statistical noise. In this respect, the results received support the hypothesis. A question still remains of how consistent these correlations are among species and individual trees.

The comparison of all the species studied answered whether the elemental contents in all the species can react consistently to any weather parameters. We found no one pair of the relation ‘weather parameter–element content’ that showed a consistent correlation across the species. This means that the right choice of species for dendrochemical research is of great importance because what is not seen in one species may be revealed in another.

To some extent, the requirements imposed on species for a dendrochemistry study may be weakened by using a chemical treatment approach. As follows from the data presented, the correlations may not be found in some species unless a chemical treatment is applied (see Spruce and Larch data in Table 1). Therefore, the results also supported the second part of the hypothesis—regarding the role of the chemical treatment. Thus, chemical treatment can reveal the reaction of tree rings’ elemental content to some weather parameters. However, the correlations may disappear after the treatments. Elements such as Sr, P, and Ti in Siberian pine provide an example of this behavior. We found the correlations of the elements in the species in the untreated cores only (Table 1 and Table 2).

It must be noted that the research comes laden with an important implication. The use of May–August weather data implies that the deposition of the elements in the xylem occurs over the course of growth or immediately after its cessation. There is no firm proof of this and the supposition is founded on common sense anticipating that such behavior may be more likely.

Hypothetically, the cause of better correlation between a climatic signal and elemental content after chemical treatment is because of the treatment removing elements occurring in the inter-cell space. These are mostly pectinates and oxalates of various metal elements. The accumulation and translocation of the compounds result from complicated processes, many of which take place far later than the time of tree ring formation. Because of this, they may distort possible relationships between climatic parameters at the time of the ring growth and the elements’ deposition in the xylem. It is noteworthy to mention that no crystal-like structures similar to those reported by Hietz et al. [14] were registered on X-ray images in the trees sampled.

In the search for consistent correlations, we adopted a rule that there should be at least one significant (*p* < 0.05) coefficient among the trees of one species. This is because not a single case was found where all the three coefficients were significant at this level. In part, the problem of significance is a problem of the sampling size. In our case, the number of the triplets was not over 10–13, which may be not enough for this kind of data. Nevertheless, the very fact of the significance of some coefficients provides evidence that the significance may be improved with longer data, i.e., older trees. In this research, we focused on data from the afforestation experiment that were beneficial from the viewpoint of species collection but provided only relatively young trees for the analysis.

## 4. Materials and Methods

### 4.1. Species and Geographical Location

**Figure 2:**
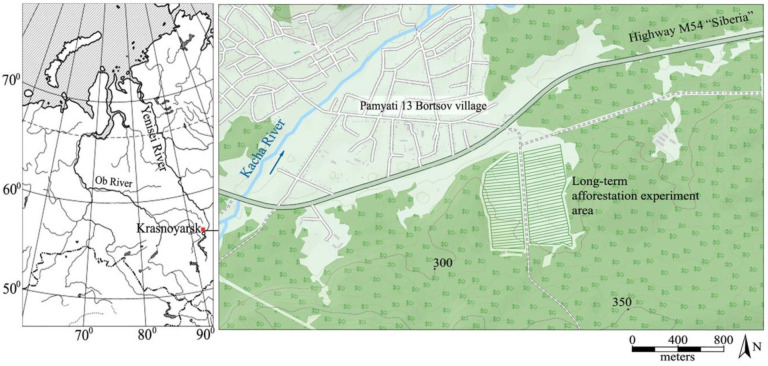
Location and situation plan of study area. The red square on the left is the geographical position of the study area.

For the study, four widely spread Siberian conifer species were taken. They are Siberian spruce (*Picea obovata* Ledeb.), Scots pine (*Pinus sylvestris* L.), Siberian larch (*Larix sibirica* Ledeb.), and Siberian pine (*Pinus sibirica* Du Tour). The trees grow in pure even-aged forest stands with an initial planting density of 40,000 saplings per ha. The trees were planted as 2–3-year-old seedlings in 1971–1972 as a part of an afforestation experiment [15,16,17]. The location of the experiment area is shown in Figure 2.

### 4.2. Sampling

In October 2017, from each species, we took three trees without apparent damage for sampling. We extracted a 12 mm core from every sample tree at breast height. The cores were air-dried for ca. two weeks under room conditions in a laboratory. Then, a circular saw was applied to produce 2 mm thick slices from the cores perpendicular to the wood grain. Altogether, 12 cores were treated in the study.

### 4.3. X-ray Scanning

Itrax Multiscanner (COX Analytical Systems) coupled with Multiscanner Navigator software was applied to scan the wooden slices to estimate the content of a spectrum of chemical elements. In principle, the multi-scanner can detect a lot of elements, but not any lighter than Al. We chose several elements that are known to be or maybe biologically active. They are Al, P, S, K, Ca, Ti, Mn, Fe, Cu, Zn, and Sr. The spatial resolution along the slices was 100 µm, with the width of the scanning beam being 2 mm. The multi-scanner outputs are the so-called counts or count rates, which are relative dimensionless units.

The Ca count rates are of special significance to dendrochemical studies because Ca is reported to have a sharp peak in the very early cells of a tree ring [18,19]. It can therefore be used, in parallel with optical analysis, to separate a count rate sequence into series corresponding to individual tree rings.

### 4.4. Chemical Treatment

To test the above hypothesis, we performed a double chemical treatment of the wooden slices. Preliminarily, the first scanning of the slices took place under the original condition in 2017.

In 2021, the slices were subjected to extraction in pure alcohol at a boiling temperature of 78 °C, the time of the extraction being eight hours. After treatment, the slices were air-dried in a desiccator at room temperature above a silica gel layer. To avoid warpage of the slices, the final drying went under pressure in a drying oven under 60 °C. A second scan followed the drying stage.

The slices were then subjected to a treatment in 0.2M HCl at 60 °C for three hours, which was followed by threefold washing off in bi-distilled water. After the drying procedure as given above, we scanned the slices for a third time.

The HCl concentration was 0.2M despite that the standard methods recommend 0.5M [20] removing the Ca carbonate and even up to 2.0M for removing Ca oxalate. We used a lower HCl concentration to ensure the wood integrity for the further X-ray fluorescence analysis. According to Schilling [21], an application of 0.2M HCl allows for the complete removal of the Ca oxalates.

As a result, each element studied in each slice had three sequences of count rates: original, after alcohol extraction, and after HCl.

### 4.5. Weather Data

We used a few meteorological parameters to relate to the dendrochemical properties of the tree rings. The meteorological data were available from the open database of the Krasnoyarsk Weather Station, WMO ID = 29570. Numerous studies [22,23,24] have shown that May to August is the time of the year when the growth of cambium and cell differentiation take place.

The list of the weather parameters included: (1) mean monthly humidity (from May to August), (2) mean monthly temperature (from May to August), (3) monthly sum of precipitations (from June to August), and (4) mean monthly wind speed (from May to August). We did not use the May precipitations in the analysis on the following grounds. In the study area, the climate is classified as Dfb [25]. In some years, the May precipitations are solid precipitations (snow) because of low temperature. Only from June to August are the mean daily temperatures sure to be above +5 °C.

Wind speed is not a usual parameter in dendroclimatic studies. However, there are some grounds for considering wind speed. Given high air temperatures in the summer, the wind increases evaporation and therefore increases the uprising flux of soil solutions. It is the soil solutions that bear the ions up the tree trunk where they can be absorbed by the growing xylem.

Moreover, a compound humidity index by Vysotskii–Ivanov has been calculated for June to August. The humidity index (HI) is as follows:

HI = R/Ep,

where R is the monthly sum of precipitation and Ep is the monthly evaporation.

The humidity index was used in two forms, ‘as is’, and as subjected to detrending (see Statistical Analysis subsection below).

Altogether, we included 21 various weather parameters in the analysis.

### 4.6. Statistical Analysis

**Figure 3:**
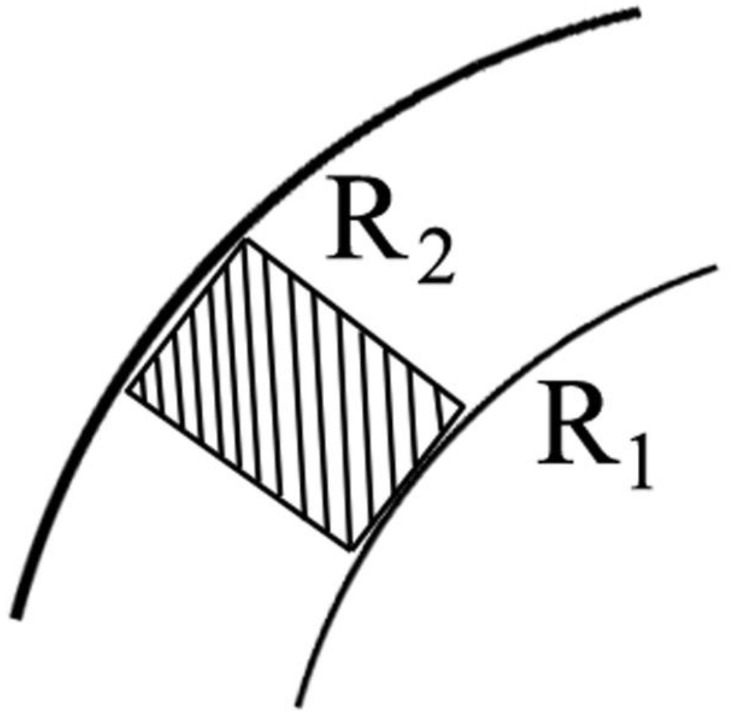
Count concentration geometry. R1 and R2 are radii of older and younger tree rings, correspondingly. Count concentration is calculated per mm^2^ of the dashed rectangle with a width of 2 mm (width of the scanning beam). Count quantity is calculated for the area between R1 and R2 circumferences.

Elemental content parameter: As is known, the counts originate from a scanning band of 2 mm that crosses the tree rings. For the current analysis, the number of counts falling on average into 1 mm^2^ within a tree ring was used. That is, the parameter is an analogue of count concentration per 1 mm^2^ in a tree ring. Figure 3 provides a geometrical presentation of the approach.

#### 4.6.1. Detrending

The series of elements’ counts in every tree were subjected to detrending as is normally performed in dendrochronological studies. In fact, there is no clear understanding of if the detrending should apply to the elements’ counts. However, the need for detrending may have the same grounds as with the tree-ring widths. Tree-ring widths are detrended because of the non-linear growth curve. The counts of some elements also have long-term trends [12,13]. The best example is Ca, which decreases from the pith to the bark. That is why detrending serves to avoid the creation of illusory correlations not really related to climate.

Detrending was performed using ARSTAN software (by Dr. Edward R. Cook, Columbia University, New York, NY, USA and Paul J. Krusic, University of Cambridge, Cambridge, UK), with the detrending function being the Hugershoff curve. As the result of detrending, the absolute count values are transformed into indices oscillating around the unity.

#### 4.6.2. Correlation Calculations

To estimate relationships between elements’ contents and weather parameters, the non-parametrical Spearman rho coefficient and Kendall tau coefficient have been calculated between all the available pairs ‘weather parameter—element counts’ for the triplets of the calendar years from 2017 to 1978/79. The number of the years varied a bit because of the variability in trees’ growth: the number of tree rings at breast height in different trees was slightly different. Non-parametrical correlation coefficients were measured because parametrical approaches require the normal distribution of data, while the amount of data available was rather low. The latter was in part because instead of using the values of individual years/tree rings, we made use of mean values (weather parameters and elemental counts) for triplets of consecutive years, for example, from 1978 to 1980, 1981 to 1983, etc. If the number of tree-rings could not be divided by three exactly then the outermost narrow rings were taken as a group of four or five. Consequently, the number of triplets varied from 10 to 13, which also means that the series of triplet years may be slightly different for the individual trees (see Figure 1). A preliminary study showed that the use of individual years produces mostly low correlations. We discuss the problem below.

Both coefficients, Spearman and Kendall, are non-parametric approaches. However, the mathematics of the coefficients are different and therefore provide slightly different results. One coefficient may be more sensitive/demanding than the other. The use of both coefficients serve the purpose of possessing two competing viewpoints on the data.

All the correlation calculations were performed with the help of Statistica 6 software.

## 5. Conclusions

A common feature of dendrochemical data received through X-ray fluorescence methods is the great variability of elements’ counts between different individual trees. It is often hard to see anything consistent in the data because one even very strong correlation in one tree may be absolutely absent in another tree or, what is most confusing, may have the opposite sign in another tree. Plenty of examples for such elemental behavior may be found in the matrices provided in the electronic Supplementary Material. Only relationships repeatedly found in a substantial number of trees may be the basis of further interpretations and inferences.

Another source of unwanted variability relates to the method of X-ray fluorescence and the possible drift of elements across the tree trunks. The accuracy of spatial resolution, which is especially important for narrow tree rings, or the distribution of elements among the neighboring rings, create a noise that may obscure the correlations sought. As mentioned above, the calculations for individual tree rings, i.e., individual years, result in very weak correlations—possibly because of the noise. An aggregation of the data into consecutive time sections (triplets in our case) greatly improves the correlations. Probably, sometimes such aggregation is the only solution when searching for relationships. An obvious weakness of the triplet approach is that it dramatically reduces the amount of data and may result in a statistical significance problem.

Because the correlations react to chemical treatment, one may suppose that the elements’ arrangement in the xylem is a multi-layered structure. A consecutive extraction of the most movable forms of the elements should provide a path to revealing the forms of the elements that are deeper and more tightly bound to the xylem. However, it is not granted that the deeper-located elements will show the correlations sought.

In this study, we sought consistent correlations between weather parameters and the content of a range of elements in the tree rings. We conducted no discussion on why one or another element’s content should react to weather during the growing season of tree rings. Such mechanisms, according to our best knowledge, are not known today. However, consistent correlations found within the four conifer species may help one concentrate the research on reliable data.

## Figures and Tables

**Table 1:** Consistent correlations for the conifer species according to Spearman rho correlation matrices.

Weather par	alcCa *	orgSr	alcK	orgP	alcS	orgTi	hclTi	alcMn	hclCu	alcZn
Iv_jn_ind **						*Sib.pine*(+)				
Iv_jl_ind										
Iv_au_ind										
Iv-Vys_jn						*Sib. pine*(+)				
Iv-Vys_jl										
Iv-Vys_aug										
Hum_may					Larch(−)					
Hum_jn									Larch(−)	
Hum_jl			Spruce(−)							
Hum_aug		*Sc. pine*(−)		*Sib. pine*(−)						
T_may										*Sc. pine*(−)
T_jn										
T_jl										
T_aug			*Sc. pine*(+)					*Sib. pine*(−)		
Prec_jn						*Sib. pine*(+)			Larch(−)	
Prec_jl										
Prec_aug										
Wind_my										
Wind_jn										
Wind_jl	Spruce(−) ^†^									
Wind_aug							Larch(−)			

* org = original content of the element; alc = content of element after alcohol extraction; hcl = content of element after HCl treatment. ** Iv_jn_ind, Iv_jl_ind, Iv_au_ind = Vysotskii–Ivanov humidity index detrended for June, July, and August, correspondingly; Iv-Vys_jn, Iv-Vys_jl, Iv-Vys_aug = Vysotskii–Ivanov humidity index in original form for June, July, and August, correspondingly; Hum_may, Hum_jn, Hum_jl, Hum_aug = mean humidity for May, June, July, and August, correspondingly; T_may, T_jn, T_jl, T_aug = mean temperature for May, June, July, and August, correspondingly; Prec_jn, Prec_jl, Prec_aug = monthly sum of precipitations for June, July, and August, correspondingly; Wind_my, Wind_jn, Wind_jl, Wind_aug = mean wind speed for May, June, July, and August, correspondingly. ^†^ (−), (+) denote negative and positive relationships, correspondingly.

**Table 2:** Consistent correlations for the conifer species according to Kendall tau correlation matrices.

Weather par	orgSr *	orgP	orgTi
Iv_jn_ind **			*Sib. pine*(+)
Iv_jl_ind			
Iv_au_ind			
Iv-Vys_jn			*Sib. pine*(+)
Iv-Vys_jl			
Iv-Vys_aug			
Hum_may			
Hum_jn			
Hum_jl			
Hum_aug	*Sc. pine*(−) ^†^	*Sib. pine*(−)	
T_may			
T_jn			
T_jl			
T_aug			
Prec_jn			
Prec_jl			
Prec_aug			
Wind_my			
Wind_jn			
Wind_jl			
Wind_aug			

* as in Table 1. ** as in Table 1. ^†^ as in Table 1.

## Data Availability

Data available on request.

## Supplementary Materials

The following supporting information can be downloaded at: https://www.mdpi.com/article/10.3390/plants11233240/s1, Table S1: A Spearman rho correlation matrix for Spruce #1; Table S2: A Kendall tau correlation matrix for Spruce #1; Table S3: A Spearman rho correlation matrix for Spruce #2; Table S4: A Kendall tau correlation matrix for Spruce #2; Table S5: A Spearman rho correlation matrix for Spruce #3; Table S6: A Kendall tau correlation matrix for Spruce #3; Table S7: A Spearman rho correlation matrix for Siberian pine #1; Table S8: A Kendall tau correlation matrix for Siberian pine #1; Table S9: A Spearman rho correlation matrix for Siberian pine #2; Table S10: A Kendall tau correlation matrix for Siberian pine #2; Table S11: A Spearman rho correlation matrix for Siberian pine #3; Table S12: A Kendall tau correlation matrix for Siberian pine #3; Table S13: A Spearman rho correlation matrix for Siberian larch #1; Table S14: A Kendall tau correlation matrix for Siberian larch #1; Table S15: A Spearman rho correlation matrix for Siberian larch #2; Table S16: A Kendall tau correlation matrix for Siberian larch #2; Table S17: A Spearman rho correlation matrix for Siberian larch #3; Table S18: A Kendall tau correlation matrix for Siberian larch #3; Table S19: A Spearman rho correlation matrix for Scots pine #1; Table S20: A Kendall tau correlation matrix for Scots pine #1; Table S21: A Spearman rho correlation matrix for Scots pine #2; Table S22: A Kendall tau correlation matrix for Scots pine #2; Table S23: A Spearman rho correlation matrix for Scots pine #3; Table S24: A Kendall tau correlation matrix for Scots pine #3; Table S25: Scots pine #1. Initial core. After alcohol extraction. After HCl extraction; Table S26: Scots pine #2. Initial core. After alcohol extraction. After HCl extraction; Table S27: Scots pine #3. Initial core. After alcohol extraction. After HCl extraction.

## Appendix A

**Table A1:** Elemental contents in trees under study, mean counts for no treatment case.

Species	Range of Years	Al	P	S	Ti	Mn	Fe	Cu	Zn	Ca	Sr	K
*P. sylvestris* #1	1978–2017	8.9	10.1	28.2	1.7	197.0	78.3	138.2	158.6	2681.3	257.8	572.7
*P. sylvestris* #2	1978–2017	9.6	12.0	31.4	1.9	343.4	84.9	172.7	192.8	2760.2	225.5	674.2
*P. sylvestris* #3	1978–2017	9.5	10.6	30.0	2.4	233.2	99.3	133.2	149.7	2635.0	276.7	526.7
*P. sibirica* #1	1978–2017	9.1	12.9	33.4	1.8	181.1	78.5	156.6	143.2	1876.0	280.1	590.5
*P. sibirica* #2	1981–2017	8.4	13.6	38.6	1.6	148.3	89.3	193.7	169.7	1994.5	269.3	683.7
*P. sibirica* #3	1983–2017	8.1	13.3	38.1	2.3	177.3	103.6	189.7	173.4	1972.7	261.1	551.3
*P. obovata* #1	1979–2017	10.1	8.8	26.8	7.1	261.8	75.7	141.6	154.1	3946.8	313.0	749.4
*P. obovata* #2	1979–2017	10.3	8.5	26.0	11.8	344.5	62.4	131.5	181.4	3798.2	274.4	739.8
*P. obovata* #3	1979–2017	9.9	8.6	27.0	4.8	283.0	79.3	153.8	176.8	4005.0	260.2	667.4
*L. sibirica* #1	1983–2017	9.6	11.1	30.2	3.7	155.5	68.0	162.2	154.0	3294.9	174.0	812.9
*L. sibirica* #2	1978–2017	7.6	12.9	33.4	5.7	157.8	68.7	189.1	206.0	1953.2	121.8	400.7
*L. sibirica* #3	1978–2017	9.6	10.2	28.7	5.0	171.3	63.5	149.5	161.2	2720.4	187.8	818.9

**Table A2:** Elemental contents in trees under study, mean counts after alcohol extraction.

Species	Range of Years	Al	P	S	Ti	Mn	Fe	Cu	Zn	Ca	Sr	K
*P. sylvestris* #1	1978–2017	53.2	12.5	6.7	14.0	198.8	77.7	256.7	295.1	540.1	102.4	94.3
*P. sylvestris* #2	1978–2017	49.0	7.2	3.7	15.3	279.3	87.9	195.4	252.4	390.4	169.3	73.8
*P. sylvestris* #3	1978–2017	50.4	8.6	4.5	14.4	206.1	107.1	110.7	190.9	420.8	100.4	72.6
*P. sibirica* #1	1978–2017	53.8	12.8	7.3	14.5	181.7	76.9	159.0	214.4	375.8	43.5	108.4
*P. sibirica* #2	1981–2017	49.2	8.2	4.1	15.5	145.8	106.1	150.9	218.0	334.7	110.9	98.5
*P. sibirica* #3	1983–2017	53.1	12.2	6.4	16.4	185.0	121.9	161.7	277.7	412.5	85.6	109.4
*P. obovata* #1	1979–2017	51.9	9.9	5.0	15.4	248.6	77.7	134.8	225.9	662.0	91.3	112.8
*P. obovata* #2	1979–2017	51.2	9.1	4.7	17.3	291.4	59.0	216.6	266.0	565.7	137.8	101.9
*P. obovata* #3	1979–2017	47.2	4.6	2.2	15.4	223.4	63.7	80.3	167.0	448.4	163.5	68.2
*L. sibirica* #1	1983–2017	54.6	16.1	8.5	13.9	154.6	67.7	155.8	254.0	602.0	53.7	125.5
*L. sibirica* #2	1978–2017	53.0	12.7	6.4	14.1	150.6	56.6	112.3	218.9	286.0	88.2	83.4
*L. sibirica* #3	1978–2017	50.9	9.5	4.2	12.8	145.2	45.4	82.0	172.2	335.9	101.4	81.2

**Table A3:** Elemental contents in trees under study, mean counts after HCl extraction.

Species	Range of Years	Al	P	S	Ti	Mn	Fe	Cu	Zn	Ca	Sr	K
*P. sylvestris* #1	1978–2017	43.9	3.9	1.4	15.0	72.3	63.8	85.0	109.4	55.4	192.1	10.3
*P. sylvestris* #2	1978–2017	35.3	0.8	0.1	14.2	78.5	64.4	51.8	64.0	39.9	288.5	8.6
*P. sylvestris* #3	1978–2017	38.2	1.6	0.3	14.6	80.4	62.7	60.1	62.8	34.9	246.0	8.3
*P. sibirica* #1	1978–2017	44.5	3.5	1.2	16.1	86.0	71.6	114.2	79.8	49.6	196.3	9.4
*P. sibirica* #2	1981–2017	38.4	1.8	0.4	15.4	78.2	69.5	98.3	93.6	43.4	260.7	12.7
*P. sibirica* #3	1983–2017	48.5	5.7	2.3	16.4	83.9	91.2	119.8	152.7	56.1	247.1	15.1
*P. obovata* #1	1979–2017	44.7	4.0	1.8	14.5	86.9	71.9	115.9	85.4	56.9	207.0	9.9
*P. obovata* #2	1979–2017	38.1	0.8	0.2	13.0	78.8	51.4	50.1	59.1	38.3	244.8	7.5
*P. obovata* #3	1979–2017	32.8	0.5	0.1	13.7	79.2	51.6	55.0	43.1	33.9	314.2	7.7
*L. sibirica* #1	1983–2017	44.3	5.3	2.2	13.6	66.0	62.7	99.4	115.8	56.1	188.7	11.0
*L. sibirica* #2	1978–2017	36.8	1.8	0.5	12.6	69.6	45.0	84.8	78.6	42.9	261.2	9.8
*L. sibirica* #3	1978–2017	37.8	1.4	0.3	13.4	70.5	50.3	61.5	56.7	59.7	235.1	9.7

## References

[1] Binda G., Di Iorio A., Monticelli D. (2021). The what, how, why, and when of dendrochemistry: (paleo) environmental information from the chemical analysis of tree rings. Sci. Total Environ..

[2] Watmough S.A. (1997). An evaluation of the use of dendrochemical analyses in environmental monitoring. Environ. Rev..

[3] Watmough S.A. (1999). Monitoring historical changes in soil and atmospheric trace metal levels by dendrochemical analysis. Environ. Pollut..

[4] Lageard J.G.A., Howell J.A., Rothwell J.J., Drew I.B. (2008). The utility of Pinus sylvestris L. in dendrochemical investigations: Pollution impact of lead mining and smelting in Darley Dale, Derbyshire, UK. Environ. Pollut..

[5] Austruy A., Yung L., Ambros J.P., Girardclos O., Keller C., Angeletti B., Chalot M. (2019). Evaluation of historical atmospheric pollution in an industrial area by dendrochemical approaches. Chemosphere.

[6] Balouet C., Burken J., Martelain J., Lageard J., Karg F., Megson D. (2021). Dendrochemical forensics as material evidence in courts: How could trees lie?. Environ. Forensics.

[7] Xu G., Liu X., Qin D., Chen T., Sun W., An W., Ren J. (2014). Drought history inferred from treering δ13C and δ18O in the central Tianshan Mountains of China and linkage with the North Atlantic Oscillation. Theor. Appl. Climatol..

[8] Witt G.B., English N.B., Balanzategui D., Hua Q., Gadd P., Heijnis H., Bird M.I. (2017). The climate reconstruction potential of Acacia cambagei (gidgee) for semi-arid regions of Australia using stable isotopes and elemental abundances. J. Arid Environ..

[9] Sánchez-Salguero R., Camarero J.J., Hevia A., Sangüesa-Barreda G., Galván J.D., Gutiérrez E. (2019). Testing annual tree-ring chemistry by X-ray fluorescence for dendroclimatic studies in high-elevation forests from the Spanish Pyrenees. Quat. Int..

[10] McCarroll D., Loader N.J. (2004). Stable isotopes in tree rings. Quat. Sci. Rev..

[11] Cutter B.E., Guyette R.P. (1993). Anatomical, chemical, and ecological factors affecting tree species choice in dendrochemistry studies. J. Environ. Qual..

[12] Gavrikov V.L., Fertikov A.I., Sharafutdinov R.A., Vaganov E.A. (2021). Species-specific and Non-species-specific Elemental Trends in Tree Rings. Dokl. Earth Sci..

[13] Gavrikov V.L., Fertikov A.I., Sharafutdinov R.A., Vaganov E.A. (2021). Variability in Elemental Composition of Conifer Tree Rings. LesnoyZhurnal [Russ. For. J.].

[14] Hietz P., Horsky M., Prohaska T., Lang I., Grabner M. (2015). High-resolution densitometry and elemental analysis of tropical wood. Trees.

[15] Schugalei L.S., Semechkina M.G., Yashihin G.I., Dmitrienko V.K. (1995). Modeling of Artificial Forest Biogeocenoses’ Development.

[16] Menyailo O.V., Hungate B.A., Zech W. (2002). Tree species mediated soil chemical changes in a Siberian artificial afforestation experiment. Plant Soil.

[17] Schugalei L.S., Binkley D., Menyailo O. (2005). The Siberian afforestation experiment: History, methodology, and problems. Tree Species Effects on Soils: Implications for Global Change.

[18] Silkin P.P., Ekimova N.V. (2012). Relationship of strontium and calcium concentrations with the parameters of cell structure in Siberian spruce and fir tree-rings. Dendrochronologia.

[19] Scharnweber T., Hevia A., Buras A., van der Maaten E., Wilmking M. (2016). Common trends in elements? Within- and between-tree variations of wood-chemistry measured by X-ray fluorescence—A dendrochemical study. Sci. Total Environ..

[20] Dumoulin J.P., Comby-Zerbino C., Delqué-Količ E., Moreau C., Caffy I., Hain S., Beck L. (2017). Status report on sample preparation protocols developed at the LMC14 Laboratory, Saclay, France: From sample collection to 14C AMS measurement. Radiocarbon.

[21] Schilling J.S. (2006). Oxalate Production and Cation Translocation during Wood Biodegradation by Fungi. Ph.D. Thesis.

[22] Vaganov E.A., Hughes M.K., Shashkin A.V. (2006). Growth Dynamics of Conifer Tree Rings: Images of Past and Future Environments.

[23] Anchukaitis K.J., Breitenmoser P., Briffa K.R., Buchwal A., Büntgen U., Cook E.R., D’Arrigo R.D., Esper J., Evans M.N., Frank D. (2012). Tree rings and volcanic cooling. Nat. Geosci..

[24] Fonti M.V., Saurer M., Guillet S., Corona C., Fonti P., Myglan V.S., Kirdyanov A.V., Naumova O.V., Ovchinnikov D.V., Shashkin A.V. (2019). Siberian tree-ring and stable isotope proxies as indicators of temperature and moisture changes after major stratospheric volcanic eruptions. Clim. Past.

[25] Kottek M., Grieser J., Beck C., Rudolf B., Rubel F. (2006). World map of the Köppen-Geiger climate classification updated. Meteorol. Z..

