# Supplementary material for: Dendrochemical Challenge in Climate Science: Whether Chemical Elements in Wood Reflect the Fluctuations in Weather Parameters
Source: Plants (Basel). 2022 Nov 25;11(23):3240. doi: 10.3390/plants11233240 (PMC9740880; doi:10.3390/plants11233240)
Supplement: Supplementary file 1 [file plants-11-03240-s001.zip › Supplementary_material_for_Plants.pdf]

Dendrochemical challenge in climate science: whether chemical elements in wood reflect the fluctuations in weather parameters
Supplementary material

Table S1. A Spearman rho correlation matrix for Spruce #1.

Weather par**	orgCa*	alcCa	hclCa	orgSr	alcSr	hclSr	orgK	alcK	hclK	orgAl	alcAl	hclAl
**Iv_jn_ind**	0.08	-0.01	-0.26	0.08	0.15	0.04	-0.18	-0.27	0.08	0.14	0.18	-0.19
**Iv_jl_ind**	-0.24	-0.26	-0.38	-0.06	0.17	-0.15	-0.34	-0.34	0.02	-0.21	-0.19	-0.29
**Iv_au_ind**	**0.59**†	**0.58**	0.22	-0.04	0.13	0.03	0.24	-0.11	0.34	0.00	0.02	-0.15
**Iv-Vys_jn**	-0.05	-0.15	-0.30	0.09	0.23	0.00	-0.21	-0.35	-0.04	0.13	0.13	-0.18
**Iv-Vys_jl**	-0.24	-0.27	-0.29	0.04	0.30	-0.18	-0.26	-0.33	0.09	-0.20	-0.13	-0.36
**Iv-Vys_aug**	0.52	0.45	0.01	-0.10	0.07	-0.13	0.01	-0.24	0.15	-0.06	-0.04	-0.08
**Hum_may**	0.30	0.31	-0.05	0.49	0.18	0.06	-0.03	-0.03	0.12	-0.19	-0.34	-0.31
**Hum_jn**	-0.37	-0.38	-0.31	-0.38	0.05	0.06	-0.35	-0.34	-0.54	-0.09	-0.08	0.45
**Hum_jl**	-0.41	-0.46	-0.43	0.29	0.31	0.08	-0.38	-0.49	-0.23	**-0.57**	-0.48	-0.19
**Hum_aug**	0.35	0.23	-0.17	0.23	0.48	-0.25	-0.18	-0.37	0.19	-0.09	0.04	-0.30
**T_may**	-0.53	-0.51	-0.08	-0.53	-0.32	0.05	-0.08	-0.10	-0.32	-0.21	0.02	0.43
**T_jn**	-0.10	-0.17	-0.42	-0.13	-0.07	-0.09	-0.36	-0.41	-0.20	-0.41	-0.19	0.18
**T_jl**	0.53	0.43	0.30	0.23	-0.31	0.27	0.29	0.20	-0.07	0.23	-0.24	-0.04
**T_aug**	-0.21	-0.02	0.33	0.05	-0.23	0.05	0.40	0.37	0.34	0.05	0.16	0.13
**Prec_jn**	0.06	-0.05	-0.27	0.19	0.16	0.05	-0.15	-0.33	0.00	0.07	0.03	-0.28
**Prec_jl**	-0.36	-0.41	-0.33	-0.23	0.06	-0.34	-0.32	-0.23	0.04	-0.04	0.10	-0.16
**Prec_aug**	0.53	0.53	0.19	0.02	-0.10	0.05	0.21	-0.01	0.28	-0.09	-0.14	-0.10
**Wind_my**	0.21	0.16	0.40	-0.15	-0.01	0.20	0.31	0.07	-0.03	0.17	0.02	0.14
**Wind_jn**	-0.08	0.02	0.36	0.01	0.23	0.21	0.31	0.17	0.09	0.27	0.12	0.06
**Wind_jl**	-0.47	-0.45	0.19	0.10	0.08	-0.28	0.16	0.22	0.24	0.08	0.21	-0.23
**Wind_aug**	-0.09	-0.02	0.49	0.19	0.01	0.15	0.36	0.44	0.02	0.22	-0.11	-0.07

* org = original content of the element; alc = content of element after alcohol extraction; hcl = content of element after HCl treatment

** Iv_jn_ind, Iv_jl_ind, Iv_au_ind = Vysotskii-Ivanov humidity index detrended for June, July, and August, correspondingly; Iv-Vys_jn, Iv-Vys_jl, Iv-Vys_aug = Vysotskii-Ivanov humidity index in original form for June, July, and August, correspondingly; Hum_may, Hum_jn, Hum_jl, Hum_aug = mean humidity for May, June, July, and August, correspondingly; T_may, T_jn, T_jl, T_aug = mean temperature for May, June, July, and August, correspondingly; Prec_jn, Prec_jl, Prec_aug = monthly sum of precipitations for June, July, and August, correspondingly; Wind_my, Wind_jn, Wind_jl, Wind_aug = mean wind speed for May, June, July, and August, correspondingly

[†] the values marked by bold red font are significant at $p < 0.05$

Table S1. A Spearman rho correlation matrix for Spruce #1 (continued 1).

Weather par	orgP	alcP	hclP	orgS	alcS	hclS	orgTi	alcTi	hclTi	orgMn	alcMn	hclMn
**Iv_jn_ind**	0.06	-0.08	0.16	-0.14	**0.63**	-0.01	0.23	-0.18	-0.34	0.12	-0.05	-0.18
**Iv_jl_ind**	0.08	0.23	0.07	**-0.61**	-0.46	0.06	0.18	-0.04	-0.16	-0.01	0.02	0.18
**Iv_au_ind**	0.34	-0.11	-0.10	-0.13	-0.11	-0.41	0.12	-0.07	-0.08	-0.19	-0.35	-0.40
**Iv-Vys_jn**	0.04	-0.10	0.18	-0.06	**0.56**	-0.05	0.35	-0.25	-0.38	0.24	0.09	-0.09
**Iv-Vys_jl**	0.09	0.22	0.03	**-0.59**	-0.41	0.07	0.30	-0.04	-0.12	-0.03	0.02	0.19
**Iv-Vys_aug**	0.20	-0.11	-0.13	-0.25	-0.19	-0.47	0.02	-0.09	-0.25	-0.08	-0.27	-0.31
**Hum_may**	-0.04	-0.26	-0.27	0.08	-0.03	-0.03	-0.29	0.28	-0.01	-0.18	-0.19	0.24
**Hum_jn**	-0.07	-0.08	0.24	-0.05	-0.15	-0.02	0.04	-0.08	-0.44	0.32	0.15	-0.03
**Hum_jl**	-0.04	-0.19	-0.16	-0.25	-0.40	0.20	0.29	-0.09	-0.34	0.10	0.01	**0.70**
**Hum_aug**	0.01	-0.06	-0.16	-0.46	0.03	-0.28	0.06	0.19	-0.33	-0.09	-0.22	-0.10
**T_may**	0.19	0.03	0.04	0.02	-0.29	0.14	0.18	-0.36	-0.22	0.08	-0.09	0.01
**T_jn**	0.02	-0.15	-0.28	-0.13	-0.30	-0.37	0.01	-0.18	-0.38	0.18	0.01	0.38
**T_jl**	-0.06	-0.44	0.13	0.42	0.38	0.10	0.08	-0.40	0.04	0.08	-0.03	-0.20
**T_aug**	**0.68**	-0.21	-0.51	0.47	0.03	-0.10	-0.05	-0.21	0.31	-0.40	-0.39	-0.07
**Prec_jn**	0.04	-0.18	0.13	0.03	0.58	-0.03	0.35	-0.30	-0.37	0.20	0.03	-0.02
**Prec_jl**	0.08	0.38	0.08	-0.68	-0.32	0.07	0.21	-0.12	-0.11	0.00	0.07	0.01
**Prec_aug**	0.36	-0.23	-0.25	-0.02	-0.20	-0.34	-0.03	-0.16	-0.03	-0.25	-0.41	-0.24
**Wind_my**	0.07	-0.10	0.29	-0.02	-0.04	0.13	0.45	-0.35	0.05	0.06	-0.02	-0.35
**Wind_jn**	0.23	-0.04	0.21	0.13	0.07	0.17	0.28	-0.04	0.21	-0.06	0.00	-0.29
**Wind_jl**	0.26	0.23	-0.15	-0.26	-0.16	0.40	0.30	-0.14	0.27	-0.32	-0.15	0.01
**Wind_aug**	-0.01	0.00	0.19	0.11	-0.13	**0.58**	0.08	0.03	0.44	-0.21	-0.02	-0.13

Table S1. A Spearman rho correlation matrix for Spruce #1 (continued 2).

Weather par	orgFe	alcFe	hclFe	orgCu	alcCu	hclCu	orgZn	alcZn	hclZn
**Iv_jn_ind**	-0.24	0.07	-0.02	-0.05	0.01	0.27	0.25	0.20	-0.18
**Iv_jl_ind**	-0.19	-0.05	-0.55	0.07	0.02	0.36	0.31	0.22	-0.09
**Iv_au_ind**	0.12	0.31	-0.22	0.38	0.31	-0.12	0.25	0.54	0.04
**Iv-Vys_jn**	-0.25	0.04	0.05	-0.25	-0.10	0.19	0.05	0.00	-0.13
**Iv-Vys_jl**	-0.13	-0.01	-0.49	0.09	-0.01	0.47	0.35	0.27	-0.03
**Iv-Vys_aug**	-0.04	0.11	-0.35	0.27	0.16	-0.22	0.23	0.36	-0.10
**Hum_may**	0.16	0.18	-0.19	0.14	-0.07	0.26	0.31	0.02	-0.35
**Hum_jn**	-0.45	-0.23	0.18	-0.23	-0.13	-0.32	-0.41	-0.32	0.01
**Hum_jl**	-0.19	-0.23	-0.25	-0.41	**-0.56**	0.36	-0.02	-0.37	-0.14
**Hum_aug**	-0.16	0.14	-0.47	0.45	0.08	0.44	**0.65**	**0.55**	-0.35
**T_may**	-0.23	-0.49	0.33	-0.33	-0.26	**-0.59**	**-0.63**	-0.50	0.45
**T_jn**	-0.19	-0.20	-0.32	-0.42	-0.37	-0.38	-0.29	-0.44	-0.25
**T_jl**	0.13	-0.13	0.38	-0.31	-0.15	-0.24	-0.13	-0.20	0.13
**T_aug**	0.36	0.09	**0.60**	-0.08	0.13	**-0.74**	**-0.64**	-0.41	0.49
**Prec_jn**	-0.15	0.03	0.04	-0.33	-0.20	0.18	0.05	-0.08	-0.15
**Prec_jl**	-0.25	-0.20	-0.45	0.12	0.07	0.25	0.27	0.24	0.05
**Prec_aug**	0.16	0.13	-0.14	0.20	0.16	-0.35	0.07	0.21	0.07
**Wind_my**	-0.04	-0.10	0.28	0.02	0.07	-0.03	-0.05	0.27	0.46
**Wind_jn**	0.06	0.24	0.44	0.17	0.31	0.09	-0.12	0.26	0.40
**Wind_jl**	0.09	-0.27	0.20	0.19	0.00	0.23	0.10	0.08	0.53
**Wind_aug**	0.18	-0.09	0.44	0.23	0.14	0.28	0.08	0.15	0.48

Table S2. A Kendall tau correlation matrix for Spruce #1.

Weather par**	orgCa*	alcCa	hclCa	orgSr	alcSr	hclSr	orgK	alcK	hclK	orgAl	alcAl	hclAl
**Iv_jn_ind**	0.08	0.05	-0.21	0.03	0.13	0.03	-0.15	-0.18	0.08	0.15	0.13	-0.13
**Iv_jl_ind**	-0.18	-0.21	-0.26	-0.08	0.13	-0.13	-0.26	-0.28	0.03	-0.15	-0.13	-0.18
**Iv_au_ind**	**0.49**†	**0.46**	0.15	-0.13	0.13	-0.03	0.21	-0.08	0.23	0.00	0.03	-0.08
**Iv-Vys_jn**	-0.03	-0.05	-0.26	0.08	0.13	0.08	-0.21	-0.23	-0.03	0.10	0.13	-0.08
**Iv-Vys_jl**	-0.21	-0.23	-0.23	0.00	0.21	-0.15	-0.23	-0.26	0.05	-0.18	-0.10	-0.26
**Iv-Vys_aug**	0.41	0.33	0.03	-0.10	0.00	-0.10	0.08	-0.10	0.10	-0.08	0.00	-0.05
**Hum_may**	0.21	0.18	-0.03	0.36	0.15	0.10	-0.03	0.00	0.10	-0.13	-0.31	-0.26
**Hum_jn**	-0.23	-0.26	-0.26	-0.33	0.03	0.03	-0.21	-0.23	-0.38	-0.05	-0.03	0.33
**Hum_jl**	-0.31	-0.33	-0.33	0.21	0.21	0.05	-0.23	-0.36	-0.15	**-0.44**	-0.36	-0.10
**Hum_aug**	0.30	0.17	-0.12	0.19	0.37	-0.17	-0.12	-0.22	0.17	0.01	0.06	-0.25
**T_may**	-0.41	-0.38	-0.03	-0.36	-0.26	0.05	-0.03	-0.10	-0.21	-0.18	0.00	0.31
**T_jn**	-0.10	-0.13	-0.38	-0.15	-0.10	-0.10	-0.28	-0.36	-0.10	-0.28	-0.15	0.05
**T_jl**	0.38	0.31	0.21	0.13	-0.18	0.18	0.21	0.18	-0.03	0.26	-0.08	-0.03
**T_aug**	-0.10	0.03	0.23	0.05	-0.15	0.10	0.28	0.31	0.26	0.03	0.15	0.10
**Prec_jn**	0.05	-0.03	-0.18	0.10	0.10	0.05	-0.13	-0.21	0.00	0.03	0.05	-0.15
**Prec_jl**	-0.26	-0.28	-0.23	-0.15	0.00	-0.26	-0.23	-0.21	0.05	-0.13	0.05	-0.10
**Prec_aug**	0.38	0.36	0.15	-0.03	-0.08	0.03	0.21	0.03	0.23	-0.10	-0.08	-0.08
**Wind_my**	0.12	0.09	0.27	-0.09	-0.01	0.12	0.22	0.01	-0.01	0.19	0.04	0.09
**Wind_jn**	-0.01	0.01	0.30	0.01	0.22	0.19	0.25	0.12	0.06	0.17	0.06	0.04
**Wind_jl**	-0.38	-0.38	0.20	0.04	0.04	-0.22	0.09	0.17	0.20	0.07	0.20	-0.17
**Wind_aug**	-0.09	-0.04	0.30	0.20	0.04	0.09	0.20	0.33	0.04	0.17	-0.07	-0.07

* as in Table S1

** as in Table S1

† as in Table S1

Table S2. A Kendall tau correlation matrix for Spruce #1 (continued 1).

Weather par	orgP	alcP	hclP	orgS	alcS	hclS	orgTi	alcTi	hclTi	orgMn	alcMn	hclMn
**Iv_jn_ind**	0.00	0.00	0.13	-0.08	0.41	-0.03	0.15	-0.13	-0.23	0.10	-0.05	-0.13
**Iv_jl_ind**	0.00	0.15	0.03	**-0.44**	-0.31	0.08	0.10	0.03	-0.13	0.00	0.05	0.13
**Iv_au_ind**	0.21	-0.05	-0.13	-0.08	-0.05	-0.18	0.10	-0.03	-0.08	-0.15	-0.26	-0.28
**Iv-Vys_jn**	0.05	-0.05	0.13	-0.08	0.36	-0.03	0.21	-0.18	-0.28	0.15	0.10	-0.08
**Iv-Vys_jl**	0.03	0.18	0.00	-0.41	-0.28	0.05	0.23	0.00	-0.10	-0.03	0.03	0.10
**Iv-Vys_aug**	0.18	-0.08	-0.05	-0.15	-0.18	-0.26	0.03	-0.05	-0.15	-0.08	-0.18	-0.21
**Hum_may**	-0.03	-0.23	-0.15	0.10	-0.03	-0.05	-0.23	0.21	0.00	-0.13	-0.13	0.15
**Hum_jn**	0.00	-0.05	0.08	0.03	-0.10	-0.03	0.05	-0.08	-0.33	0.15	-0.05	-0.03
**Hum_jl**	0.03	-0.13	-0.15	-0.15	-0.33	0.15	0.13	-0.05	-0.21	0.03	-0.03	**0.46**
**Hum_aug**	-0.01	-0.19	-0.06	-0.35	0.01	-0.17	0.09	0.12	-0.14	-0.04	-0.14	-0.09
**T_may**	0.13	0.03	0.00	0.00	-0.23	0.10	0.08	-0.21	-0.21	0.03	-0.08	-0.05
**T_jn**	-0.03	-0.13	-0.15	-0.05	-0.23	-0.31	0.03	-0.05	-0.36	0.13	0.03	0.26
**T_jl**	-0.05	-0.31	0.08	0.28	0.26	0.08	0.05	-0.28	0.03	0.05	-0.05	-0.08
**T_aug**	**0.49**	-0.13	-0.41	0.31	0.03	-0.05	-0.03	-0.15	0.26	-0.28	-0.23	-0.05
**Prec_jn**	0.03	-0.08	0.10	0.00	0.33	0.00	0.28	-0.21	-0.26	0.13	0.08	0.00
**Prec_jl**	0.03	0.28	0.05	**-0.46**	-0.23	0.05	0.13	-0.05	-0.10	-0.03	0.03	0.00
**Prec_aug**	0.26	-0.21	-0.18	-0.03	-0.15	-0.18	-0.05	-0.08	-0.03	-0.21	-0.31	-0.13
**Wind_my**	0.01	-0.06	0.22	0.04	-0.06	0.06	0.22	-0.17	-0.01	0.04	-0.01	-0.27
**Wind_jn**	0.19	-0.06	0.14	0.09	0.09	0.12	0.17	-0.04	0.17	-0.09	0.01	-0.19
**Wind_jl**	0.17	0.17	-0.12	-0.20	-0.09	0.33	0.25	-0.12	0.17	-0.22	-0.14	0.01
**Wind_aug**	-0.01	0.01	0.09	0.07	-0.07	**0.43**	0.04	-0.01	0.27	-0.14	0.01	-0.12

Table S2. A Kendall tau correlation matrix for Spruce #1 (continued 2).

Weather par	orgFe	alcFe	hclFe	orgCu	alcCu	hclCu	orgZn	alcZn	hclZn
**Iv_jn_ind**	-0.21	0.08	-0.05	-0.03	-0.05	0.21	0.21	0.13	-0.10
**Iv_jl_ind**	-0.10	-0.03	-0.31	0.03	0.00	0.26	0.26	0.13	-0.05
**Iv_au_ind**	0.10	0.23	-0.15	0.28	0.26	-0.05	0.10	0.38	0.05
**Iv-Vys_jn**	-0.26	-0.03	0.00	-0.13	-0.05	0.10	0.05	-0.03	-0.10
**Iv-Vys_jl**	-0.13	0.05	-0.28	0.05	-0.03	0.33	0.28	0.15	-0.03
**Iv-Vys_aug**	-0.03	0.10	-0.23	0.21	0.18	-0.13	0.08	0.26	-0.08
**Hum_may**	0.08	0.10	-0.13	0.10	-0.03	0.18	0.23	0.00	-0.28
**Hum_jn**	-0.36	-0.18	0.10	-0.13	-0.10	-0.21	-0.26	-0.23	0.00
**Hum_jl**	-0.03	-0.21	-0.23	-0.31	-0.38	0.33	-0.03	-0.15	-0.08
**Hum_aug**	-0.12	0.12	-0.37	0.32	0.04	0.27	**0.48**	0.40	-0.27
**T_may**	-0.13	-0.31	0.23	-0.21	-0.23	-0.38	**-0.44**	-0.31	0.28
**T_jn**	-0.08	-0.10	-0.28	-0.31	-0.23	-0.28	-0.18	-0.31	-0.23
**T_jl**	0.10	-0.13	0.26	-0.23	-0.05	-0.15	-0.10	-0.13	0.10
**T_aug**	0.18	0.05	**0.49**	-0.05	0.08	**-0.59**	**-0.49**	-0.31	0.33
**Prec_jn**	-0.18	0.00	0.03	-0.21	-0.13	0.13	0.03	-0.10	-0.13
**Prec_jl**	-0.13	-0.10	-0.23	0.10	0.03	0.13	0.18	0.15	0.08
**Prec_aug**	0.10	0.08	-0.10	0.18	0.15	-0.21	0.00	0.18	0.05
**Wind_my**	0.01	-0.06	0.12	-0.01	0.12	0.04	-0.01	0.27	0.32
**Wind_jn**	0.09	0.14	0.40	0.17	0.22	0.09	-0.12	0.22	0.35
**Wind_jl**	0.09	-0.22	0.20	0.14	0.04	0.17	0.04	-0.01	0.41
**Wind_aug**	0.07	-0.09	0.30	0.14	0.12	0.17	0.07	0.12	0.38

Table S3. A Spearman rho correlation matrix for Spruce #2.

Weather par**	orgCa*	alcCa	hclCa	orgSr	alcSr	hclSr	orgK	alcK	hclK	orgAl	alcAl	hclAl
**Iv_jn_ind**	-0.14	-0.29	0.50	0.26	-0.33	-0.21	-0.31	**-0.88**	0.07	**-0.71**	0.05	0.33
**Iv_jl_ind**	-0.17	-0.36	0.43	0.00	-0.33	0.05	-0.43	-0.36	-0.19	0.29	0.17	-0.19
**Iv_au_ind**	0.07	0.02	-0.05	0.55	0.33	0.12	**-0.88**	-0.21	0.26	-0.45	-0.02	**0.79**
**Iv-Vys_jn**	0.05	-0.12	0.57	0.36	-0.21	-0.10	-0.12	-0.69	0.19	-0.50	0.10	0.12
**Iv-Vys_jl**	-0.17	-0.36	0.43	0.00	-0.33	0.05	-0.43	-0.36	-0.19	0.29	0.17	-0.19
**Iv-Vys_aug**	0.14	0.12	-0.07	0.55	0.33	0.14	**-0.79**	-0.07	0.29	-0.31	-0.07	**0.79**
**Hum_may**	0.19	0.12	-0.05	0.33	0.12	0.43	-0.14	-0.10	0.33	-0.48	-0.69	-0.29
**Hum_jn**	-0.07	-0.17	0.29	0.19	-0.29	-0.33	-0.05	-0.17	-0.10	0.19	0.02	0.10
**Hum_jl**	0.10	-0.14	0.60	0.24	-0.26	0.36	-0.38	-0.45	0.12	0.05	-0.05	-0.29
**Hum_aug**	-0.16	-0.23	-0.08	0.30	-0.04	0.16	**-0.75**	-0.47	0.11	**-0.73**	-0.55	0.32
**T_may**	-0.26	-0.31	0.17	-0.05	-0.38	-0.48	-0.14	-0.10	-0.33	0.40	0.17	0.21
**T_jn**	0.60	0.60	0.36	0.31	0.24	0.55	-0.02	0.14	0.50	0.19	0.26	0.38
**T_jl**	0.07	0.19	-0.36	0.21	0.36	-0.14	0.02	0.12	0.19	-0.48	-0.12	0.43
**T_aug**	-0.10	-0.17	0.24	0.07	-0.40	-0.29	0.17	-0.21	-0.10	0.02	-0.24	-0.12
**Prec_jn**	0.21	0.05	0.57	0.52	-0.10	0.07	-0.19	-0.60	0.36	-0.50	0.00	0.21
**Prec_jl**	-0.31	-0.38	0.17	-0.31	-0.31	-0.14	-0.21	-0.17	-0.38	0.38	0.31	-0.14
**Prec_aug**	0.14	0.12	-0.07	0.55	0.33	0.14	**-0.79**	-0.07	0.29	-0.31	-0.07	**0.79**
**Wind_my**	-0.14	-0.12	-0.21	0.19	0.31	-0.24	-0.52	-0.02	-0.05	-0.17	0.31	0.60
**Wind_jn**	-0.25	-0.35	0.11	0.01	-0.10	-0.36	0.01	-0.28	-0.19	-0.12	0.17	-0.24
**Wind_jl**	**-0.75†**	**-0.81**	-0.22	-0.55	-0.62	-0.55	0.07	-0.19	**-0.74**	0.16	-0.40	**-0.75**
**Wind_aug**	-0.57	-0.52	-0.55	-0.43	-0.17	-0.55	0.19	0.26	-0.60	0.24	-0.29	-0.57

* as in Table S1

** as in Table S1

† as in Table S1

Table S3. A Spearman rho correlation matrix for Spruce #2 (continued 1).

Weather par	orgP	alcP	hclP	orgS	alcS	hclS	orgTi	alcTi	hclTi	orgMn	alcMn	hclMn
**Iv_jn_ind**	-0.52	0.33	0.38	**-0.74**	-0.36	0.22	-0.48	-0.36	0.50	-0.29	-0.29	-0.31
**Iv_jl_ind**	0.45	-0.31	-0.38	-0.43	0.36	-0.14	**-0.76**	0.17	**0.74**	**-0.71**	-0.55	-0.10
**Iv_au_ind**	-0.31	0.26	-0.14	-0.40	-0.14	0.33	-0.14	0.50	-0.05	0.19	0.29	-0.17
**Iv-Vys_jn**	-0.43	0.29	0.21	-0.69	-0.45	0.14	-0.26	-0.40	0.45	-0.12	-0.19	-0.10
**Iv-Vys_jl**	0.45	-0.31	-0.38	-0.43	0.36	-0.14	**-0.76**	0.17	**0.74**	**-0.71**	-0.55	-0.10
**Iv-Vys_aug**	-0.29	0.17	-0.19	-0.29	-0.24	0.25	0.05	0.67	-0.14	0.29	0.33	-0.02
**Hum_may**	**-0.71**	-0.64	-0.33	0.05	**-0.79**	-0.65	0.17	0.10	0.05	-0.10	-0.07	0.19
**Hum_jn**	0.07	0.21	0.14	-0.38	-0.26	-0.03	0.14	0.31	0.12	0.10	-0.17	-0.05
**Hum_jl**	0.05	-0.62	-0.57	-0.45	-0.14	-0.38	-0.64	0.10	**0.83**	**-0.71**	-0.52	0.19
**Hum_aug**	-0.66	-0.34	-0.11	-0.26	-0.42	-0.27	-0.35	0.36	0.20	-0.35	-0.19	-0.26
**T_may**	0.40	0.26	0.19	-0.29	0.17	0.11	-0.05	0.48	0.12	-0.05	-0.24	-0.19
**T_jn**	-0.02	-0.31	-0.48	0.07	-0.24	0.14	0.14	0.29	0.10	0.14	0.31	**0.74**
**T_jl**	-0.50	0.55	0.40	0.07	-0.33	0.30	0.55	-0.07	-0.62	0.69	0.52	-0.14
**T_aug**	-0.19	-0.05	0.21	-0.21	-0.50	-0.30	0.21	0.17	0.12	-0.02	-0.29	0.00
**Prec_jn**	-0.55	0.14	0.05	-0.64	-0.67	0.05	-0.10	-0.19	0.38	0.00	-0.07	0.10
**Prec_jl**	0.64	-0.07	-0.12	-0.17	**0.71**	0.08	-0.69	0.02	0.48	-0.60	-0.43	-0.24
**Prec_aug**	-0.29	0.17	-0.19	-0.29	-0.24	0.25	0.05	0.67	-0.14	0.29	0.33	-0.02
**Wind_my**	0.14	**0.74**	0.19	-0.29	0.40	0.63	-0.12	0.12	-0.24	0.33	0.33	-0.43
**Wind_jn**	0.11	0.53	0.30	-0.42	0.18	0.18	-0.22	-0.49	0.14	-0.01	-0.16	-0.46
**Wind_jl**	0.30	-0.20	0.28	-0.01	0.35	-0.55	-0.41	-0.08	0.26	-0.66	**-0.78**	-0.68
**Wind_aug**	0.33	0.19	0.33	0.21	0.38	-0.27	0.12	-0.05	-0.31	0.00	-0.24	-0.62

Table S3. A Spearman rho correlation matrix for Spruce #2 (continued 2).

Weather par	orgFe	alcFe	hclFe	orgCu	alcCu	hclCu	orgZn	alcZn	hclZn
**Iv_jn_ind**	0.14	0.24	-0.02	0.05	-0.38	-0.52	-0.36	0.07	-0.40
**Iv_jl_ind**	0.05	-0.14	0.60	0.50	**-0.81**	0.05	-0.31	0.14	-0.19
**Iv_au_ind**	-0.50	-0.14	0.60	0.31	-0.05	-0.52	**-0.88**	0.26	-0.69
**Iv-Vys_jn**	0.14	0.38	-0.10	-0.24	-0.21	-0.60	-0.17	0.10	-0.29
**Iv-Vys_jl**	0.05	-0.14	0.60	0.50	**-0.81**	0.05	-0.31	0.14	-0.19
**Iv-Vys_aug**	-0.50	-0.12	0.57	0.19	0.07	-0.43	**-0.86**	0.36	-0.55
**Hum_may**	0.50	0.48	0.36	-0.52	0.33	-0.43	-0.14	0.52	0.38
**Hum_jn**	-0.38	-0.10	-0.07	-0.07	-0.05	-0.19	-0.26	0.00	-0.05
**Hum_jl**	0.45	0.31	0.69	0.05	-0.64	-0.19	-0.29	0.52	0.00
**Hum_aug**	0.14	0.04	0.66	0.23	-0.14	-0.40	**-0.74**	0.43	-0.17
**T_may**	-0.55	-0.43	0.00	0.36	-0.29	0.14	-0.33	-0.14	-0.14
**T_jn**	0.33	0.48	0.29	-0.36	0.00	0.10	-0.10	0.69	-0.02
**T_jl**	-0.36	0.00	-0.38	-0.26	0.64	-0.36	-0.07	-0.19	-0.19
**T_aug**	0.00	0.10	-0.19	-0.29	0.07	-0.12	-0.07	0.10	0.29
**Prec_jn**	0.17	0.50	0.05	-0.40	-0.07	-0.67	-0.29	0.33	-0.24
**Prec_jl**	-0.07	-0.38	0.29	0.69	**-0.74**	0.36	-0.07	-0.19	-0.19
**Prec_aug**	-0.50	-0.12	0.57	0.19	0.07	-0.43	**-0.86**	0.36	-0.55
**Wind_my**	**-0.81**	-0.48	0.10	0.52	-0.05	-0.29	-0.45	-0.38	**-0.76**
**Wind_jn**	-0.34	-0.17	-0.28	0.13	-0.13	-0.37	0.11	-0.55	-0.29
**Wind_jl**	-0.06	-0.54	-0.06	0.51	-0.36	0.25	0.13	-0.54	0.37
**Wind_aug**	-0.50	-0.64	-0.31	0.33	0.17	0.14	0.24	**-0.76**	0.24

Table S4. A Kendall tau correlation matrix for Spruce #2.

Weather par**	orgCa*	alcCa	hclCa	orgSr	alcSr	hclSr	orgK	alcK	hclK	orgAl	alcAl	hclAl
**Iv_jn_ind**	-0.07	-0.21	0.36	0.21	-0.29	-0.14	-0.14	**-0.71**	0.07	**-0.57**	0.07	0.21
**Iv_jl_ind**	-0.14	-0.29	0.43	0.14	-0.21	0.07	-0.36	-0.21	-0.14	0.21	0.14	-0.14
**Iv_au_ind**	0.07	0.07	-0.07	0.36	0.29	0.14	**-0.71**	0.00	0.21	-0.29	-0.07	**0.64**
**Iv-Vys_jn**	0.00	-0.14	0.43	0.29	-0.21	-0.07	0.07	-0.50	0.14	-0.36	0.00	0.14
**Iv-Vys_jl**	-0.14	-0.29	0.43	0.14	-0.21	0.07	-0.36	-0.21	-0.14	0.21	0.14	-0.14
**Iv-Vys_aug**	0.21	0.21	-0.07	0.36	0.29	0.14	**-0.57**	0.00	0.21	-0.29	-0.07	**0.64**
**Hum_may**	0.07	0.07	-0.07	0.21	0.00	0.29	-0.14	-0.14	0.21	-0.29	-0.50	-0.21
**Hum_jn**	-0.07	-0.07	0.21	0.07	-0.29	-0.29	0.00	-0.14	-0.07	0.14	0.07	0.07
**Hum_jl**	0.07	-0.07	0.50	0.21	-0.14	0.29	-0.29	-0.29	0.07	0.00	-0.07	-0.21
**Hum_aug**	-0.11	-0.18	-0.11	0.18	-0.11	0.11	-0.55	-0.40	0.04	**-0.62**	-0.47	0.25
**T_may**	-0.21	-0.21	0.07	-0.07	-0.43	-0.43	-0.14	-0.14	-0.21	0.43	0.07	0.21
**T_jn**	0.43	0.43	0.29	0.14	0.07	0.36	-0.07	0.07	0.29	0.21	0.14	0.29
**T_jl**	0.00	0.14	-0.29	0.00	0.21	-0.21	0.07	0.07	0.00	-0.36	-0.14	0.29
**T_aug**	-0.07	-0.07	0.21	0.07	-0.29	-0.29	0.14	-0.14	-0.07	0.00	-0.07	-0.07
**Prec_jn**	0.14	0.00	0.43	0.43	-0.07	0.07	-0.07	-0.36	0.29	-0.36	0.00	0.29
**Prec_jl**	-0.29	-0.29	0.14	-0.14	-0.21	-0.07	-0.21	-0.07	-0.29	0.21	0.43	-0.14
**Prec_aug**	0.21	0.21	-0.07	0.36	0.29	0.14	**-0.57**	0.00	0.21	-0.29	-0.07	**0.64**
**Wind_my**	-0.14	-0.14	-0.14	0.14	0.21	-0.21	-0.36	-0.07	-0.14	-0.07	0.14	0.43
**Wind_jn**	-0.11	-0.25	0.04	0.04	-0.04	-0.18	-0.04	-0.18	0.04	-0.04	0.04	-0.11
**Wind_jl**	-0.55	**-0.69†**	-0.18	-0.40	-0.47	-0.33	0.04	-0.04	-0.55	0.18	-0.33	-0.55
**Wind_aug**	-0.43	-0.29	-0.43	-0.29	-0.07	-0.36	0.21	0.21	-0.43	0.07	-0.14	-0.43

* as in Table S1

** as in Table S1

† as in Table S1

Table S4. A Kendall tau correlation matrix for Spruce #2 (continued 1).

Weather par	orgP	alcP	hclP	orgS	alcS	hclS	orgTi	alcTi	hclTi	orgMn	alcMn	hclMn
**Iv_jn_ind**	-0.43	0.29	0.29	-0.50	-0.07	0.18	-0.36	-0.21	0.36	-0.21	-0.21	-0.14
**Iv_jl_ind**	0.36	-0.21	-0.21	-0.29	0.29	-0.09	**-0.57**	0.14	**0.57**	**-0.57**	-0.43	-0.07
**Iv_au_ind**	-0.14	0.14	-0.14	-0.36	-0.21	0.27	-0.07	0.36	-0.07	0.21	0.21	-0.14
**Iv-Vys_jn**	-0.21	0.21	0.21	-0.43	-0.29	0.09	-0.14	-0.29	0.29	-0.14	-0.29	-0.07
**Iv-Vys_jl**	0.36	-0.21	-0.21	-0.29	0.29	-0.09	**-0.57**	0.14	**0.57**	**-0.57**	-0.43	-0.07
**Iv-Vys_aug**	-0.14	0.14	-0.29	-0.21	-0.21	0.18	0.07	0.50	-0.07	0.21	0.21	0.00
**Hum_may**	**-0.57**	-0.43	-0.29	0.07	**-0.64**	-0.53	0.07	0.07	0.07	-0.07	-0.07	0.14
**Hum_jn**	0.00	0.14	0.14	-0.36	-0.21	0.00	0.07	0.21	0.07	0.07	-0.07	0.00
**Hum_jl**	0.00	-0.43	-0.43	-0.36	-0.07	-0.36	-0.50	0.07	**0.64**	-0.50	-0.36	0.14
**Hum_aug**	-0.55	-0.25	-0.11	-0.18	-0.33	-0.23	-0.33	0.33	0.18	-0.33	-0.18	-0.18
**T_may**	0.29	0.14	0.14	-0.21	0.07	0.00	-0.07	0.36	0.07	-0.07	-0.21	-0.14
**T_jn**	-0.07	-0.21	-0.36	0.00	-0.14	0.09	-0.14	0.29	0.14	0.14	0.29	**0.64**
**T_jl**	-0.36	0.36	0.36	0.00	-0.29	0.27	0.29	0.00	-0.43	**0.57**	0.43	-0.07
**T_aug**	0.00	0.00	0.14	-0.21	-0.36	-0.27	0.21	0.07	0.07	-0.07	-0.21	0.00
**Prec_jn**	-0.21	0.07	0.07	-0.43	-0.43	0.00	0.00	-0.14	0.29	0.00	-0.14	0.07
**Prec_jl**	0.50	-0.07	-0.07	-0.14	**0.57**	0.09	-0.43	0.00	0.29	-0.43	-0.29	-0.21
**Prec_aug**	-0.14	0.14	-0.29	-0.21	-0.21	0.18	0.07	0.50	-0.07	0.21	0.21	0.00
**Wind_my**	0.07	**0.64**	0.07	-0.14	0.29	0.45	-0.14	0.00	-0.14	0.14	0.14	-0.36
**Wind_jn**	0.11	0.40	0.18	-0.33	0.11	0.14	-0.11	-0.33	0.11	0.04	-0.11	-0.33
**Wind_jl**	0.33	-0.18	0.18	0.04	0.25	-0.45	-0.25	-0.11	0.11	-0.55	**-0.69**	-0.55
**Wind_aug**	0.21	0.21	0.21	0.14	0.29	-0.18	0.14	0.00	-0.14	0.00	-0.14	-0.36

Table S4. A Kendall tau correlation matrix for Spruce #2 (continued 2).

Weather par	orgFe	alcFe	hclFe	orgCu	alcCu	hclCu	orgZn	alcZn	hclZn
**Iv_jn_ind**	0.14	0.21	0.00	0.07	-0.21	-0.29	-0.29	0.07	-0.21
**Iv_jl_ind**	0.07	-0.14	0.50	0.43	**-0.57**	-0.07	-0.21	0.14	-0.14
**Iv_au_ind**	-0.43	-0.07	0.43	0.21	-0.07	-0.43	**-0.71**	0.21	-0.50
**Iv-Vys_jn**	0.07	0.29	-0.07	-0.14	0.00	-0.36	-0.07	0.14	-0.14
**Iv-Vys_jl**	0.07	-0.14	0.50	0.43	**-0.57**	-0.07	-0.21	0.14	-0.14
**Iv-Vys_aug**	-0.43	-0.07	0.43	0.07	0.07	-0.29	**-0.71**	0.21	-0.36
**Hum_may**	0.43	0.21	0.29	-0.36	0.21	-0.29	-0.14	0.36	0.21
**Hum_jn**	-0.29	-0.07	-0.14	-0.07	-0.07	-0.14	-0.14	-0.07	-0.07
**Hum_jl**	0.29	0.21	**0.57**	0.07	-0.50	-0.14	-0.29	0.36	0.07
**Hum_aug**	0.11	0.04	0.55	0.11	-0.18	-0.25	-0.55	0.33	-0.11
**T_may**	-0.43	-0.36	0.00	0.21	-0.21	0.14	-0.29	-0.21	-0.21
**T_jn**	0.36	0.29	0.21	-0.29	-0.14	0.07	-0.07	0.43	0.00
**T_jl**	-0.21	0.00	-0.36	-0.29	0.43	-0.21	0.07	-0.14	-0.14
**T_aug**	0.00	0.07	-0.14	-0.21	0.07	-0.14	0.00	0.07	0.21
**Prec_jn**	0.07	0.43	0.07	-0.29	0.00	-0.50	-0.21	0.29	-0.14
**Prec_jl**	-0.07	-0.29	0.21	**0.57**	**-0.57**	0.21	-0.07	-0.14	-0.14
**Prec_aug**	-0.43	-0.07	0.43	0.07	0.07	-0.29	**-0.71**	0.21	-0.36
**Wind_my**	**-0.64**	-0.43	0.07	0.43	0.00	-0.21	-0.36	-0.29	**-0.57**
**Wind_jn**	-0.25	-0.11	-0.11	0.11	-0.11	-0.25	-0.04	-0.40	-0.18
**Wind_jl**	-0.11	-0.33	-0.04	0.40	-0.25	0.18	0.04	-0.33	0.40
**Wind_aug**	-0.36	**-0.57**	-0.21	0.29	0.14	0.07	0.21	**-0.57**	0.14

Table S5. A Spearman rho correlation matrix for Spruce #3.

Weather par	orgCa*	alcCa	hclCa	orgSr	alcSr	hclSr	orgK	alcK	hclK	orgAl	alcAl	hclAl
**Iv_jn_ind****	0.20	0.18	-0.36	0.02	-0.14	0.19	-0.16	-0.19	0.08	-0.07	0.20	-0.36
**Iv_jl_ind**	0.07	-0.08	-0.13	-0.45	-0.21	-0.12	-0.59	-0.38	0.06	0.01	0.49	0.52
**Iv_au_ind**	-0.38	-0.34	-0.31	-0.27	-0.26	-0.42	-0.02	0.27	-0.01	-0.15	-0.25	-0.12
**Iv-Vys_jn**	0.26	0.28	-0.23	-0.04	-0.09	0.31	-0.06	-0.19	-0.02	-0.03	0.11	-0.27
**Iv-Vys_jl**	0.07	-0.09	-0.13	-0.40	-0.14	-0.12	**-0.55**	-0.35	-0.08	-0.01	0.45	0.43
**Iv-Vys_aug**	-0.34	-0.31	-0.41	-0.30	-0.46	-0.33	-0.18	0.09	-0.07	-0.25	-0.21	-0.04
**Hum_may**	0.25	0.17	-0.21	0.42	-0.24	0.13	-0.13	-0.09	-0.28	-0.13	0.07	-0.42
**Hum_jn**	-0.04	0.08	0.07	-0.53	-0.17	0.12	0.07	-0.14	0.47	0.18	0.10	0.24
**Hum_jl**	0.09	0.03	0.03	-0.01	-0.13	0.50	-0.54	**-0.79**	0.08	0.24	0.37	0.38
**Hum_aug**	0.04	-0.05	**-0.67†**	-0.10	-0.52	-0.20	-0.47	-0.14	-0.32	-0.37	0.23	-0.45
**T_may**	-0.51	-0.41	0.35	-0.53	0.13	0.12	0.15	-0.19	**0.61**	0.45	-0.16	**0.70**
**T_jn**	-0.14	-0.03	-0.19	-0.01	-0.27	0.48	-0.45	**-0.57**	0.15	0.08	-0.06	0.37
**T_jl**	-0.16	-0.14	0.18	0.36	0.02	0.02	0.43	0.16	-0.12	-0.15	-0.51	-0.20
**T_aug**	-0.29	-0.16	0.21	0.09	0.47	0.35	**0.65**	0.29	0.19	0.44	**-0.69**	0.11
**Prec_jn**	0.24	0.24	-0.18	0.12	-0.10	0.37	-0.05	-0.23	-0.09	-0.03	0.03	-0.29
**Prec_jl**	-0.02	-0.18	-0.16	-0.53	-0.12	-0.19	-0.53	-0.30	0.03	-0.04	0.42	**0.55**
**Prec_aug**	-0.43	-0.41	-0.21	-0.11	-0.23	-0.23	0.02	0.13	0.04	-0.07	-0.38	0.04
**Wind_my**	-0.50	-0.45	0.20	-0.36	0.13	-0.43	0.24	0.21	0.17	-0.02	-0.24	0.22
**Wind_jn**	-0.07	-0.01	0.23	-0.30	0.34	-0.24	0.47	0.42	0.15	0.15	-0.10	-0.06
**Wind_jl**	-0.18	-0.37	0.16	-0.23	0.33	-0.15	0.08	0.02	-0.18	0.14	0.01	0.32
**Wind_aug**	-0.09	-0.23	0.44	-0.09	0.35	-0.43	0.46	0.37	-0.07	0.06	-0.03	0.07

* as in Table S1

** as in Table S1

† as in Table S1

Table S5. A Spearman rho correlation matrix for Spruce #3 (continued 1).

Weather par	orgP	alcP	hclP	orgS	alcS	hclS	orgTi	alcTi	hclTi	orgMn	alcMn	hclMn
**Iv_jn_ind**	-0.13	0.20	0.29	-0.37	-0.08	**-0.56**	-0.19	0.17	-0.26	0.05	-0.02	0.14
**Iv_jl_ind**	-0.05	-0.01	-0.25	-0.11	0.35	0.17	-0.15	0.15	0.36	-0.03	-0.04	-0.07
**Iv_au_ind**	0.42	0.00	0.14	-0.10	-0.08	-0.02	**0.58**	0.24	-0.05	0.48	0.38	0.33
**Iv-Vys_jn**	-0.26	0.15	0.18	-0.19	-0.03	**-0.63**	-0.19	0.11	-0.29	0.01	-0.03	-0.02
**Iv-Vys_jl**	-0.07	0.05	-0.26	-0.02	0.28	0.22	-0.12	0.24	0.30	0.02	-0.04	-0.02
**Iv-Vys_aug**	0.32	-0.09	-0.06	0.04	-0.09	-0.07	0.41	0.28	-0.05	0.29	0.26	0.21
**Hum_may**	-0.29	-0.01	-0.34	0.22	-0.10	-0.42	0.02	0.33	-0.19	0.10	0.14	0.00
**Hum_jn**	0.05	-0.20	-0.13	-0.04	-0.01	**-0.62**	-0.24	-0.25	0.33	-0.23	-0.03	-0.19
**Hum_jl**	**-0.64**	-0.15	**-0.60**	-0.03	0.31	-0.19	-0.27	0.28	0.24	-0.42	-0.36	**-0.58**
**Hum_aug**	0.08	0.22	-0.18	0.18	-0.26	-0.21	-0.01	**0.62**	-0.13	0.29	0.20	0.39
**T_may**	0.16	-0.52	-0.07	-0.18	0.06	-0.20	0.01	-0.40	0.48	-0.49	-0.36	-0.40
**T_jn**	-0.42	-0.11	-0.28	-0.07	0.36	0.19	0.00	0.06	0.08	-0.27	-0.20	-0.54
**T_jl**	0.00	-0.25	0.01	-0.12	-0.17	-0.44	0.24	0.10	-0.46	-0.19	-0.19	-0.08
**T_aug**	0.08	-0.29	0.00	-0.02	0.06	-0.52	0.45	-0.29	0.26	0.01	0.03	-0.18
**Prec_jn**	-0.37	0.06	0.14	-0.19	-0.03	**-0.63**	-0.10	0.14	-0.42	-0.04	-0.08	-0.12
**Prec_jl**	0.13	0.03	-0.13	-0.08	0.24	0.35	-0.23	0.09	0.38	-0.10	-0.15	0.06
**Prec_aug**	0.29	-0.21	-0.06	-0.10	0.00	-0.13	0.56	0.19	0.01	0.21	0.19	0.09
**Wind_my**	0.49	-0.07	0.14	-0.26	-0.09	0.15	0.28	0.07	0.10	0.01	-0.09	0.18
**Wind_jn**	0.36	0.11	0.19	-0.12	-0.07	-0.30	0.16	-0.11	0.22	0.28	0.21	0.28
**Wind_jl**	0.23	-0.16	-0.23	0.20	-0.13	0.02	-0.08	0.12	0.37	-0.20	-0.30	0.14
**Wind_aug**	0.39	-0.17	-0.16	0.13	-0.25	-0.19	0.00	0.00	0.24	-0.07	-0.09	0.27

Table S5. A Spearman rho correlation matrix for Spruce #3 (continued 2).

Weather par	orgFe	alcFe	hclFe	orgCu	alcCu	hclCu	orgZn	alcZn	hclZn
**Iv_jn_ind**	0.05	0.03	-0.09	-0.09	0.07	-0.25	-0.18	-0.19	-0.12
**Iv_jl_ind**	-0.48	0.08	0.09	0.40	0.20	0.26	**0.56**	**0.68**	0.50
**Iv_au_ind**	-0.09	0.05	**0.66**	0.16	-0.50	0.08	0.27	-0.23	-0.47
**Iv-Vys_jn**	0.03	0.01	-0.27	-0.23	0.19	-0.35	-0.25	-0.10	-0.03
**Iv-Vys_jl**	-0.49	0.20	0.03	0.42	0.21	0.34	**0.58**	**0.63**	0.44
**Iv-Vys_aug**	-0.10	-0.02	0.52	0.07	-0.35	0.00	0.26	-0.16	-0.38
**Hum_may**	0.02	0.38	0.19	-0.03	-0.24	0.05	0.09	-0.16	-0.09
**Hum_jn**	0.07	-0.27	-0.29	-0.18	0.48	-0.38	-0.13	0.13	0.25
**Hum_jl**	-0.50	0.36	-0.23	-0.18	0.36	-0.24	0.04	0.47	**0.74**
**Hum_aug**	-0.12	0.41	0.18	0.34	-0.06	0.34	0.48	-0.13	-0.29
**T_may**	-0.09	-0.36	-0.15	-0.30	0.33	-0.53	-0.24	0.23	0.34
**T_jn**	-0.19	0.01	0.03	-0.34	0.01	-0.44	-0.11	0.18	0.32
**T_jl**	0.14	-0.31	0.09	**-0.69**	-0.29	**-0.56**	**-0.75**	**-0.63**	-0.40
**T_aug**	**0.56**	0.08	0.18	-0.28	-0.39	-0.41	-0.14	-0.29	-0.37
**Prec_jn**	-0.05	0.02	-0.19	-0.37	0.08	-0.46	-0.37	-0.15	-0.03
**Prec_jl**	-0.33	-0.03	-0.07	0.46	0.30	0.34	**0.57**	**0.62**	0.36
**Prec_aug**	-0.02	-0.01	**0.66**	-0.08	**-0.55**	-0.16	0.10	-0.26	-0.40
**Wind_my**	-0.12	-0.26	0.12	-0.07	-0.01	-0.06	-0.16	-0.22	-0.19
**Wind_jn**	0.20	-0.01	-0.01	0.17	0.04	0.13	0.07	-0.12	-0.19
**Wind_jl**	-0.03	0.21	-0.25	0.30	0.24	0.35	0.38	0.23	0.03
**Wind_aug**	0.07	-0.01	-0.10	0.17	0.13	0.31	0.07	-0.07	-0.08

Table S6. A Kendall tau correlation matrix for Spruce #3.

Weather par	orgCa*	alcCa	hclCa	orgSr	alcSr	hclSr	orgK	alcK	hclK	orgAl	alcAl	hclAl
**Iv_jn_ind****	0.15	0.15	-0.26	0.08	-0.08	0.18	-0.13	-0.13	0.10	-0.03	0.13	-0.21
**Iv_jl_ind**	0.00	-0.10	-0.10	-0.28	-0.18	-0.08	**-0.44**	-0.28	0.00	0.03	0.38	0.36
**Iv_au_ind**	-0.31	-0.26	-0.26	-0.13	-0.18	-0.28	0.03	0.18	0.00	-0.13	-0.18	-0.05
**Iv-Vys_jn**	0.21	0.21	-0.15	-0.03	-0.08	0.23	-0.08	-0.08	0.00	-0.03	0.08	-0.21
**Iv-Vys_jl**	0.03	-0.08	-0.08	-0.26	-0.15	-0.10	-0.41	-0.21	-0.08	0.00	0.36	0.33
**Iv-Vys_aug**	-0.23	-0.23	-0.28	-0.26	-0.31	-0.26	-0.10	0.05	-0.08	-0.21	-0.15	0.03
**Hum_may**	0.23	0.13	-0.18	0.26	-0.15	0.05	-0.10	-0.10	-0.23	-0.05	0.05	-0.33
**Hum_jn**	-0.05	0.05	0.05	-0.38	-0.18	0.08	0.13	-0.08	0.31	0.13	0.03	0.15
**Hum_jl**	0.08	-0.03	0.03	0.05	-0.05	0.41	-0.41	**-0.67**	0.08	0.15	0.31	0.28
**Hum_aug**	0.06	0.01	**-0.50**†	-0.06	-0.35	-0.09	-0.35	-0.09	-0.22	-0.25	0.14	-0.32
**T_may**	-0.38	-0.28	0.23	-0.41	0.10	0.10	0.15	-0.15	**0.49**	0.26	-0.15	**0.54**
**T_jn**	-0.08	-0.08	-0.18	0.00	-0.15	0.36	-0.31	-0.36	0.08	0.05	-0.10	0.23
**T_jl**	-0.10	-0.10	0.15	0.28	0.03	0.08	0.28	0.08	-0.10	-0.08	-0.38	-0.15
**T_aug**	-0.23	-0.08	0.13	0.00	0.31	0.21	**0.51**	0.26	0.18	0.31	**-0.51**	0.03
**Prec_jn**	0.18	0.23	-0.13	0.05	-0.10	0.26	-0.05	-0.15	-0.03	-0.05	0.05	-0.18
**Prec_jl**	-0.03	-0.13	-0.08	-0.36	-0.10	-0.15	-0.36	-0.21	-0.03	0.00	0.31	**0.44**
**Prec_aug**	-0.36	-0.31	-0.15	-0.13	-0.18	-0.18	0.03	0.08	0.05	-0.08	-0.28	0.05
**Wind_my**	-0.37	-0.43	0.14	-0.25	0.09	-0.37	0.14	0.14	0.12	-0.06	-0.14	0.17
**Wind_jn**	-0.09	-0.04	0.22	-0.19	0.32	-0.14	0.35	0.30	0.12	0.06	-0.04	-0.04
**Wind_jl**	-0.07	-0.25	0.14	-0.17	0.27	-0.09	0.01	0.01	-0.17	0.12	0.01	0.25
**Wind_aug**	-0.04	-0.17	0.33	-0.07	0.25	-0.33	0.33	0.33	-0.04	0.01	-0.04	0.04

* as in Table S1

** as in Table S1

† as in Table S1

Table S6. A Kendall tau correlation matrix for Spruce #3 (continued 1).

Weather par	orgP	alcP	hclP	orgS	alcS	hclS	orgTi	alcTi	hclTi	orgMn	alcMn	hclMn
**Iv_jn_ind**	-0.13	0.13	0.16	-0.26	-0.09	**-0.45**	-0.13	0.15	-0.15	0.03	-0.03	0.10
**Iv_jl_ind**	0.03	0.03	-0.24	-0.10	0.32	0.12	-0.08	0.10	0.26	-0.03	-0.08	-0.05
**Iv_au_ind**	0.28	0.03	0.08	-0.05	-0.09	-0.02	0.38	0.15	0.00	0.33	0.28	0.26
**Iv-Vys_jn**	-0.23	0.13	0.11	-0.21	-0.04	**-0.54**	-0.13	0.10	-0.15	-0.03	-0.03	0.00
**Iv-Vys_jl**	0.05	0.05	-0.21	-0.03	0.30	0.17	-0.05	0.18	0.18	0.00	-0.05	-0.03
**Iv-Vys_aug**	0.21	-0.05	0.00	0.08	-0.06	-0.07	0.26	0.18	-0.03	0.21	0.21	0.13
**Hum_may**	-0.15	0.00	-0.32	0.13	-0.12	-0.35	-0.05	0.18	-0.18	0.05	0.10	0.03
**Hum_jn**	-0.03	-0.13	-0.16	-0.05	-0.01	**-0.50**	-0.18	-0.15	0.26	-0.18	-0.08	-0.10
**Hum_jl**	**-0.46**	-0.10	**-0.43**	0.03	0.25	-0.17	-0.21	0.23	0.18	-0.26	-0.26	-0.38
**Hum_aug**	0.06	0.17	-0.13	0.19	-0.21	-0.17	-0.04	**0.43**	-0.06	0.22	0.12	0.27
**T_may**	0.10	-0.31	-0.11	-0.13	0.01	-0.17	-0.05	-0.33	0.38	-0.31	-0.21	-0.23
**T_jn**	-0.26	-0.05	-0.24	-0.13	0.19	0.17	0.00	0.03	0.03	-0.10	-0.05	**-0.44**
**T_jl**	-0.03	-0.23	0.00	-0.05	-0.19	-0.35	0.08	0.05	-0.31	-0.18	-0.13	-0.05
**T_aug**	0.05	-0.15	0.00	-0.03	-0.04	**-0.45**	0.36	-0.18	0.18	0.00	0.00	-0.13
**Prec_jn**	-0.31	0.10	0.05	-0.18	-0.01	**-0.54**	-0.10	0.08	-0.23	-0.05	-0.05	-0.08
**Prec_jl**	0.10	0.00	-0.11	-0.08	0.22	0.31	-0.10	0.03	0.28	-0.05	-0.10	0.03
**Prec_aug**	0.18	-0.13	-0.03	-0.05	0.01	-0.12	0.33	0.05	0.00	0.13	0.13	0.05
**Wind_my**	0.35	-0.06	0.16	-0.19	-0.08	0.12	0.19	0.09	0.06	-0.01	-0.06	0.14
**Wind_jn**	0.27	0.04	0.17	-0.01	0.00	-0.26	0.14	-0.06	0.19	0.19	0.14	0.19
**Wind_jl**	0.14	-0.12	-0.15	0.17	-0.11	0.00	-0.01	0.14	0.25	-0.17	-0.22	0.07
**Wind_aug**	0.33	-0.12	-0.08	0.07	-0.18	-0.17	0.01	0.01	0.17	-0.12	-0.12	0.20

Table S6. A Kendall tau correlation matrix for Spruce #3 (continued 2).

Weather par	orgFe	alcFe	hclFe	orgCu	alcCu	hclCu	orgZn	alcZn	hclZn
**Iv_jn_ind**	0.05	0.00	-0.08	-0.10	0.05	-0.21	-0.18	-0.18	0.00
**Iv_jl_ind**	-0.31	0.00	0.08	0.31	0.15	0.21	0.38	**0.54**	0.36
**Iv_au_ind**	-0.10	0.05	**0.44**	0.10	-0.36	0.05	0.13	-0.13	-0.36
**Iv-Vys_jn**	0.00	0.00	-0.18	-0.15	0.10	-0.26	-0.18	-0.08	0.00
**Iv-Vys_jl**	-0.33	0.13	0.00	0.28	0.18	0.23	0.41	**0.46**	0.33
**Iv-Vys_aug**	-0.13	0.08	0.31	0.03	-0.28	-0.03	0.15	-0.10	-0.33
**Hum_may**	-0.03	0.23	0.10	-0.08	-0.13	0.08	0.05	-0.10	-0.08
**Hum_jn**	0.05	-0.21	-0.18	-0.15	0.31	-0.31	-0.13	0.13	0.21
**Hum_jl**	-0.38	0.28	-0.15	-0.08	0.28	-0.08	0.10	0.31	**0.54**
**Hum_aug**	-0.12	0.25	0.17	0.25	-0.04	0.27	0.37	-0.09	-0.25
**T_may**	-0.08	-0.23	-0.10	-0.23	0.23	-0.33	-0.15	0.21	0.28
**T_jn**	-0.13	0.03	0.00	-0.23	-0.03	-0.33	-0.10	0.10	0.23
**T_jl**	0.10	-0.26	-0.03	**-0.51**	-0.15	-0.41	**-0.59**	**-0.49**	-0.31
**T_aug**	0.38	0.08	0.15	-0.18	-0.33	-0.28	-0.10	-0.21	-0.23
**Prec_jn**	-0.03	0.03	-0.15	-0.23	0.03	-0.33	-0.26	-0.15	0.03
**Prec_jl**	-0.18	-0.03	-0.05	0.33	0.18	0.23	0.41	**0.46**	0.23
**Prec_aug**	-0.05	0.05	**0.44**	-0.10	-0.41	-0.15	0.03	-0.18	-0.31
**Wind_my**	-0.09	-0.09	0.09	-0.04	-0.01	-0.01	-0.12	-0.12	-0.12
**Wind_jn**	0.14	0.04	-0.01	0.14	-0.01	0.09	0.06	-0.06	-0.19
**Wind_jl**	-0.07	0.14	-0.22	0.22	0.14	0.22	0.27	0.17	-0.01
**Wind_aug**	0.07	0.04	-0.07	0.12	0.12	0.22	0.04	-0.07	-0.09

Table S7. A Spearman rho correlation matrix for Siberian pine #1.

Weather par	orgCa*	alcCa	hclCa	orgSr	alcSr	hclSr	orgK	alcK	hclK	orgAl	alcAl	hclAl
**Iv_jn_ind****	0.44	0.36	-0.16	0.01	0.22	**0.62**	-0.04	-0.07	-0.01	0.45	-0.34	0.34
**Iv_jl_ind**	-0.35	-0.40	0.24	-0.31	0.40	0.18	-0.01	0.19	-0.36	0.04	-0.25	0.40
**Iv_au_ind**	-0.40	-0.48	-0.02	0.34	-0.12	-0.08	0.09	0.12	-0.10	0.17	-0.32	0.46
**Iv-Vys_jn**	0.49	0.41	0.02	0.07	0.06	0.50	0.05	-0.19	0.07	0.20	-0.21	0.29
**Iv-Vys_jl**	-0.30	-0.35	0.20	-0.27	0.42	0.27	-0.03	0.10	-0.32	-0.01	-0.24	0.40
**Iv-Vys_aug**	-0.35	-0.42	0.24	0.18	-0.12	0.09	-0.10	-0.09	-0.09	0.09	-0.27	**0.58**
**Hum_may**	-0.26	-0.23	0.02	-0.25	-0.46	0.17	0.04	0.08	0.25	-0.15	0.22	-0.24
**Hum_jn**	**0.64**[†]	**0.58**	0.01	-0.01	-0.04	0.38	-0.20	-0.40	-0.07	0.20	0.00	0.10
**Hum_jl**	-0.25	-0.34	0.13	0.01	-0.07	0.53	0.06	-0.03	0.01	-0.01	-0.21	0.24
**Hum_aug**	-0.41	-0.53	0.44	-0.19	-0.07	0.34	0.01	-0.04	-0.07	-0.11	-0.04	0.50
**T_may**	0.14	0.15	0.28	0.12	0.02	-0.31	-0.24	-0.48	-0.35	-0.32	-0.03	0.25
**T_jn**	-0.12	-0.21	-0.07	-0.04	-0.10	-0.09	-0.31	-0.07	-0.32	0.26	0.05	0.19
**T_jl**	0.42	0.46	0.27	0.23	0.03	0.01	-0.06	-0.40	0.52	-0.31	0.14	0.13
**T_aug**	-0.05	0.08	-0.23	0.18	**-0.73**	-0.21	0.23	-0.03	0.16	-0.23	-0.03	-0.38
**Prec_jn**	0.45	0.36	0.06	0.19	0.10	0.53	0.05	-0.20	0.20	0.20	-0.27	0.37
**Prec_jl**	-0.32	-0.30	0.19	-0.25	**0.59**	0.15	0.00	0.20	-0.27	0.03	-0.23	0.29
**Prec_aug**	-0.19	-0.24	0.03	0.48	-0.26	0.02	0.02	-0.08	0.14	0.16	-0.31	0.40
**Wind_my**	0.26	0.32	0.06	0.21	0.40	-0.06	0.20	-0.08	0.19	-0.09	-0.03	0.18
**Wind_jn**	0.27	0.35	0.35	0.07	0.26	-0.01	0.18	-0.15	0.05	-0.15	-0.09	0.13
**Wind_jl**	-0.26	-0.17	0.13	-0.04	0.12	-0.17	0.09	0.21	-0.02	-0.17	-0.12	-0.29
**Wind_aug**	-0.02	0.11	0.10	0.18	0.23	-0.04	-0.14	0.02	0.45	-0.15	-0.27	-0.06

* as in Table S1

** as in Table S1

† as in Table S1

Table S7. A Spearman rho correlation matrix for Siberian pine #1 (continued 1).

Weather par	orgP	alcP	hclP	orgS	alcS	hclS	orgTi	alcTi	hclTi	orgMn	alcMn	hclMn
**Iv_jn_ind**	-0.03	0.24	0.06	-0.24	0.16	0.13	**0.60**	0.20	**0.65**	0.45	**0.60**	0.35
**Iv_jl_ind**	**-0.68**	-0.21	-0.29	-0.19	-0.12	-0.06	-0.24	-0.03	0.21	0.15	0.27	0.33
**Iv_au_ind**	-0.19	0.03	0.30	0.48	0.12	0.02	-0.48	-0.34	-0.18	-0.32	-0.48	0.15
**Iv-Vys_jn**	0.06	0.16	0.00	-0.21	0.29	0.04	0.51	0.34	0.54	0.34	0.54	0.35
**Iv-Vys_jl**	**-0.74**	-0.12	-0.21	-0.16	-0.03	-0.03	-0.16	-0.13	0.32	0.15	0.26	0.15
**Iv-Vys_aug**	-0.31	-0.10	0.11	0.43	0.17	-0.07	**-0.57**	-0.26	0.03	-0.14	-0.36	0.06
**Hum_may**	-0.09	0.05	-0.19	0.03	-0.03	-0.20	-0.16	0.13	0.28	-0.14	-0.19	-0.24
**Hum_jn**	0.32	0.09	-0.03	0.00	0.15	0.06	0.41	0.09	0.38	0.47	0.47	0.05
**Hum_jl**	-0.37	0.04	0.00	0.25	0.23	-0.37	-0.20	-0.27	0.47	0.14	0.15	-0.04
**Hum_aug**	**-0.75**	0.17	-0.05	0.12	0.37	-0.02	-0.34	-0.21	0.28	-0.05	-0.09	-0.12
**T_may**	0.06	-0.50	-0.09	0.33	-0.01	-0.03	-0.45	-0.18	-0.29	-0.06	-0.20	-0.10
**T_jn**	0.08	-0.03	0.14	0.43	-0.15	-0.06	-0.38	-0.52	-0.12	0.17	-0.18	-0.16
**T_jl**	0.25	-0.25	-0.04	-0.30	0.25	-0.42	0.07	**0.59**	0.37	0.15	0.13	-0.07
**T_aug**	0.46	-0.27	-0.16	0.38	-0.20	-0.04	-0.27	0.26	-0.14	**-0.57**	**-0.57**	0.05
**Prec_jn**	0.10	0.08	0.06	-0.19	0.34	-0.18	0.42	0.37	**0.60**	0.38	**0.55**	0.38
**Prec_jl**	**-0.66**	-0.23	-0.30	-0.25	-0.20	-0.08	-0.20	-0.07	0.21	0.18	0.25	0.22
**Prec_aug**	0.10	-0.03	0.30	0.55	0.21	-0.12	-0.43	-0.20	-0.07	-0.24	-0.47	0.09
**Wind_my**	-0.10	-0.05	-0.09	-0.31	0.04	-0.01	0.14	0.33	0.23	0.01	0.04	0.14
**Wind_jn**	-0.09	-0.11	-0.29	-0.28	0.13	0.24	0.18	0.45	-0.04	-0.04	0.16	0.34
**Wind_jl**	-0.06	-0.09	-0.05	-0.11	-0.01	0.18	0.06	0.04	-0.54	-0.22	0.04	0.11
**Wind_aug**	0.01	-0.10	0.20	-0.41	0.22	0.19	0.35	0.44	-0.20	-0.20	0.03	-0.01

Table S7. A Spearman rho correlation matrix for Siberian pine #1 (continued 2).

Weather par	orgFe	alcFe	hclFe	orgCu	alcCu	hclCu	orgZn	alcZn	hclZn
**Iv_jn_ind**	0.22	0.14	-0.04	-0.27	**-0.57**	**-0.60**	-0.23	-0.38	0.20
**Iv_jl_ind**	**-0.60**	-0.17	-0.46	0.11	-0.34	0.06	-0.45	-0.45	-0.17
**Iv_au_ind**	-0.35	0.25	-0.09	0.41	0.21	0.35	-0.49	-0.22	0.37
**Iv-Vys_jn**	0.09	0.06	0.01	-0.33	-0.43	**-0.60**	-0.19	-0.25	0.12
**Iv-Vys_jl**	**-0.58**	-0.33	-0.47	0.04	-0.46	0.01	-0.38	**-0.61**	-0.29
**Iv-Vys_aug**	-0.19	0.34	0.00	0.06	-0.08	0.15	-0.46	-0.41	0.14
**Hum_may**	0.20	0.02	0.10	-0.13	0.03	-0.13	0.26	-0.13	-0.28
**Hum_jn**	0.37	0.09	0.14	-0.44	-0.41	**-0.62**	0.07	-0.10	0.07
**Hum_jl**	-0.46	-0.18	-0.24	-0.12	-0.28	-0.30	-0.31	**-0.68**	-0.32
**Hum_aug**	-0.50	-0.21	-0.45	-0.05	-0.35	0.15	-0.26	**-0.62**	-0.15
**T_may**	-0.21	-0.13	0.16	-0.09	-0.05	-0.01	-0.18	-0.10	-0.23
**T_jn**	-0.01	0.19	-0.06	0.22	0.10	0.05	0.02	-0.07	0.35
**T_jl**	0.52	0.32	**0.65**	-0.54	-0.09	-0.53	0.23	0.01	-0.12
**T_aug**	0.27	0.19	0.51	0.06	0.40	-0.07	-0.05	0.21	-0.24
**Prec_jn**	0.12	0.24	0.14	-0.38	-0.38	**-0.70**	-0.24	-0.29	0.14
**Prec_jl**	-0.43	-0.14	-0.35	0.04	-0.36	0.16	-0.40	-0.41	-0.29
**Prec_aug**	0.05	0.54	0.27	0.08	0.18	0.10	-0.41	-0.16	0.29
**Wind_my**	0.14	0.07	0.18	-0.08	-0.11	0.04	-0.13	-0.01	0.01
**Wind_jn**	0.04	0.09	0.06	-0.30	-0.24	0.14	-0.29	0.17	-0.16
**Wind_jl**	-0.28	-0.15	-0.17	-0.01	0.11	0.40	-0.10	0.29	-0.32
**Wind_aug**	0.46	0.25	0.40	-0.44	-0.17	0.04	0.07	0.11	-0.31

Table S8. A Kendall tau correlation matrix for Siberian pine #1.

Weather par	orgCa*	alcCa	hclCa	orgSr	alcSr	hclSr	orgK	alcK	hclK	orgAl	alcAl	hclAl
**Iv_jn_ind****	0.31	0.28	-0.08	-0.05	0.18	**0.44**	-0.05	-0.08	-0.05	0.33	-0.23	0.26
**Iv_jl_ind**	-0.28	-0.26	0.15	-0.18	0.26	0.10	0.03	0.10	-0.28	0.05	-0.21	0.23
**Iv_au_ind**	-0.28	-0.36	0.00	0.23	-0.05	0.00	0.08	0.10	-0.03	0.15	-0.21	0.28
**Iv-Vys_jn**	0.33	0.31	0.10	0.03	0.15	0.36	-0.03	-0.15	0.03	0.10	-0.10	0.18
**Iv-Vys_jl**	-0.23	-0.26	0.15	-0.18	0.26	0.15	0.03	0.05	-0.23	0.00	-0.15	0.23
**Iv-Vys_aug**	-0.23	-0.31	0.15	0.13	-0.05	0.05	-0.08	-0.05	-0.08	0.05	-0.15	**0.44**
**Hum_may**	-0.18	-0.15	0.05	-0.18	-0.41	0.10	0.03	0.10	0.18	-0.10	0.21	-0.18
**Hum_jn**	**0.44**†	0.36	0.05	0.03	0.05	0.26	-0.18	-0.26	-0.08	0.10	0.00	0.08
**Hum_jl**	-0.15	-0.23	0.13	0.00	-0.03	0.33	0.05	-0.03	0.05	-0.03	-0.13	0.15
**Hum_aug**	-0.27	-0.35	0.22	-0.14	-0.01	0.30	-0.04	-0.06	-0.04	-0.06	-0.01	0.30
**T_may**	0.08	0.10	0.15	0.08	-0.05	-0.26	-0.18	-0.31	-0.28	-0.21	-0.05	0.18
**T_jn**	-0.03	-0.10	-0.05	-0.03	-0.05	-0.10	-0.18	-0.05	-0.28	0.21	0.05	0.13
**T_jl**	0.26	0.28	0.18	0.21	0.03	-0.03	-0.05	-0.33	0.36	-0.23	0.08	0.10
**T_aug**	-0.08	0.00	-0.21	0.08	**-0.56**	-0.15	0.23	0.00	0.08	-0.21	0.05	-0.33
**Prec_jn**	0.26	0.23	0.08	0.10	0.13	0.33	0.05	-0.13	0.10	0.13	-0.18	0.26
**Prec_jl**	-0.23	-0.21	0.15	-0.13	0.41	0.05	-0.03	0.10	-0.23	0.05	-0.21	0.13
**Prec_aug**	-0.13	-0.15	0.05	0.33	-0.15	0.00	0.03	-0.05	0.13	0.10	-0.21	0.23
**Wind_my**	0.21	0.23	0.03	0.18	0.26	-0.03	0.16	-0.03	0.13	-0.08	-0.05	0.16
**Wind_jn**	0.22	0.27	0.27	0.06	0.12	-0.01	0.12	-0.12	0.04	-0.06	-0.19	0.17
**Wind_jl**	-0.17	-0.09	0.06	0.01	0.14	-0.09	0.09	0.14	-0.04	-0.12	-0.04	-0.19
**Wind_aug**	-0.01	0.12	0.09	0.14	0.25	0.01	-0.17	0.01	0.35	-0.09	-0.22	-0.04

* as in Table S1

** as in Table S1

† as in Table S1

Table S8. A Kendall tau correlation matrix for Siberian pine #1 (continued 1).

Weather par	orgP	alcP	hclP	orgS	alcS	hclS	orgTi	alcTi	hclTi	orgMn	alcMn	hclMn
**Iv_jn_ind**	0.00	0.13	0.05	-0.18	0.10	0.10	**0.44**	0.21	**0.44**	0.31	**0.46**	0.23
**Iv_jl_ind**	**-0.54**	-0.15	-0.28	-0.10	-0.08	0.03	-0.15	0.03	0.15	0.13	0.18	0.26
**Iv_au_ind**	-0.18	0.05	0.23	0.36	0.13	0.08	-0.31	-0.23	-0.10	-0.18	-0.33	0.05
**Iv-Vys_jn**	0.03	0.10	-0.03	-0.15	0.18	0.08	0.36	0.28	0.36	0.23	0.38	0.21
**Iv-Vys_jl**	**-0.54**	-0.10	-0.13	-0.10	-0.03	-0.03	-0.10	-0.08	0.26	0.13	0.18	0.10
**Iv-Vys_aug**	-0.18	-0.10	0.08	0.26	0.13	-0.08	**-0.46**	-0.23	0.10	-0.03	-0.23	0.05
**Hum_may**	-0.08	0.05	-0.13	0.05	-0.08	-0.13	-0.10	0.08	0.21	-0.08	-0.18	-0.15
**Hum_jn**	0.18	0.05	-0.03	0.00	0.08	0.03	0.26	0.08	0.26	0.38	0.28	0.00
**Hum_jl**	-0.26	0.03	0.05	0.23	0.15	-0.21	-0.13	-0.15	**0.44**	0.10	0.10	-0.03
**Hum_aug**	**-0.58**	0.09	-0.04	0.06	0.32	-0.01	-0.27	-0.14	0.30	0.01	-0.04	-0.06
**T_may**	0.03	-0.36	-0.08	0.21	-0.08	0.03	-0.31	-0.08	-0.21	-0.03	-0.13	-0.05
**T_jn**	0.08	0.00	0.08	0.26	-0.13	-0.03	-0.26	-0.38	-0.05	0.13	-0.13	-0.10
**T_jl**	0.21	-0.18	-0.05	-0.23	0.21	-0.31	0.08	**0.51**	0.33	0.10	0.15	-0.03
**T_aug**	0.23	-0.21	-0.13	0.26	-0.08	-0.03	-0.21	0.18	-0.10	-0.33	-0.38	0.05
**Prec_jn**	0.05	0.08	0.00	-0.13	0.26	-0.10	0.28	0.31	**0.44**	0.26	0.36	0.23
**Prec_jl**	**-0.54**	-0.15	-0.23	-0.15	-0.18	-0.03	-0.15	-0.03	0.21	0.13	0.18	0.10
**Prec_aug**	0.03	0.00	0.18	0.41	0.13	-0.03	-0.26	-0.13	-0.05	-0.13	-0.33	0.05
**Wind_my**	-0.08	-0.03	-0.03	-0.18	0.03	-0.03	0.10	0.21	0.16	-0.03	-0.03	0.10
**Wind_jn**	-0.04	-0.06	-0.09	-0.22	0.06	0.17	0.14	0.30	-0.04	-0.06	0.14	0.25
**Wind_jl**	-0.04	-0.06	-0.09	-0.01	0.06	0.12	-0.01	-0.01	-0.35	-0.12	-0.01	0.04
**Wind_aug**	-0.01	-0.04	0.14	-0.30	0.12	0.09	0.22	0.30	-0.17	-0.14	0.06	-0.04

Table S8. A Kendall tau correlation matrix for Siberian pine #1 (continued 2).

Weather par	orgFe	alcFe	hclFe	orgCu	alcCu	hclCu	orgZn	alcZn	hclZn
**Iv_jn_ind**	0.18	0.08	-0.03	-0.18	-0.41	**-0.46**	-0.13	-0.31	0.13
**Iv_jl_ind**	-0.41	-0.15	-0.31	0.10	-0.23	0.08	-0.31	-0.28	-0.10
**Iv_au_ind**	-0.21	0.21	-0.10	0.26	0.18	0.28	-0.41	-0.18	0.31
**Iv-Vys_jn**	0.10	0.05	0.00	-0.26	-0.28	**-0.44**	-0.10	-0.18	0.10
**Iv-Vys_jl**	-0.41	-0.21	-0.31	0.05	-0.33	0.03	-0.26	**-0.44**	-0.21
**Iv-Vys_aug**	-0.10	0.26	0.00	0.10	-0.08	0.08	-0.36	-0.28	0.10
**Hum_may**	0.15	0.10	0.05	-0.10	0.03	-0.08	0.15	-0.13	-0.21
**Hum_jn**	0.26	0.05	0.10	-0.36	-0.28	**-0.44**	0.05	-0.03	0.05
**Hum_jl**	-0.33	-0.18	-0.13	-0.08	-0.15	-0.21	-0.23	**-0.51**	-0.28
**Hum_aug**	-0.35	-0.14	-0.30	-0.01	-0.30	0.12	-0.19	**-0.50**	-0.12
**T_may**	-0.15	0.00	0.10	0.00	-0.08	-0.08	-0.15	-0.08	-0.21
**T_jn**	0.00	0.21	-0.05	0.21	0.03	0.03	0.00	-0.08	0.26
**T_jl**	0.33	0.18	**0.44**	-0.38	-0.05	**-0.46**	0.13	0.05	-0.08
**T_aug**	0.21	0.15	0.31	0.05	0.28	-0.08	0.00	0.13	-0.15
**Prec_jn**	0.13	0.18	0.08	-0.28	-0.26	**-0.56**	-0.13	-0.21	0.18
**Prec_jl**	-0.36	-0.10	-0.26	0.00	-0.23	0.13	-0.31	-0.33	-0.21
**Prec_aug**	0.05	**0.51**	0.15	0.05	0.13	0.08	-0.31	-0.18	0.21
**Wind_my**	0.13	0.08	0.18	-0.03	-0.10	0.00	-0.08	0.00	0.03
**Wind_jn**	-0.04	0.09	0.06	-0.22	-0.19	0.09	-0.25	0.09	-0.17
**Wind_jl**	-0.22	-0.17	-0.12	-0.06	0.06	0.32	-0.09	0.22	-0.22
**Wind_aug**	0.32	0.17	0.27	-0.30	-0.12	0.04	0.04	0.09	-0.14

Table S9. A Spearman rho correlation matrix for Siberian pine #2.

Weather par	orgCa*	alcCa	hclCa	orgSr	alcSr	hclSr	orgK	alcK	hclK	orgAl	alcAl	hclAl
**Iv_jn_ind****	-0.01	0.06	0.20	0.41	**0.76**	0.13	-0.07	-0.06	0.04	-0.06	-0.06	0.29
**Iv_jl_ind**	-0.21	-0.22	**0.73**†	0.02	0.34	0.39	-0.41	0.12	0.24	-0.17	0.34	0.21
**Iv_au_ind**	-0.29	-0.22	-0.10	**-0.59**	-0.34	0.10	-0.12	0.43	-0.28	-0.45	0.08	0.02
**Iv-Vys_jn**	0.10	0.15	0.15	0.38	**0.66**	0.12	-0.13	-0.20	0.02	0.09	-0.11	0.32
**Iv-Vys_jl**	-0.23	-0.26	0.57	-0.13	0.42	0.24	-0.37	0.17	0.23	-0.23	0.37	0.19
**Iv-Vys_aug**	-0.27	-0.27	0.13	-0.38	-0.17	-0.01	-0.36	0.25	0.03	-0.42	0.12	0.33
**Hum_may**	-0.41	-0.48	0.06	0.25	0.31	-0.41	0.16	0.24	0.17	-0.25	-0.26	0.10
**Hum_jn**	0.45	0.48	0.02	0.36	0.37	0.14	-0.28	-0.48	0.02	0.22	-0.07	0.29
**Hum_jl**	-0.25	-0.31	0.34	-0.27	0.34	-0.08	-0.41	0.26	0.20	-0.53	0.23	0.20
**Hum_aug**	-0.36	-0.42	0.12	-0.39	0.20	-0.39	-0.46	0.26	0.23	-0.39	0.13	0.57
**T_may**	0.46	0.43	-0.01	-0.50	-0.43	0.38	-0.43	-0.36	-0.18	0.14	0.35	-0.15
**T_jn**	0.14	0.15	0.03	-0.20	-0.34	0.09	-0.45	-0.03	-0.10	-0.10	-0.10	0.21
**T_jl**	0.06	-0.09	-0.03	0.43	0.14	-0.30	0.12	-0.21	0.24	0.31	-0.50	0.15
**T_aug**	-0.15	-0.13	-0.28	0.01	-0.10	0.10	0.57	0.17	-0.27	-0.16	-0.06	-0.45
**Prec_jn**	0.08	0.08	0.25	0.41	0.56	0.13	-0.18	-0.17	0.09	0.05	-0.19	0.31
**Prec_jl**	-0.20	-0.22	**0.70**	0.03	0.24	0.35	-0.24	0.16	0.41	-0.16	0.45	0.10
**Prec_aug**	-0.15	-0.13	-0.19	-0.40	-0.38	-0.03	-0.08	0.25	-0.17	-0.38	-0.02	0.08
**Wind_my**	-0.06	-0.09	-0.08	0.15	0.14	-0.04	0.42	0.10	0.28	0.28	-0.12	0.01
**Wind_jn**	0.09	0.12	0.07	0.14	0.02	0.16	0.16	-0.18	0.20	0.26	0.27	0.08
**Wind_jl**	0.15	0.19	0.15	-0.13	-0.36	0.25	0.02	-0.18	-0.01	0.07	0.57	-0.36
**Wind_aug**	-0.13	-0.14	0.00	0.18	0.04	-0.16	0.38	-0.07	0.11	-0.04	0.32	-0.31

* as in Table S1

** as in Table S1

† as in Table S1

Table S9. A Spearman rho correlation matrix for Siberian pine #2 (continued 1).

Weather par	orgP	alcP	hclP	orgS	alcS	hclS	orgTi	alcTi	hclTi	orgMn	alcMn	hclMn
**Iv_jn_ind**	-0.33	0.11	0.31	-0.38	**-0.68**	0.28	0.57	-0.30	0.43	0.01	0.15	-0.08
**Iv_jl_ind**	-0.30	0.56	0.45	-0.08	**-0.64**	0.01	0.41	-0.52	0.46	0.27	0.10	-0.31
**Iv_au_ind**	-0.01	-0.11	-0.41	0.10	0.38	0.02	-0.15	0.09	0.24	-0.15	-0.04	-0.10
**Iv-Vys_jn**	-0.24	0.04	0.36	-0.24	**-0.60**	0.28	**0.64**	-0.24	0.42	0.00	0.11	-0.22
**Iv-Vys_jl**	-0.35	**0.66**	0.27	-0.13	**-0.63**	0.00	0.36	**-0.59**	0.41	0.27	0.13	-0.30
**Iv-Vys_aug**	-0.18	0.17	-0.18	0.17	0.22	-0.04	-0.08	-0.12	0.19	-0.03	-0.06	-0.16
**Hum_may**	-0.24	0.23	0.03	0.00	0.00	-0.17	-0.27	-0.19	-0.24	0.08	0.00	0.18
**Hum_jn**	0.06	-0.16	0.20	-0.18	-0.41	0.25	0.42	0.11	0.00	-0.16	-0.06	-0.06
**Hum_jl**	-0.37	**0.59**	-0.01	0.04	-0.52	0.33	0.29	-0.36	0.27	-0.10	-0.15	-0.23
**Hum_aug**	-0.40	**0.76**	-0.19	-0.07	-0.16	-0.04	0.07	**-0.65**	0.24	0.38	0.30	-0.39
**T_may**	0.36	-0.23	-0.13	0.41	0.26	-0.02	-0.01	0.40	-0.15	-0.37	-0.48	-0.17
**T_jn**	0.37	-0.17	-0.24	0.11	0.16	-0.03	-0.11	0.33	-0.33	-0.14	-0.10	-0.12
**T_jl**	0.01	-0.17	0.29	0.50	0.10	-0.12	0.23	0.10	-0.20	-0.18	-0.29	-0.10
**T_aug**	-0.01	**-0.61**	-0.17	0.17	**0.66**	-0.28	-0.52	0.45	-0.20	-0.45	-0.41	**0.59**
**Prec_jn**	-0.21	-0.01	0.43	-0.01	-0.56	0.32	**0.68**	-0.13	0.38	-0.15	-0.08	-0.24
**Prec_jl**	-0.38	**0.61**	0.40	-0.08	**-0.59**	0.01	0.36	-0.54	0.46	0.27	0.11	-0.14
**Prec_aug**	-0.03	-0.27	-0.36	0.26	0.51	0.09	-0.17	0.27	0.11	-0.34	-0.24	0.03
**Wind_my**	-0.20	-0.02	0.07	0.13	0.08	-0.29	0.35	-0.22	0.26	0.13	0.14	0.02
**Wind_jn**	-0.30	0.05	0.28	-0.05	0.03	-0.08	0.20	-0.27	0.45	0.20	0.15	0.03
**Wind_jl**	-0.06	0.13	0.22	-0.08	-0.03	0.25	-0.21	-0.03	0.22	0.10	-0.02	0.10
**Wind_aug**	-0.41	0.20	0.29	-0.07	0.02	0.20	-0.30	-0.20	0.21	0.06	-0.06	0.31

Table S9. A Spearman rho correlation matrix for Siberian pine #2 (continued 2).

Weather par	orgFe	alcFe	hclFe	orgCu	alcCu	hclCu	orgZn	alcZn	hclZn
**Iv_jn_ind**	0.39	0.20	-0.45	-0.13	0.16	0.18	-0.27	0.15	0.01
**Iv_jl_ind**	0.57	0.01	-0.57	0.06	0.29	0.43	-0.39	0.03	0.22
**Iv_au_ind**	0.01	-0.31	0.32	**0.64**	0.41	-0.12	-0.43	0.02	0.09
**Iv-Vys_jn**	0.34	0.14	-0.42	-0.17	0.14	0.24	-0.23	0.25	0.10
**Iv-Vys_jl**	0.45	-0.05	**-0.66**	0.18	0.31	0.42	-0.30	-0.02	0.15
**Iv-Vys_aug**	0.19	-0.26	0.09	0.38	0.16	0.07	-0.27	0.01	0.04
**Hum_may**	0.16	-0.17	-0.10	-0.24	-0.15	-0.34	0.37	-0.36	-0.23
**Hum_jn**	0.14	0.50	-0.08	-0.29	-0.26	0.29	0.00	0.39	0.01
**Hum_jl**	0.35	-0.03	**-0.59**	0.27	0.30	0.38	-0.25	-0.05	0.24
**Hum_aug**	0.13	-0.35	-0.50	0.52	0.07	0.28	-0.07	0.03	-0.22
**T_may**	-0.13	0.13	0.27	0.04	0.01	0.32	-0.08	0.28	0.45
**T_jn**	0.07	0.34	0.46	0.20	-0.24	0.03	0.04	0.30	-0.08
**T_jl**	0.17	-0.13	0.00	-0.57	-0.09	-0.13	0.44	-0.14	0.25
**T_aug**	-0.08	-0.22	**0.60**	-0.28	0.13	**-0.62**	0.09	-0.45	0.09
**Prec_jn**	0.45	0.13	-0.36	-0.26	0.19	0.24	-0.23	0.20	0.29
**Prec_jl**	0.47	0.04	**-0.59**	0.07	0.29	0.45	-0.38	-0.13	0.18
**Prec_aug**	-0.02	-0.24	0.43	0.37	0.23	-0.14	-0.29	-0.01	0.14
**Wind_my**	0.08	-0.21	-0.11	-0.03	0.33	-0.06	0.06	-0.32	0.07
**Wind_jn**	-0.04	-0.22	-0.22	-0.11	0.12	0.20	-0.24	-0.06	0.06
**Wind_jl**	-0.31	-0.08	-0.22	-0.05	-0.03	0.29	-0.33	0.04	0.16
**Wind_aug**	-0.27	-0.38	-0.41	-0.36	-0.04	-0.01	-0.07	-0.32	0.07

Table S10. A Kendall tau correlation matrix for Siberian pine #2.

Weather par	orgCa*	alcCa	hclCa	orgSr	alcSr	hclSr	orgK	alcK	hclK	orgAl	alcAl	hclAl
**Iv_jn_ind****	0.03	0.09	0.15	0.33	**0.58**	0.18	-0.03	-0.03	0.06	-0.09	-0.03	0.15
**Iv_jl_ind**	-0.12	-0.12	**0.48**†	0.00	0.24	0.27	-0.30	0.06	0.15	-0.12	0.24	0.12
**Iv_au_ind**	-0.18	-0.12	-0.06	-0.42	-0.24	0.15	-0.06	0.30	-0.21	-0.36	0.00	0.06
**Iv-Vys_jn**	0.09	0.15	0.09	0.33	**0.52**	0.18	-0.15	-0.15	0.00	0.03	-0.03	0.15
**Iv-Vys_jl**	-0.15	-0.15	0.39	-0.09	0.33	0.18	-0.27	0.09	0.18	-0.15	0.27	0.15
**Iv-Vys_aug**	-0.18	-0.18	0.12	-0.24	-0.12	0.03	-0.30	0.18	0.03	-0.36	0.06	0.24
**Hum_may**	-0.24	-0.36	0.00	0.12	0.24	-0.27	0.06	0.18	0.09	-0.18	-0.12	0.12
**Hum_jn**	0.27	0.33	0.03	0.33	0.27	0.18	-0.21	-0.27	0.06	0.15	-0.03	0.15
**Hum_jl**	-0.15	-0.21	0.27	-0.15	0.27	0.00	-0.33	0.21	0.18	-0.39	0.15	0.15
**Hum_aug**	-0.23	-0.29	0.17	-0.26	0.08	-0.20	-0.32	0.17	0.14	-0.26	0.08	0.35
**T_may**	0.39	0.33	0.03	-0.39	-0.27	0.24	-0.27	-0.27	-0.12	0.09	0.27	-0.15
**T_jn**	0.21	0.15	0.09	-0.21	-0.21	0.18	-0.33	-0.03	-0.06	-0.09	-0.09	0.15
**T_jl**	0.06	-0.06	-0.06	0.30	0.12	-0.21	0.12	-0.12	0.15	0.18	-0.36	0.12
**T_aug**	-0.18	-0.18	-0.18	0.00	-0.12	0.09	0.36	0.18	-0.15	-0.12	-0.06	-0.30
**Prec_jn**	0.03	-0.03	0.15	0.33	**0.45**	0.18	-0.15	-0.03	0.06	-0.03	-0.15	0.15
**Prec_jl**	-0.12	-0.12	**0.48**	0.00	0.18	0.27	-0.18	0.12	0.27	-0.12	0.30	0.06
**Prec_aug**	-0.09	-0.03	-0.15	-0.33	-0.21	0.00	-0.09	0.15	-0.12	-0.33	-0.03	0.09
**Wind_my**	-0.03	-0.09	-0.06	0.18	0.09	-0.03	0.37	0.03	0.22	0.22	-0.09	0.00
**Wind_jn**	0.08	0.14	0.05	0.11	0.05	0.05	0.17	-0.17	0.14	0.17	0.20	0.05
**Wind_jl**	0.06	0.06	0.12	0.00	-0.30	0.21	0.00	-0.12	0.03	0.06	0.42	-0.24
**Wind_aug**	-0.11	-0.08	-0.05	0.11	0.02	-0.11	0.23	-0.08	0.05	0.02	0.26	-0.23

* as in Table S1

** as in Table S1

† as in Table S1

Table S10. A Kendall tau correlation matrix for Siberian pine #2 (continued 1).

Weather par	orgP	alcP	hclP	orgS	alcS	hclS	orgTi	alcTi	hclTi	orgMn	alcMn	hclMn
**Iv_jn_ind**	-0.15	0.12	0.21	-0.21	**-0.55**	0.26	0.41	-0.18	0.33	0.00	0.09	-0.06
**Iv_jl_ind**	-0.24	0.39	0.30	-0.06	**-0.45**	0.00	0.38	-0.39	0.30	0.21	0.12	-0.27
**Iv_au_ind**	0.00	-0.03	-0.30	0.12	0.27	0.07	-0.11	0.03	0.18	-0.09	0.00	-0.09
**Iv-Vys_jn**	-0.15	0.06	0.21	-0.15	**-0.48**	0.26	**0.53**	-0.18	0.33	0.00	0.09	-0.18
**Iv-Vys_jl**	-0.27	**0.48**	0.21	-0.09	-0.42	-0.03	0.29	**-0.48**	0.27	0.18	0.09	-0.24
**Iv-Vys_aug**	-0.12	0.15	-0.12	0.12	0.09	-0.03	-0.05	-0.09	0.12	0.03	-0.06	-0.15
**Hum_may**	-0.18	0.15	0.00	-0.06	0.03	-0.16	-0.17	-0.15	-0.12	0.09	0.00	0.15
**Hum_jn**	0.09	-0.12	0.15	-0.09	-0.30	0.23	0.26	0.12	0.03	-0.12	-0.03	0.00
**Hum_jl**	-0.21	**0.48**	0.03	0.03	-0.36	0.23	0.17	-0.30	0.15	0.00	-0.09	-0.18
**Hum_aug**	-0.32	**0.56**	-0.08	-0.02	-0.26	0.02	0.06	**-0.44**	0.17	0.26	0.17	-0.32
**T_may**	0.27	-0.18	-0.09	0.27	0.18	-0.03	0.02	0.30	-0.15	-0.24	-0.33	-0.12
**T_jn**	0.27	-0.12	-0.15	0.03	0.12	-0.03	-0.02	0.24	-0.21	-0.12	-0.09	-0.12
**T_jl**	0.06	-0.15	0.18	0.36	0.03	-0.10	0.17	0.09	-0.18	-0.09	-0.18	-0.09
**T_aug**	0.00	-0.39	-0.12	0.12	**0.45**	-0.23	-0.38	0.33	-0.18	-0.33	-0.30	**0.45**
**Prec_jn**	-0.09	0.00	0.27	-0.03	-0.42	0.30	**0.60**	-0.12	0.27	-0.12	-0.09	-0.18
**Prec_jl**	-0.30	**0.45**	0.30	-0.06	**-0.45**	0.03	0.29	**-0.45**	0.30	0.21	0.18	-0.15
**Prec_aug**	-0.03	-0.18	-0.33	0.21	0.36	0.10	-0.08	0.12	0.09	-0.24	-0.09	0.00
**Wind_my**	-0.15	-0.03	0.03	0.09	0.09	-0.23	0.23	-0.15	0.22	0.09	0.15	0.06
**Wind_jn**	-0.17	0.02	0.23	-0.08	0.05	-0.08	0.03	-0.20	0.32	0.14	0.11	0.05
**Wind_jl**	-0.06	0.09	0.18	-0.06	-0.03	0.16	-0.14	-0.03	0.18	0.09	0.00	0.09
**Wind_aug**	-0.41	0.17	0.14	-0.05	-0.05	0.20	-0.18	-0.17	0.17	0.05	-0.02	0.20

Table S10. A Kendall tau correlation matrix for Siberian pine #2 (continued 2).

Weather par	orgFe	alcFe	hclFe	orgCu	alcCu	hclCu	orgZn	alcZn	hclZn
**Iv_jn_ind**	0.24	0.27	-0.39	-0.09	0.09	0.06	-0.24	0.09	0.00
**Iv_jl_ind**	**0.45**	0.00	-0.42	0.06	0.18	0.27	-0.27	0.00	0.09
**Iv_au_ind**	0.03	-0.24	0.24	**0.48**	0.30	-0.09	-0.33	0.00	0.09
**Iv-Vys_jn**	0.18	0.21	-0.33	-0.15	0.09	0.12	-0.18	0.21	0.06
**Iv-Vys_jl**	0.42	-0.03	**-0.45**	0.15	0.21	0.30	-0.24	-0.03	0.12
**Iv-Vys_aug**	0.15	-0.18	0.06	0.24	0.12	0.09	-0.21	0.06	0.03
**Hum_may**	0.09	-0.12	-0.12	-0.12	-0.06	-0.21	0.27	-0.24	-0.21
**Hum_jn**	0.12	**0.45**	-0.09	-0.21	-0.15	0.18	0.00	0.27	0.00
**Hum_jl**	0.30	-0.03	-0.39	0.21	0.21	0.30	-0.18	-0.03	0.18
**Hum_aug**	0.08	-0.23	-0.35	0.41	0.05	0.20	-0.14	-0.02	-0.17
**T_may**	-0.06	0.09	0.15	-0.03	0.03	0.24	-0.12	0.21	0.24
**T_jn**	0.06	0.33	0.33	0.15	-0.15	0.06	0.00	0.21	-0.06
**T_jl**	0.15	-0.06	0.00	-0.42	-0.12	-0.09	0.33	-0.12	0.15
**T_aug**	-0.09	-0.18	**0.48**	-0.18	0.12	-0.39	0.09	-0.30	0.09
**Prec_jn**	0.30	0.21	-0.21	-0.21	0.15	0.12	-0.18	0.09	0.24
**Prec_jl**	0.39	0.06	-0.42	0.06	0.18	0.27	-0.27	-0.06	0.03
**Prec_aug**	-0.06	-0.15	0.27	0.27	0.15	-0.12	-0.18	0.09	0.06
**Wind_my**	0.03	-0.18	-0.03	-0.03	0.28	-0.09	0.00	-0.28	0.06
**Wind_jn**	0.02	-0.17	-0.17	-0.11	0.08	0.11	-0.17	-0.05	-0.02
**Wind_jl**	-0.21	-0.06	-0.18	-0.06	0.00	0.21	-0.21	0.06	0.09
**Wind_aug**	-0.20	-0.29	-0.26	-0.26	-0.02	-0.08	-0.08	-0.23	0.05

Table S11. A Spearman rho correlation matrix for Siberian pine #3.

Weather par	orgCa*	alcCa	hclCa	orgSr	alcSr	hclSr	orgK	alcK	hclK	orgAl	alcAl	hclAl
**Iv_jn_ind****	-0.35	-0.29	-0.15	0.17	0.44	-0.15	0.40	-0.51	0.25	-0.15	-0.34	-0.01
**Iv_jl_ind**	-0.25	0.02	-0.22	0.44	0.04	-0.32	-0.26	**-0.69**	-0.11	-0.07	0.34	-0.11
**Iv_au_ind**	**0.71**†	0.37	0.45	-0.07	-0.24	-0.21	-0.21	0.37	-0.18	0.50	0.29	0.15
**Iv-Vys_jn**	-0.18	-0.15	-0.05	0.29	0.22	-0.30	0.52	-0.34	0.22	-0.07	-0.23	0.15
**Iv-Vys_jl**	-0.15	0.04	-0.06	0.42	0.17	-0.28	-0.19	**-0.76**	-0.09	-0.03	0.16	-0.28
**Iv-Vys_aug**	0.40	0.15	0.31	-0.05	-0.41	-0.09	-0.27	0.48	-0.26	0.34	0.20	0.19
**Hum_may**	0.13	-0.02	-0.35	-0.29	-0.02	-0.24	-0.06	0.32	0.60	0.00	0.09	-0.16
**Hum_jn**	-0.02	0.15	-0.10	0.30	0.03	-0.24	0.52	-0.10	-0.04	0.13	-0.20	-0.04
**Hum_jl**	-0.10	-0.01	0.18	**0.64**	0.06	-0.41	0.13	**-0.86**	0.04	-0.08	-0.09	-0.32
**Hum_aug**	0.14	-0.10	-0.32	0.27	-0.30	-0.60	-0.54	0.19	0.33	-0.15	0.60	0.15
**T_may**	0.15	0.43	0.17	0.29	-0.07	0.15	0.60	0.01	-0.38	0.13	-0.36	-0.25
**T_jn**	-0.21	-0.01	0.05	0.29	-0.59	-0.32	0.09	-0.08	-0.01	0.00	0.17	0.13
**T_jl**	0.21	-0.04	0.05	-0.30	0.04	0.37	0.15	**0.71**	-0.02	-0.03	-0.32	0.13
**T_aug**	0.57	**0.79**	0.07	**-0.63**	0.18	0.08	0.28	0.15	-0.11	**0.82**	0.25	0.08
**Prec_jn**	-0.21	-0.20	0.06	0.44	0.08	-0.33	0.45	-0.37	0.10	-0.10	-0.24	0.14
**Prec_jl**	-0.16	-0.07	-0.05	0.50	0.13	-0.12	-0.37	-0.59	-0.26	-0.17	0.07	-0.35
**Prec_aug**	0.45	0.13	0.33	-0.31	-0.18	0.12	-0.19	0.59	-0.22	0.37	0.05	0.23
**Wind_my**	0.32	0.08	0.44	-0.11	-0.06	0.20	0.15	0.41	-0.16	0.09	-0.23	-0.04
**Wind_jn**	0.28	0.10	-0.05	0.16	0.31	0.01	0.00	0.15	-0.13	-0.05	0.02	0.11
**Wind_jl**	-0.14	0.00	-0.39	-0.09	0.48	0.56	-0.26	0.11	-0.43	-0.16	-0.15	-0.16
**Wind_aug**	-0.11	-0.33	-0.17	-0.10	0.40	**0.69**	-0.37	0.37	-0.30	-0.44	-0.33	-0.06

* as in Table S1

** as in Table S1

† as in Table S1

Table S11. A Spearman rho correlation matrix for Siberian pine #3 (continued 1).

Weather par	orgP	alcP	hclP	orgS	alcS	hclS	orgTi	alcTi	hclTi	orgMn	alcMn	hclMn
**Iv_jn_ind**	0.47	0.17	-0.60	-0.35	-0.59	0.38	0.46	0.13	-0.02	-0.18	-0.24	0.32
**Iv_jl_ind**	-0.23	0.03	0.06	0.15	0.20	-0.27	-0.47	0.28	-0.21	0.54	**0.67**	**0.65**
**Iv_au_ind**	-0.31	-0.42	-0.08	0.58	-0.19	-0.56	0.03	-0.17	0.13	0.31	-0.06	-0.51
**Iv-Vys_jn**	0.49	0.13	-0.57	-0.13	-0.57	0.20	0.46	-0.03	-0.02	-0.25	-0.27	0.34
**Iv-Vys_jl**	-0.15	-0.02	-0.09	0.18	0.10	-0.12	-0.26	0.39	-0.10	0.59	**0.65**	0.49
**Iv-Vys_aug**	-0.21	-0.15	-0.10	0.41	-0.13	-0.50	0.02	-0.18	0.15	0.15	-0.13	-0.35
**Hum_may**	0.15	-0.03	0.34	-0.08	-0.30	-0.05	0.38	**-0.71**	0.25	0.02	-0.22	-0.40
**Hum_jn**	**0.62**	-0.14	-0.48	0.07	-0.49	0.15	0.24	-0.15	-0.16	0.00	-0.15	0.26
**Hum_jl**	0.09	0.25	-0.35	0.43	0.04	0.00	0.27	0.46	0.29	0.43	0.54	0.50
**Hum_aug**	-0.48	0.03	0.52	0.30	-0.22	**-0.80**	-0.16	**-0.67**	0.17	0.35	0.35	0.11
**T_may**	0.51	-0.22	-0.21	0.31	0.12	0.35	-0.02	0.17	-0.14	0.02	0.10	0.15
**T_jn**	0.15	0.42	0.00	0.28	0.43	-0.20	0.08	0.00	0.15	-0.09	0.09	0.37
**T_jl**	0.14	-0.12	-0.01	-0.15	-0.37	0.16	0.25	-0.34	0.13	-0.40	-0.52	-0.50
**T_aug**	0.20	**-0.87**	-0.05	0.07	0.02	0.02	-0.30	-0.10	-0.55	0.17	-0.33	-0.50
**Prec_jn**	0.44	0.25	**-0.64**	0.00	-0.48	0.13	0.47	0.09	0.07	-0.20	-0.17	0.42
**Prec_jl**	-0.30	0.03	-0.02	0.20	0.08	-0.15	-0.35	0.41	-0.02	**0.64**	**0.75**	0.46
**Prec_aug**	-0.15	-0.26	-0.18	0.18	-0.28	-0.30	0.11	-0.17	0.10	-0.02	-0.39	-0.59
**Wind_my**	0.01	-0.11	-0.08	0.09	0.05	0.17	0.18	0.04	0.07	-0.23	-0.23	-0.43
**Wind_jn**	-0.20	-0.42	0.01	0.05	-0.50	-0.16	-0.25	-0.17	-0.21	0.12	0.09	-0.01
**Wind_jl**	-0.17	-0.44	0.14	-0.40	-0.26	0.18	**-0.69**	0.09	-0.48	0.25	0.23	0.08
**Wind_aug**	-0.39	-0.09	0.21	-0.39	-0.28	0.17	-0.34	0.07	-0.04	-0.06	0.07	-0.16

Table S11. A Spearman rho correlation matrix for Siberian pine #3 (continued 2).

Weather par	orgFe	alcFe	hclFe	orgCu	alcCu	hclCu	orgZn	alcZn	hclZn
**Iv_jn_ind**	**0.61**	**0.70**	0.36	-0.24	-0.45	0.02	0.02	0.25	0.37
**Iv_jl_ind**	0.07	-0.04	0.05	0.56	-0.23	0.42	-0.34	-0.19	-0.04
**Iv_au_ind**	-0.09	-0.40	-0.40	0.13	0.26	-0.42	0.32	0.31	-0.45
**Iv-Vys_jn**	**0.63**	0.51	0.12	-0.41	-0.44	-0.15	0.02	0.33	0.39
**Iv-Vys_jl**	0.05	0.02	0.08	0.55	-0.11	0.39	-0.36	-0.22	-0.20
**Iv-Vys_aug**	-0.23	-0.45	-0.45	0.01	0.04	-0.54	0.23	0.31	-0.30
**Hum_may**	0.01	0.25	0.45	0.10	0.52	0.13	0.29	0.07	-0.05
**Hum_jn**	0.40	0.22	-0.10	**-0.61**	-0.27	-0.22	-0.05	0.07	-0.01
**Hum_jl**	0.10	0.02	-0.05	0.39	-0.23	0.18	-0.46	0.08	-0.10
**Hum_aug**	-0.02	-0.20	0.07	0.55	0.28	-0.03	0.01	0.05	0.09
**T_may**	-0.05	-0.24	-0.43	**-0.71**	-0.06	-0.15	-0.33	-0.30	-0.30
**T_jn**	-0.20	-0.34	-0.34	0.01	-0.22	-0.05	-0.23	0.20	0.13
**T_jl**	0.01	0.05	-0.05	-0.58	0.07	-0.48	0.30	0.12	0.08
**T_aug**	0.26	0.14	0.05	-0.24	0.29	0.26	**0.65**	0.07	-0.50
**Prec_jn**	0.50	0.34	-0.05	-0.35	-0.52	-0.26	-0.12	0.35	0.35
**Prec_jl**	-0.15	-0.16	-0.04	0.55	-0.11	0.21	-0.50	-0.36	-0.25
**Prec_aug**	-0.11	-0.23	-0.29	-0.14	0.06	-0.55	0.44	0.35	-0.25
**Wind_my**	-0.25	-0.28	-0.34	-0.36	0.33	-0.33	-0.06	-0.17	-0.23
**Wind_jn**	0.31	0.07	-0.04	-0.20	0.03	-0.21	0.05	-0.16	0.01
**Wind_jl**	0.05	0.14	0.18	-0.15	-0.12	0.07	-0.04	-0.57	-0.12
**Wind_aug**	-0.13	0.04	0.13	-0.10	-0.06	-0.23	-0.06	-0.39	0.11

Table S12. A Kendall tau correlation matrix for Siberian pine #3.

Weather par	orgCa*	alcCa	hclCa	orgSr	alcSr	hclSr	orgK	alcK	hclK	orgAl	alcAl	hclAl
**Iv_jn_ind****	-0.24	-0.09	-0.13	0.09	0.35	-0.05	0.31	-0.38	0.16	-0.09	-0.31	-0.02
**Iv_jl_ind**	-0.20	-0.05	-0.16	0.27	0.02	-0.31	-0.16	**-0.49**	-0.09	-0.05	0.31	-0.05
**Iv_au_ind**	0.56	0.20	0.31	0.02	-0.16	-0.13	-0.13	0.27	-0.20	0.35	0.20	0.13
**Iv-Vys_jn**	-0.09	-0.09	-0.05	0.24	0.13	-0.20	0.45	-0.31	0.16	-0.02	-0.24	0.13
**Iv-Vys_jl**	-0.09	0.05	-0.05	0.24	0.13	-0.27	-0.13	**-0.53**	-0.05	-0.02	0.13	-0.24
**Iv-Vys_aug**	0.27	0.13	0.24	-0.05	-0.24	-0.13	-0.20	0.35	-0.27	0.20	0.13	0.13
**Hum_may**	0.05	-0.02	-0.27	-0.20	-0.02	-0.20	-0.05	0.27	0.45	0.05	0.05	-0.16
**Hum_jn**	0.04	0.11	-0.07	0.22	0.04	-0.18	0.44	0.00	0.00	0.07	-0.15	-0.07
**Hum_jl**	-0.05	0.02	0.13	**0.49**	0.02	-0.31	0.13	**-0.71**	-0.02	-0.05	-0.05	-0.20
**Hum_aug**	0.05	-0.09	-0.27	0.16	-0.24	-0.42	-0.35	0.13	0.24	-0.09	**0.49**	0.13
**T_may**	0.09	0.31	0.13	0.20	-0.13	0.13	0.42	0.09	-0.24	0.09	-0.27	-0.20
**T_jn**	-0.16	-0.02	0.02	0.16	-0.45	-0.27	0.09	-0.02	0.02	0.05	0.13	0.13
**T_jl**	0.16	0.02	-0.02	-0.16	-0.05	0.27	0.13	**0.53**	-0.02	-0.05	-0.27	0.09
**T_aug**	0.35	**0.64**†	0.02	**-0.49**	0.13	0.09	0.31	0.13	-0.13	**0.64**	0.13	-0.02
**Prec_jn**	-0.05	-0.13	0.05	0.35	0.02	-0.24	**0.49**	-0.35	0.05	-0.05	-0.20	0.09
**Prec_jl**	-0.09	-0.02	0.02	0.31	0.13	-0.13	-0.27	-0.38	-0.20	-0.09	0.05	-0.31
**Prec_aug**	0.27	0.05	0.24	-0.20	-0.09	0.09	-0.20	**0.49**	-0.20	0.27	0.05	0.20
**Wind_my**	0.28	0.06	0.31	-0.13	0.06	0.13	0.09	0.31	-0.09	0.09	-0.13	-0.02
**Wind_jn**	0.22	0.07	-0.04	0.11	0.18	0.00	-0.04	0.11	-0.15	0.00	0.00	0.07
**Wind_jl**	-0.13	-0.02	-0.31	-0.06	0.35	**0.50**	-0.17	0.06	-0.31	-0.02	-0.17	-0.09
**Wind_aug**	-0.07	-0.30	-0.15	-0.07	0.34	**0.56**	-0.22	0.22	-0.26	-0.34	-0.26	-0.07

* as in Table S1

** as in Table S1

† as in Table S1

Table S12. A Kendall tau correlation matrix for Siberian pine #3 (continued 1).

Weather par	orgP	alcP	hclP	orgS	alcS	hclS	orgTi	alcTi	hclTi	orgMn	alcMn	hclMn
**Iv_jn_ind**	0.38	0.20	-0.42	-0.27	-0.42	0.38	0.38	0.09	-0.02	-0.20	-0.20	0.16
**Iv_jl_ind**	-0.16	0.09	0.05	0.13	0.13	-0.24	-0.31	0.20	-0.13	0.42	**0.49**	**0.49**
**Iv_au_ind**	-0.20	-0.24	0.02	0.38	-0.13	-0.42	0.09	-0.13	0.05	0.24	-0.05	-0.35
**Iv-Vys_jn**	0.45	0.13	-0.42	-0.13	-0.42	0.16	0.38	-0.05	-0.02	-0.20	-0.20	0.24
**Iv-Vys_jl**	-0.13	-0.02	-0.05	0.16	0.09	-0.13	-0.20	0.31	-0.02	0.45	**0.53**	0.38
**Iv-Vys_aug**	-0.13	-0.09	-0.05	0.24	-0.13	-0.35	0.09	-0.13	0.13	0.09	-0.13	-0.27
**Hum_may**	0.09	-0.02	0.16	0.02	-0.20	-0.05	0.24	**-0.49**	0.13	0.09	-0.13	-0.27
**Hum_jn**	**0.51**	-0.04	-0.37	0.07	-0.37	0.07	0.15	-0.07	-0.11	-0.04	-0.07	0.15
**Hum_jl**	0.13	0.24	-0.24	0.35	0.05	0.05	0.20	0.27	0.24	0.27	0.42	0.35
**Hum_aug**	-0.35	-0.02	0.38	0.16	-0.20	**-0.64**	-0.13	**-0.49**	0.05	0.24	0.24	0.02
**T_may**	0.35	-0.20	-0.16	0.20	0.13	0.27	-0.02	0.13	-0.05	-0.02	0.05	0.13
**T_jn**	0.16	0.35	0.02	0.16	0.31	-0.20	0.09	0.02	0.13	-0.05	0.16	0.16
**T_jl**	0.13	-0.13	-0.09	-0.09	-0.24	0.13	0.20	-0.31	0.09	-0.31	-0.38	-0.31
**T_aug**	0.16	**-0.75**	0.02	-0.05	0.02	0.02	-0.20	-0.05	-0.45	0.09	-0.27	-0.42
**Prec_jn**	**0.49**	0.16	-0.45	-0.02	-0.31	0.13	0.35	0.05	0.02	-0.24	-0.09	0.27
**Prec_jl**	-0.20	0.05	0.02	0.16	0.02	-0.13	-0.27	0.38	-0.02	0.45	**0.60**	0.31
**Prec_aug**	-0.13	-0.09	-0.13	0.09	-0.27	-0.20	0.16	-0.13	0.05	0.02	-0.27	-0.42
**Wind_my**	-0.06	-0.09	-0.06	0.06	-0.02	0.13	0.17	0.02	0.06	-0.17	-0.17	-0.43
**Wind_jn**	-0.18	-0.40	0.00	0.04	-0.40	-0.07	-0.15	-0.18	-0.18	0.11	0.04	0.04
**Wind_jl**	-0.09	-0.31	0.06	-0.31	-0.13	0.13	**-0.50**	0.06	-0.39	0.20	0.17	0.02
**Wind_aug**	-0.22	-0.07	0.22	-0.30	-0.15	0.15	-0.26	0.00	-0.07	-0.04	0.11	-0.11

Table S12. A Kendall tau correlation matrix for Siberian pine #3 (continued 2).

Weather par	orgFe	alcFe	hclFe	orgCu	alcCu	hclCu	orgZn	alcZn	hclZn
**Iv_jn_ind**	0.45	**0.53**	0.27	-0.20	-0.35	0.02	0.02	0.16	0.31
**Iv_jl_ind**	0.05	-0.02	0.02	0.42	-0.24	0.27	-0.16	-0.09	-0.02
**Iv_au_ind**	-0.13	-0.35	-0.24	0.16	0.16	-0.27	0.24	0.24	-0.35
**Iv-Vys_jn**	0.45	0.31	0.05	-0.35	-0.35	-0.13	0.02	0.24	0.31
**Iv-Vys_jl**	0.02	0.02	0.05	0.38	-0.05	0.31	-0.20	-0.13	-0.13
**Iv-Vys_aug**	-0.13	-0.27	-0.31	0.02	0.09	-0.35	0.16	0.16	-0.20
**Hum_may**	0.02	0.16	0.35	0.09	0.38	0.09	0.24	0.02	-0.13
**Hum_jn**	0.29	0.15	-0.04	-0.44	-0.15	-0.15	0.04	0.00	0.00
**Hum_jl**	0.05	-0.02	-0.05	0.27	-0.24	0.13	-0.31	0.05	-0.09
**Hum_aug**	0.02	-0.13	-0.02	0.38	0.24	-0.05	-0.05	0.02	0.09
**T_may**	-0.02	-0.16	-0.35	**-0.53**	-0.02	-0.09	-0.24	-0.24	-0.16
**T_jn**	-0.05	-0.27	-0.24	0.02	-0.20	-0.05	-0.13	0.09	0.16
**T_jl**	-0.02	-0.02	-0.05	-0.38	0.13	-0.31	0.13	-0.02	0.05
**T_aug**	0.16	0.09	-0.02	-0.20	0.24	0.24	0.45	0.09	-0.42
**Prec_jn**	0.35	0.20	-0.05	-0.31	-0.38	-0.16	-0.09	0.20	0.27
**Prec_jl**	-0.13	-0.13	-0.02	0.38	-0.05	0.16	-0.27	-0.20	-0.13
**Prec_aug**	-0.05	-0.20	-0.16	-0.05	0.02	-0.42	0.31	0.24	-0.20
**Wind_my**	-0.13	-0.20	-0.24	-0.31	0.20	-0.24	0.02	-0.13	-0.13
**Wind_jn**	0.18	-0.07	-0.04	-0.18	0.00	-0.11	0.04	-0.11	0.00
**Wind_jl**	-0.02	0.06	0.17	-0.17	-0.13	-0.02	-0.06	**-0.46**	-0.09
**Wind_aug**	-0.22	0.00	0.11	-0.04	-0.07	-0.11	-0.04	-0.26	0.04

Table S13. A Spearman rho correlation matrix for Siberian larch #1.

Weather par	orgCa*	alcCa	hclCa	orgSr	alcSr	hclSr	orgK	alcK	hclK	orgAl	alcAl	hclAl
**Iv_jn_ind****	-0.43	-0.22	0.01	-0.34	0.47	0.41	-0.33	-0.17	-0.15	0.03	0.16	-0.24
**Iv_jl_ind**	-0.27	0.32	-0.13	-0.43	0.00	0.40	0.06	-0.04	-0.02	-0.38	0.09	-0.18
**Iv_au_ind**	0.39	0.04	-0.13	0.38	-0.15	0.25	0.45	0.03	**-0.79**	0.44	0.09	-0.09
**Iv-Vys_jn**	-0.34	-0.41	0.13	-0.35	0.26	0.50	-0.45	-0.22	-0.31	-0.06	0.15	-0.27
**Iv-Vys_jl**	-0.36	0.27	-0.25	-0.25	0.22	0.46	0.14	-0.01	-0.09	-0.23	0.13	-0.20
**Iv-Vys_aug**	0.51	0.11	0.07	0.30	-0.18	0.13	0.30	-0.06	**-0.65**	0.36	0.01	-0.08
**Hum_may**	0.22	0.33	0.12	0.53	0.35	-0.19	0.31	0.27	-0.17	0.22	0.06	0.45
**Hum_jn**	-0.33	-0.50	-0.05	-0.11	0.04	0.55	-0.55	-0.17	-0.46	-0.09	0.36	-0.26
**Hum_jl**	-0.27	0.23	0.17	-0.33	0.32	**0.70**	-0.01	-0.03	-0.24	-0.35	-0.10	-0.12
**Hum_aug**	0.45	0.50	0.05	0.24	0.18	0.16	0.52	-0.13	-0.37	0.14	-0.09	-0.05
**T_may**	-0.31	-0.60	-0.08	-0.05	-0.35	0.30	**-0.65**	-0.09	0.02	-0.33	0.15	-0.15
**T_jn**	0.12	0.07	**0.65**†	-0.31	-0.32	0.07	-0.26	0.13	-0.05	-0.56	-0.15	0.24
**T_jl**	0.35	-0.25	-0.01	0.35	-0.03	-0.24	-0.07	-0.19	-0.03	0.45	-0.10	-0.05
**T_aug**	-0.31	-0.35	-0.35	0.06	-0.57	-0.04	-0.05	0.51	-0.38	0.02	0.59	0.28
**Prec_jn**	-0.26	-0.36	0.21	-0.38	0.25	0.57	-0.44	-0.28	-0.35	-0.10	0.07	-0.34
**Prec_jl**	-0.18	0.35	-0.36	-0.09	0.28	0.42	0.22	-0.21	-0.02	-0.05	0.01	-0.36
**Prec_aug**	0.46	-0.01	-0.05	0.37	-0.12	-0.03	0.31	-0.05	-0.58	0.57	0.05	-0.07
**Wind_my**	0.00	-0.47	-0.23	0.46	0.03	-0.24	-0.03	-0.05	0.06	0.28	0.00	-0.10
**Wind_jn**	-0.03	-0.31	-0.56	0.10	0.07	0.28	0.03	-0.42	-0.18	0.37	0.12	-0.54
**Wind_jl**	-0.14	-0.05	**-0.81**	0.06	0.00	-0.05	-0.03	-0.38	0.30	0.35	0.21	-0.49
**Wind_aug**	0.27	0.08	-0.54	0.23	0.21	-0.28	0.18	-0.53	0.47	0.59	-0.24	-0.47

* as in Table S1

** as in Table S1

† as in Table S1

Table S13. A Spearman rho correlation matrix for Siberian larch #1 (continued 1).

Weather par	orgP	alcP	hclP	orgS	alcS	hclS	orgTi	alcTi	hclTi	orgMn	alcMn	hclMn
**Iv_jn_ind**	0.06	-0.12	0.57	0.47	-0.45	0.07	-0.52	0.22	0.58	0.45	-0.01	0.40
**Iv_jl_ind**	-0.55	0.14	-0.55	-0.18	0.60	0.30	-0.35	-0.19	0.01	**-0.72**	-0.28	-0.48
**Iv_au_ind**	0.30	-0.25	-0.34	0.45	-0.25	0.11	0.10	-0.48	0.16	0.33	0.53	-0.45
**Iv-Vys_jn**	0.17	-0.05	0.47	0.51	-0.41	0.24	-0.47	0.06	**0.71**	0.53	-0.02	0.35
**Iv-Vys_jl**	-0.40	0.05	-0.38	-0.06	0.45	0.22	-0.32	-0.08	0.07	-0.53	-0.07	-0.36
**Iv-Vys_aug**	0.27	-0.12	-0.30	0.44	-0.24	0.06	0.12	-0.26	0.08	0.25	0.31	-0.35
**Hum_may**	0.40	-0.45	0.32	-0.15	-0.59	0.14	0.38	0.35	0.14	0.28	0.42	0.19
**Hum_jn**	0.25	-0.01	0.27	0.51	-0.17	0.17	-0.26	0.02	0.49	0.38	-0.05	0.24
**Hum_jl**	-0.05	-0.03	-0.28	0.07	0.08	0.55	-0.51	0.17	0.55	-0.25	0.06	-0.20
**Hum_aug**	0.13	-0.29	-0.26	-0.12	-0.04	0.40	0.31	-0.20	0.13	-0.05	0.33	-0.32
**T_may**	0.21	0.22	-0.04	0.06	0.34	0.03	-0.04	0.02	0.03	0.01	-0.21	0.21
**T_jn**	-0.13	0.33	-0.41	-0.21	0.16	0.52	-0.15	0.19	0.26	-0.41	-0.36	-0.24
**T_jl**	0.56	-0.23	0.47	0.29	-0.49	-0.33	0.29	0.04	-0.07	**0.71**	0.26	0.45
**T_aug**	-0.21	-0.09	-0.27	0.12	0.02	-0.14	-0.16	-0.55	-0.12	-0.08	-0.02	-0.40
**Prec_jn**	0.17	0.03	0.35	0.55	-0.34	0.30	-0.49	0.09	**0.74**	0.46	-0.04	0.27
**Prec_jl**	-0.28	0.04	-0.36	-0.03	0.53	0.05	-0.13	-0.05	-0.13	-0.47	0.00	-0.29
**Prec_aug**	0.34	-0.21	-0.03	0.55	-0.45	-0.18	0.13	-0.27	0.02	0.50	0.39	-0.17
**Wind_my**	0.30	0.13	0.34	0.09	-0.04	-0.36	0.47	0.01	-0.19	0.43	0.23	0.31
**Wind_jn**	0.20	-0.20	0.19	0.33	0.08	-0.22	0.09	-0.49	-0.05	0.43	0.25	0.12
**Wind_jl**	-0.16	-0.07	0.11	0.11	0.45	**-0.68**	0.12	-0.29	**-0.68**	-0.06	-0.11	0.14
**Wind_aug**	0.17	-0.17	0.35	0.09	0.11	**-0.72**	0.33	-0.09	**-0.64**	0.29	0.15	0.39

Table S13. A Spearman rho correlation matrix for Siberian larch #1 (continued 2).

Weather par	orgFe	alcFe	hclFe	orgCu	alcCu	hclCu	orgZn	alcZn	hclZn
**Iv_jn_ind**	-0.15	0.41	0.55	0.55	-0.06	**-0.83**	-0.01	-0.08	0.15
**Iv_jl_ind**	-0.27	0.15	-0.09	0.06	-0.31	0.51	-0.14	-0.48	0.10
**Iv_au_ind**	**0.72**	-0.13	0.35	-0.36	0.29	0.05	-0.25	0.09	-0.82
**Iv-Vys_jn**	-0.04	0.25	**0.71**	0.50	-0.01	**-0.76**	0.08	-0.14	-0.06
**Iv-Vys_jl**	-0.24	0.33	-0.06	0.16	-0.17	0.34	-0.16	-0.49	0.10
**Iv-Vys_aug**	0.49	-0.15	0.46	-0.41	0.08	-0.03	-0.18	0.08	-0.67
**Hum_may**	0.04	0.34	0.32	-0.22	0.54	-0.28	0.45	0.33	0.11
**Hum_jn**	0.01	0.02	**0.81**	0.48	0.03	-0.53	0.13	-0.03	-0.10
**Hum_jl**	-0.27	0.50	0.41	0.38	-0.01	-0.04	-0.15	**-0.84**	-0.06
**Hum_aug**	0.06	0.33	0.31	**-0.65**	0.13	0.29	0.45	-0.09	-0.25
**T_may**	-0.08	-0.35	0.20	0.52	-0.02	0.05	0.02	-0.16	0.03
**T_jn**	-0.26	-0.12	0.31	0.02	-0.06	0.18	0.09	-0.47	-0.06
**T_jl**	0.29	-0.18	0.22	-0.12	0.12	-0.46	0.11	0.50	-0.15
**T_aug**	**0.70**	**-0.69**	-0.15	0.25	0.35	0.17	-0.46	0.58	-0.33
**Prec_jn**	-0.09	0.28	**0.77**	0.45	-0.09	**-0.71**	0.05	-0.32	-0.14
**Prec_jl**	-0.29	0.37	-0.11	-0.02	-0.31	0.40	-0.11	-0.48	0.11
**Prec_aug**	**0.63**	-0.20	0.35	-0.35	0.14	-0.25	-0.26	0.36	-0.64
**Wind_my**	0.14	-0.06	-0.28	-0.07	0.17	-0.06	0.15	0.19	-0.05
**Wind_jn**	0.30	-0.03	0.04	-0.08	-0.09	-0.04	0.07	0.23	-0.21
**Wind_jl**	-0.01	-0.19	-0.41	-0.03	-0.46	0.20	-0.11	0.46	0.28
**Wind_aug**	-0.03	0.02	-0.45	-0.30	-0.40	0.00	0.00	0.39	0.21

Table S14. A Kendall tau correlation matrix for Siberian larch #1.

Weather par	orgCa*	alcCa	hclCa	orgSr	alcSr	hclSr	orgK	alcK	hclK	orgAl	alcAl	hclAl
**Iv_jn_ind****	-0.35	-0.13	0.02	-0.27	0.31	0.24	-0.20	-0.13	-0.09	0.05	0.09	-0.20
**Iv_jl_ind**	-0.16	0.20	-0.09	-0.31	-0.02	0.35	0.05	-0.02	0.02	-0.27	0.05	-0.16
**Iv_au_ind**	0.31	0.02	-0.05	0.24	-0.13	0.16	0.31	0.02	**-0.60**	0.27	0.09	-0.13
**Iv-Vys_jn**	-0.27	-0.27	0.09	-0.20	0.16	0.31	-0.35	-0.20	-0.24	-0.02	0.09	-0.20
**Iv-Vys_jl**	-0.20	0.16	-0.20	-0.13	0.09	0.38	0.09	0.02	-0.09	-0.16	0.09	-0.13
**Iv-Vys_aug**	0.38	0.09	0.09	0.16	-0.13	0.09	0.24	-0.05	-0.45	0.27	0.02	-0.13
**Hum_may**	0.16	0.24	0.09	0.45	0.24	-0.13	0.24	0.24	-0.16	0.13	0.09	0.38
**Hum_jn**	-0.26	-0.37	0.00	-0.04	0.04	0.44	-0.40	-0.15	-0.33	0.00	0.26	-0.22
**Hum_jl**	-0.16	0.20	0.13	-0.24	0.27	**0.56**	0.05	-0.02	-0.13	-0.20	-0.09	-0.09
**Hum_aug**	0.31	0.31	0.09	0.16	0.16	0.09	0.38	-0.13	-0.24	0.13	-0.05	-0.05
**T_may**	-0.24	-0.45	-0.02	-0.02	-0.31	0.20	**-0.53**	-0.09	0.02	-0.20	0.05	-0.09
**T_jn**	0.09	0.02	0.45	-0.27	-0.20	0.09	-0.20	0.02	-0.09	-0.38	-0.13	0.09
**T_jl**	0.20	-0.16	-0.02	0.27	-0.02	-0.24	-0.02	-0.16	-0.05	0.31	-0.02	-0.02
**T_aug**	-0.20	-0.27	-0.20	0.02	-0.42	0.02	0.02	0.38	-0.31	0.05	0.45	0.24
**Prec_jn**	-0.24	-0.31	0.20	-0.24	0.20	0.42	-0.38	-0.24	-0.20	0.02	0.05	-0.24
**Prec_jl**	-0.13	0.24	-0.27	-0.05	0.16	0.31	0.16	-0.13	-0.02	-0.02	0.02	-0.27
**Prec_aug**	0.31	0.02	-0.05	0.24	-0.13	-0.05	0.24	-0.05	-0.45	0.35	0.09	-0.13
**Wind_my**	-0.02	-0.39	-0.20	0.31	0.02	-0.20	-0.02	-0.02	0.06	0.17	-0.06	-0.09
**Wind_jn**	-0.07	-0.22	-0.40	0.07	0.07	0.18	0.00	-0.26	-0.11	0.18	0.11	-0.33
**Wind_jl**	-0.17	-0.02	**-0.61**†	-0.02	0.02	-0.06	-0.02	-0.28	0.17	0.17	0.17	-0.35
**Wind_aug**	0.19	0.04	-0.30	0.19	0.15	-0.22	0.11	-0.34	0.41	0.41	-0.15	-0.34

* as in Table S1

** as in Table S1

† as in Table S1

Table S14. A Kendall tau correlation matrix for Siberian larch #1 (continued 1).

Weather par	orgP	alcP	hclP	orgS	alcS	hclS	orgTi	alcTi	hclTi	orgMn	alcMn	hclMn
**Iv_jn_ind**	0.05	-0.16	0.31	0.35	-0.38	0.09	-0.38	0.13	0.42	0.35	-0.02	0.20
**Iv_jl_ind**	-0.35	0.09	-0.38	-0.13	0.45	0.27	-0.20	-0.13	0.02	**-0.56**	-0.20	-0.35
**Iv_au_ind**	0.27	-0.24	-0.20	0.20	-0.16	0.09	0.13	-0.38	0.13	0.20	0.35	-0.31
**Iv-Vys_jn**	0.13	-0.09	0.31	0.35	-0.31	0.24	-0.31	0.05	**0.56**	0.42	-0.02	0.20
**Iv-Vys_jl**	-0.24	0.05	-0.27	-0.02	0.35	0.16	-0.16	-0.02	0.05	-0.38	-0.02	-0.24
**Iv-Vys_aug**	0.20	-0.09	-0.20	0.27	-0.16	0.02	0.13	-0.16	0.05	0.13	0.20	-0.24
**Hum_may**	0.27	-0.31	0.24	-0.09	-0.45	0.09	0.27	0.27	0.05	0.20	0.35	0.13
**Hum_jn**	0.26	0.00	0.26	0.37	-0.07	0.07	-0.15	-0.04	0.33	0.29	-0.04	0.26
**Hum_jl**	-0.05	0.02	-0.16	0.09	0.02	0.42	-0.42	0.16	0.45	-0.20	0.09	-0.20
**Hum_aug**	0.05	-0.24	-0.20	-0.09	-0.02	0.31	0.20	-0.16	0.13	-0.02	0.20	-0.24
**T_may**	0.24	0.09	-0.09	0.02	0.31	0.05	0.02	0.02	0.02	0.02	-0.13	0.16
**T_jn**	-0.02	0.20	-0.27	-0.16	0.05	0.31	-0.09	0.20	0.20	-0.31	-0.24	-0.16
**T_jl**	0.38	-0.20	0.35	0.16	-0.35	-0.24	0.24	0.02	-0.05	**0.53**	0.16	0.31
**T_aug**	-0.16	-0.09	-0.20	0.05	0.05	-0.13	-0.09	-0.38	-0.02	-0.02	-0.02	-0.31
**Prec_jn**	0.09	0.02	0.27	0.38	-0.27	0.27	-0.35	0.02	**0.60**	0.38	-0.05	0.16
**Prec_jl**	-0.16	0.05	-0.27	-0.02	0.42	0.02	-0.02	-0.02	-0.09	-0.31	-0.02	-0.16
**Prec_aug**	0.27	-0.16	-0.05	0.35	-0.31	-0.20	0.20	-0.16	-0.02	0.27	0.27	-0.09
**Wind_my**	0.24	0.06	0.13	0.09	-0.06	-0.24	0.28	-0.02	-0.13	0.31	0.13	0.17
**Wind_jn**	0.11	-0.22	0.07	0.18	0.07	-0.18	0.04	-0.33	0.00	0.33	0.18	0.11
**Wind_jl**	-0.09	-0.13	0.06	0.09	0.31	**-0.50**	0.09	-0.20	**-0.46**	-0.09	-0.06	0.09
**Wind_aug**	0.15	-0.11	0.26	0.04	0.11	**-0.52**	0.30	-0.07	-0.41	0.22	0.07	0.30

Table S14. A Kendall tau correlation matrix for Siberian larch #1 (continued 2).

Weather par	orgFe	alcFe	hclFe	orgCu	alcCu	hclCu	orgZn	alcZn	hclZn
**Iv_jn_ind**	-0.05	0.31	0.45	0.45	-0.05	**-0.67**	-0.02	-0.02	0.13
**Iv_jl_ind**	-0.16	0.05	-0.09	0.05	-0.24	0.38	-0.05	-0.35	0.02
**Iv_au_ind**	**0.53**	-0.13	0.24	-0.27	0.24	-0.02	-0.16	-0.02	**-0.67**
**Iv-Vys_jn**	0.02	0.16	**0.53**	0.38	0.02	**-0.60**	0.05	-0.09	-0.02
**Iv-Vys_jl**	-0.13	0.16	-0.05	0.09	-0.13	0.27	-0.09	-0.31	0.05
**Iv-Vys_aug**	0.38	-0.05	0.38	-0.27	0.02	-0.09	-0.16	0.05	**-0.53**
**Hum_may**	0.02	0.24	0.16	-0.13	0.45	-0.24	**0.49**	0.27	0.20
**Hum_jn**	0.04	0.00	**0.62**	0.37	0.07	-0.40	0.15	0.00	-0.11
**Hum_jl**	-0.16	0.42	0.35	0.35	-0.02	-0.05	-0.13	**-0.71**	-0.05
**Hum_aug**	0.02	0.24	0.24	-0.42	0.09	0.20	0.27	-0.02	-0.24
**T_may**	-0.09	-0.31	0.13	0.35	0.05	0.09	0.02	-0.05	0.02
**T_jn**	-0.20	-0.05	0.24	0.02	-0.05	0.13	0.13	-0.31	-0.02
**T_jl**	0.13	-0.16	0.13	-0.09	0.13	-0.35	0.09	0.31	-0.13
**T_aug**	**0.53**	**-0.56**	-0.05	0.24	0.24	0.13	-0.38	**0.49**	-0.31
**Prec_jn**	-0.09	0.20	**0.64**	0.42	-0.02	**-0.56**	0.02	-0.20	-0.13
**Prec_jl**	-0.13	0.24	-0.05	-0.05	-0.20	0.27	-0.09	-0.31	0.05
**Prec_aug**	**0.53**	-0.13	0.24	-0.27	0.02	-0.24	-0.24	0.20	-0.45
**Wind_my**	0.17	-0.06	-0.17	-0.06	0.13	-0.06	0.13	0.17	-0.02
**Wind_jn**	0.26	0.00	-0.07	-0.04	-0.04	-0.07	0.04	0.11	-0.04
**Wind_jl**	0.06	-0.13	-0.28	-0.02	-0.35	0.13	-0.06	0.28	0.24
**Wind_aug**	0.04	0.00	-0.30	-0.15	-0.30	-0.04	-0.07	0.26	0.04

Table S15. A Spearman rho correlation matrix for Siberian larch #2.

Weather par	orgCa*	alcCa	hclCa	orgSr	alcSr	hclSr	orgK	alcK	hclK	orgAl	alcAl	hclAl
**Iv_jn_ind****	0.51	-0.02	-0.35	0.21	-0.06	**-0.63**	0.37	0.15	0.04	-0.41	0.31	-0.01
**Iv_jl_ind**	0.32	0.07	0.23	0.21	0.46	0.00	**0.57**	-0.29	-0.06	-0.10	-0.07	0.40
**Iv_au_ind**	-0.31	-0.34	0.23	-0.31	-0.17	0.31	-0.03	-0.15	-0.05	0.13	0.06	0.46
**Iv-Vys_jn**	0.34	-0.05	-0.34	0.09	-0.23	-0.55	0.10	0.21	0.08	-0.31	0.25	-0.10
**Iv-Vys_jl**	0.35	0.06	0.13	0.21	0.43	-0.02	**0.56**	-0.33	0.07	-0.14	-0.12	0.31
**Iv-Vys_aug**	-0.15	-0.23	0.23	-0.21	-0.13	0.09	0.09	-0.26	-0.06	-0.07	-0.13	0.26
**Hum_may**	0.09	0.20	-0.27	0.30	0.12	0.10	0.04	-0.30	-0.10	0.10	-0.30	-0.15
**Hum_jn**	0.33	-0.09	-0.07	-0.02	-0.40	**-0.64**	0.07	0.37	0.11	-0.48	0.14	-0.40
**Hum_jl**	0.34	-0.09	0.08	0.03	0.03	0.03	0.43	-0.23	0.12	-0.14	-0.22	0.15
**Hum_aug**	0.26	0.16	-0.08	0.26	0.25	-0.02	0.33	-0.48	0.11	-0.15	-0.33	0.08
**T_may**	-0.41	-0.43	0.55	**-0.62**	-0.47	0.04	-0.33	0.14	0.17	-0.07	-0.05	-0.10
**T_jn**	0.18	-0.10	**0.60**	-0.08	-0.15	-0.05	0.39	0.23	0.08	-0.18	0.01	-0.01
**T_jl**	-0.05	0.23	-0.24	0.08	-0.17	-0.21	-0.23	0.26	0.22	0.05	-0.07	-0.29
**T_aug**	**-0.79**†	-0.53	-0.18	-0.53	**-0.60**	0.38	**-0.84**	-0.15	-0.33	0.34	-0.04	0.03
**Prec_jn**	0.36	-0.02	-0.27	0.08	-0.21	-0.51	0.19	0.25	0.10	-0.26	0.24	-0.03
**Prec_jl**	0.24	0.13	0.17	0.24	**0.58**	0.09	0.53	-0.34	-0.08	-0.10	-0.20	0.42
**Prec_aug**	-0.38	-0.37	0.12	-0.41	-0.39	0.18	-0.21	-0.08	-0.11	0.05	-0.01	0.25
**Wind_my**	-0.21	0.09	-0.30	0.11	0.10	0.05	-0.21	0.04	0.07	0.03	-0.08	0.18
**Wind_jn**	-0.32	-0.03	-0.34	-0.08	0.00	-0.12	-0.47	-0.16	-0.21	-0.12	-0.09	0.04
**Wind_jl**	-0.23	-0.04	-0.02	-0.23	0.23	0.21	-0.27	-0.31	-0.25	0.18	0.02	0.06
**Wind_aug**	-0.25	0.12	-0.51	-0.12	0.23	-0.10	-0.38	-0.34	-0.12	0.22	0.18	-0.17

* as in Table S1

** as in Table S1

† as in Table S1

Table S15. A Spearman rho correlation matrix for Siberian larch #2 (continued 1).

Weather par	orgP	alcP	hclP	orgS	alcS	hclS	orgTi	alcTi	hclTi	orgMn	alcMn	hclMn
**Iv_jn_ind**	-0.07	-0.20	-0.29	0.36	-0.31	0.04	-0.09	0.37	-0.07	0.24	0.08	0.01
**Iv_jl_ind**	-0.12	-0.29	-0.12	-0.40	-0.38	-0.50	-0.09	**0.69**	0.31	0.40	0.12	-0.43
**Iv_au_ind**	0.14	0.24	-0.01	**-0.69**	0.32	0.08	0.16	-0.01	0.10	-0.29	-0.26	0.27
**Iv-Vys_jn**	0.01	-0.15	-0.23	0.18	-0.30	0.03	-0.24	0.19	-0.14	0.03	-0.01	-0.02
**Iv-Vys_jl**	-0.03	-0.35	-0.23	-0.31	-0.46	-0.33	-0.02	**0.59**	0.36	0.44	0.15	-0.45
**Iv-Vys_aug**	-0.12	0.13	-0.13	**-0.56**	0.16	-0.17	0.23	0.01	-0.05	-0.21	-0.14	0.17
**Hum_may**	0.00	-0.25	-0.07	0.31	-0.31	-0.45	0.01	-0.22	-0.16	0.10	0.35	0.01
**Hum_jn**	-0.26	-0.12	-0.38	0.30	-0.23	0.19	0.06	0.09	-0.03	-0.06	-0.09	-0.04
**Hum_jl**	0.04	-0.30	-0.32	-0.43	**-0.57**	-0.28	0.16	0.34	0.33	0.19	0.04	-0.35
**Hum_aug**	-0.06	-0.48	-0.38	-0.34	-0.54	-0.39	0.17	0.15	0.11	0.29	0.36	-0.35
**T_may**	-0.13	0.32	-0.15	**-0.62**	0.30	0.31	0.03	-0.13	0.17	-0.51	**-0.58**	-0.01
**T_jn**	-0.37	0.01	-0.24	-0.28	0.07	0.03	0.44	0.21	0.36	0.00	-0.07	-0.08
**T_jl**	0.01	0.38	0.46	0.40	0.33	-0.12	-0.40	-0.49	**-0.72**	-0.21	0.07	0.30
**T_aug**	0.34	0.39	0.08	-0.11	0.39	0.08	-0.24	**-0.60**	-0.25	**-0.81**	-0.49	**0.62**
**Prec_jn**	-0.02	0.00	-0.05	0.10	-0.19	-0.09	-0.26	0.20	-0.25	0.02	-0.03	0.04
**Prec_jl**	-0.05	-0.18	0.05	-0.19	-0.22	-0.41	-0.03	**0.59**	0.23	0.43	0.15	-0.33
**Prec_aug**	0.05	0.42	0.07	-0.49	0.47	0.08	0.20	-0.25	-0.23	-0.49	-0.33	0.49
**Wind_my**	0.34	0.28	0.41	0.28	0.37	0.15	-0.35	-0.25	-0.38	-0.10	0.05	0.33
**Wind_jn**	0.10	0.10	0.16	0.08	0.17	0.00	-0.31	-0.14	-0.44	-0.22	-0.09	0.17
**Wind_jl**	-0.02	-0.02	0.10	-0.27	-0.03	-0.05	0.02	0.12	0.02	0.05	-0.16	-0.22
**Wind_aug**	-0.06	0.29	0.35	0.36	0.35	0.03	-0.12	-0.33	**-0.71**	0.05	-0.02	0.26

Table S15. A Spearman rho correlation matrix for Siberian larch #2 (continued 2).

Weather par	orgFe	alcFe	hclFe	orgCu	alcCu	hclCu	orgZn	alcZn	hclZn
**Iv_jn_ind**	-0.04	0.03	0.19	0.15	**0.62**	-0.41	0.17	-0.44	0.23
**Iv_jl_ind**	0.25	-0.12	-0.01	-0.12	0.05	0.19	-0.03	-0.36	0.40
**Iv_au_ind**	-0.03	-0.27	-0.23	0.13	-0.24	0.52	0.13	0.32	-0.01
**Iv-Vys_jn**	-0.20	0.20	0.12	0.03	**0.60**	-0.50	0.10	-0.33	0.15
**Iv-Vys_jl**	0.30	-0.14	0.05	-0.12	0.07	0.10	0.03	-0.44	0.49
**Iv-Vys_aug**	0.01	-0.13	-0.10	0.19	-0.02	0.30	0.00	0.05	0.04
**Hum_may**	0.31	0.26	-0.21	0.01	-0.17	0.11	-0.25	0.08	-0.02
**Hum_jn**	-0.33	0.10	0.39	0.25	**0.64**	**-0.65**	0.10	-0.37	0.12
**Hum_jl**	0.15	-0.07	0.07	0.09	0.05	0.03	0.07	-0.29	0.45
**Hum_aug**	0.49	0.23	-0.10	-0.22	-0.01	0.21	0.08	-0.19	0.53
**T_may**	**-0.59**	-0.26	0.23	0.18	0.10	-0.25	-0.09	-0.12	-0.05
**T_jn**	0.01	-0.27	0.29	0.42	-0.10	0.21	0.29	0.12	0.17
**T_jl**	-0.23	0.36	0.06	0.16	0.25	-0.48	-0.12	0.02	-0.51
**T_aug**	**-0.56**	-0.09	-0.45	0.15	-0.15	0.04	**-0.59**	0.38	**-0.59**
**Prec_jn**	-0.21	0.19	0.13	0.14	**0.57**	-0.46	0.13	-0.29	0.04
**Prec_jl**	0.29	-0.20	0.02	-0.09	0.03	0.16	-0.11	-0.39	0.23
**Prec_aug**	-0.23	-0.17	-0.17	0.29	-0.05	0.28	-0.01	0.29	-0.27
**Wind_my**	-0.08	0.05	-0.12	-0.05	0.16	-0.17	-0.12	0.05	-0.42
**Wind_jn**	-0.23	0.23	-0.17	-0.33	0.40	-0.32	-0.36	-0.21	-0.28
**Wind_jl**	0.01	0.00	-0.15	-0.46	-0.15	0.13	-0.20	-0.16	0.09
**Wind_aug**	0.05	0.16	-0.15	-0.31	0.05	-0.12	-0.23	-0.28	-0.24

Table S16. A Kendall tau correlation matrix for Siberian larch #2.

Weather par	orgCa*	alcCa	hclCa	orgSr	alcSr	hclSr	orgK	alcK	hclK	orgAl	alcAl	hclAl
**Iv_jn_ind****	0.41	-0.03	-0.31	0.18	0.00	-0.41	0.28	0.10	0.03	-0.28	0.21	0.00
**Iv_jl_ind**	0.23	0.10	0.18	0.15	0.28	0.03	**0.46**	-0.23	0.00	-0.05	-0.08	0.28
**Iv_au_ind**	-0.23	-0.26	0.13	-0.21	-0.18	0.28	0.00	-0.13	0.00	0.10	0.03	0.28
**Iv-Vys_jn**	0.23	0.00	-0.28	0.05	-0.18	-0.38	0.05	0.03	0.05	-0.15	0.18	-0.08
**Iv-Vys_jl**	0.33	0.05	0.13	0.15	0.33	0.03	**0.46**	-0.23	0.05	-0.10	-0.13	0.28
**Iv-Vys_aug**	-0.13	-0.15	0.13	-0.10	-0.13	0.03	0.05	-0.23	-0.05	-0.10	-0.13	0.23
**Hum_may**	0.08	0.15	-0.18	0.21	0.08	0.08	0.05	-0.18	-0.10	0.05	-0.18	-0.13
**Hum_jn**	0.23	-0.10	-0.08	-0.05	-0.33	**-0.49**	0.05	0.23	0.05	-0.26	0.08	-0.23
**Hum_jl**	0.21	-0.03	0.05	0.03	0.00	0.10	0.33	-0.15	0.08	-0.13	-0.15	0.15
**Hum_aug**	0.22	0.09	-0.12	0.19	0.12	0.01	0.25	-0.40	0.09	-0.12	-0.25	0.01
**T_may**	-0.33	-0.31	0.38	**-0.46**	-0.33	0.03	-0.26	0.13	0.10	-0.05	-0.03	-0.08
**T_jn**	0.13	-0.05	0.33	0.00	-0.13	-0.03	0.26	0.13	0.05	-0.21	-0.03	-0.03
**T_jl**	-0.05	0.18	-0.15	0.03	-0.15	-0.21	-0.18	0.21	0.13	0.03	-0.05	-0.21
**T_aug**	**-0.59†**	-0.41	-0.08	-0.41	**-0.49**	0.23	**-0.67**	-0.08	-0.21	0.26	0.03	0.03
**Prec_jn**	0.26	-0.03	-0.21	-0.03	-0.21	-0.31	0.08	0.10	0.03	-0.13	0.15	0.00
**Prec_jl**	0.18	0.10	0.13	0.15	**0.44**	0.08	**0.46**	-0.28	-0.10	-0.05	-0.13	0.33
**Prec_aug**	-0.28	-0.26	0.03	-0.26	-0.28	0.13	-0.15	-0.08	-0.05	0.05	-0.03	0.13
**Wind_my**	-0.13	0.05	-0.18	0.05	0.08	0.05	-0.16	0.05	0.08	0.05	-0.08	0.10
**Wind_jn**	-0.17	-0.04	-0.14	-0.04	0.14	-0.12	-0.25	-0.04	-0.19	-0.14	-0.09	0.06
**Wind_jl**	-0.17	-0.04	-0.01	-0.19	0.09	0.12	-0.19	-0.32	-0.25	0.09	0.01	0.01
**Wind_aug**	-0.19	0.14	-0.35	-0.06	0.17	-0.06	-0.22	-0.32	-0.06	0.17	0.09	-0.12

* as in Table S1

** as in Table S1

† as in Table S1

Table S16. A Kendall tau correlation matrix for Siberian larch #2 (continued 1).

Weather par	orgP	alcP	hclP	orgS	alcS	hclS	orgTi	alcTi	hclTi	orgMn	alcMn	hclMn
**Iv_jn_ind**	-0.03	-0.08	-0.18	0.33	-0.15	-0.01	-0.10	0.26	-0.05	0.23	0.10	-0.03
**Iv_jl_ind**	-0.10	-0.21	-0.05	-0.26	-0.23	-0.41	-0.13	**0.54**	0.23	0.26	0.08	-0.31
**Iv_au_ind**	0.10	0.15	0.00	**-0.56**	0.18	0.04	0.08	-0.03	0.08	-0.21	-0.18	0.21
**Iv-Vys_jn**	0.00	-0.05	-0.10	0.15	-0.23	0.01	-0.18	0.13	-0.13	0.00	-0.03	-0.05
**Iv-Vys_jl**	-0.05	-0.21	-0.15	-0.26	-0.28	-0.25	-0.03	**0.44**	0.28	0.36	0.08	-0.31
**Iv-Vys_aug**	-0.05	0.10	-0.10	-0.41	0.08	-0.17	0.13	-0.03	-0.03	-0.15	-0.08	0.15
**Hum_may**	-0.05	-0.15	-0.05	0.26	-0.18	-0.35	0.03	-0.08	-0.08	0.05	0.28	0.05
**Hum_jn**	-0.15	-0.05	-0.26	0.26	-0.13	0.17	0.03	0.03	-0.03	-0.05	-0.13	-0.05
**Hum_jl**	0.08	-0.23	-0.23	-0.38	**-0.46**	-0.25	0.05	0.21	0.26	0.13	0.05	-0.23
**Hum_aug**	-0.01	-0.35	-0.27	-0.25	-0.37	-0.29	0.14	0.12	0.12	0.19	0.22	-0.30
**T_may**	-0.10	0.26	-0.10	-0.41	0.28	0.20	0.03	-0.03	0.18	-0.41	**-0.44**	0.00
**T_jn**	-0.15	0.05	-0.15	-0.21	0.08	0.01	0.33	0.18	0.28	-0.05	-0.03	-0.05
**T_jl**	-0.03	0.28	0.33	0.28	0.21	-0.09	-0.36	-0.36	**-0.51**	-0.13	0.05	0.28
**T_aug**	0.26	0.26	0.05	-0.05	0.33	0.04	-0.18	**-0.44**	-0.18	**-0.62**	-0.38	**0.46**
**Prec_jn**	0.03	0.08	0.03	0.08	-0.10	-0.12	-0.21	0.15	-0.21	-0.03	-0.05	0.08
**Prec_jl**	-0.05	-0.10	0.05	-0.10	-0.13	-0.30	-0.08	**0.44**	0.18	0.31	0.08	-0.21
**Prec_aug**	0.05	0.36	0.05	-0.31	0.33	0.04	0.08	-0.13	-0.13	-0.36	-0.23	0.36
**Wind_my**	0.29	0.29	0.34	0.13	0.31	0.12	-0.21	-0.18	-0.26	-0.10	0.03	0.26
**Wind_jn**	0.04	0.09	0.12	0.06	0.12	0.03	-0.19	-0.09	-0.30	-0.09	-0.06	0.14
**Wind_jl**	0.04	-0.01	0.09	-0.25	0.01	-0.05	0.06	0.04	-0.01	0.01	-0.12	-0.17
**Wind_aug**	-0.04	0.22	0.27	0.27	0.25	0.03	-0.09	-0.25	**-0.55**	0.04	-0.04	0.06

Table S16. A Kendall tau correlation matrix for Siberian larch #2 (continued 2).

Weather par	orgFe	alcFe	hclFe	orgCu	alcCu	hclCu	orgZn	alcZn	hclZn
**Iv_jn_ind**	-0.03	0.05	0.13	0.10	**0.44**	-0.33	0.08	-0.33	0.21
**Iv_jl_ind**	0.15	-0.08	0.00	-0.08	0.00	0.15	0.00	-0.26	0.28
**Iv_au_ind**	0.00	-0.18	-0.15	0.08	-0.21	0.41	0.10	0.26	-0.03
**Iv-Vys_jn**	-0.15	0.13	0.10	0.03	**0.46**	-0.36	0.10	-0.26	0.08
**Iv-Vys_jl**	0.15	-0.03	0.05	-0.08	0.05	0.05	0.00	-0.31	0.33
**Iv-Vys_aug**	0.00	-0.13	-0.05	0.13	0.00	0.26	0.00	0.00	0.08
**Hum_may**	0.15	0.18	-0.10	0.03	0.00	0.10	-0.15	0.05	-0.08
**Hum_jn**	-0.26	0.03	0.31	0.18	**0.46**	-0.41	0.05	-0.26	0.08
**Hum_jl**	0.08	0.00	0.03	0.05	0.03	0.03	0.08	-0.18	0.36
**Hum_aug**	0.35	0.12	-0.09	-0.12	-0.04	0.14	0.04	-0.17	**0.43**
**T_may**	**-0.46**	-0.18	0.15	0.13	0.15	-0.21	-0.05	-0.10	-0.08
**T_jn**	0.00	-0.18	0.26	0.38	-0.05	0.10	0.26	0.15	0.08
**T_jl**	-0.18	0.26	0.03	0.05	0.13	-0.33	-0.08	0.03	-0.36
**T_aug**	-0.41	-0.08	-0.36	0.18	-0.05	0.05	**-0.46**	0.26	-0.33
**Prec_jn**	-0.23	0.15	0.08	0.10	0.38	-0.33	0.13	-0.13	0.00
**Prec_jl**	0.21	-0.08	0.05	-0.13	0.00	0.15	-0.05	-0.26	0.13
**Prec_aug**	-0.15	-0.08	-0.10	0.18	0.00	0.26	0.00	0.21	-0.23
**Wind_my**	-0.08	0.00	-0.08	-0.03	0.13	-0.13	-0.05	0.08	-0.36
**Wind_jn**	-0.14	0.14	-0.12	-0.25	0.30	-0.22	-0.27	-0.17	-0.25
**Wind_jl**	0.01	-0.01	-0.09	-0.30	-0.09	0.09	-0.12	-0.12	0.09
**Wind_aug**	0.04	0.06	-0.12	-0.22	0.01	-0.09	-0.17	-0.25	-0.14

Table S17. A Spearman rho correlation matrix for Siberian larch #3.

Weather par	orgCa*	alcCa	hclCa	orgSr	alcSr	hclSr	orgK	alcK	hclK	orgAl	alcAl	hclAl
**Iv_jn_ind****	-0.30	-0.31	0.08	-0.21	-0.22	0.25	0.01	-0.38	0.41	0.09	-0.10	-0.42
**Iv_jl_ind**	0.06	0.05	-0.51	-0.21	-0.53	**-0.57**	-0.20	0.20	-0.13	**0.57**	0.45	0.04
**Iv_au_ind**	0.38	0.32	0.28	0.12	0.25	-0.18	0.02	0.29	-0.25	0.52	0.19	0.10
**Iv-Vys_jn**	-0.28	-0.35	0.01	-0.27	-0.07	0.07	0.05	-0.32	0.48	0.10	0.04	-0.43
**Iv-Vys_jl**	0.06	-0.04	-0.54	-0.13	**-0.57†**	-0.37	-0.25	0.18	-0.13	0.51	0.37	0.03
**Iv-Vys_aug**	0.20	0.23	0.18	-0.14	0.10	-0.14	0.05	0.20	0.01	0.38	0.21	-0.09
**Hum_may**	-0.01	0.25	-0.02	0.24	0.08	0.33	0.23	-0.20	0.02	-0.14	**-0.60**	-0.01
**Hum_jn**	-0.50	-0.47	0.10	-0.45	-0.18	0.34	-0.05	-0.26	**0.58**	-0.16	0.05	**-0.57**
**Hum_jl**	-0.09	-0.03	-0.36	-0.17	-0.43	-0.10	-0.07	0.21	0.03	0.49	0.23	-0.14
**Hum_aug**	0.36	0.27	-0.43	0.04	-0.24	-0.15	-0.10	-0.07	0.15	0.44	0.12	-0.23
**T_may**	-0.22	-0.37	0.01	-0.45	-0.02	-0.23	-0.19	**0.55**	0.03	0.14	**0.66**	-0.04
**T_jn**	-0.15	0.08	0.17	-0.21	-0.23	0.21	-0.32	0.07	0.04	0.21	0.03	-0.32
**T_jl**	-0.37	-0.35	0.25	-0.23	0.38	0.13	0.14	-0.39	0.39	-0.48	-0.36	-0.14
**T_aug**	-0.04	0.05	0.53	0.23	**0.68**	0.19	**0.55**	0.38	-0.27	-0.11	-0.25	0.40
**Prec_jn**	-0.35	-0.34	0.07	-0.37	-0.04	-0.02	0.08	-0.31	0.49	0.12	0.04	-0.42
**Prec_jl**	0.08	-0.02	-0.46	-0.15	-0.51	-0.51	-0.16	0.20	-0.27	0.33	0.39	0.25
**Prec_aug**	0.18	0.21	0.51	-0.05	0.43	-0.02	0.24	0.20	-0.03	0.21	0.09	0.02
**Wind_my**	0.10	-0.26	0.16	0.13	0.33	-0.06	0.07	-0.21	-0.11	-0.22	-0.12	0.28
**Wind_jn**	0.19	-0.16	-0.06	-0.15	0.25	-0.46	0.30	-0.01	0.12	-0.19	0.43	0.18
**Wind_jl**	0.27	0.22	-0.36	-0.02	-0.11	**-0.61**	0.20	0.43	-0.20	-0.01	**0.59**	0.36
**Wind_aug**	0.12	0.12	0.09	0.06	0.27	-0.12	0.43	-0.02	0.11	-0.55	-0.04	0.26

* as in Table S1

** as in Table S1

† as in Table S1

Table S17. A Spearman rho correlation matrix for Siberian larch #3 (continued 1).

Weather par	orgP	alcP	hclP	orgS	alcS	hclS	orgTi	alcTi	hclTi	orgMn	alcMn	hclMn
**Iv_jn_ind**	-0.31	0.40	0.30	-0.33	0.36	0.01	**0.55**	0.08	-0.13	-0.29	-0.31	-0.27
**Iv_jl_ind**	0.07	0.09	0.10	0.01	0.29	0.40	0.19	-0.32	0.10	0.03	-0.07	-0.16
**Iv_au_ind**	-0.08	**-0.73**	-0.43	0.35	0.20	-0.16	-0.12	-0.20	0.35	0.25	0.17	0.24
**Iv-Vys_jn**	-0.26	0.45	0.30	-0.40	0.27	0.02	0.48	0.04	-0.26	-0.34	-0.31	-0.33
**Iv-Vys_jl**	0.18	0.13	0.19	0.05	0.30	0.21	0.33	-0.23	0.06	0.03	-0.05	-0.06
**Iv-Vys_aug**	-0.09	**-0.62**	-0.25	0.17	-0.02	-0.12	0.00	-0.15	0.38	0.00	-0.09	0.27
**Hum_may**	0.32	-0.13	0.29	0.14	**-0.56**	0.08	-0.05	0.54	0.03	0.11	0.12	0.54
**Hum_jn**	-0.34	0.47	0.14	-0.34	0.05	-0.22	0.28	-0.04	-0.10	**-0.58**	-0.53	-0.34
**Hum_jl**	0.26	0.02	0.02	0.18	-0.03	-0.01	0.26	0.04	-0.14	-0.05	-0.05	0.16
**Hum_aug**	0.12	-0.19	0.35	0.16	0.09	-0.10	0.28	-0.08	0.12	-0.01	-0.08	0.35
**T_may**	0.01	-0.09	-0.38	0.00	-0.07	-0.24	-0.16	-0.42	0.20	-0.31	-0.28	-0.24
**T_jn**	-0.30	-0.37	-0.39	0.26	-0.11	-0.28	-0.31	-0.23	0.49	-0.21	-0.27	0.01
**T_jl**	-0.03	0.13	0.14	**-0.63**	-0.47	0.12	0.25	0.54	-0.14	-0.27	-0.21	-0.08
**T_aug**	0.40	-0.37	-0.27	0.23	-0.49	0.01	-0.30	0.38	0.03	0.26	0.32	0.42
**Prec_jn**	-0.29	0.36	0.18	-0.47	0.16	0.12	0.47	0.12	-0.27	-0.35	-0.32	-0.35
**Prec_jl**	0.18	0.12	0.06	-0.05	0.29	0.41	0.30	-0.24	0.07	0.13	0.02	-0.11
**Prec_aug**	-0.13	**-0.70**	-0.47	0.15	-0.06	-0.19	-0.08	-0.02	0.25	0.09	0.04	0.27
**Wind_my**	0.10	0.10	0.07	-0.40	0.23	0.12	0.49	0.21	-0.05	0.14	0.12	-0.06
**Wind_jn**	-0.04	0.31	0.20	-0.45	0.35	0.24	0.36	-0.23	-0.24	-0.04	-0.05	-0.25
**Wind_jl**	0.11	0.17	0.01	0.10	0.18	0.26	-0.28	-0.42	-0.37	0.20	0.21	-0.16
**Wind_aug**	0.00	0.03	0.28	-0.27	-0.11	0.17	-0.01	0.15	-0.33	0.13	0.14	0.03

Table S17. A Spearman rho correlation matrix for Siberian larch #3 (continued 2).

Weather par	orgFe	alcFe	hclFe	orgCu	alcCu	hclCu	orgZn	alcZn	hclZn
**Iv_jn_ind**	-0.45	-0.15	0.05	0.05	-0.45	-0.23	0.21	0.04	-0.10
**Iv_jl_ind**	-0.13	-0.25	-0.27	0.15	0.20	-0.09	-0.32	0.27	-0.09
**Iv_au_ind**	0.22	-0.01	0.09	0.41	0.48	0.08	-0.16	0.06	0.27
**Iv-Vys_jn**	-0.33	-0.05	-0.08	0.00	-0.49	-0.23	0.31	0.03	-0.24
**Iv-Vys_jl**	-0.21	-0.38	-0.16	0.14	0.20	0.13	-0.35	0.21	-0.09
**Iv-Vys_aug**	-0.05	-0.19	0.01	0.18	0.24	-0.08	-0.03	0.05	0.24
**Hum_may**	-0.23	0.04	0.16	-0.22	-0.04	0.03	-0.26	-0.33	0.32
**Hum_jn**	-0.40	-0.11	0.17	-0.25	**-0.74**	-0.43	0.47	0.05	-0.14
**Hum_jl**	-0.49	-0.43	0.17	0.07	0.10	-0.05	-0.26	0.08	0.10
**Hum_aug**	-0.09	-0.23	-0.06	-0.11	0.15	0.33	-0.37	-0.17	0.13
**T_may**	0.01	-0.23	-0.08	0.08	-0.08	-0.18	0.34	0.40	-0.18
**T_jn**	-0.13	-0.14	0.42	-0.09	-0.10	-0.34	-0.11	-0.04	0.32
**T_jl**	-0.30	-0.20	-0.32	-0.14	-0.31	-0.04	0.40	-0.38	-0.42
**T_aug**	0.12	0.35	0.15	0.27	0.08	-0.23	0.21	0.14	0.30
**Prec_jn**	-0.45	-0.16	-0.14	0.05	-0.42	-0.32	0.34	0.00	-0.27
**Prec_jl**	-0.09	-0.35	-0.29	0.18	0.31	0.05	-0.29	0.22	-0.20
**Prec_aug**	0.03	-0.05	0.09	0.30	0.27	-0.13	0.16	0.02	0.24
**Wind_my**	0.22	-0.07	-0.31	0.24	0.09	0.35	0.12	-0.26	-0.48
**Wind_jn**	0.34	0.16	**-0.57**	0.05	-0.07	0.05	0.43	0.18	-0.50
**Wind_jl**	0.27	0.18	-0.38	0.00	0.35	0.06	0.09	0.54	-0.06
**Wind_aug**	-0.06	-0.03	-0.55	-0.02	0.25	0.30	0.40	0.29	-0.08

Table S18. A Kendall tau correlation matrix for Siberian larch #3.

Weather par	orgCa*	alcCa	hclCa	orgSr	alcSr	hclSr	orgK	alcK	hclK	orgAl	alcAl	hclAl
**Iv_jn_ind****	-0.18	-0.21	0.00	-0.15	-0.18	0.15	0.03	-0.31	0.26	0.10	-0.15	-0.31
**Iv_jl_ind**	0.00	0.03	-0.38	-0.18	-0.41	**-0.44**	-0.21	0.13	-0.08	0.38	0.33	0.03
**Iv_au_ind**	0.26	0.23	0.18	0.08	0.21	-0.13	0.00	0.13	-0.18	0.33	0.13	0.13
**Iv-Vys_jn**	-0.21	-0.23	-0.03	-0.23	0.00	0.03	0.05	-0.28	0.28	0.08	-0.03	-0.33
**Iv-Vys_jl**	0.05	-0.08	-0.38	-0.08	**-0.51**	-0.23	-0.21	0.18	-0.08	0.38	0.28	0.08
**Iv-Vys_aug**	0.15	0.18	0.13	-0.08	0.10	-0.13	0.00	0.13	-0.03	0.23	0.13	-0.08
**Hum_may**	0.05	0.18	0.03	0.18	0.05	0.23	0.21	-0.13	0.03	-0.08	-0.38	-0.03
**Hum_jn**	-0.36	-0.28	0.03	-0.28	-0.10	0.23	-0.05	-0.18	0.38	-0.08	-0.03	**-0.44**
**Hum_jl**	-0.03	-0.05	-0.26	-0.10	-0.28	0.00	-0.03	0.15	0.00	0.31	0.15	-0.05
**Hum_aug**	0.25	0.22	-0.32	0.06	-0.19	-0.12	-0.09	-0.06	0.09	0.32	0.06	-0.14
**T_may**	-0.15	-0.28	0.13	-0.33	0.00	-0.13	-0.05	**0.44**	0.03	0.13	**0.54**	-0.08
**T_jn**	-0.10	0.03	0.13	-0.18	-0.10	0.08	-0.21	0.08	0.08	0.13	0.03	-0.28
**T_jl**	-0.28	-0.26	0.21	-0.10	0.33	0.10	0.08	-0.26	0.26	-0.31	-0.26	-0.10
**T_aug**	-0.05	0.03	**0.44†**	0.08	**0.51**	0.18	0.36	0.28	-0.18	-0.08	-0.13	0.28
**Prec_jn**	-0.23	-0.21	0.10	-0.31	0.08	0.00	0.13	-0.21	0.31	0.10	0.00	-0.31
**Prec_jl**	0.05	-0.03	-0.33	-0.08	-0.41	-0.33	-0.10	0.13	-0.23	0.23	0.28	0.18
**Prec_aug**	0.10	0.13	0.38	-0.08	0.36	-0.08	0.15	0.08	-0.03	0.08	0.08	-0.03
**Wind_my**	0.08	-0.16	0.13	0.10	0.21	-0.03	0.10	-0.16	-0.05	-0.10	-0.13	0.26
**Wind_jn**	0.14	-0.09	-0.04	-0.01	0.14	-0.30	0.25	0.01	0.09	-0.14	0.27	0.14
**Wind_jl**	0.19	0.17	-0.30	0.01	-0.06	**-0.45**	0.14	0.32	-0.12	-0.01	0.35	0.22
**Wind_aug**	0.06	0.09	-0.01	0.04	0.17	-0.12	0.27	-0.04	0.01	**-0.43**	-0.04	0.19

* as in Table S1

** as in Table S1

† as in Table S1

Table S18. A Kendall tau correlation matrix for Siberian larch #3 (continued 1).

Weather par	orgP	alcP	hclP	orgS	alcS	hclS	orgTi	alcTi	hclTi	orgMn	alcMn	hclMn
**Iv_jn_ind**	-0.23	0.30	0.23	-0.21	0.28	-0.04	**0.44**	0.10	-0.13	-0.18	-0.21	-0.18
**Iv_jl_ind**	0.05	0.01	0.05	0.03	0.21	0.25	0.15	-0.23	0.10	0.00	-0.08	-0.10
**Iv_au_ind**	0.00	**-0.55**	-0.31	0.28	0.10	-0.04	-0.10	-0.13	0.26	0.21	0.13	0.21
**Iv-Vys_jn**	-0.15	0.32	0.21	-0.23	0.15	-0.01	0.31	0.08	-0.21	-0.21	-0.23	-0.15
**Iv-Vys_jl**	0.10	0.12	0.10	0.08	0.21	0.14	0.21	-0.18	0.10	0.00	-0.08	-0.05
**Iv-Vys_aug**	0.00	-0.40	-0.21	0.18	-0.05	-0.04	-0.05	-0.13	0.26	0.05	-0.03	0.26
**Hum_may**	0.21	-0.12	0.21	0.08	**-0.46**	0.09	-0.05	**0.44**	0.05	0.10	0.08	0.36
**Hum_jn**	-0.26	0.32	0.10	-0.18	-0.05	-0.14	0.21	-0.03	-0.05	-0.36	-0.33	-0.21
**Hum_jl**	0.23	0.04	0.08	0.15	-0.03	0.01	0.18	-0.05	-0.08	-0.03	0.00	0.18
**Hum_aug**	0.12	-0.10	0.19	0.14	0.09	-0.11	0.30	-0.06	0.06	-0.01	-0.04	0.27
**T_may**	0.00	-0.04	-0.31	-0.03	0.00	-0.12	-0.10	-0.28	0.15	-0.21	-0.18	-0.21
**T_jn**	-0.21	-0.25	-0.36	0.23	-0.05	-0.14	-0.26	-0.13	0.36	-0.21	-0.23	-0.05
**T_jl**	-0.03	0.06	0.13	**-0.46**	-0.33	0.14	0.18	0.36	-0.08	-0.18	-0.10	-0.08
**T_aug**	0.26	-0.27	-0.26	0.13	-0.31	0.01	-0.21	0.28	0.00	0.21	0.23	0.26
**Prec_jn**	-0.18	0.19	0.13	-0.31	0.08	0.12	0.28	0.15	-0.18	-0.18	-0.21	-0.23
**Prec_jl**	0.15	0.06	0.05	-0.03	0.15	0.33	0.21	-0.18	0.05	0.05	-0.03	-0.05
**Prec_aug**	-0.10	**-0.55**	-0.36	0.13	-0.05	-0.14	-0.10	-0.03	0.15	0.05	-0.03	0.26
**Wind_my**	0.03	0.04	0.03	-0.26	0.18	0.09	0.36	0.13	0.00	0.08	0.05	-0.08
**Wind_jn**	-0.04	0.23	0.17	-0.30	0.25	0.21	0.27	-0.17	-0.19	-0.01	0.01	-0.19
**Wind_jl**	0.06	0.10	0.01	0.06	0.09	0.21	-0.19	-0.32	-0.27	0.14	0.17	-0.12
**Wind_aug**	0.14	0.03	0.25	-0.22	-0.09	0.11	0.01	0.14	-0.27	0.12	0.14	0.06

Table S18. A Kendall tau correlation matrix for Siberian larch #3 (continued 2).

Weather par	orgFe	alcFe	hclFe	orgCu	alcCu	hclCu	orgZn	alcZn	hclZn
**Iv_jn_ind**	-0.33	-0.13	0.03	0.05	-0.31	-0.26	0.15	0.00	-0.05
**Iv_jl_ind**	-0.10	-0.21	-0.21	0.08	0.13	-0.03	-0.23	0.18	-0.08
**Iv_au_ind**	0.21	0.00	0.05	0.28	0.33	0.03	-0.13	0.08	0.23
**Iv-Vys_jn**	-0.21	0.00	-0.05	-0.03	-0.38	-0.23	0.23	0.03	-0.13
**Iv-Vys_jl**	-0.15	-0.31	-0.21	0.08	0.18	0.08	-0.23	0.08	-0.08
**Iv-Vys_aug**	-0.05	-0.10	0.00	0.13	0.18	-0.13	-0.03	0.03	0.18
**Hum_may**	-0.15	0.05	0.10	-0.18	-0.03	0.08	-0.18	-0.23	0.28
**Hum_jn**	-0.26	-0.05	0.15	-0.13	**-0.54**	-0.28	0.28	0.03	-0.08
**Hum_jl**	-0.33	-0.33	0.13	0.00	0.10	-0.05	-0.15	0.05	0.10
**Hum_aug**	-0.12	-0.17	-0.04	-0.04	0.12	0.22	-0.27	-0.12	0.09
**T_may**	-0.05	-0.21	-0.10	0.08	-0.08	-0.08	0.23	0.28	-0.13
**T_jn**	-0.10	-0.15	0.31	-0.03	-0.08	-0.28	-0.08	-0.03	0.28
**T_jl**	-0.23	-0.13	-0.23	-0.10	-0.21	0.00	0.21	-0.31	-0.36
**T_aug**	0.05	0.26	0.10	0.18	0.13	-0.18	0.18	0.08	0.18
**Prec_jn**	-0.33	-0.08	-0.08	0.05	-0.26	-0.26	0.26	0.00	-0.15
**Prec_jl**	-0.05	-0.26	-0.21	0.13	0.23	0.03	-0.28	0.13	-0.18
**Prec_aug**	0.00	-0.05	0.05	0.18	0.18	-0.18	0.08	0.03	0.23
**Wind_my**	0.13	-0.03	-0.18	0.21	0.05	0.29	0.16	-0.18	-0.31
**Wind_jn**	0.22	0.09	**-0.43**	0.06	-0.04	0.04	0.32	0.12	-0.35
**Wind_jl**	0.25	0.19	-0.22	0.01	0.27	0.06	0.12	0.37	-0.04
**Wind_aug**	-0.04	-0.04	-0.40	-0.01	0.14	0.22	0.30	0.22	-0.06

Table S19. A Spearman rho correlation matrix for Scots pine #1.

Weather par	orgCa*	alcCa	hclCa	orgSr	alcSr	hclSr	orgK	alcK	hclK	orgAl	alcAl	hclAl
**Iv_jn_ind****	0.42	0.25	-0.02	-0.39	-0.25	**-0.62**	-0.28	-0.12	-0.24	-0.09	-0.15	0.23
**Iv_jl_ind**	-0.42	-0.47	-0.29	-0.24	-0.05	-0.15	-0.11	-0.03	-0.13	0.45	0.14	-0.02
**Iv_au_ind**	-0.47	-0.15	-0.04	0.27	0.29	-0.25	0.19	0.27	0.10	0.29	-0.11	-0.12
**Iv-Vys_jn**	0.47	0.30	-0.07	-0.36	-0.08	**-0.61**	-0.16	-0.09	-0.22	-0.13	-0.13	0.17
**Iv-Vys_jl**	-0.36	-0.45	-0.32	-0.33	-0.07	-0.18	-0.13	-0.09	-0.19	0.51	0.01	0.08
**Iv-Vys_aug**	-0.38	-0.09	-0.23	0.11	0.14	-0.36	-0.09	-0.01	0.19	0.34	-0.14	-0.02
**Hum_may**	-0.09	-0.23	0.09	-0.16	**-0.59**	-0.02	-0.11	-0.16	0.24	0.19	-0.19	0.49
**Hum_jn**	**0.66**†	**0.57**	-0.27	-0.29	0.05	-0.42	-0.42	-0.39	-0.15	-0.43	-0.30	-0.03
**Hum_jl**	-0.29	-0.35	-0.44	-0.32	-0.05	-0.31	-0.20	-0.29	-0.07	0.29	-0.41	0.04
**Hum_aug**	-0.37	-0.34	-0.42	-0.48	-0.07	-0.51	-0.25	-0.28	-0.02	**0.62**	-0.16	0.38
**T_may**	0.07	0.26	-0.40	0.23	**0.59**	0.09	0.03	-0.07	-0.06	-0.07	-0.12	-0.52
**T_jn**	-0.03	0.12	-0.40	0.02	0.25	-0.10	-0.41	-0.27	0.03	-0.13	-0.29	-0.20
**T_jl**	0.42	0.27	0.21	0.09	-0.19	-0.11	-0.02	0.10	0.24	-0.02	0.17	0.34
**T_aug**	-0.03	0.10	0.40	0.54	-0.23	0.21	0.52	0.34	0.34	-0.19	-0.23	-0.04
**Prec_jn**	0.40	0.24	-0.05	-0.27	-0.08	**-0.60**	-0.18	-0.05	-0.14	-0.09	-0.08	0.14
**Prec_jl**	-0.47	-0.47	-0.16	-0.06	-0.10	0.08	-0.05	0.03	0.02	0.42	0.21	-0.01
**Prec_aug**	-0.27	0.09	0.02	0.38	0.20	-0.26	0.11	0.17	0.28	0.08	-0.16	-0.13
**Wind_my**	0.09	0.10	0.33	0.26	-0.05	-0.04	0.29	0.42	0.23	0.11	0.25	0.31
**Wind_jn**	0.08	0.20	0.16	0.16	0.10	-0.05	0.25	0.19	0.14	-0.03	0.37	-0.03
**Wind_jl**	-0.34	-0.26	0.07	0.09	0.20	0.50	0.29	0.02	-0.16	0.01	0.39	-0.53
**Wind_aug**	-0.04	-0.04	0.53	0.08	-0.24	0.34	0.25	0.15	-0.10	0.20	**0.58**	-0.09

* as in Table S1

** as in Table S1

† as in Table S1

Table S19. A Spearman rho correlation matrix for Scots pine #1 (continued 1).

Weather par	orgP	alcP	hclP	orgS	alcS	hclS	orgTi	alcTi	hclTi	orgMn	alcMn	hclMn
**Iv_jn_ind**	0.24	0.29	0.49	0.32	-0.04	0.13	-0.30	-0.21	-0.47	**0.59**	0.47	-0.07
**Iv_jl_ind**	0.12	**-0.59**	-0.05	0.29	-0.46	0.30	**-0.62**	**-0.81**	-0.10	-0.33	-0.41	-0.29
**Iv_au_ind**	0.14	-0.01	-0.13	-0.20	-0.05	-0.11	-0.02	-0.34	0.20	-0.34	-0.44	-0.05
**Iv-Vys_jn**	0.19	0.27	0.34	0.39	-0.12	-0.01	-0.48	-0.24	-0.43	**0.58**	0.48	-0.10
**Iv-Vys_jl**	0.27	**-0.57**	0.03	0.42	-0.46	0.40	-0.44	**-0.77**	-0.05	-0.25	-0.30	-0.28
**Iv-Vys_aug**	0.12	-0.12	0.11	-0.16	-0.03	0.11	-0.27	-0.47	0.23	-0.20	-0.34	-0.14
**Hum_may**	-0.08	0.08	0.41	-0.18	0.13	**0.58**	-0.09	0.23	-0.05	-0.07	-0.13	0.22
**Hum_jn**	0.09	0.24	0.34	0.10	0.12	-0.24	-0.24	0.03	-0.27	**0.73**	**0.70**	-0.16
**Hum_jl**	0.38	-0.35	0.09	0.29	-0.53	0.37	**-0.63**	-0.53	-0.08	-0.13	-0.25	-0.25
**Hum_aug**	0.42	-0.18	0.45	0.25	-0.35	0.50	-0.33	**-0.68**	0.29	-0.19	-0.37	-0.29
**T_may**	-0.05	-0.45	-0.51	0.07	0.02	-0.47	-0.23	-0.30	0.16	0.00	0.17	-0.24
**T_jn**	0.13	-0.02	0.08	**-0.57**	0.24	-0.25	0.03	-0.02	0.12	0.04	-0.10	-0.32
**T_jl**	-0.12	0.26	0.20	-0.06	0.34	0.30	-0.27	0.15	-0.23	0.42	0.27	-0.01
**T_aug**	-0.53	0.03	-0.31	-0.19	0.27	-0.09	-0.02	0.43	-0.18	-0.13	0.14	**0.64**
**Prec_jn**	0.19	0.26	0.29	0.31	-0.14	0.07	**-0.66**	-0.28	-0.48	0.53	0.35	-0.16
**Prec_jl**	0.02	**-0.65**	-0.09	0.25	-0.41	0.40	-0.32	**-0.70**	0.01	-0.38	-0.40	-0.18
**Prec_aug**	0.00	0.15	-0.03	-0.29	0.09	-0.10	-0.09	-0.12	0.18	-0.13	-0.23	0.05
**Wind_my**	-0.15	0.04	0.06	0.05	0.14	0.22	0.23	-0.14	-0.05	0.12	0.11	0.13
**Wind_jn**	-0.32	-0.09	-0.09	0.39	-0.18	-0.09	-0.10	-0.31	0.11	0.05	0.21	0.16
**Wind_jl**	-0.15	-0.31	**-0.57**	0.48	-0.50	-0.25	-0.04	-0.02	0.28	-0.51	-0.24	0.13
**Wind_aug**	-0.09	0.17	-0.11	**0.56**	0.00	0.20	0.20	0.33	0.15	-0.12	0.06	0.36

Table S19. A Spearman rho correlation matrix for Scots pine #1 (continued 2).

Weather par	orgFe	alcFe	hclFe	orgCu	alcCu	hclCu	orgZn	alcZn	hclZn
**Iv_jn_ind**	-0.12	0.01	0.12	-0.05	-0.01	0.20	**0.55**	0.33	**0.59**
**Iv_jl_ind**	-0.37	-0.21	-0.18	**0.61**	-0.37	0.54	0.51	-0.20	0.10
**Iv_au_ind**	-0.07	0.25	0.01	0.08	0.12	0.47	-0.33	-0.46	0.09
**Iv-Vys_jn**	-0.11	-0.08	0.15	-0.12	-0.19	0.16	0.54	0.30	0.44
**Iv-Vys_jl**	-0.50	-0.26	-0.31	**0.66**	-0.32	**0.56**	**0.60**	-0.18	0.13
**Iv-Vys_aug**	-0.09	0.10	-0.18	0.16	0.16	0.40	-0.20	-0.52	0.11
**Hum_may**	-0.27	-0.52	-0.23	0.13	0.47	-0.13	-0.20	0.11	0.07
**Hum_jn**	0.05	0.08	0.10	-0.30	-0.19	-0.12	0.35	0.11	0.29
**Hum_jl**	-0.53	-0.38	-0.27	0.37	-0.21	0.34	0.50	-0.30	0.05
**Hum_aug**	**-0.61**	-0.50	-0.50	**0.69**	-0.09	**0.58**	0.29	-0.28	0.34
**T_may**	0.07	0.29	-0.09	-0.11	-0.41	0.09	0.01	-0.49	-0.41
**T_jn**	-0.01	0.29	0.11	-0.02	0.02	0.15	-0.25	-0.53	0.07
**T_jl**	0.27	0.11	0.32	-0.42	0.42	-0.29	-0.08	0.15	-0.19
**T_aug**	0.13	-0.06	-0.04	-0.49	0.41	-0.29	**-0.62**	0.08	-0.29
**Prec_jn**	-0.02	-0.01	0.27	-0.19	-0.10	0.13	0.51	0.21	0.34
**Prec_jl**	-0.23	-0.07	-0.23	0.54	-0.21	0.38	0.41	-0.20	-0.04
**Prec_aug**	0.19	0.32	0.04	-0.25	0.32	0.14	-0.46	-0.42	0.00
**Wind_my**	0.12	0.31	0.18	-0.14	0.28	0.10	-0.09	0.09	0.00
**Wind_jn**	0.26	0.07	-0.11	-0.06	-0.22	-0.04	0.12	0.19	-0.01
**Wind_jl**	0.25	-0.19	-0.29	0.18	-0.51	-0.27	0.21	0.20	-0.37
**Wind_aug**	0.52	-0.08	-0.25	-0.09	0.27	-0.51	0.09	**0.59**	-0.23

Table S20. A Kendall tau correlation matrix for Scots pine #1.

Weather par	orgCa*	alcCa	hclCa	orgSr	alcSr	hclSr	orgK	alcK	hclK	orgAl	alcAl	hclAl
**Iv_jn_ind****	0.28	0.18	-0.03	-0.31	-0.21	-0.49	-0.18	-0.08	-0.13	-0.10	-0.10	0.21
**Iv_jl_ind**	-0.31	-0.36	-0.26	-0.18	-0.03	-0.10	-0.10	-0.05	-0.10	0.33	0.08	-0.03
**Iv_au_ind**	-0.36	-0.10	0.00	0.23	0.18	-0.15	0.15	0.21	0.05	0.18	-0.13	-0.08
**Iv-Vys_jn**	0.31	0.21	-0.05	-0.28	-0.08	**-0.46**	-0.10	-0.05	-0.15	-0.13	-0.13	0.13
**Iv-Vys_jl**	-0.31	-0.31	-0.26	-0.18	-0.08	-0.10	-0.10	-0.10	-0.15	0.38	0.03	0.08
**Iv-Vys_aug**	-0.31	-0.05	-0.10	0.08	0.13	-0.26	-0.05	0.00	0.10	0.23	-0.13	-0.03
**Hum_may**	-0.10	-0.21	0.00	-0.08	**-0.44**	0.00	-0.10	-0.10	0.15	0.13	-0.13	0.33
**Hum_jn**	**0.46**†	0.36	-0.21	-0.23	0.03	-0.31	-0.31	-0.26	-0.10	-0.33	-0.23	0.03
**Hum_jl**	-0.23	-0.18	-0.28	-0.21	-0.05	-0.23	-0.13	-0.23	-0.08	0.21	-0.31	0.10
**Hum_aug**	-0.25	-0.25	-0.27	**-0.43**	-0.04	-0.40	-0.22	-0.17	-0.09	**0.48**	-0.14	0.32
**T_may**	0.00	0.15	-0.26	0.13	**0.44**	0.05	0.05	0.00	-0.05	-0.03	-0.03	**-0.44**
**T_jn**	-0.05	0.10	-0.36	-0.03	0.23	-0.05	-0.31	-0.15	0.00	-0.08	-0.18	-0.13
**T_jl**	0.28	0.18	0.18	0.00	-0.15	-0.08	-0.03	0.08	0.18	0.00	0.15	0.26
**T_aug**	-0.10	0.05	0.31	0.38	-0.18	0.15	0.36	0.26	0.15	-0.08	-0.18	-0.03
**Prec_jn**	0.13	0.13	-0.03	-0.21	-0.10	**-0.44**	-0.08	-0.03	-0.13	-0.15	-0.05	0.10
**Prec_jl**	-0.31	-0.36	-0.15	-0.03	-0.08	0.05	-0.05	0.00	0.00	0.33	0.13	0.03
**Prec_aug**	-0.21	0.05	0.00	0.28	0.18	-0.15	0.10	0.15	0.21	0.08	-0.13	-0.13
**Wind_my**	0.03	0.08	0.26	0.26	-0.05	-0.03	0.21	0.31	0.16	0.05	0.21	0.23
**Wind_jn**	0.12	0.14	0.17	0.17	0.06	-0.04	0.19	0.09	0.17	-0.01	0.30	-0.01
**Wind_jl**	-0.30	-0.22	0.04	0.06	0.17	0.35	0.19	-0.01	-0.14	-0.01	0.22	-0.37
**Wind_aug**	0.01	0.01	**0.43**	0.04	-0.19	0.25	0.22	0.12	-0.04	0.12	0.37	-0.12

* as in Table S1

** as in Table S1

† as in Table S1

Table S20. A Kendall tau correlation matrix for Scots pine #1 (continued 1).

Weather par	orgP	alcP	hclP	orgS	alcS	hclS	orgTi	alcTi	hclTi	orgMn	alcMn	hclMn
**Iv_jn_ind**	0.15	0.21	0.33	0.23	-0.03	0.08	-0.21	-0.13	-0.36	**0.44**	0.38	-0.03
**Iv_jl_ind**	0.08	**-0.49**	0.00	0.21	-0.31	0.21	**-0.44**	**-0.62**	-0.08	-0.31	-0.31	-0.26
**Iv_au_ind**	0.08	0.08	-0.05	-0.05	-0.05	-0.05	-0.03	-0.26	0.13	-0.26	-0.31	0.00
**Iv-Vys_jn**	0.13	0.18	0.21	0.31	-0.05	0.00	-0.28	-0.10	-0.33	0.41	0.41	-0.05
**Iv-Vys_jl**	0.18	**-0.49**	0.00	0.36	-0.26	0.31	-0.33	**-0.56**	-0.08	-0.26	-0.21	-0.21
**Iv-Vys_aug**	0.08	-0.13	0.10	-0.10	0.00	0.10	-0.23	-0.36	0.13	-0.15	-0.21	-0.10
**Hum_may**	-0.08	0.03	0.31	-0.15	0.10	0.41	-0.03	0.15	-0.03	-0.10	-0.10	0.21
**Hum_jn**	0.03	0.18	0.21	0.05	0.10	-0.15	-0.13	0.05	-0.18	**0.56**	**0.51**	-0.10
**Hum_jl**	0.31	-0.26	0.08	0.18	-0.38	0.38	**-0.46**	-0.33	-0.05	-0.13	-0.13	-0.18
**Hum_aug**	0.32	-0.09	0.32	0.19	-0.25	0.40	-0.22	**-0.50**	0.19	-0.14	-0.25	-0.19
**T_may**	-0.03	-0.28	-0.31	0.05	0.00	-0.36	-0.23	-0.26	0.13	0.00	0.15	-0.10
**T_jn**	0.08	-0.03	0.05	-0.41	0.15	-0.15	0.03	0.00	0.08	0.05	-0.05	-0.26
**T_jl**	-0.10	0.21	0.18	-0.08	0.23	0.23	-0.21	0.13	-0.10	0.33	0.18	-0.03
**T_aug**	-0.38	0.08	-0.21	-0.05	0.21	-0.05	-0.03	0.26	-0.18	-0.10	0.05	**0.46**
**Prec_jn**	0.10	0.21	0.13	0.23	-0.13	0.08	**-0.46**	-0.18	-0.36	0.33	0.23	-0.08
**Prec_jl**	-0.03	**-0.54**	-0.05	0.15	-0.31	0.31	-0.18	**-0.46**	0.03	-0.31	-0.26	-0.15
**Prec_aug**	-0.03	0.08	0.00	-0.21	0.05	-0.05	-0.03	-0.05	0.08	-0.10	-0.15	0.05
**Wind_my**	-0.13	0.08	0.05	0.05	0.08	0.16	0.16	-0.10	-0.05	0.08	0.08	0.10
**Wind_jn**	-0.25	-0.09	-0.04	0.25	-0.14	-0.04	-0.09	-0.22	0.09	0.04	0.19	0.14
**Wind_jl**	-0.14	-0.25	**-0.45**	0.37	**-0.43**	-0.14	0.01	-0.01	0.22	-0.40	-0.19	0.04
**Wind_aug**	-0.04	0.14	-0.04	0.40	0.01	0.09	0.14	0.27	0.06	-0.04	0.12	0.17

Table S20. A Kendall tau correlation matrix for Scots pine #1 (continued 2).

Weather par	orgFe	alcFe	hclFe	orgCu	alcCu	hclCu	orgZn	alcZn	hclZn
**Iv_jn_ind**	-0.05	0.03	0.05	-0.05	-0.03	0.13	**0.49**	0.21	0.41
**Iv_jl_ind**	-0.28	-0.10	-0.13	**0.49**	-0.21	0.41	0.36	-0.13	0.13
**Iv_au_ind**	-0.08	0.21	-0.08	0.08	0.05	0.31	-0.26	-0.33	0.08
**Iv-Vys_jn**	-0.08	0.00	0.08	-0.08	-0.21	0.10	**0.46**	0.23	0.33
**Iv-Vys_jl**	-0.33	-0.21	-0.23	**0.49**	-0.21	**0.46**	**0.46**	-0.13	0.13
**Iv-Vys_aug**	-0.08	0.10	-0.13	0.08	0.10	0.31	-0.10	-0.38	0.08
**Hum_may**	-0.18	-0.36	-0.13	0.08	0.36	-0.05	-0.15	0.03	-0.03
**Hum_jn**	0.03	0.05	0.03	-0.18	-0.21	-0.05	0.26	0.08	0.18
**Hum_jl**	-0.36	-0.28	-0.15	0.26	-0.18	0.28	0.38	-0.21	0.10
**Hum_aug**	**-0.43**	-0.30	-0.40	**0.53**	-0.04	0.40	0.25	-0.19	0.27
**T_may**	0.08	0.21	-0.08	-0.08	-0.26	0.10	0.05	-0.38	-0.28
**T_jn**	0.03	0.26	0.08	-0.08	0.05	0.10	-0.15	-0.38	0.03
**T_jl**	0.15	0.08	0.21	-0.31	0.33	-0.18	-0.08	0.10	-0.10
**T_aug**	0.13	0.00	-0.03	-0.28	0.36	-0.21	**-0.46**	0.03	-0.18
**Prec_jn**	0.05	0.08	0.21	-0.15	-0.08	0.08	0.38	0.15	0.21
**Prec_jl**	-0.18	-0.05	-0.18	**0.49**	-0.15	0.31	0.31	-0.13	-0.03
**Prec_aug**	0.13	0.31	0.03	-0.13	0.21	0.10	-0.31	-0.28	-0.03
**Wind_my**	0.10	0.21	0.16	-0.13	0.26	0.05	-0.13	0.03	0.00
**Wind_jn**	0.22	-0.06	-0.04	-0.06	-0.14	-0.01	0.09	0.09	-0.04
**Wind_jl**	0.17	-0.12	-0.25	0.14	-0.37	-0.19	0.12	0.12	-0.30
**Wind_aug**	0.40	-0.04	-0.17	-0.04	0.17	-0.37	0.09	**0.45**	-0.12

Table S21. A Spearman rho correlation matrix for Scots pine #2.

Weather par	orgCa*	alcCa	hclCa	orgSr	alcSr	hclSr	orgK	alcK	hclK	orgAl	alcAl	hclAl
**Iv_jn_ind****	0.26	0.20	0.40	0.40	0.36	-0.03	0.05	-0.11	0.13	**-0.69**	-0.15	0.29
**Iv_jl_ind**	-0.41	-0.26	-0.24	**-0.57**	**-0.62**	-0.02	-0.14	-0.08	**0.59**	0.00	0.47	0.47
**Iv_au_ind**	-0.07	-0.20	-0.13	-0.32	-0.25	0.41	0.05	-0.15	0.30	0.15	-0.03	0.34
**Iv-Vys_jn**	0.25	0.18	0.32	0.43	0.30	-0.10	-0.07	-0.22	0.11	-0.54	-0.14	0.27
**Iv-Vys_jl**	-0.37	-0.27	-0.35	**-0.58**	**-0.63**	-0.10	0.01	0.03	0.52	-0.23	0.45	0.54
**Iv-Vys_aug**	-0.02	0.01	-0.26	-0.25	-0.21	0.42	0.01	-0.04	0.37	-0.05	0.24	0.46
**Hum_may**	-0.26	-0.05	-0.21	-0.03	0.01	-0.34	0.21	0.32	-0.37	-0.18	0.45	0.02
**Hum_jn**	0.46	0.37	0.19	**0.63**	0.42	-0.02	-0.17	-0.19	-0.07	-0.51	-0.31	0.07
**Hum_jl**	-0.42	-0.36	-0.54	-0.26	-0.40	-0.24	0.04	-0.05	0.25	-0.44	0.44	**0.66**
**Hum_aug**	-0.32	-0.28	-0.50	**-0.59**	**-0.56**	-0.11	0.02	-0.02	0.39	-0.38	0.51	**0.82**
**T_may**	0.23	0.19	-0.36	0.05	-0.24	0.33	-0.18	-0.07	0.19	0.21	-0.14	-0.04
**T_jn**	0.05	0.01	-0.16	-0.05	-0.09	0.18	-0.32	-0.27	0.04	0.00	-0.19	0.13
**T_jl**	0.30	**0.55**†	0.25	0.47	0.54	-0.08	-0.10	0.07	-0.15	-0.31	0.16	-0.16
**T_aug**	0.09	0.09	-0.05	0.40	0.21	0.14	0.44	0.39	-0.49	0.37	0.00	-0.53
**Prec_jn**	0.20	0.22	0.34	0.46	0.37	-0.07	-0.14	-0.27	0.17	**-0.55**	-0.03	0.31
**Prec_jl**	-0.35	-0.17	-0.25	**-0.59**	-0.54	0.05	0.00	0.10	**0.61**	0.01	0.52	0.34
**Prec_aug**	0.14	0.08	-0.05	0.07	0.13	0.52	0.09	-0.07	0.16	0.04	0.00	0.20
**Wind_my**	0.27	0.26	0.25	-0.01	0.16	0.12	0.14	0.10	0.22	-0.10	0.03	-0.07
**Wind_jn**	0.29	0.27	0.19	0.08	0.09	0.31	0.06	0.06	0.39	0.18	0.09	-0.05
**Wind_jl**	-0.27	-0.25	-0.10	-0.21	-0.22	0.13	0.09	0.12	0.14	**0.56**	0.12	-0.17
**Wind_aug**	0.10	0.31	0.33	0.22	0.45	0.28	0.43	0.53	-0.14	0.05	0.17	-0.34

* as in Table S1

** as in Table S1

† as in Table S1

Table S21. A Spearman rho correlation matrix for Scots pine #2 (continued 1).

Weather par	orgP	alcP	hclP	orgS	alcS	hclS	orgTi	alcTi	hclTi	orgMn	alcMn	hclMn
**Iv_jn_ind**	0.02	-0.31	0.15	-0.14	0.07	0.04	0.26	0.22	-0.18	-0.15	-0.13	0.25
**Iv_jl_ind**	0.24	0.48	0.39	-0.24	**0.57**	0.21	0.46	0.01	-0.17	-0.23	0.05	0.28
**Iv_au_ind**	0.18	-0.07	0.03	0.26	**0.62**	-0.05	0.44	-0.37	-0.24	0.44	**0.65**	-0.19
**Iv-Vys_jn**	0.01	-0.38	0.17	-0.31	0.01	-0.19	0.27	0.10	-0.07	-0.13	-0.18	0.21
**Iv-Vys_jl**	0.26	0.55	0.44	-0.19	**0.60**	0.16	0.34	0.07	0.02	-0.28	0.04	0.24
**Iv-Vys_aug**	0.18	-0.12	0.05	0.13	**0.58**	-0.07	0.30	-0.28	-0.10	0.29	0.54	-0.12
**Hum_may**	0.37	0.14	-0.22	0.22	-0.13	0.17	-0.22	0.41	0.14	-0.03	-0.09	0.13
**Hum_jn**	-0.34	-0.43	-0.01	-0.43	-0.32	-0.30	-0.01	0.03	0.03	-0.31	-0.34	-0.03
**Hum_jl**	0.24	0.39	0.41	-0.34	0.45	-0.01	0.32	-0.04	0.18	-0.44	-0.17	-0.03
**Hum_aug**	0.44	0.26	0.13	-0.04	**0.64**	0.01	0.21	-0.13	0.19	0.01	0.36	0.23
**T_may**	-0.35	-0.04	0.17	-0.35	0.14	-0.51	0.07	-0.30	0.17	-0.10	0.03	-0.45
**T_jn**	-0.30	0.00	-0.07	-0.07	0.04	-0.10	0.09	-0.23	-0.14	-0.15	0.10	-0.27
**T_jl**	-0.01	-0.51	0.27	0.09	-0.32	-0.29	-0.37	0.28	0.38	0.08	-0.29	0.11
**T_aug**	0.19	-0.21	-0.39	0.23	-0.20	-0.16	0.03	0.25	-0.12	0.38	0.11	-0.32
**Prec_jn**	0.02	-0.43	0.32	-0.29	0.04	-0.17	0.30	0.07	-0.05	-0.15	-0.24	0.19
**Prec_jl**	0.20	0.53	0.44	-0.13	0.46	0.30	0.24	0.05	-0.06	-0.18	0.03	0.30
**Prec_aug**	0.08	-0.39	-0.04	0.24	0.36	-0.13	0.25	-0.35	-0.14	0.45	0.52	-0.27
**Wind_my**	0.12	-0.20	0.29	0.19	-0.01	-0.13	-0.09	0.13	0.14	0.41	0.15	0.30
**Wind_jn**	0.06	-0.32	0.01	-0.17	-0.01	-0.11	0.04	-0.15	-0.02	0.37	0.18	0.29
**Wind_jl**	-0.02	0.26	-0.13	-0.13	0.09	0.30	0.01	-0.28	-0.09	-0.08	-0.03	-0.08
**Wind_aug**	0.13	-0.25	-0.17	0.50	0.04	0.42	-0.43	0.16	0.12	0.21	0.07	-0.02

Table S21. A Spearman rho correlation matrix for Scots pine #2 (continued 2).

Weather par	orgFe	alcFe	hclFe	orgCu	alcCu	hclCu	orgZn	alcZn	hclZn
**Iv_jn_ind**	-0.31	0.46	0.21	**-0.55**	0.30	0.41	0.52	-0.18	-0.02
**Iv_jl_ind**	-0.47	-0.30	-0.21	-0.18	0.02	-0.07	0.14	-0.29	0.12
**Iv_au_ind**	-0.08	-0.34	-0.28	-0.06	-0.13	0.20	-0.32	-0.22	-0.08
**Iv-Vys_jn**	-0.32	0.37	0.20	**-0.57**	0.10	0.25	0.49	-0.38	-0.05
**Iv-Vys_jl**	**-0.61**	-0.24	-0.18	-0.26	0.12	-0.08	0.08	-0.26	0.08
**Iv-Vys_aug**	-0.20	-0.19	-0.24	-0.35	-0.07	0.03	-0.21	-0.11	-0.11
**Hum_may**	-0.15	-0.10	-0.41	-0.07	0.33	0.06	-0.18	**0.69**	0.18
**Hum_jn**	-0.07	0.52	0.37	-0.54	0.07	0.15	0.30	-0.12	-0.18
**Hum_jl**	**-0.76**	-0.14	-0.36	-0.45	0.05	0.07	-0.18	-0.05	0.00
**Hum_aug**	**-0.79**	-0.32	-0.46	**-0.57**	0.40	-0.04	-0.12	0.01	0.19
**T_may**	0.13	-0.04	0.31	-0.11	**-0.63**	-0.40	-0.20	**-0.59**	-0.38
**T_jn**	0.15	-0.04	-0.13	-0.12	0.09	0.22	-0.32	0.41	-0.08
**T_jl**	0.22	0.45	0.20	-0.24	-0.18	-0.25	0.42	0.07	-0.27
**T_aug**	0.38	-0.18	-0.04	0.27	-0.46	0.01	-0.37	-0.08	-0.20
**Prec_jn**	-0.29	0.42	0.15	**-0.58**	-0.01	0.23	0.54	-0.35	-0.10
**Prec_jl**	-0.37	-0.23	-0.08	-0.03	-0.01	-0.22	0.14	-0.26	-0.01
**Prec_aug**	0.06	-0.07	-0.13	-0.17	-0.25	0.12	-0.24	-0.15	-0.22
**Wind_my**	0.08	0.10	0.23	-0.01	-0.06	-0.17	0.32	-0.37	-0.33
**Wind_jn**	-0.02	0.06	0.38	-0.10	-0.26	-0.39	0.41	**-0.82**	-0.16
**Wind_jl**	-0.01	-0.17	0.12	0.45	-0.35	-0.38	0.00	-0.44	0.28
**Wind_aug**	0.23	0.40	0.37	0.28	-0.19	-0.38	0.40	-0.14	0.23

Table S22. A Kendall tau correlation matrix for Scots pine #2.

Weather par	orgCa*	alcCa	hclCa	orgSr	alcSr	hclSr	orgK	alcK	hclK	orgAl	alcAl	hclAl
**Iv_jn_ind****	0.21	0.15	0.28	0.26	0.26	-0.03	0.08	-0.10	0.05	**-0.56**	-0.10	0.26
**Iv_jl_ind**	-0.28	-0.13	-0.15	**-0.44**[†]	**-0.49**	-0.05	-0.10	-0.08	0.28	0.03	0.33	0.33
**Iv_au_ind**	-0.03	-0.18	-0.15	-0.23	-0.18	0.31	0.05	-0.13	0.23	0.18	-0.03	0.23
**Iv-Vys_jn**	0.23	0.18	0.26	0.28	0.18	-0.05	0.00	-0.18	0.03	**-0.44**	-0.08	0.18
**Iv-Vys_jl**	-0.23	-0.18	-0.26	**-0.49**	**-0.49**	-0.10	0.00	0.03	0.28	-0.18	0.33	0.38
**Iv-Vys_aug**	0.03	-0.03	-0.21	-0.13	-0.13	0.26	0.00	-0.03	0.28	-0.08	0.18	0.33
**Hum_may**	-0.18	-0.03	-0.10	-0.03	-0.03	-0.21	0.10	0.23	-0.28	-0.13	0.33	-0.03
**Hum_jn**	0.28	0.18	0.10	**0.44**	0.28	-0.05	-0.10	-0.13	-0.08	**-0.44**	-0.23	0.03
**Hum_jl**	-0.21	-0.21	-0.38	-0.15	-0.31	-0.18	0.03	-0.05	0.10	-0.31	0.41	**0.46**
**Hum_aug**	-0.22	-0.17	-0.37	**-0.48**	**-0.43**	-0.04	0.06	-0.01	0.27	-0.25	**0.43**	**0.63**
**T_may**	0.18	0.13	-0.26	0.03	-0.18	0.31	-0.15	-0.08	0.13	0.18	-0.08	-0.08
**T_jn**	0.03	0.03	-0.10	-0.03	-0.08	0.10	-0.26	-0.18	-0.03	-0.03	-0.18	0.08
**T_jl**	0.21	0.41	0.18	0.36	0.41	-0.13	-0.08	0.05	-0.10	-0.21	0.10	-0.10
**T_aug**	0.08	0.08	-0.05	0.23	0.13	0.10	0.31	0.28	-0.28	0.28	0.03	-0.33
**Prec_jn**	0.15	0.21	0.23	0.31	0.26	-0.08	-0.13	-0.26	0.10	**-0.46**	0.00	0.21
**Prec_jl**	-0.23	-0.13	-0.15	**-0.49**	**-0.44**	0.00	0.00	0.03	0.33	0.03	0.38	0.23
**Prec_aug**	0.13	0.03	0.00	-0.03	0.03	0.41	0.10	-0.03	0.08	0.03	-0.03	0.13
**Wind_my**	0.21	0.21	0.18	-0.03	0.03	0.18	0.10	0.08	0.18	-0.10	0.03	-0.08
**Wind_jn**	0.17	0.09	0.09	0.06	0.06	0.19	0.04	0.04	0.32	0.09	0.04	-0.04
**Wind_jl**	-0.19	-0.14	-0.12	-0.14	-0.19	0.09	0.04	0.06	0.09	**0.43**	0.09	-0.06
**Wind_aug**	0.09	0.22	0.27	0.19	0.35	0.22	0.27	0.37	-0.09	0.04	0.12	-0.30

* as in Table S1

** as in Table S1

[†] as in Table S1

Table S22. A Kendall tau correlation matrix for Scots pine #2 (continued 1).

Weather par	orgP	alcP	hclP	orgS	alcS	hclS	orgTi	alcTi	hclTi	orgMn	alcMn	hclMn
**Iv_jn_ind**	0.00	-0.21	0.07	-0.10	0.00	0.04	0.23	0.18	-0.08	-0.08	-0.13	0.18
**Iv_jl_ind**	0.18	0.38	0.30	-0.13	**0.44**	0.16	0.36	0.00	-0.15	-0.15	0.05	0.26
**Iv_au_ind**	0.13	-0.03	0.04	0.13	**0.44**	-0.04	0.31	-0.26	-0.10	0.36	**0.51**	-0.10
**Iv-Vys_jn**	0.03	-0.23	0.09	-0.28	-0.03	-0.10	0.21	0.05	0.05	-0.05	-0.15	0.15
**Iv-Vys_jl**	0.18	0.38	0.35	-0.13	**0.44**	0.10	0.26	0.05	0.00	-0.21	0.00	0.21
**Iv-Vys_aug**	0.13	-0.13	0.01	0.03	**0.44**	-0.04	0.21	-0.21	-0.05	0.26	0.36	-0.05
**Hum_may**	0.28	0.13	-0.14	0.13	-0.08	0.10	-0.15	0.36	0.10	0.00	-0.05	0.05
**Hum_jn**	-0.23	-0.28	-0.04	**-0.44**	-0.28	-0.19	0.00	0.00	0.10	-0.26	-0.21	-0.10
**Hum_jl**	0.15	0.26	0.27	-0.31	0.31	-0.01	0.18	-0.08	0.13	-0.33	-0.13	-0.03
**Hum_aug**	0.32	0.17	0.05	-0.06	**0.43**	0.06	0.19	-0.04	0.12	0.01	0.27	0.14
**T_may**	-0.28	-0.03	0.12	-0.28	0.08	-0.40	0.10	-0.15	0.05	-0.05	0.00	-0.36
**T_jn**	-0.28	0.03	-0.07	-0.18	0.03	-0.07	0.05	-0.15	-0.10	-0.05	0.05	-0.15
**T_jl**	-0.05	-0.36	0.20	0.05	-0.26	-0.22	-0.23	0.23	0.28	0.08	-0.18	0.03
**T_aug**	0.18	-0.18	-0.25	0.18	-0.08	-0.13	0.05	0.21	-0.10	0.31	0.05	-0.21
**Prec_jn**	0.00	-0.31	0.22	-0.31	0.00	-0.04	0.23	0.08	0.08	-0.03	-0.18	0.08
**Prec_jl**	0.13	**0.44**	0.33	-0.13	0.33	0.22	0.15	0.05	0.00	-0.10	0.05	0.26
**Prec_aug**	0.08	-0.18	-0.07	0.13	0.23	-0.10	0.15	-0.21	-0.05	0.36	0.36	-0.10
**Wind_my**	0.05	-0.16	0.20	0.13	-0.08	-0.08	-0.08	0.08	0.13	0.26	0.08	0.16
**Wind_jn**	-0.01	-0.22	0.03	-0.06	-0.04	-0.14	-0.01	-0.14	-0.04	0.25	0.19	0.22
**Wind_jl**	0.04	0.14	-0.08	-0.14	0.06	0.24	0.04	-0.25	-0.04	-0.06	-0.01	-0.09
**Wind_aug**	0.09	-0.19	-0.11	0.40	0.01	0.32	-0.30	0.09	0.09	0.12	0.06	0.01

Table S22. A Kendall tau correlation matrix for Scots pine #2 (continued 2).

Weather par	orgFe	alcFe	hclFe	orgCu	alcCu	hclCu	orgZn	alcZn	hclZn
**Iv_jn_ind**	-0.21	0.33	0.13	**-0.44**	0.18	0.28	0.31	-0.10	0.00
**Iv_jl_ind**	-0.28	-0.26	-0.10	-0.21	0.05	-0.05	0.23	-0.18	0.08
**Iv_au_ind**	-0.08	-0.21	-0.21	0.00	-0.05	0.15	-0.23	-0.13	-0.08
**Iv-Vys_jn**	-0.18	0.26	0.21	-0.41	0.05	0.15	0.33	-0.18	-0.03
**Iv-Vys_jl**	-0.38	-0.21	-0.10	-0.21	0.05	-0.10	0.13	-0.13	0.03
**Iv-Vys_aug**	-0.18	-0.10	-0.10	-0.26	-0.10	0.00	-0.18	-0.03	-0.08
**Hum_may**	-0.08	-0.10	-0.26	-0.10	0.21	0.05	-0.08	**0.49**	0.08
**Hum_jn**	-0.03	0.41	0.21	-0.41	0.00	0.15	0.08	-0.08	-0.13
**Hum_jl**	**-0.56**	-0.08	-0.18	-0.33	0.03	0.08	-0.10	-0.05	0.00
**Hum_aug**	**-0.58**	-0.25	-0.35	-0.37	0.30	-0.01	-0.09	0.09	0.17
**T_may**	0.13	-0.05	0.21	-0.15	**-0.46**	-0.31	-0.13	**-0.44**	-0.28
**T_jn**	0.13	0.00	-0.05	-0.15	0.00	0.15	-0.23	0.38	-0.03
**T_jl**	0.10	0.33	0.13	-0.23	-0.18	-0.18	0.26	0.00	-0.15
**T_aug**	0.23	-0.10	-0.05	0.21	-0.31	-0.05	-0.23	-0.03	-0.18
**Prec_jn**	-0.21	0.33	0.13	**-0.44**	-0.08	0.18	0.36	-0.15	-0.05
**Prec_jl**	-0.18	-0.15	0.00	-0.05	0.00	-0.15	0.13	-0.18	0.03
**Prec_aug**	0.08	0.00	0.00	-0.15	-0.15	0.05	-0.13	-0.08	-0.13
**Wind_my**	0.05	0.03	0.13	0.03	-0.08	-0.08	0.21	-0.26	-0.23
**Wind_jn**	-0.01	0.04	0.25	-0.09	-0.19	-0.27	0.25	**-0.68**	-0.17
**Wind_jl**	-0.04	-0.12	0.14	0.35	-0.25	-0.22	-0.01	-0.27	0.17
**Wind_aug**	0.25	0.30	0.30	0.22	-0.09	-0.30	0.27	-0.09	0.17

Table S23. A Spearman rho correlation matrix for Scots pine #3.

Weather par	orgCa*	alcCa	hclCa	orgSr	alcSr	hclSr	orgK	alcK	hclK	orgAl	alcAl	hclAl
**Iv_jn_ind****	0.32	0.25	**0.64**†	0.25	0.27	0.27	-0.09	-0.38	0.41	0.09	-0.37	0.19
**Iv_jl_ind**	0.13	0.15	0.48	-0.29	0.17	-0.25	-0.23	-0.29	0.25	0.01	0.17	0.01
**Iv_au_ind**	-0.40	-0.13	-0.26	-0.18	-0.25	-0.02	-0.19	0.05	-0.19	-0.23	-0.12	-0.40
**Iv-Vys_jn**	0.21	0.20	0.42	0.16	0.25	0.21	-0.06	-0.33	0.19	0.05	-0.31	0.13
**Iv-Vys_jl**	0.28	0.20	**0.61**	-0.16	0.31	-0.18	-0.09	-0.22	0.29	-0.02	0.23	0.09
**Iv-Vys_aug**	-0.19	0.01	-0.25	-0.40	-0.15	0.01	-0.25	-0.12	-0.20	-0.41	0.08	-0.36
**Hum_may**	0.43	0.20	0.01	**-0.57**	-0.34	-0.35	0.30	0.14	-0.03	-0.05	0.18	0.40
**Hum_jn**	0.11	0.02	0.26	0.31	0.34	0.38	-0.19	-0.39	0.27	-0.08	-0.24	-0.01
**Hum_jl**	0.12	-0.10	0.40	-0.34	0.14	-0.20	0.02	-0.11	0.24	-0.14	0.15	-0.02
**Hum_aug**	0.46	0.40	0.25	**-0.61**	-0.03	-0.26	-0.16	-0.24	-0.08	-0.21	0.38	0.12
**T_may**	-0.49	-0.31	-0.32	0.25	0.45	0.25	-0.15	-0.02	-0.20	-0.38	0.10	-0.49
**T_jn**	-0.28	-0.29	-0.02	-0.09	-0.12	0.03	-0.47	-0.34	0.32	-0.31	-0.16	-0.22
**T_jl**	0.07	0.05	-0.27	-0.12	-0.05	0.13	0.23	-0.04	-0.19	-0.44	-0.08	0.22
**T_aug**	-0.32	-0.18	**-0.57**	-0.03	-0.24	-0.03	0.54	**0.63**	-0.45	0.01	-0.09	-0.18
**Prec_jn**	0.08	0.08	0.32	0.03	0.16	0.18	-0.08	-0.35	0.18	-0.08	-0.35	0.08
**Prec_jl**	0.16	0.14	0.43	-0.12	0.21	-0.12	-0.08	-0.13	0.25	0.00	0.30	-0.03
**Prec_aug**	-0.45	-0.20	-0.53	-0.21	-0.28	0.15	-0.12	0.09	-0.33	-0.36	-0.12	-0.49
**Wind_my**	0.11	0.22	0.02	0.33	0.04	0.28	0.26	0.13	-0.08	-0.15	0.01	0.06
**Wind_jn**	0.05	0.32	-0.23	0.18	0.19	0.29	0.05	0.07	-0.44	0.10	0.25	-0.24
**Wind_jl**	-0.19	-0.07	-0.31	-0.02	0.16	-0.13	0.01	0.28	-0.42	0.47	0.35	-0.28
**Wind_aug**	0.13	0.24	-0.42	-0.03	0.16	0.10	0.28	0.34	**-0.61**	0.23	0.20	0.05

* as in Table S1

** as in Table S1

† as in Table S1

Table S23. A Spearman rho correlation matrix for Scots pine #3 (continued 1).

Weather par	orgP	alcP	hclP	orgS	alcS	hclS	orgTi	alcTi	hclTi	orgMn	alcMn	hclMn
**Iv_jn_ind**	0.09	0.07	-0.08	0.23	-0.44	-0.09	0.35	-0.18	0.50	0.30	0.30	-0.12
**Iv_jl_ind**	-0.48	0.11	-0.11	**0.65**	0.21	0.16	0.38	0.03	0.36	0.39	0.09	0.52
**Iv_au_ind**	0.29	0.15	0.08	-0.04	0.29	0.25	**0.60**	0.16	-0.23	-0.10	-0.32	0.30
**Iv-Vys_jn**	0.09	0.06	-0.15	0.21	-0.36	-0.30	0.18	-0.16	0.34	0.15	0.20	-0.06
**Iv-Vys_jl**	-0.42	0.05	-0.15	**0.72**	0.11	0.30	0.34	-0.07	0.25	0.48	0.20	0.45
**Iv-Vys_aug**	0.24	0.20	-0.23	0.04	0.36	0.29	**0.77**	0.15	-0.16	0.09	-0.14	0.41
**Hum_may**	0.35	0.29	-0.04	0.18	-0.09	0.32	0.08	-0.47	0.14	0.31	0.40	-0.19
**Hum_jn**	0.00	-0.16	-0.26	-0.17	-0.42	-0.34	0.04	0.10	0.18	-0.03	0.10	-0.10
**Hum_jl**	-0.05	-0.12	-0.36	**0.62**	-0.13	0.34	0.42	-0.13	0.13	0.25	0.07	0.29
**Hum_aug**	0.18	0.34	-0.15	0.54	0.04	**0.63**	**0.62**	-0.22	-0.14	**0.65**	0.45	0.49
**T_may**	-0.39	-0.33	-0.40	-0.16	0.42	-0.43	-0.08	0.51	-0.35	-0.43	-0.53	0.29
**T_jn**	-0.02	-0.10	-0.03	-0.41	-0.12	0.33	0.43	0.42	0.04	-0.11	-0.21	0.31
**T_jl**	0.21	0.18	-0.47	-0.16	0.07	-0.13	0.02	-0.18	0.16	-0.10	0.06	-0.05
**T_aug**	0.35	0.06	0.01	-0.13	0.34	**-0.62**	-0.32	-0.23	-0.17	-0.49	-0.35	-0.53
**Prec_jn**	0.11	0.05	-0.30	0.20	-0.29	-0.24	0.34	-0.11	0.43	0.07	0.08	0.03
**Prec_jl**	-0.53	0.06	-0.15	**0.62**	0.29	0.23	0.25	-0.01	0.26	0.37	0.10	0.46
**Prec_aug**	0.46	0.10	-0.16	-0.24	0.32	0.08	**0.62**	0.17	-0.27	-0.25	-0.37	0.15
**Wind_my**	0.07	0.28	-0.05	0.09	0.13	-0.07	-0.10	-0.26	-0.02	0.04	0.09	0.06
**Wind_jn**	-0.10	0.16	-0.10	0.17	0.37	**-0.57**	-0.21	-0.06	-0.24	0.01	0.04	0.06
**Wind_jl**	-0.39	-0.33	0.11	0.19	0.41	-0.36	-0.40	0.23	-0.31	-0.14	-0.21	-0.11
**Wind_aug**	0.06	-0.09	-0.10	0.01	0.49	-0.17	-0.12	-0.02	-0.18	0.04	0.08	-0.49

Table S23. A Spearman rho correlation matrix for Scots pine #3 (continued 2).

Weather par	orgFe	alcFe	hclFe	orgCu	alcCu	hclCu	orgZn	alcZn	hclZn
**Iv_jn_ind**	0.20	**0.59**	**0.58**	0.02	0.05	-0.46	0.27	0.04	0.01
**Iv_jl_ind**	**0.74**	**0.75**	**0.59**	-0.21	-0.07	0.07	0.27	-0.23	0.50
**Iv_au_ind**	0.28	0.12	-0.08	-0.21	-0.04	0.33	-0.34	-0.40	-0.17
**Iv-Vys_jn**	0.12	0.42	0.38	0.03	0.14	-0.38	0.21	0.03	0.00
**Iv-Vys_jl**	**0.76**	**0.84**	**0.74**	-0.14	-0.15	-0.02	0.30	-0.10	0.49
**Iv-Vys_aug**	0.24	0.21	0.07	-0.21	-0.10	0.21	-0.13	-0.13	-0.01
**Hum_may**	-0.26	-0.18	0.19	0.45	-0.02	0.25	0.34	**0.70**	-0.27
**Hum_jn**	-0.09	0.10	0.09	-0.16	-0.14	**-0.57**	0.05	0.17	0.04
**Hum_jl**	0.49	0.52	**0.59**	0.05	-0.20	0.04	0.21	0.18	0.14
**Hum_aug**	0.53	**0.61**	0.53	-0.07	-0.28	0.22	0.29	0.26	0.16
**T_may**	0.11	-0.07	-0.24	-0.43	-0.12	-0.18	-0.43	-0.35	0.35
**T_jn**	0.23	-0.08	-0.19	-0.39	-0.42	-0.07	-0.30	-0.07	-0.11
**T_jl**	-0.41	-0.27	-0.04	0.47	**0.57**	-0.20	0.21	0.34	-0.25
**T_aug**	**-0.68**	**-0.65**	-0.34	0.32	0.26	0.30	-0.20	0.17	-0.34
**Prec_jn**	0.11	0.39	0.37	0.10	0.27	-0.34	0.21	-0.01	-0.06
**Prec_jl**	**0.63**	**0.67**	**0.57**	-0.11	0.00	0.01	0.36	-0.17	**0.56**
**Prec_aug**	-0.11	-0.15	-0.25	-0.08	0.09	0.23	-0.31	-0.21	-0.27
**Wind_my**	0.01	0.09	0.14	0.26	0.45	-0.15	0.26	-0.01	-0.01
**Wind_jn**	-0.16	0.11	-0.08	-0.07	0.33	-0.09	0.21	-0.18	0.39
**Wind_jl**	-0.15	-0.03	-0.26	-0.19	0.02	0.26	-0.14	-0.43	0.47
**Wind_aug**	**-0.62**	-0.07	-0.07	0.26	0.43	0.09	-0.02	-0.17	0.17

Table S24. A Kendall tau correlation matrix for Scots pine #3.

Weather par	orgCa*	alcCa	hclCa	orgSr	alcSr	hclSr	orgK	alcK	hclK	orgAl	alcAl	hclAl
**Iv_jn_ind****	0.31	0.21	0.46	0.21	0.15	0.26	-0.03	-0.26	0.31	0.08	-0.23	0.18
**Iv_jl_ind**	0.08	0.13	0.33	-0.13	0.08	-0.18	-0.21	-0.23	0.13	0.00	0.10	-0.05
**Iv_au_ind**	-0.28	-0.13	-0.23	-0.08	-0.18	-0.03	-0.10	0.03	-0.13	-0.21	-0.10	-0.31
**Iv-Vys_jn**	0.18	0.13	0.23	0.18	0.23	0.23	-0.05	-0.28	0.08	0.05	-0.26	0.05
**Iv-Vys_jl**	0.18	0.13	**0.49†**	-0.08	0.23	-0.13	-0.10	-0.18	0.23	-0.05	0.21	0.00
**Iv-Vys_aug**	-0.13	-0.03	-0.18	-0.28	-0.03	0.03	-0.21	-0.13	-0.13	-0.21	0.10	-0.26
**Hum_may**	0.38	0.18	0.03	-0.38	-0.23	-0.23	0.21	0.08	0.03	-0.05	0.15	0.26
**Hum_jn**	0.13	-0.03	0.13	0.23	0.23	0.28	-0.10	-0.33	0.18	-0.05	-0.15	0.00
**Hum_jl**	0.15	-0.05	0.26	-0.26	0.10	-0.15	-0.03	-0.05	0.15	-0.08	0.13	0.03
**Hum_aug**	0.35	0.25	0.22	**-0.43**	-0.06	-0.17	-0.09	-0.17	-0.09	-0.06	0.27	0.09
**T_may**	-0.33	-0.13	-0.23	0.18	0.33	0.18	-0.10	0.03	-0.13	-0.26	0.05	-0.41
**T_jn**	-0.18	-0.18	-0.03	-0.03	-0.08	0.08	-0.36	-0.28	0.23	-0.15	-0.10	-0.15
**T_jl**	0.05	0.05	-0.21	-0.10	-0.05	0.00	0.13	0.00	-0.15	-0.33	-0.08	0.23
**T_aug**	-0.33	-0.13	**-0.44**	-0.03	-0.18	-0.03	0.31	**0.44**	-0.28	0.05	-0.10	-0.21
**Prec_jn**	0.10	0.10	0.21	0.00	0.10	0.15	-0.08	-0.26	0.10	-0.03	-0.28	0.03
**Prec_jl**	0.13	0.13	0.33	-0.03	0.13	-0.13	-0.05	-0.08	0.18	-0.05	0.21	0.00
**Prec_aug**	-0.33	-0.13	**-0.44**	-0.03	-0.18	0.18	-0.10	0.03	-0.23	-0.26	-0.10	-0.41
**Wind_my**	0.08	0.16	-0.03	0.23	0.00	0.21	0.21	0.08	-0.08	-0.13	-0.05	-0.03
**Wind_jn**	0.09	0.25	-0.12	0.12	0.17	0.17	0.12	0.12	-0.30	0.04	0.22	-0.14
**Wind_jl**	-0.12	-0.06	-0.27	-0.04	0.09	-0.09	-0.04	0.12	-0.32	0.35	0.27	-0.19
**Wind_aug**	0.12	0.17	-0.30	-0.01	0.17	0.09	0.27	0.25	**-0.50**	0.22	0.14	0.04

* as in Table S1

** as in Table S1

† as in Table S1

Table S24. A Kendall tau correlation matrix for Scots pine #3 (continued 1).

Weather par	orgP	alcP	hclP	orgS	alcS	hclS	orgTi	alcTi	hclTi	orgMn	alcMn	hclMn
**Iv_jn_ind**	0.05	0.08	-0.05	0.13	-0.33	-0.08	0.26	-0.18	0.33	0.18	0.26	-0.05
**Iv_jl_ind**	-0.33	0.15	-0.08	**0.46**	0.15	0.11	0.28	0.00	0.26	0.26	0.08	**0.44**
**Iv_au_ind**	0.23	0.10	0.03	-0.05	0.21	0.24	0.38	0.10	-0.21	-0.05	-0.23	0.28
**Iv-Vys_jn**	0.08	0.05	-0.13	0.15	-0.26	-0.19	0.13	-0.15	0.21	0.10	0.13	-0.03
**Iv-Vys_jl**	-0.28	0.10	-0.08	**0.51**	0.05	0.21	0.23	-0.05	0.21	0.31	0.13	0.33
**Iv-Vys_aug**	0.23	0.10	-0.13	0.05	0.26	0.21	**0.59**	0.10	-0.05	0.05	-0.13	0.38
**Hum_may**	0.28	0.21	-0.03	0.10	-0.05	0.19	0.03	-0.36	0.10	0.21	0.33	-0.13
**Hum_jn**	-0.03	-0.10	-0.18	-0.10	-0.31	-0.21	-0.03	0.05	0.10	0.00	0.08	-0.08
**Hum_jl**	0.00	-0.08	-0.26	**0.44**	-0.08	0.24	0.36	-0.13	0.08	0.18	0.10	0.15
**Hum_aug**	0.19	0.30	-0.09	0.40	0.06	**0.50**	**0.45**	-0.19	-0.12	**0.45**	0.32	0.37
**T_may**	-0.23	-0.21	-0.28	-0.10	0.26	-0.32	-0.08	0.36	-0.26	-0.36	-0.38	0.18
**T_jn**	0.08	-0.10	-0.03	-0.31	-0.10	0.21	0.28	0.31	0.05	-0.15	-0.18	0.23
**T_jl**	0.21	0.13	-0.36	-0.13	0.03	-0.11	0.00	-0.13	0.08	-0.13	0.05	-0.05
**T_aug**	0.28	0.05	0.03	-0.05	0.21	**-0.43**	-0.23	-0.21	-0.15	-0.36	-0.28	-0.33
**Prec_jn**	0.10	0.03	-0.21	0.13	-0.28	-0.16	0.26	-0.08	0.28	0.03	0.05	0.05
**Prec_jl**	-0.38	0.10	-0.13	0.41	0.21	0.16	0.18	0.00	0.15	0.26	0.08	0.33
**Prec_aug**	0.38	0.05	-0.13	-0.15	0.26	0.05	0.38	0.10	-0.26	-0.15	-0.28	0.18
**Wind_my**	0.03	0.21	0.00	0.08	0.08	-0.03	-0.08	-0.18	0.00	0.05	0.08	0.03
**Wind_jn**	-0.09	0.12	-0.09	0.12	0.27	**-0.47**	-0.14	-0.01	-0.14	0.01	0.04	-0.01
**Wind_jl**	-0.30	-0.25	0.12	0.17	0.30	-0.26	-0.25	0.14	-0.19	-0.09	-0.12	-0.06
**Wind_aug**	-0.01	-0.09	-0.12	0.04	0.35	-0.12	-0.04	-0.01	-0.06	0.09	0.06	-0.30

Table S24. A Kendall tau correlation matrix for Scots pine #3 (continued 2).

Weather par	orgFe	alcFe	hclFe	orgCu	alcCu	hclCu	orgZn	alcZn	hclZn
**Iv_jn_ind**	0.10	**0.46**	0.41	0.03	0.03	-0.36	0.23	0.00	0.03
**Iv_jl_ind**	**0.59**	**0.54**	**0.44**	-0.15	0.00	0.08	0.21	-0.18	0.36
**Iv_au_ind**	0.18	0.08	-0.03	-0.15	-0.05	0.23	-0.26	-0.28	-0.21
**Iv-Vys_jn**	0.08	0.33	0.33	0.00	0.10	-0.33	0.15	0.03	0.00
**Iv-Vys_jl**	**0.59**	**0.64**	**0.59**	-0.10	-0.05	0.03	0.26	-0.08	0.41
**Iv-Vys_aug**	0.18	0.13	0.08	-0.21	-0.10	0.08	-0.10	-0.08	0.05
**Hum_may**	-0.18	-0.13	0.13	0.36	0.00	0.18	0.26	**0.54**	-0.21
**Hum_jn**	-0.08	0.08	0.08	-0.10	-0.05	-0.38	0.00	0.13	0.00
**Hum_jl**	0.36	0.41	**0.46**	-0.03	-0.13	0.10	0.18	0.15	0.13
**Hum_aug**	0.37	**0.43**	0.37	-0.01	-0.22	0.12	0.25	0.19	0.12
**T_may**	0.03	-0.03	-0.23	-0.26	-0.05	-0.13	-0.26	-0.28	0.26
**T_jn**	0.13	-0.03	-0.18	-0.31	-0.31	-0.13	-0.15	-0.08	-0.10
**T_jl**	-0.31	-0.21	-0.05	0.28	0.38	-0.10	0.18	0.21	-0.23
**T_aug**	**-0.49**	**-0.49**	-0.23	0.26	0.15	0.18	-0.15	0.13	-0.26
**Prec_jn**	0.05	0.31	0.26	0.08	0.23	-0.26	0.18	0.00	-0.08
**Prec_jl**	**0.49**	**0.49**	**0.44**	-0.10	0.05	0.03	0.31	-0.13	0.41
**Prec_aug**	-0.08	-0.13	-0.13	-0.10	0.05	0.08	-0.15	-0.13	-0.26
**Wind_my**	-0.03	0.08	0.08	0.21	0.39	-0.10	0.16	0.03	0.00
**Wind_jn**	-0.09	0.12	-0.06	-0.04	0.22	-0.01	0.12	-0.12	0.37
**Wind_jl**	-0.12	-0.01	-0.17	-0.14	0.04	0.17	-0.09	-0.32	0.37
**Wind_aug**	**-0.45**	-0.09	-0.04	0.22	0.32	0.04	-0.01	-0.14	0.14
