# Supplementary material for: Dendrochemical Challenge in Climate Science: Whether Chemical Elements in Wood Reflect the Fluctuations in Weather Parameters
Source: Plants (Basel). 2022 Nov 25;11(23):3240. doi: 10.3390/plants11233240 (PMC9740880; doi:10.3390/plants11233240)
Supplement: Supplementary file 1 [file plants-11-03240-s001.zip › Table S25.pdf]

Table S25. Scots pine #1. Initial core. After alcohol extraction. After HCl extraction.

inner border of the bark

pith

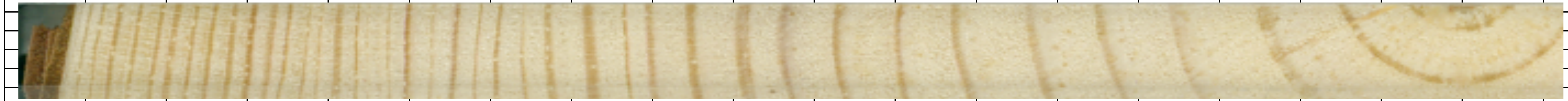

position (mm	Al	P	S	Ti	Mn	Fe	Cu	Zn	Ca	Sr	K
18.01	0	6	19	35	88	77	0	0	86	1093	37
18.11	9	4	0	6	73	102	0	0	58	1132	29
18.21	15	10	27	0	76	106	0	0	159	791	35
18.30	13	0	17	0	82	132	0	0	1465	956	49
18.41	7	0	37	26	110	119	0	0	6405	1028	109
18.51	33	0	34	0	87	242	0	0	10905	752	148
18.61	15	0	71	0	77	130	0	0	11974	809	196
18.71	14	11	44	0	111	153	0	0	13207	885	266
18.81	20	0	38	14	121	111	0	51	15529	847	316
18.91	14	0	50	0	154	142	0	77	13635	754	359
19.01	11	6	70	18	181	85	0	104	10657	978	316
19.11	24	0	52	9	200	137	0	47	9345	708	327
19.21	25	8	22	0	223	177	0	92	9283	525	307
19.31	21	0	52	0	241	705	15	131	8973	591	315
19.41	5	9	56	0	220	415	96	39	9758	545	348
19.51	14	25	65	0	122	188	95	275	11173	429	431
19.61	0	14	45	19	168	91	98	48	11175	670	523
19.70	0	0	27	21	195	104	51	168	11063	513	601
19.81	14	0	52	32	145	100	0	100	8925	311	670
19.90	0	20	40	0	115	0	112	92	8742	330	715
20.01	10	0	32	0	123	106	108	46	9135	493	752
20.11	24	22	59	0	177	87	0	114	8162	513	733
20.21	21	11	28	4	175	80	0	118	7068	587	760
20.31	14	0	40	0	175	67	0	105	7502	712	1100
20.41	19	7	68	20	204	122	16	124	9424	563	1941
20.51	13	7	82	24	246	111	42	61	12175	757	2816
20.60	18	19	81	0	271	74	79	122	13766	469	3769
20.71	32	44	98	0	333	153	121	284	14901	443	4209
20.81	13	33	51	0	343	83	139	220	14066	464	4730
20.91	19	24	91	0	302	65	189	190	12212	450	4875
21.01	0	36	68	0	278	141	37	186	10202	260	4572
21.11	0	17	43	0	272	114	158	232	7786	278	3986
21.20	38	41	67	0	214	66	137	173	5303	315	3329
21.31	0	12	48	0	178	89	148	166	3422	188	2537
21.41	0	0	34	0	174	100	128	234	1977	356	1649
21.51	0	0	17	0	109	83	101	170	1465	239	1103
21.61	12	5	42	0	85	43	92	121	1514	398	1000
21.71	10	0	49	0	144	112	86	179	1494	205	873
21.80	0	29	35	0	92	108	140	132	1444	390	942
21.91	6	24	18	0	92	85	88	91	1565	338	984
22.00	0	16	6	0	96	46	141	107	1590	220	873
22.11	0	4	33	0	98	91	124	178	1488	178	886
22.20	16	5	19	0	115	69	281	214	1413	239	887

22.31	25	6	24	0	132	53	11	180	1394	296	924								
22.41	6	8	34	0	137	83	145	77	1534	493	948								
22.51	8	19	55	0	152	72	100	122	1475	238	816								
22.60	22	0	40	0	134	111	173	18	1645	234	823								
22.71	12	18	42	0	177	140	136	141	1703	225	861								
22.80	4	13	45	0	129	147	201	89	1658	280	773								
22.91	5	34	38	0	135	94	222	166	1600	347	858								
23.00	8	20	20	0	136	31	140	138	1772	0	952								
23.11	0	12	37	0	113	91	210	228	1717	185	820								
23.21	17	13	47	0	126	35	221	102	1580	139	679								
23.31	19	21	17	0	119	89	209	213	1379	133	655								
23.41	11	12	37	0	122	86	191	131	1450	280	681								
23.51	8	20	29	0	95	53	230	124	1647	381	717								
23.61	0	0	23	0	128	110	60	0	1868	312	651								
23.71	18	10	45	0	124	146	152	65	1943	225	708								
23.80	14	28	70	0	162	112	198	140	2103	274	856								
23.91	0	14	35	0	166	113	193	161	2178	74	980								
24.01	10	12	39	0	128	65	214	151	2119	152	896								
24.11	9	27	9	0	135	44	282	134	1918	40	705								
24.20	0	33	32	0	99	67	186	177	1768	137	607								
24.31	4	32	31	0	133	38	182	172	1545	203	708								
24.40	18	0	27	0	148	110	204	183	1477	240	758								
24.51	0	28	38	0	115	84	179	75	1403	169	676								
24.61	17	19	28	0	145	30	109	35	1503	233	607								
24.71	13	33	47	0	165	49	68	0	1534	325	543								
24.81	0	16	42	0	106	72	128	128	1676	636	623								
24.91	21	24	37	15	145	63	126	0	1887	600	580								
25.01	10	6	24	37	97	63	134	158	2047	367	650								
25.11	0	20	40	0	152	42	98	112	2075	302	759								
25.21	14	6	33	0	155	34	266	210	1992	102	672								
25.31	0	0	33	0	154	69	281	279	2103	277	708								
25.41	16	0	27	0	139	89	273	292	1931	157	636								
25.50	16	14	28	0	134	12	165	133	1818	110	585								
25.61	0	0	37	0	155	80	193	202	1775	52	601								
25.70	21	35	26	0	105	66	142	243	1941	48	660								
25.81	13	4	37	0	134	105	186	137	1919	223	613								
25.90	0	17	37	0	121	63	152	38	2045	184	692								
26.01	0	15	34	0	175	52	155	125	2038	33	786								
26.11	39	32	17	0	121	68	215	262	1880	77	790								
26.21	0	0	24	0	133	42	238	137	1844	0	646								
26.30	7	13	27	0	153	0	132	126	1651	60	576								
26.41	0	5	15	0	125	65	94	145	1746	76	534								
26.51	0	22	40	0	95	55	132	215	1721	187	542								
26.61	29	42	35	0	114	56	76	63	1727	381	492								
26.71	20	0	20	26	120	90	138	43	1870	365	554								
26.81	0	0	25	0	151	54	49	26	2138	524	547								
26.91	24	0	22	0	128	73	176	176	2172	330	585								
27.01	4	4	30	0	141	76	149	263	2134	282	681								
27.11	5	10	42	0	152	65	258	120	2010	68	714								
27.21	0	0	22	0	154	102	189	181	1902	137	717								
27.31	0	0	35	9	164	54	156	222	1868	36	654								
27.40	5	10	34	0	133	72	209	156	1661	50	510								
27.51	0	4	30	0	196	98	91	212	1777	209	524								
27.61	14	8	33	0	146	110	0	19	1772	137	512								
27.71	23	0	45	0	171	128	90	0	2012	539	483								
27.81	21	18	31	0	152	172	159	205	2112	171	598								
27.91	8	37	37	0	207	66	75	113	2168	151	643								
28.01	6	0	15	0	125	125	278	249	2072	54	636								

28.10	13	0	16	0	199	65	247	338	1884	0	585								
28.21	19	0	25	0	118	86	256	213	1810	33	519								
28.31	0	28	49	0	116	75	167	196	1652	181	514								
28.41	6	22	41	0	132	78	190	205	1634	261	495								
28.51	14	23	53	0	190	123	150	140	1589	53	463								
28.61	0	24	37	0	96	167	39	100	1637	456	472								
28.70	13	0	9	0	150	99	55	58	1836	319	501								
28.81	18	13	35	0	113	85	52	121	2068	526	538								
28.91	0	8	40	0	160	110	48	194	2121	354	596								
29.01	0	0	35	0	105	91	197	212	2308	124	642								
29.11	0	22	37	0	170	76	112	311	2166	198	601								
29.21	0	10	28	0	163	36	209	175	1967	52	545								
29.31	0	21	32	0	166	38	231	357	1898	56	653								
29.41	0	9	39	0	134	88	252	161	1686	126	538								
29.51	0	10	28	0	171	73	206	61	1624	191	456								
29.61	0	10	41	0	101	73	12	146	1753	333	460								
29.70	27	20	34	0	118	72	56	17	1808	293	433								
29.81	12	16	59	0	159	75	121	78	2098	336	557								
29.91	9	23	17	0	190	100	175	192	2278	230	632								
30.01	6	7	46	0	195	97	222	124	2527	278	690								
30.11	6	39	49	0	143	47	134	121	2469	63	734								
30.21	8	12	30	0	143	62	214	212	2350	0	674								
30.31	0	21	40	0	160	55	267	275	2197	70	507								
30.41	0	16	36	0	184	99	202	423	1946	94	457								
30.51	20	19	41	0	90	67	120	210	1858	104	424								
30.61	6	17	44	0	154	46	157	202	1898	241	430								
30.71	0	8	20	0	176	116	147	37	2068	276	439								
30.81	7	5	35	0	166	81	0	20	2320	434	560								
30.91	0	17	31	0	152	105	71	108	2495	159	585								
31.01	0	17	27	0	107	116	102	293	2540	311	542								
31.11	0	7	34	0	203	0	240	171	2307	318	529								
31.21	0	0	29	0	169	25	228	350	2183	205	492								
31.31	23	0	25	0	232	62	149	150	1946	85	450								
31.41	0	9	29	0	146	65	186	221	1859	80	458								
31.50	0	6	5	14	180	44	186	139	1944	72	476								
31.61	25	12	40	0	169	91	87	118	2055	389	508								
31.70	0	0	28	0	145	85	18	81	2177	291	494								
31.81	19	5	27	0	169	89	146	150	2239	177	553								
31.90	10	17	24	0	200	117	191	81	2393	346	624								
32.01	15	17	19	0	167	45	267	296	2264	121	592								
32.11	18	0	23	0	207	53	190	248	2071	99	475								
32.21	13	12	34	0	230	85	234	224	1795	198	431								
32.31	14	16	23	0	137	83	126	188	1680	278	373								
32.41	9	0	18	0	151	83	180	94	1705	340	388								
32.51	0	7	38	0	165	135	58	160	1807	248	510								
32.61	0	26	46	0	182	122	100	93	1917	423	392								
32.71	0	0	43	0	155	114	26	59	2123	358	468								
32.81	0	0	37	0	178	104	143	83	2273	472	514								
32.91	8	11	10	0	202	71	44	124	2490	391	566								
33.01	6	7	34	6	147	56	94	243	2439	225	564								
33.11	28	12	28	0	157	126	131	187	2346	172	624								
33.20	21	28	35	0	165	88	232	197	2240	306	639								
33.31	0	12	25	14	196	68	161	283	2066	156	659								
33.40	0	19	52	0	160	89	172	217	1858	285	582								
33.51	0	9	20	0	184	100	114	199	2067	228	497								
33.61	11	9	5	0	171	104	99	86	2271	315	695								
33.71	33	32	50	0	156	106	168	139	2508	268	833								
33.81	7	38	27	0	192	79	199	235	2448	339	817								

33.91	0	19	57	0	191	17	250	281	2151	0	623								
34.01	13	6	8	0	154	158	143	166	1881	200	455								
34.11	0	0	16	0	149	95	93	30	2053	309	471								
34.21	9	6	37	0	200	97	102	115	2161	493	447								
34.31	0	25	39	0	172	99	97	207	2276	411	458								
34.40	14	13	37	0	188	97	35	180	2488	150	533								
34.51	10	20	44	0	229	65	132	23	2487	289	559								
34.60	0	19	40	0	187	77	124	195	2411	199	503								
34.71	11	9	11	0	181	21	253	367	2010	0	475								
34.81	9	22	13	0	88	0	134	329	1715	20	389								
34.91	13	25	23	0	165	57	175	213	1621	0	424								
35.01	20	6	35	0	142	60	192	190	1583	0	388								
35.10	8	29	36	0	143	57	146	156	1760	284	377								
35.20	14	0	39	0	162	34	121	88	1940	261	419								
35.31	14	7	27	0	199	79	112	82	2115	419	451								
35.41	0	31	38	0	174	119	62	122	2301	370	455								
35.51	0	13	42	0	129	78	112	74	2326	322	447								
35.60	0	0	15	0	149	93	57	127	2567	427	617								
35.71	0	0	45	0	194	56	124	244	2504	203	745								
35.81	0	0	9	0	173	13	276	250	1999	0	667								
35.91	13	17	17	0	132	90	150	180	1926	58	594								
36.01	0	9	40	33	149	106	68	134	1971	293	404								
36.11	0	0	52	82	125	117	104	150	2101	463	527								
36.20	9	14	19	62	142	115	106	60	2377	512	700								
36.31	27	18	30	0	145	89	142	216	2381	259	751								
36.41	10	0	44	0	174	57	210	71	2040	79	627								
36.51	10	0	33	0	165	48	131	128	1964	17	477								
36.61	11	0	19	28	154	99	73	124	1910	471	414								
36.71	0	12	24	10	197	137	142	63	2116	447	492								
36.81	0	12	28	0	199	143	105	100	2422	451	509								
36.91	0	15	7	0	212	163	44	86	2678	526	681								
37.00	0	0	8	0	190	115	171	119	2530	248	636								
37.11	13	11	16	0	134	50	175	152	2369	323	494								
37.20	0	0	37	0	219	82	89	33	2501	379	521								
37.31	15	11	46	0	152	126	132	61	2747	306	717								
37.41	20	17	28	0	188	77	156	203	2548	146	697								
37.50	4	5	19	0	197	96	100	333	2172	225	519								
37.61	0	32	22	0	151	56	162	195	2135	0	429								
37.70	23	16	49	0	223	250	149	232	2301	394	476								
37.81	8	5	39	7	185	148	62	125	2342	334	463								
37.91	20	20	36	0	162	99	80	125	2312	444	519								
38.01	6	27	33	0	217	136	0	180	2458	382	522								
38.11	13	17	39	0	190	164	30	187	2628	430	570								
38.21	18	17	59	0	194	109	84	131	2956	371	753								
38.31	0	0	32	0	241	132	124	154	3060	298	747								
38.40	5	14	19	0	180	58	190	386	2489	144	570								
38.51	0	0	39	0	187	0	208	256	2103	100	484								
38.61	13	15	5	0	186	78	236	221	1993	0	439								
38.71	11	6	26	0	109	77	133	181	2120	438	387								
38.80	0	12	45	0	153	47	106	217	2071	32	491								
38.91	9	0	29	0	191	77	20	93	2011	128	422								
39.00	0	18	16	26	126	129	58	60	2104	399	415								
39.11	7	23	24	0	173	136	72	87	2276	457	445								
39.21	8	7	15	0	213	46	43	136	2465	239	538								
39.31	0	5	39	0	131	124	106	117	2568	411	550								
39.41	0	0	23	0	161	95	93	31	2708	496	518								
39.50	13	0	36	0	205	142	137	46	3050	428	647								
39.61	0	25	47	0	171	112	147	90	3384	324	875								

39.71	11	0	29	0	188	79	207	234	3005	117	823								
39.81	0	17	22	0	166	6	238	163	2418	191	623								
39.91	13	20	24	0	220	55	150	205	2283	191	464								
40.01	14	0	20	11	153	57	109	21	2104	485	396								
40.11	0	0	0	0	201	63	101	90	2124	260	419								
40.20	0	0	18	0	154	71	8	0	2284	395	517								
40.31	0	0	31	5	187	120	80	43	2313	227	508								
40.41	0	0	23	0	185	51	80	162	2501	384	511								
40.50	16	0	38	6	225	85	82	123	2643	371	503								
40.61	0	10	21	0	210	0	0	110	3012	303	627								
40.71	12	9	16	0	229	141	139	51	3206	420	590								
40.81	11	0	46	0	171	60	91	188	3020	230	609								
40.91	0	0	31	0	213	129	278	196	2947	0	616								
41.01	0	0	0	0	169	5	284	289	2518	241	538								
41.11	8	0	9	0	241	70	131	252	2425	0	432								
41.21	8	0	40	0	203	90	58	131	2316	8	471								
41.31	0	25	21	0	219	87	114	119	2343	363	467								
41.41	0	23	42	0	214	123	94	35	2431	316	463								
41.51	9	7	34	0	224	104	57	261	2294	365	505								
41.61	28	20	16	0	201	116	80	205	2381	357	479								
41.71	0	22	45	0	208	98	94	123	2313	500	487								
41.80	0	12	18	0	215	122	147	109	2543	382	546								
41.91	0	16	31	0	211	112	125	137	2546	399	595								
42.01	0	16	22	0	178	56	147	123	2572	62	546								
42.11	32	8	28	0	169	17	140	156	2509	100	511								
42.20	4	7	25	0	206	47	262	104	2299	129	504								
42.31	0	12	31	10	177	281	79	149	2176	186	477								
42.41	0	0	6	0	218	192	25	197	2358	325	504								
42.50	10	19	33	0	212	82	136	163	2334	252	515								
42.61	11	0	18	0	160	64	154	65	2564	486	472								
42.71	0	8	19	0	201	42	121	82	2667	477	479								
42.80	0	14	27	0	195	96	19	125	2737	434	558								
42.91	27	17	24	0	244	80	86	184	3070	517	689								
43.01	14	0	24	28	230	43	190	125	3124	236	801								
43.11	10	31	48	0	186	55	203	362	2577	0	656								
43.21	0	0	16	17	136	61	174	382	2053	0	555								
43.30	14	12	22	0	163	92	194	190	2038	190	554								
43.41	0	21	26	0	188	73	154	187	1949	84	447								
43.51	0	0	20	0	193	45	136	89	1887	227	421								
43.61	10	19	46	0	201	131	69	119	2081	440	420								
43.70	7	0	41	0	164	80	43	23	2045	371	439								
43.81	0	0	13	0	154	83	89	73	1975	575	447								
43.90	10	11	25	0	163	139	112	193	2069	469	465								
44.01	0	4	30	0	207	64	46	33	2275	551	483								
44.11	5	9	41	0	173	96	59	67	2335	546	520								
44.20	14	0	37	0	184	64	149	87	2668	399	621								
44.31	5	18	35	0	243	112	178	129	2962	282	769								
44.41	21	10	27	0	169	73	145	256	2566	232	729								
44.50	10	8	25	0	142	62	256	247	2026	0	571								
44.61	6	12	17	0	179	18	115	247	2196	155	481								
44.71	14	0	30	0	178	80	133	185	2299	219	543								
44.81	19	7	5	0	186	107	138	201	2477	366	609								
44.91	18	7	25	0	175	95	67	0	2565	440	527								
45.01	0	4	20	0	174	111	101	152	2539	451	444								
45.10	0	0	12	0	209	35	67	162	2604	395	517								
45.21	24	7	37	0	186	118	162	125	2522	382	536								
45.31	0	0	31	0	178	116	173	77	2446	330	534								
45.41	7	7	33	0	223	58	103	162	2634	392	593								

45.51	11	14	31	0	206	108	102	157	2969	314	635								
45.61	0	10	19	0	171	118	251	248	2766	234	703								
45.70	16	16	27	0	169	43	171	279	2542	80	883								
45.81	10	0	27	0	198	123	182	278	2155	32	817								
45.91	15	5	20	0	165	104	105	261	1740	74	627								
46.01	17	16	22	0	135	15	151	199	1665	122	555								
46.11	17	23	44	8	172	73	121	174	1661	89	420								
46.21	20	4	40	0	133	55	112	119	1879	323	471								
46.31	26	36	21	0	137	62	95	75	2141	269	483								
46.41	0	23	30	0	146	54	165	35	2226	267	588								
46.51	0	0	44	0	200	84	18	178	2233	280	512								
46.61	29	28	30	0	188	93	36	0	2243	337	512								
46.71	0	9	14	0	198	129	103	60	2373	490	574								
46.81	0	14	26	0	201	73	41	71	2269	398	574								
46.91	26	16	32	0	147	68	64	208	2400	501	545								
47.01	28	26	23	0	186	147	97	154	2445	537	560								
47.11	13	0	5	0	201	142	182	139	2519	386	576								
47.20	0	8	33	0	218	113	226	25	2678	503	571								
47.30	0	21	27	0	173	48	78	122	2727	368	557								
47.41	4	23	32	0	180	110	164	159	2789	376	575								
47.50	0	13	41	0	233	53	93	185	2966	483	653								
47.61	36	7	37	0	229	126	233	192	2881	222	654								
47.71	16	9	35	0	263	87	256	227	2512	273	649								
47.80	16	25	34	15	170	38	282	360	2070	44	518								
47.91	0	9	41	0	182	38	210	300	1769	0	538								
48.01	0	19	26	0	139	73	180	99	1822	0	513								
48.11	0	5	14	0	123	28	192	212	1786	0	537								
48.20	27	10	36	0	103	56	116	159	1880	109	608								
48.31	26	10	38	0	165	119	160	101	1965	230	633								
48.41	11	0	26	0	131	140	100	79	2031	191	645								
48.51	0	16	37	0	175	90	108	43	1939	409	532								
48.60	19	0	29	0	189	187	67	78	2131	404	573								
48.70	0	6	50	0	196	178	75	131	2389	488	588								
48.81	17	0	52	0	203	134	163	117	2398	582	597								
48.90	13	0	39	0	214	157	140	124	2454	429	693								
49.01	7	7	21	0	215	158	160	131	2547	282	599								
49.11	10	0	17	0	191	136	207	107	2893	201	620								
49.21	16	0	9	0	253	80	87	143	3348	447	574								
49.31	13	0	5	0	204	70	137	148	2867	526	604								
49.41	0	15	23	0	198	87	56	136	2753	362	597								
49.51	21	0	40	0	186	60	30	216	2682	445	682								
49.61	15	7	55	0	213	130	27	149	2922	537	721								
49.71	26	0	6	0	223	74	142	181	3383	235	1009								
49.81	0	28	35	0	264	58	210	306	3334	156	1008								
49.91	18	8	23	0	210	57	236	348	2663	0	790								
50.01	16	0	8	0	180	113	214	220	2427	71	741								
50.11	36	11	35	0	192	76	204	291	2218	142	608								
50.21	28	20	26	0	216	82	152	110	2252	467	566								
50.31	0	5	25	0	193	45	112	113	2358	349	554								
50.41	0	0	63	0	181	55	158	125	2369	136	621								
50.51	0	0	0	0	113	48	38	0	2371	343	638								
50.61	31	18	46	0	183	85	63	10	2442	374	628								
50.70	5	32	26	0	248	81	167	86	2422	381	607								
50.81	10	24	52	0	166	108	179	196	2637	271	761								
50.91	0	8	20	0	201	75	150	194	3111	318	968								
51.01	0	0	23	0	169	24	181	207	2810	90	851								
51.11	0	25	35	0	168	77	279	250	2313	78	720								
51.21	31	14	51	0	124	4	244	308	1967	38	626								

51.31	20	0	12	0	123	18	233	325	1857	0	617								
51.40	0	13	5	0	179	101	114	216	1906	41	664								
51.51	27	0	15	23	127	34	185	219	2121	279	703								
51.61	0	13	24	0	157	38	106	8	2204	400	833								
51.71	0	12	37	0	180	35	98	109	2236	363	791								
51.81	13	25	32	0	228	87	115	208	2313	279	729								
51.91	16	0	23	10	213	30	62	75	2369	394	611								
52.01	0	12	17	15	168	51	133	207	2415	398	690								
52.10	30	0	39	0	174	91	74	153	2511	475	668								
52.21	9	0	30	0	222	105	115	289	2571	480	748								
52.31	11	6	25	0	195	93	107	209	2571	450	750								
52.41	10	0	12	0	192	77	157	90	2746	245	700								
52.51	15	25	23	8	215	98	170	132	2597	373	688								
52.61	9	15	18	0	177	147	215	207	2740	492	662								
52.71	6	0	5	0	207	84	124	129	2792	312	686								
52.81	0	11	45	0	170	30	156	107	3030	515	679								
52.91	56	33	48	0	203	57	110	229	3154	141	739								
53.01	7	0	19	0	166	29	75	194	3474	213	791								
53.11	14	0	32	0	192	86	260	273	2944	112	717								
53.20	35	23	7	0	215	15	256	283	2517	0	574								
53.31	5	11	34	0	180	22	225	310	2313	56	561								
53.41	24	15	34	0	204	59	134	297	2232	0	541								
53.51	16	32	32	0	170	24	162	161	2211	112	522								
53.61	16	15	15	0	134	22	224	280	2144	0	469								
53.71	10	25	30	0	161	71	128	107	2283	251	472								
53.81	0	23	39	0	135	39	144	152	2326	198	542								
53.91	0	12	21	0	182	44	164	152	2363	342	469								
54.01	14	0	35	0	160	59	37	48	2484	369	532								
54.11	11	0	27	0	188	117	42	136	2600	288	510								
54.21	0	0	38	0	183	78	39	163	2569	506	520								
54.31	26	11	42	0	211	104	194	118	2628	329	497								
54.41	5	11	23	0	188	50	185	243	2797	277	513								
54.50	10	26	24	0	208	97	53	58	2649	499	432								
54.61	12	11	10	0	224	20	73	175	2749	472	497								
54.70	11	4	25	0	241	113	134	229	2697	543	505								
54.81	12	21	41	0	211	95	91	159	2753	472	499								
54.91	9	16	38	0	206	124	69	146	2781	168	516								
55.00	8	12	27	0	220	115	194	229	2836	384	507								
55.11	12	4	28	0	202	81	72	97	2803	150	513								
55.21	13	0	43	0	227	125	118	117	2795	415	457								
55.30	9	8	27	0	163	131	205	181	2840	497	562								
55.41	5	16	7	20	225	101	126	170	3057	400	483								
55.51	17	0	24	0	244	84	183	159	3569	161	638								
55.60	5	0	15	0	203	35	119	267	3499	226	613								
55.71	20	22	32	0	250	80	235	204	3060	43	555								
55.80	0	18	21	11	256	20	225	230	2779	0	505								
55.91	0	25	26	0	201	0	195	344	2557	12	477								
56.01	11	11	13	0	159	59	299	295	2575	202	484								
56.10	13	15	37	0	185	26	178	193	2749	54	457								
56.21	17	18	40	0	183	4	191	194	2740	55	395								
56.31	0	0	22	0	205	42	179	170	2365	179	421								
56.41	14	9	0	0	170	48	194	204	2422	260	454								
56.51	0	22	16	0	145	40	87	161	2506	146	431								
56.61	12	0	28	0	187	72	43	103	2550	276	443								
56.71	12	20	31	0	156	97	135	244	2704	397	428								
56.81	0	0	19	0	210	54	114	102	2874	194	461								
56.91	0	17	30	11	254	89	103	158	2951	496	475								
57.00	16	0	30	0	219	99	8	159	2983	152	486								

57.11	10	6	27	0	245	58	85	68	2745	368	477								
57.21	12	19	21	29	200	84	108	127	2819	544	468								
57.31	0	0	19	0	220	135	144	190	2830	430	460								
57.40	9	9	33	0	236	117	88	193	3023	303	509								
57.51	10	28	24	18	176	82	90	53	3087	303	469								
57.60	21	0	24	0	226	90	90	167	3441	157	571								
57.71	13	0	16	40	242	84	169	79	3739	375	561								
57.81	9	18	44	0	253	92	123	252	3923	206	620								
57.91	0	11	40	0	185	35	129	305	3599	173	578								
58.01	6	22	32	0	163	73	191	367	3308	117	553								
58.11	0	0	17	0	257	15	242	322	2941	85	587								
58.21	9	25	44	0	254	47	222	270	2705	80	514								
58.31	0	13	16	0	218	101	142	213	2595	178	466								
58.41	18	0	23	0	193	12	224	151	2610	145	464								
58.51	18	0	31	11	162	28	100	164	2539	11	480								
58.61	0	14	40	5	133	35	32	121	2496	22	452								
58.70	0	0	23	0	172	68	91	181	2331	113	451								
58.81	0	0	16	0	156	58	113	249	2191	325	428								
58.90	19	27	37	0	188	17	118	175	2313	325	434								
59.01	5	21	27	0	209	114	69	108	2476	346	462								
59.11	17	9	34	0	181	82	96	45	2556	198	451								
59.20	14	16	25	0	144	81	67	166	2612	286	464								
59.31	9	0	27	0	258	44	229	129	2638	171	478								
59.40	15	19	38	0	201	27	42	144	2622	285	495								
59.51	12	14	35	0	275	95	64	149	2670	379	481								
59.61	7	0	20	0	210	76	25	94	2726	350	464								
59.70	28	24	47	0	218	73	105	90	2805	211	481								
59.81	0	10	27	0	185	56	163	140	2860	472	500								
59.90	11	26	27	0	231	33	98	135	2765	183	467								
60.01	27	25	37	0	185	60	152	123	2960	400	480								
60.11	0	0	32	0	190	101	193	113	2873	183	553								
60.21	0	13	16	0	250	62	201	109	3082	361	486								
60.31	0	9	20	0	242	83	175	93	3133	444	512								
60.41	0	18	29	12	231	144	111	96	3267	167	532								
60.51	0	0	46	0	211	88	135	252	3507	294	621								
60.61	14	24	24	0	241	80	163	215	3638	302	603								
60.71	0	0	22	5	232	80	102	301	3393	237	614								
60.81	0	0	13	0	235	36	233	284	2760	119	584								
60.91	14	16	38	0	217	45	215	352	2496	0	518								
61.00	23	26	41	0	131	0	175	164	2327	40	445								
61.11	5	4	21	0	219	87	246	191	2192	136	471								
61.21	15	12	25	0	207	45	168	270	2215	170	437								
61.31	0	11	29	0	176	81	173	195	2181	0	431								
61.41	0	34	27	0	200	43	133	175	2206	74	458								
61.50	7	0	17	0	127	65	93	223	2262	113	395								
61.61	21	0	32	0	223	26	56	201	2323	502	434								
61.71	0	0	5	0	176	31	140	102	2401	217	479								
61.81	0	4	35	0	180	27	101	34	2626	454	460								
61.91	4	11	24	0	240	121	160	203	2788	251	477								
62.01	19	6	35	0	211	81	151	203	2687	299	444								
62.11	0	0	15	0	206	58	151	38	2619	246	474								
62.20	0	8	28	0	191	52	130	92	2722	215	439								
62.31	30	15	13	0	213	73	201	147	2722	483	409								
62.41	23	17	28	26	184	109	57	160	2733	234	421								
62.51	13	31	30	0	187	80	222	122	2783	541	461								
62.61	0	7	14	0	200	83	136	144	2891	313	494								
62.70	0	12	44	0	175	126	116	77	2888	381	503								
62.81	17	8	15	0	243	93	0	207	3061	430	544								

62.90	0	0	16	19	279	120	149	156	3088	393	497								
63.01	24	25	24	0	250	101	149	110	3162	267	471								
63.11	17	0	17	0	193	85	119	155	3133	245	551								
63.20	0	10	41	0	208	114	112	170	3157	436	571								
63.31	13	9	10	0	185	110	84	64	3186	252	534								
63.40	0	8	15	0	209	85	74	113	3247	484	552								
63.51	24	0	33	0	166	30	64	111	3263	498	597								
63.60	13	11	26	0	253	122	120	46	3467	363	500								
63.71	0	0	28	0	225	155	105	57	3731	446	612								
63.81	0	17	50	0	272	95	62	221	4136	126	741								
63.91	0	7	14	0	276	110	183	262	3783	124	724								
64.00	0	6	17	0	232	73	291	281	3261	75	601								
64.11	0	19	16	0	177	103	328	454	2816	104	554								
64.21	0	4	23	0	244	66	327	333	2472	0	498								
64.31	25	0	26	0	181	54	195	267	2399	0	472								
64.41	4	5	14	0	186	110	70	218	2501	27	464								
64.51	21	6	22	0	167	115	36	210	2615	98	513								
64.61	0	11	24	0	165	134	70	186	2709	297	411								
64.71	10	0	34	0	159	55	200	58	2635	374	448								
64.81	0	8	9	0	233	105	127	106	2610	433	469								
64.91	10	0	6	0	168	97	127	169	2685	392	435								
65.01	0	0	25	0	201	85	54	168	2836	225	478								
65.10	9	0	28	0	205	97	175	59	2902	225	508								
65.21	0	30	32	0	222	97	83	249	2830	133	474								
65.31	0	0	32	0	169	124	69	184	2910	162	513								
65.40	0	0	24	0	239	119	81	76	2796	226	494								
65.51	0	16	26	0	248	99	130	184	2859	488	460								
65.61	0	0	16	0	181	85	112	85	2926	220	521								
65.70	29	11	29	0	217	74	122	190	2865	334	518								
65.81	0	0	0	0	200	74	62	0	2942	371	477								
65.91	5	28	42	0	231	88	80	70	2959	289	524								
66.01	0	0	36	0	255	63	105	156	2968	509	542								
66.11	6	22	24	34	238	135	151	51	3106	417	505								
66.21	0	12	17	0	243	70	62	149	3007	404	467								
66.31	6	14	20	0	179	91	96	114	3110	545	525								
66.41	0	0	38	0	226	98	173	131	3218	304	527								
66.51	0	0	25	0	264	86	173	57	3184	313	515								
66.61	25	7	42	0	274	37	18	214	3445	530	592								
66.71	13	0	35	0	241	95	119	70	3831	303	617								
66.81	4	0	27	0	221	46	121	201	3800	189	604								
66.91	0	14	29	0	204	47	178	179	3335	220	590								
67.00	0	0	12	10	244	85	194	321	3058	263	609								
67.11	19	17	18	0	228	57	227	160	2851	108	548								
67.21	20	0	35	0	202	25	203	252	2661	103	533								
67.31	12	9	43	0	189	60	164	271	2456	0	481								
67.41	0	9	17	0	184	49	235	289	2554	163	487								
67.50	23	0	9	0	168	40	263	338	2474	0	440								
67.61	31	20	20	0	149	67	124	125	2459	78	451								
67.71	8	26	18	0	166	12	188	189	2550	179	463								
67.81	11	17	29	0	160	87	225	160	2610	246	457								
67.91	16	30	32	0	149	105	121	184	2673	266	435								
68.00	0	15	19	0	234	73	156	182	2767	291	490								
68.11	0	0	19	0	215	69	135	192	2883	320	477								
68.21	4	6	36	27	176	92	23	127	2720	344	553								
68.31	0	0	28	0	216	110	63	137	2790	417	502								
68.41	28	0	29	0	187	48	64	118	2943	248	479								
68.51	8	15	20	0	211	79	59	172	3164	358	535								
68.61	13	10	44	0	250	78	82	100	3000	238	505								

68.71	0	10	31	0	179	37	149	105	2884	310	468								
68.81	5	10	33	0	188	59	234	227	2972	398	519								
68.90	11	0	28	0	170	67	184	189	3095	218	544								
69.01	10	0	21	0	214	77	147	103	2987	152	512								
69.10	0	0	14	0	247	74	75	118	3046	300	568								
69.21	0	15	29	0	182	51	91	194	2997	283	512								
69.31	0	22	26	0	188	44	194	147	3027	391	570								
69.41	11	0	18	0	246	81	221	185	3079	424	527								
69.51	7	6	23	0	187	19	165	101	3061	400	510								
69.61	6	0	11	0	242	76	170	68	3006	184	532								
69.71	28	15	53	7	204	75	126	121	3112	498	513								
69.81	6	0	7	0	185	46	126	84	2989	382	518								
69.91	5	0	24	0	213	122	108	54	3137	449	548								
70.01	11	0	37	0	167	97	106	167	3147	310	532								
70.10	23	6	26	0	221	110	110	183	3288	304	521								
70.21	7	0	12	0	263	59	118	39	3353	322	560								
70.30	0	0	31	0	233	112	93	199	3420	423	578								
70.41	9	21	12	0	241	133	90	78	3581	287	571								
70.51	0	8	29	0	264	166	132	194	3564	114	609								
70.61	18	11	26	0	250	139	140	124	3779	309	630								
70.71	23	8	16	0	227	112	140	185	3706	237	647								
70.81	15	0	26	0	243	52	158	296	3507	348	625								
70.91	0	21	30	0	280	67	191	293	3271	0	638								
71.01	10	8	31	7	201	122	119	191	3084	18	552								
71.10	0	13	0	0	229	32	208	307	3029	27	522								
71.21	18	24	39	0	186	0	127	288	2885	0	584								
71.31	16	18	24	0	233	11	159	243	2754	161	532								
71.41	36	14	24	0	198	41	277	253	2527	0	501								
71.51	8	4	15	0	174	63	167	244	2597	40	486								
71.61	26	17	12	0	183	70	184	251	2585	92	491								
71.71	12	0	15	0	199	82	222	243	2756	177	516								
71.81	6	6	11	0	185	155	202	130	2886	147	502								
71.91	8	0	31	0	207	73	151	194	2861	62	491								
72.01	5	15	19	6	203	7	115	126	2882	236	499								
72.11	12	0	8	0	214	48	156	157	2943	231	468								
72.20	0	6	16	0	221	57	65	204	3097	434	547								
72.31	0	0	29	0	228	120	188	177	3239	232	526								
72.41	4	0	5	0	273	114	195	114	3297	335	494								
72.51	0	6	42	15	201	106	53	133	3148	77	555								
72.61	14	14	12	0	236	128	130	90	3293	163	491								
72.70	5	10	34	0	258	107	34	66	3388	393	512								
72.81	10	6	42	0	211	82	156	65	3472	422	528								
72.90	0	20	34	0	231	78	173	109	3563	268	610								
73.01	14	12	31	0	257	121	147	151	3528	160	549								
73.11	13	7	29	0	303	123	123	107	3559	179	484								
73.21	0	0	42	0	227	61	86	82	3628	469	586								
73.31	21	16	14	0	260	43	67	131	3470	360	567								
73.41	17	0	39	0	230	95	166	47	3627	300	606								
73.50	0	9	37	0	260	114	100	105	3742	441	524								
73.61	22	15	33	0	193	112	140	96	3630	326	558								
73.71	0	0	46	0	302	129	114	131	3588	349	599								
73.80	0	11	19	0	276	95	107	198	3741	609	601								
73.91	6	0	40	0	241	147	167	102	3728	479	615								
74.00	19	5	7	0	214	140	148	95	3767	39	545								
74.11	10	6	37	0	243	122	158	139	3761	317	605								
74.21	12	33	28	0	285	127	118	148	3832	418	603								
74.31	27	20	31	0	267	70	126	232	3824	465	625								
74.40	0	5	20	0	268	104	151	199	3684	102	607								

74.51	0	12	27	0	221	31	165	189	3646	246	663								
74.61	0	14	30	0	260	27	240	285	3488	167	634								
74.71	10	11	28	0	245	13	156	278	3432	101	586								
74.81	15	0	25	0	259	61	200	243	3348	120	593								
74.91	15	7	25	0	270	0	156	154	3305	35	614								
75.01	21	9	27	0	201	54	215	215	3170	64	528								
75.11	7	0	12	0	224	41	198	165	3143	97	562								
75.20	26	15	44	0	256	81	237	186	2944	72	541								
75.31	0	8	22	0	202	80	214	189	2945	15	499								
75.40	12	13	23	0	228	24	145	266	2980	0	536								
75.51	26	30	19	0	178	46	138	125	3000	71	542								
75.61	0	15	21	0	227	28	100	189	3167	222	513								
75.71	40	0	14	0	238	70	160	168	3146	151	528								
75.80	0	0	11	10	176	24	73	154	3222	249	489								
75.91	10	5	18	0	205	80	156	21	3054	334	562								
76.00	0	8	26	0	203	41	126	157	3254	276	547								
76.11	0	0	13	0	213	50	104	162	3156	191	541								
76.21	0	5	25	0	285	102	122	90	3315	187	560								
76.31	17	0	29	0	231	50	93	123	3514	185	548								
76.41	22	21	30	0	231	38	97	154	3392	190	543								
76.50	0	5	22	0	254	68	125	178	3438	269	548								
76.61	24	0	29	0	256	44	88	103	3549	219	569								
76.71	0	0	39	45	284	87	124	166	3540	411	560								
76.81	15	9	26	0	258	75	127	140	3591	421	598								
76.91	0	0	24	0	261	60	55	155	3650	277	548								
77.00	11	7	26	0	282	57	92	90	3639	461	549								
77.11	16	4	31	0	337	55	152	133	3699	362	588								
77.20	5	0	19	0	296	102	174	82	3783	267	519								
77.31	0	0	39	0	274	102	186	137	3845	337	557								
77.41	0	0	23	0	281	82	100	22	3812	219	648								
77.50	12	5	8	0	295	29	104	81	3768	221	552								
77.61	11	10	23	0	300	37	90	153	3820	236	579								
77.70	9	24	37	0	233	76	105	63	3891	237	586								
77.81	9	0	35	0	286	47	93	128	3877	157	621								
77.91	9	0	20	0	263	44	78	102	3956	247	644								
78.01	0	9	46	0	279	79	157	95	4056	229	571								
78.11	9	13	25	0	312	107	88	133	4301	427	634								
78.20	17	14	18	0	264	48	182	165	4294	278	664								
78.31	7	10	37	0	319	77	160	123	4264	346	636								
78.41	0	6	26	0	266	67	129	170	4383	291	639								
78.51	10	18	38	0	313	72	151	179	4290	189	665								
78.61	0	0	20	0	337	49	221	157	4256	205	721								
78.71	16	0	51	0	244	31	242	206	4282	101	687								
78.81	5	0	35	0	255	82	174	99	4005	0	684								
78.90	0	21	9	32	250	28	178	214	4024	178	637								
79.01	0	0	42	0	268	39	76	166	3925	0	643								
79.11	0	11	18	0	318	44	149	155	3985	126	609								
79.21	15	6	10	0	233	60	184	260	3772	65	621								
79.31	18	17	34	0	238	62	247	243	3673	188	630								
79.40	0	0	47	0	269	50	224	166	3806	93	630								
79.51	0	11	37	0	279	25	184	232	3725	129	641								
79.61	4	0	15	0	308	85	216	244	3514	148	650								
79.71	0	11	25	0	268	90	225	137	3591	156	603								
79.81	0	0	31	0	242	67	170	47	3799	177	569								
79.91	4	0	28	0	266	56	224	205	3705	186	570								
80.01	9	7	39	0	305	99	228	160	3581	385	567								
80.11	0	16	59	0	236	51	93	137	3459	70	569								
80.21	0	16	59	47	308	70	61	45	3489	48	639								

80.30	12	6	27	0	294	83	73	135	3640	128	587								
80.41	12	9	21	0	294	92	116	173	3728	273	552								
80.51	17	0	18	11	284	73	40	186	3874	313	577								
80.60	0	0	21	34	263	81	104	47	3835	213	573								
80.71	15	11	31	0	305	69	57	202	3746	393	606								
80.80	8	10	36	0	241	67	132	151	3720	224	605								
80.91	17	0	13	0	264	121	65	196	3758	391	607								
81.00	7	21	36	0	258	105	192	141	3716	214	581								
81.11	15	0	45	0	196	65	10	80	3865	483	569								
81.20	5	8	24	0	281	24	14	79	3857	399	596								
81.31	30	19	27	0	315	78	125	0	3976	256	616								
81.41	15	29	31	0	255	103	106	132	4163	496	625								
81.51	14	26	39	0	318	50	94	56	4079	356	654								
81.60	8	0	28	0	293	112	156	150	4138	566	650								
81.71	22	18	34	0	268	78	100	93	4280	180	641								
81.81	0	14	22	6	292	100	64	204	4376	317	676								
81.91	23	0	21	0	280	76	180	121	4500	164	653								
82.01	14	0	19	0	282	55	77	125	4380	392	661								
82.11	0	0	45	0	276	72	166	162	4398	358	671								
82.21	7	0	24	0	291	107	107	198	4384	231	717								
82.31	0	0	28	0	328	70	109	138	4647	487	673								
82.40	8	9	34	0	331	68	188	121	4669	338	732								
82.51	10	4	22	11	312	79	123	201	4628	361	733								
82.61	21	0	50	0	353	44	67	108	4738	321	769								
82.71	0	0	37	0	318	60	74	57	4780	317	673								
82.81	8	16	38	0	370	85	71	192	4926	327	737								
82.90	14	22	51	0	290	73	124	232	4916	137	727								
83.01	29	29	31	0	331	79	268	176	4932	308	727								
83.10	11	9	35	0	299	40	100	222	4670	109	740								
83.21	0	0	22	26	318	60	142	161	4685	119	710								
83.31	7	0	31	0	327	26	180	294	4658	105	688								
83.41	12	16	8	0	337	41	180	144	4641	206	721								
83.51	18	13	26	0	274	62	228	161	4559	111	723								
83.61	11	15	57	0	280	0	152	291	4582	23	645								
83.71	0	19	15	0	315	134	223	212	4365	58	646								
83.80	0	7	34	22	296	82	237	255	4378	273	619								
83.91	25	14	27	0	329	62	234	225	4150	168	588								
84.01	0	0	36	16	266	43	237	227	4211	295	645								
84.11	0	16	45	0	307	42	122	214	4027	0	668								
84.20	9	16	50	0	300	71	173	210	4246	55	633								
84.31	5	17	20	0	295	46	138	138	3975	278	565								
84.40	12	0	34	0	232	71	116	167	3954	174	637								
84.51	18	5	26	0	209	23	187	163	3916	15	566								
84.61	11	8	30	0	283	40	162	207	3897	77	545								
84.71	0	0	27	0	248	55	169	302	3713	133	579								
84.81	0	0	38	0	272	9	187	286	3798	164	578								
84.91	0	0	52	0	225	45	82	116	3895	217	614								
85.01	10	0	48	0	292	46	181	217	3917	113	607								
85.11	14	19	33	0	255	56	108	186	3810	121	538								
85.20	14	0	35	0	260	41	113	107	3768	164	578								
85.31	22	22	36	0	260	64	178	125	3853	204	580								
85.40	0	8	41	0	247	84	75	384	3887	87	607								
85.51	0	0	19	0	326	63	181	725	3837	78	612								
85.60	0	0	36	12	279	13	105	229	3916	133	543								
85.71	22	0	22	0	297	71	170	164	4098	236	573								
85.80	19	0	30	0	245	69	133	124	3985	218	562								
85.91	14	20	17	0	245	54	141	101	4041	309	594								
86.01	0	11	24	18	304	55	140	214	4121	189	615								

86.11	11	6	27	0	283	40	156	185	4160	123	646								
86.21	0	0	31	0	258	40	74	251	4330	94	621								
86.31	17	6	28	0	317	204	144	52	4286	335	640								
86.41	17	0	22	0	321	164	213	128	4278	308	634								
86.51	0	0	9	0	270	60	129	41	4331	207	554								
86.61	15	21	25	0	284	66	158	224	4558	67	617								
86.70	32	0	22	0	274	62	157	103	4411	347	634								
86.81	0	5	32	0	330	84	221	170	4618	420	651								
86.91	0	15	39	0	295	79	86	106	4690	226	612								
87.01	22	8	32	0	372	91	215	179	4583	117	643								
87.10	0	0	25	0	325	83	77	243	4806	288	636								
87.21	0	0	24	0	322	72	155	87	4742	271	608								
87.30	6	28	33	0	325	76	55	182	4712	271	683								
87.41	6	0	31	0	310	68	135	260	4797	231	671								
87.51	5	0	31	0	282	59	83	127	4689	46	698								
87.60	8	5	11	0	323	47	14	223	4725	342	610								
87.71	8	0	5	0	265	97	174	177	4779	241	655								
87.81	19	10	26	0	336	84	149	186	4628	206	633								
87.91	0	0	32	0	336	61	149	205	4928	86	642								
88.01	23	6	61	65	349	98	127	240	5160	270	694								
88.10	18	12	35	0	379	28	177	159	4952	282	647								
88.20	0	0	21	0	335	51	106	289	4996	47	644								
88.31	0	14	33	0	292	0	133	215	5040	208	679								
88.40	0	10	22	0	342	33	138	298	5136	203	616								
88.51	24	0	41	0	294	69	136	166	5055	201	650								
88.61	0	20	29	0	305	14	210	291	4923	174	634								
88.71	5	5	50	0	326	72	150	202	5042	369	645								
88.81	31	4	12	0	284	53	100	274	4848	245	636								
88.91	40	19	32	0	342	0	159	146	4767	213	611								
89.01	11	19	11	0	278	73	239	267	4807	254	609								
89.11	0	0	37	0	299	61	183	146	4542	130	645								
89.21	24	10	50	0	327	36	179	286	4582	177	608								
89.31	22	7	44	0	270	26	137	234	4711	436	594								
89.41	16	9	43	0	322	75	101	190	4512	324	609								
89.51	0	0	27	0	244	25	193	108	4542	205	657								
89.60	9	16	45	0	357	59	208	311	4491	184	608								
89.71	15	18	45	0	264	45	120	201	4620	212	607								
89.81	11	0	35	0	305	48	161	127	4534	266	675								
89.91	14	32	39	0	327	51	153	178	4655	161	639								
90.01	26	13	30	0	320	66	133	311	4489	304	661								
90.11	0	6	23	0	284	53	222	119	4521	341	631								
90.20	7	0	41	0	281	110	159	178	4743	288	638								
90.31	12	0	21	0	274	89	208	214	4534	94	606								
90.41	0	0	5	0	284	67	125	192	4372	168	578								
90.50	18	10	38	0	341	56	156	209	4536	158	606								
90.61	5	0	49	0	334	35	109	143	4697	52	696								
90.70	13	15	45	6	354	94	228	251	4796	166	660								
90.81	30	22	33	0	323	66	184	224	4798	291	637								
90.90	22	25	61	0	351	91	50	153	4730	254	673								
91.01	17	0	27	0	294	84	180	168	4641	145	648								
91.11	26	0	30	0	270	61	221	228	4727	155	659								
91.21	7	6	29	0	304	97	227	311	4789	350	599								
91.31	0	0	24	0	315	108	221	160	4511	286	653								
91.41	0	14	22	0	357	15	190	121	4578	196	663								
91.51	0	0	27	0	341	15	109	216	4764	29	631								
91.61	5	0	47	0	311	63	134	176	4783	148	639								
91.71	0	0	25	0	331	47	103	198	4794	225	660								
91.80	12	0	21	0	321	72	142	251	4968	237	701								

91.91	9	0	34	0	313	41	267	363	4919	206	686								
92.01	19	18	22	0	329	62	141	260	5009	331	695								
92.10	20	23	35	0	290	73	202	184	4909	109	665								
92.21	17	19	54	0	320	0	100	220	4765	270	688								
92.31	0	8	29	0	343	55	151	90	4811	518	693								
92.40	11	13	40	0	333	50	212	165	4902	280	743								
92.51	20	0	32	0	339	40	211	147	5074	124	725								
92.61	0	11	47	0	361	46	196	255	5062	288	703								
92.71	9	7	35	0	343	77	207	245	4922	293	737								
92.80	17	7	38	0	320	33	161	284	4944	386	689								
92.91	16	16	20	0	314	95	162	160	5304	319	679								
93.01	10	34	48	0	370	79	135	191	5284	190	682								
93.11	9	11	34	0	321	69	191	217	5144	231	676								
93.21	7	0	40	0	329	72	208	203	5012	192	684								
93.31	0	0	0	0	340	51	203	482	4882	115	676								
93.40	8	0	29	0	381	73	143	299	5011	38	654								
93.51	0	0	35	0	360	94	274	306	4941	454	677								
93.60	6	0	28	0	404	28	213	185	4912	15	663								
93.71	23	9	37	0	354	65	188	213	4852	238	668								
93.81	25	8	29	0	327	40	124	241	4939	154	656								
93.91	30	0	30	0	345	42	106	303	4709	0	616								
94.01	0	8	35	0	322	88	162	277	4816	104	690								
94.11	18	16	25	0	332	211	183	254	4800	154	596								
94.21	0	0	21	0	283	65	110	194	4700	529	650								
94.31	0	0	36	0	323	73	126	204	4753	137	673								
94.41	0	0	11	0	320	81	250	278	4674	131	646								
94.51	0	0	29	0	328	104	129	205	4678	191	616								
94.60	0	0	25	0	323	51	169	134	4715	283	653								
94.71	29	0	42	0	301	60	147	231	4592	94	637								
94.80	0	0	24	0	297	76	243	206	4626	200	615								
94.91	18	33	37	8	307	192	113	374	4704	165	583								
95.00	20	0	21	0	299	86	221	194	4613	89	623								
95.11	14	7	19	18	283	32	205	207	4445	55	622								
95.20	30	18	14	0	328	100	148	260	4599	228	592								
95.31	0	0	25	0	318	64	137	307	4586	66	629								
95.40	0	0	14	0	391	70	201	237	4567	303	648								
95.51	0	14	31	0	338	45	78	133	4862	129	698								
95.60	0	0	21	0	323	86	157	256	4813	0	617								
95.71	0	25	10	0	287	79	173	258	4825	166	578								
95.81	18	19	27	0	301	104	115	212	4693	275	592								
95.91	22	0	31	0	283	64	207	227	4748	134	644								
96.01	0	0	41	0	339	66	205	249	4686	213	584								
96.11	10	0	30	0	300	7	168	275	4525	265	591								
96.21	8	27	43	0	302	98	32	179	4593	175	617								
96.31	0	11	43	0	326	64	244	186	4662	284	696								
96.41	19	0	53	0	280	125	150	153	4718	307	618								
96.51	0	6	34	0	319	48	99	131	4650	67	676								
96.61	33	8	52	0	395	121	189	262	4475	314	643								
96.70	26	37	46	0	349	79	168	190	4409	113	629								
96.81	9	0	28	0	341	74	209	145	4546	215	603								
96.90	21	21	8	0	347	44	163	63	4553	46	585								
97.01	11	0	36	0	305	99	163	236	4779	172	596								
97.11	10	6	41	0	353	52	156	206	4649	254	615								
97.21	5	0	0	0	368	73	125	281	4577	103	580								
97.31	10	0	39	11	295	77	170	261	4431	318	602								
97.41	0	0	10	0	369	78	129	139	4586	379	618								
97.51	0	0	33	14	280	56	150	203	4500	220	642								
97.61	0	12	29	0	305	30	63	197	4685	94	612								

97.70	14	0	31	0	304	36	110	156	4454	339	567

Scots pine #1. After alcohol extraction.

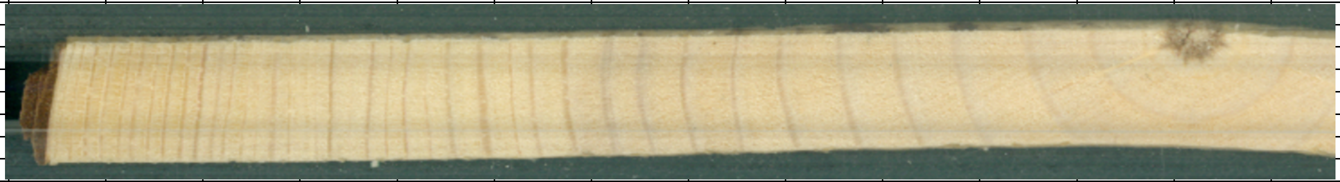

position (mm	Al	P	S	Ti	Mn	Fe	Cu	Zn	Ca	Sr	K
93.81	38	0	7	23	140	164	373	331	339	0	104
93.91	71	26	0	34	101	67	320	338	343	0	94
94.01	46	21	13	13	98	70	247	288	364	81	127
94.11	47	5	13	0	125	86	336	157	329	182	127
94.21	61	8	9	0	127	79	198	323	334	60	100
94.30	67	22	12	0	210	109	297	259	317	20	109
94.40	53	5	17	0	114	38	227	249	367	0	125
94.50	60	15	0	15	77	74	340	374	302	0	113
94.61	62	27	0	33	186	82	309	333	312	0	103
94.71	48	20	6	40	174	67	436	285	321	0	115
94.80	72	15	5	19	113	100	279	237	313	80	88
94.91	58	22	8	0	131	128	310	157	333	166	92
95.00	62	0	0	9	166	104	276	261	350	208	97
95.11	41	0	17	41	138	100	229	279	364	209	70
95.20	45	24	0	22	170	102	326	313	372	0	86
95.31	54	36	5	32	49	45	279	400	335	0	113
95.40	62	31	14	0	80	125	268	390	323	0	97
95.51	70	20	13	21	103	13	454	308	345	0	143
95.61	63	11	0	0	140	68	342	392	307	0	99
95.71	36	7	0	29	96	41	416	440	318	90	86
95.81	59	19	28	24	99	64	350	313	294	0	70
95.91	48	0	0	0	121	123	231	225	321	138	53
96.01	46	22	10	7	128	67	239	354	420	169	90
96.11	54	19	9	19	129	56	256	253	429	340	81
96.21	62	0	0	7	194	94	316	422	451	137	113
96.31	71	32	8	26	104	88	355	285	481	200	120
96.41	46	7	8	24	123	120	305	326	490	32	113
96.51	74	30	19	0	133	84	306	330	397	42	109
96.60	47	32	18	0	124	80	372	315	365	0	113
96.71	63	15	0	28	161	78	328	277	345	0	72
96.80	52	0	0	13	113	30	297	363	296	0	74
96.90	89	30	8	24	96	89	326	123	368	149	81
97.00	44	0	0	0	140	140	267	140	354	122	86
97.11	42	0	0	21	154	103	242	155	391	198	67
97.21	33	7	0	0	116	88	306	127	367	171	78
97.31	44	0	0	4	142	106	258	243	415	75	79
97.41	92	34	20	0	196	88	144	280	438	226	110
97.50	53	16	11	8	167	92	383	378	399	0	90
97.60	50	22	14	0	80	51	310	292	427	0	109
97.71	77	23	13	0	173	37	301	362	411	0	111
97.81	34	26	39	0	112	43	327	315	355	76	112
97.91	71	24	5	23	140	69	296	264	355	0	92

98.01	46	8	31	0	130	61	230	234	331	277	61								
98.11	57	14	10	0	168	133	293	360	390	42	70								
98.21	55	10	6	51	138	112	206	186	403	17	107								
98.30	43	34	15	7	157	26	215	369	416	104	90								
98.41	21	34	13	33	198	86	265	306	401	0	88								
98.51	42	18	25	0	106	77	253	295	410	0	104								
98.61	72	12	8	38	183	97	449	409	354	91	85								
98.70	63	25	8	0	145	67	224	391	338	0	73								
98.81	60	5	0	26	99	125	318	280	312	0	68								
98.91	72	10	0	22	169	113	336	280	400	148	75								
99.00	44	33	5	11	165	160	159	216	427	89	97								
99.11	89	13	0	46	157	107	234	272	464	246	111								
99.21	69	6	0	23	154	121	154	232	523	70	74								
99.31	48	0	12	0	181	88	329	215	409	59	79								
99.40	68	0	23	10	212	0	246	289	465	0	111								
99.51	67	23	0	17	145	49	344	506	462	25	114								
99.61	45	10	5	19	153	57	317	409	418	0	111								
99.70	59	26	5	8	133	97	346	90	448	0	98								
99.80	73	0	0	16	139	85	217	230	378	0	61								
99.90	74	16	0	14	148	133	257	238	404	271	85								
100.00	57	9	0	6	136	147	175	274	433	235	42								
100.11	58	0	0	20	164	78	259	176	445	159	74								
100.21	40	32	0	12	166	113	252	278	407	163	102								
100.31	53	25	0	0	153	100	363	420	453	0	98								
100.41	71	24	12	0	138	35	252	355	457	73	99								
100.51	47	40	11	0	207	15	374	398	428	0	97								
100.61	69	34	17	11	109	0	358	450	385	0	56								
100.71	44	8	13	13	130	0	285	377	325	0	76								
100.81	57	38	9	13	160	104	152	314	406	0	58								
100.91	59	0	0	35	87	150	246	244	393	69	79								
101.01	36	0	0	22	108	182	274	243	416	163	69								
101.11	49	0	0	15	199	191	203	225	397	336	87								
101.20	58	0	0	0	138	151	238	221	454	291	84								
101.31	40	19	5	21	152	118	265	186	443	240	95								
101.41	63	23	13	0	154	39	209	246	474	22	88								
101.51	37	23	0	24	195	90	306	349	437	0	113								
101.61	55	17	19	31	161	53	407	492	424	0	82								
101.71	46	14	10	0	135	72	409	455	383	0	71								
101.80	79	25	4	0	170	42	193	362	396	0	86								
101.90	63	0	0	28	128	90	375	277	340	0	63								
102.00	58	17	0	0	106	109	203	197	343	108	76								
102.11	40	0	6	0	145	139	236	286	402	269	95								
102.20	58	0	0	13	156	74	297	100	431	171	62								
102.31	35	0	5	8	175	126	213	278	503	68	115								
102.41	40	14	12	0	127	72	320	381	501	16	78								
102.51	63	19	16	24	247	77	429	323	509	0	123								
102.60	82	28	14	6	113	31	279	408	429	0	94								
102.71	49	32	6	35	189	72	301	388	429	0	93								
102.81	70	10	0	19	214	38	341	441	381	0	75								
102.90	44	9	5	0	151	91	214	298	378	0	82								
103.00	56	30	0	0	168	148	192	98	417	249	92								
103.10	61	14	16	12	163	197	262	150	427	187	80								
103.20	69	16	11	12	174	120	196	182	449	0	108								
103.30	50	0	0	0	217	63	289	219	509	68	104								
103.41	63	0	19	26	215	67	241	334	535	131	88								
103.51	50	14	18	10	187	24	408	378	505	0	96								
103.61	79	0	0	0	71	90	356	395	428	0	83								
103.71	51	25	27	25	186	88	439	475	439	0	92								

103.81	75	5	8	0	161	56	329	333	381	0	68								
103.91	27	21	0	0	152	52	285	280	341	0	82								
104.01	33	9	0	40	136	46	250	278	442	75	72								
104.10	45	14	7	13	98	118	250	176	437	198	99								
104.21	61	0	19	0	174	147	220	183	513	291	85								
104.31	44	0	14	61	181	62	234	222	469	167	90								
104.40	66	33	0	17	180	49	199	306	488	33	123								
104.51	72	0	0	0	152	0	415	503	431	47	91								
104.61	43	15	13	14	173	72	307	287	324	49	81								
104.70	62	13	31	19	96	126	260	308	392	0	49								
104.80	55	12	5	0	202	107	265	233	358	141	61								
104.90	42	11	7	0	199	210	116	204	368	358	85								
105.00	51	0	0	8	168	293	191	171	515	262	58								
105.10	32	10	0	26	159	177	131	112	454	365	59								
105.21	49	0	0	30	185	157	229	112	503	380	60								
105.31	35	0	16	0	185	139	178	154	543	55	86								
105.41	55	10	6	0	136	146	248	328	526	46	98								
105.51	47	7	0	22	156	92	293	378	531	52	91								
105.60	62	4	0	8	144	8	389	378	472	0	82								
105.70	77	22	0	26	159	89	339	272	514	0	87								
105.81	52	19	0	0	120	222	179	278	492	285	117								
105.90	42	0	18	20	181	171	191	148	487	155	72								
106.01	44	16	0	43	202	91	176	242	559	153	108								
106.10	68	12	18	0	200	62	263	189	566	117	73								
106.20	60	34	20	18	237	77	244	267	551	0	122								
106.30	52	17	5	0	212	71	288	461	509	0	87								
106.41	54	14	19	26	231	92	240	394	424	0	70								
106.51	77	26	8	18	176	165	204	271	455	172	82								
106.61	17	4	28	0	175	210	202	169	523	90	77								
106.71	46	0	0	0	222	203	261	284	525	374	93								
106.81	44	0	18	28	155	121	213	170	569	176	104								
106.91	44	8	0	0	244	155	238	204	542	164	125								
107.01	64	5	4	10	187	100	307	259	550	128	122								
107.11	60	17	0	48	120	20	334	344	494	0	124								
107.21	47	17	10	0	183	36	349	459	328	0	51								
107.31	52	20	12	0	141	35	358	392	290	0	90								
107.40	63	41	6	9	150	0	289	315	329	0	44								
107.51	44	17	0	0	150	63	252	366	325	200	73								
107.61	57	8	4	34	135	117	308	235	426	227	86								
107.71	65	19	17	18	164	165	63	192	421	76	95								
107.81	41	0	0	38	160	125	285	145	480	101	74								
107.91	38	6	0	0	173	105	220	0	483	80	82								
108.00	69	0	0	8	138	170	414	211	502	218	101								
108.10	46	9	0	45	160	155	263	245	542	254	127								
108.21	52	11	0	0	133	22	336	404	484	0	65								
108.31	49	15	17	17	138	56	353	445	432	0	70								
108.41	35	26	0	46	154	36	304	224	388	0	69								
108.51	38	0	0	0	151	80	215	165	413	247	87								
108.61	63	6	10	28	204	90	196	294	460	230	91								
108.70	57	0	0	20	199	112	223	250	563	121	118								
108.81	65	0	27	0	267	111	321	344	599	0	91								
108.91	48	6	7	11	182	39	221	397	420	0	76								
109.01	38	12	23	13	160	91	196	271	469	165	32								
109.11	68	0	0	0	198	194	200	160	470	144	71								
109.20	59	0	5	27	223	123	173	239	551	324	81								
109.30	41	5	0	33	203	192	218	283	504	97	81								
109.40	63	14	0	0	186	107	252	131	606	217	115								
109.51	62	31	22	23	181	83	201	310	624	28	102								

109.60	47	21	36	23	225	87	415	265	503	0	91								
109.70	30	0	11	19	221	124	361	288	479	92	62								
109.80	67	0	18	0	212	105	330	265	613	0	114								
109.91	31	15	12	0	200	21	362	337	571	0	102								
110.01	52	30	9	64	186	68	184	239	426	0	116								
110.11	70	5	0	24	162	113	292	343	437	0	80								
110.21	65	0	0	0	196	114	206	300	467	113	89								
110.31	94	8	25	16	173	123	207	336	511	100	85								
110.41	39	5	0	10	157	146	271	274	525	361	93								
110.50	71	0	0	0	228	138	249	296	505	153	82								
110.60	67	13	0	28	189	83	253	231	547	163	89								
110.71	74	9	0	23	211	154	300	282	657	358	107								
110.80	79	21	23	16	228	42	348	466	638	0	139								
110.90	44	8	18	0	188	0	348	464	496	0	77								
111.00	45	30	0	0	150	9	382	365	402	0	66								
111.10	53	28	0	16	139	57	290	366	389	90	62								
111.21	67	17	14	12	175	46	173	145	451	45	82								
111.31	55	0	15	0	142	59	228	332	434	133	70								
111.41	46	20	20	23	196	122	247	172	436	251	71								
111.51	47	14	0	17	159	72	249	199	501	344	82								
111.61	51	0	0	0	221	73	269	247	468	257	91								
111.71	62	23	18	25	155	151	210	208	491	100	88								
111.81	34	7	6	56	214	146	127	210	528	121	101								
111.90	10	0	0	0	220	161	284	363	527	151	112								
112.00	70	35	18	27	192	107	329	241	594	289	102								
112.11	70	17	21	0	239	39	262	338	671	0	149								
112.21	78	40	31	43	192	45	347	395	600	0	125								
112.31	57	17	5	0	222	86	285	347	436	31	108								
112.41	50	0	0	13	210	74	220	244	473	9	81								
112.50	58	34	0	0	171	165	160	229	463	369	59								
112.61	61	19	0	54	194	100	290	88	485	151	96								
112.71	59	0	0	67	182	110	314	219	538	96	102								
112.81	72	0	11	0	172	218	247	213	508	285	97								
112.91	66	0	0	0	162	129	259	220	581	164	111								
113.01	65	0	0	22	212	85	219	207	568	227	105								
113.11	35	5	14	0	189	137	329	178	613	33	98								
113.20	50	23	20	71	216	140	386	421	675	0	115								
113.30	65	45	14	6	174	0	289	525	576	0	93								
113.41	36	25	30	0	229	96	303	525	501	0	70								
113.51	58	23	34	0	149	6	277	351	479	0	82								
113.61	35	17	0	40	167	38	280	341	439	47	83								
113.71	54	0	8	0	207	86	224	286	513	144	69								
113.81	55	12	0	14	218	74	264	197	503	211	95								
113.91	25	12	13	24	184	145	228	214	549	298	82								
114.01	50	0	0	16	195	117	76	187	568	234	75								
114.11	58	35	0	28	198	71	312	212	587	115	66								
114.20	41	9	0	0	221	149	292	199	567	258	91								
114.31	45	17	0	0	217	87	155	301	455	144	111								
114.40	65	24	15	0	205	70	303	298	599	0	101								
114.51	32	0	8	28	230	80	383	410	485	0	56								
114.61	58	5	8	0	187	0	316	261	427	87	89								
114.71	70	0	18	6	224	157	188	436	467	0	87								
114.81	26	0	0	16	228	365	180	292	461	28	58								
114.91	40	0	0	0	181	163	214	238	472	221	99								
115.00	38	0	0	23	175	92	198	139	515	29	116								
115.11	61	13	6	33	172	165	127	227	587	298	93								
115.21	54	49	16	30	229	138	153	196	577	155	122								
115.31	28	0	0	5	230	64	217	214	614	220	94								

115.40	65	21	0	22	242	51	345	324	682	0	113								
115.50	65	33	26	0	228	63	381	442	648	46	142								
115.60	52	27	14	0	247	76	292	430	464	0	126								
115.70	51	27	13	0	171	56	304	459	381	0	84								
115.80	50	18	8	0	129	35	159	366	400	0	86								
115.90	73	23	5	31	187	137	194	165	377	36	92								
116.01	50	0	0	31	185	54	158	238	461	108	128								
116.10	42	0	0	0	175	153	175	220	448	65	98								
116.21	58	4	23	0	150	122	236	152	437	244	77								
116.31	27	0	0	15	123	120	199	189	505	250	75								
116.41	65	19	0	15	224	102	128	133	488	299	99								
116.50	37	0	15	6	186	135	268	260	466	162	73								
116.60	42	12	0	0	168	98	178	214	487	80	89								
116.70	67	0	0	19	136	93	213	249	582	308	112								
116.80	42	7	0	20	188	42	241	361	622	0	113								
116.90	58	19	0	27	153	25	521	456	537	0	82								
117.01	47	6	0	0	164	34	459	492	434	0	80								
117.11	80	35	31	25	188	75	276	402	428	0	100								
117.21	69	19	0	0	132	121	364	322	445	214	79								
117.31	54	18	0	0	173	134	261	230	532	113	105								
117.41	53	11	0	36	228	64	184	163	540	117	75								
117.51	31	0	0	0	180	109	134	267	543	171	78								
117.61	54	0	21	0	219	95	278	293	586	191	86								
117.70	65	0	0	8	213	151	149	239	493	153	107								
117.81	27	9	0	15	194	64	225	236	550	48	102								
117.91	69	0	8	11	220	112	225	257	582	258	79								
118.01	34	0	0	40	247	80	198	251	598	210	129								
118.10	22	7	7	12	221	38	307	393	614	41	109								
118.20	68	21	37	0	203	47	403	442	555	21	133								
118.30	68	22	0	0	169	11	286	437	473	0	135								
118.40	66	24	5	34	176	77	284	342	386	0	100								
118.51	61	23	0	0	114	45	330	305	401	0	79								
118.61	38	12	0	8	114	53	211	360	352	0	76								
118.71	46	14	0	10	186	38	134	251	337	0	87								
118.81	43	19	0	26	137	159	201	155	425	100	88								
118.91	55	25	0	10	175	112	324	172	478	5	72								
119.00	38	0	0	16	210	154	216	179	465	151	94								
119.11	42	13	8	0	187	95	317	124	492	283	100								
119.21	52	0	18	17	134	71	153	235	490	106	87								
119.30	60	0	0	0	151	81	248	238	510	192	102								
119.41	35	0	0	0	143	100	328	95	491	197	64								
119.50	64	0	12	0	213	107	191	249	566	170	123								
119.61	53	19	4	22	190	109	122	248	605	116	93								
119.71	71	0	14	0	197	78	289	239	545	125	74								
119.81	16	9	0	10	179	122	275	181	593	331	100								
119.91	39	0	0	0	187	155	213	210	561	234	80								
120.01	26	7	0	43	249	125	229	85	605	408	104								
120.11	43	7	0	0	263	69	227	224	627	116	118								
120.20	65	32	24	20	181	99	253	330	544	26	93								
120.30	87	10	7	0	168	61	372	588	487	0	114								
120.40	54	25	0	0	152	23	305	467	315	0	128								
120.50	72	46	15	0	114	0	337	474	351	0	75								
120.61	47	17	14	13	124	23	372	387	332	0	54								
120.71	53	13	0	0	146	41	395	389	316	0	96								
120.81	82	38	15	16	150	4	220	295	395	0	105								
120.91	58	28	8	0	149	77	309	412	472	58	102								
121.01	81	17	0	0	187	130	119	255	416	150	64								
121.11	78	0	0	0	180	137	261	302	481	74	43								

121.20	58	7	10	13	203	85	171	150	470	53	77								
121.31	32	0	0	0	151	134	128	261	481	133	98								
121.40	48	0	0	15	147	116	186	307	523	191	78								
121.50	71	0	13	0	220	51	238	186	544	74	75								
121.61	37	16	0	48	202	79	393	198	566	332	112								
121.70	61	10	0	18	181	108	189	301	604	327	117								
121.81	44	0	0	0	207	103	332	258	554	270	111								
121.91	43	5	4	0	177	115	264	266	532	184	112								
122.01	56	8	0	0	134	59	186	283	563	104	89								
122.11	67	0	0	22	219	94	317	191	561	222	84								
122.21	61	13	0	16	181	84	257	266	618	208	125								
122.30	61	13	12	23	248	91	316	367	657	0	151								
122.40	58	23	0	0	256	29	331	617	578	0	127								
122.50	67	12	19	0	208	21	190	420	449	0	88								
122.61	66	14	29	0	188	38	262	380	462	107	111								
122.71	48	0	16	0	185	59	195	274	476	184	62								
122.81	49	0	0	0	211	107	314	198	417	161	95								
122.90	19	0	0	22	143	91	255	212	428	27	104								
123.01	49	0	0	42	253	109	241	338	492	112	92								
123.11	30	0	0	21	213	102	310	249	492	0	104								
123.21	50	8	0	45	201	150	138	201	521	15	92								
123.31	28	6	5	6	221	80	254	220	483	319	110								
123.40	75	12	15	28	197	110	177	272	647	143	140								
123.51	53	17	29	0	266	87	301	393	611	124	135								
123.60	64	9	22	28	96	23	232	428	511	0	128								
123.71	48	0	11	31	128	34	395	412	447	0	116								
123.81	69	16	9	0	150	34	310	364	401	0	83								
123.91	75	16	12	11	179	42	275	318	380	0	89								
124.01	60	0	12	11	153	103	235	445	374	0	105								
124.11	60	30	10	22	195	81	179	281	436	57	65								
124.21	59	0	0	23	147	80	87	294	489	0	101								
124.31	69	17	0	17	162	73	217	238	462	169	101								
124.41	42	0	0	0	154	113	209	259	455	74	85								
124.51	50	15	0	38	180	89	244	149	443	0	75								
124.60	74	0	0	31	190	24	212	294	521	135	76								
124.70	42	15	0	0	258	109	248	316	508	182	89								
124.80	49	0	0	0	180	79	204	235	462	0	118								
124.91	27	13	0	21	181	169	228	283	505	98	118								
125.01	47	9	29	28	159	58	184	265	540	174	95								
125.10	56	0	0	24	164	55	152	204	537	31	102								
125.20	66	29	6	27	193	77	279	226	558	303	113								
125.31	25	17	0	0	182	90	254	190	619	188	91								
125.41	46	8	0	0	236	103	169	242	542	150	139								
125.51	40	0	0	0	273	48	280	242	611	104	123								
125.61	52	25	16	0	174	54	197	394	623	0	148								
125.71	49	29	0	10	275	0	340	475	531	0	135								
125.81	59	38	29	12	207	87	210	484	447	0	95								
125.91	56	30	27	0	245	47	418	487	440	0	74								
126.01	52	10	0	0	137	51	228	333	419	0	92								
126.11	38	0	0	0	144	20	283	419	407	0	112								
126.21	36	13	6	7	185	29	221	341	409	0	89								
126.31	42	20	0	66	191	64	287	354	465	23	90								
126.41	50	43	44	57	193	83	175	295	510	0	62								
126.50	57	0	13	13	204	77	157	190	504	20	81								
126.61	60	17	0	24	235	131	202	234	491	165	85								
126.71	51	7	15	11	178	79	110	249	490	259	93								
126.81	70	16	12	20	213	66	180	245	538	136	125								
126.90	80	8	0	43	172	96	255	238	553	0	101								

127.01	29	0	0	46	245	86	239	331	607	251	74								
127.11	42	25	0	31	195	24	245	217	556	75	90								
127.21	51	0	0	31	228	127	167	193	558	290	102								
127.31	24	0	0	15	202	97	214	38	537	37	104								
127.41	48	15	0	0	191	99	281	289	562	165	72								
127.51	47	8	5	0	238	78	226	133	606	210	85								
127.60	20	0	0	38	210	94	160	298	605	181	75								
127.70	23	17	0	6	218	137	266	185	577	157	82								
127.80	43	0	0	0	250	89	284	216	554	260	82								
127.90	70	0	0	31	215	59	277	227	605	215	86								
128.01	50	0	19	38	228	78	298	287	608	114	93								
128.10	57	23	0	20	173	20	266	372	624	0	96								
128.21	44	10	12	9	220	0	372	489	592	0	73								
128.30	48	34	39	0	219	25	232	451	528	0	122								
128.41	58	14	9	0	204	9	266	450	490	0	83								
128.51	64	33	22	29	190	12	246	423	471	0	71								
128.61	64	20	0	0	188	47	226	368	479	0	98								
128.71	49	11	13	6	129	0	286	303	451	105	71								
128.81	64	22	20	16	171	0	284	335	419	0	72								
128.91	37	27	0	0	196	56	340	258	456	0	60								
129.01	58	0	0	0	193	13	166	291	464	0	73								
129.11	62	17	6	16	192	26	190	381	463	0	96								
129.21	43	6	18	27	156	58	196	233	476	67	82								
129.31	64	0	0	34	223	111	229	321	498	188	88								
129.41	38	0	0	15	190	89	276	351	541	209	73								
129.50	62	17	0	0	177	130	179	213	569	326	78								
129.60	25	0	0	26	170	61	113	256	516	135	85								
129.71	63	21	12	17	218	69	206	159	591	365	80								
129.80	20	36	0	17	181	35	228	161	529	250	89								
129.91	24	29	9	19	180	119	232	258	583	173	89								
130.01	32	13	10	0	208	105	318	247	565	384	121								
130.10	69	17	15	15	231	161	275	331	620	198	57								
130.21	53	0	0	0	273	92	343	289	717	250	106								
130.31	65	26	12	0	249	54	416	307	689	146	90								
130.41	47	0	0	25	219	67	360	382	703	20	89								
130.50	76	8	0	0	225	11	350	429	617	0	109								
130.61	39	16	10	14	192	0	269	478	630	0	59								
130.71	44	13	10	9	228	0	288	388	496	0	126								
130.81	59	23	4	0	191	47	306	332	497	0	109								
130.91	70	6	9	37	184	58	358	409	457	0	34								
131.01	48	14	0	0	250	7	422	352	420	0	104								
131.11	53	28	0	13	226	23	264	336	439	51	48								
131.21	72	14	12	14	197	80	286	293	420	73	77								
131.31	55	19	0	26	134	63	229	219	399	60	67								
131.40	48	21	5	7	231	72	117	301	453	47	92								
131.51	51	0	0	15	170	64	29	124	513	85	93								
131.61	66	0	0	9	176	131	215	330	510	0	90								
131.71	45	0	0	10	240	71	197	167	486	147	85								
131.80	45	0	9	0	177	71	176	242	563	31	113								
131.91	53	0	4	0	111	66	188	270	489	229	65								
132.01	36	0	0	20	192	122	301	338	537	99	35								
132.11	43	21	0	40	213	110	329	247	540	394	109								
132.20	70	6	10	0	222	24	169	226	584	144	96								
132.31	40	23	8	16	226	60	230	263	537	94	87								
132.41	47	0	0	0	227	188	240	301	601	179	126								
132.51	31	0	0	22	218	88	205	303	664	186	103								
132.61	46	0	0	53	244	77	278	250	630	60	74								
132.70	42	6	0	17	194	71	194	178	583	91	100								

132.81	62	0	0	21	218	29	224	392	642	178	95								
132.91	50	0	0	25	153	113	142	222	585	211	75								
133.00	39	0	12	33	267	38	254	305	652	132	96								
133.11	53	0	0	0	241	17	260	314	671	93	108								
133.21	43	12	14	0	298	18	248	427	625	0	118								
133.31	39	32	7	39	232	53	429	447	526	0	97								
133.40	45	17	23	0	231	33	317	465	551	0	111								
133.51	63	0	0	0	192	11	258	500	447	0	79								
133.61	60	24	25	10	185	27	355	496	433	46	51								
133.71	61	19	14	9	190	32	278	420	406	0	93								
133.81	43	26	0	0	168	0	278	343	440	0	67								
133.91	36	24	0	0	157	37	275	368	439	0	67								
134.00	63	7	0	9	176	72	118	287	452	0	72								
134.10	51	0	13	13	187	76	228	365	464	117	106								
134.20	56	16	8	0	105	47	157	268	504	121	73								
134.31	75	12	13	15	149	15	196	224	521	173	73								
134.41	65	0	0	35	214	89	238	262	586	7	107								
134.51	40	0	0	0	171	33	109	301	598	195	89								
134.60	52	18	33	0	204	26	255	243	629	157	104								
134.71	62	18	15	0	193	106	116	162	582	284	69								
134.81	69	5	6	0	206	85	220	147	596	61	75								
134.90	70	21	0	0	157	149	206	169	574	191	81								
135.01	53	9	0	0	166	84	206	204	584	332	80								
135.11	39	6	0	45	172	84	276	267	595	244	99								
135.21	55	8	0	0	157	145	264	230	599	108	111								
135.31	50	0	0	0	160	96	206	207	621	146	118								
135.40	59	0	0	26	185	176	207	195	621	180	90								
135.50	41	22	0	32	210	116	300	295	627	184	104								
135.61	38	0	23	0	207	85	202	171	630	328	116								
135.71	42	0	0	37	204	56	210	242	614	155	73								
135.80	13	0	7	43	225	102	175	197	659	0	58								
135.90	42	0	0	0	264	151	218	215	689	183	72								
136.00	61	10	5	0	255	77	102	209	610	285	95								
136.11	56	6	0	19	238	136	249	263	707	181	120								
136.20	50	0	0	31	217	74	207	359	737	180	71								
136.30	19	13	0	0	252	86	261	427	766	13	141								
136.40	62	43	5	11	299	0	248	360	678	36	150								
136.50	49	9	6	21	221	72	371	536	644	0	90								
136.61	50	19	0	34	208	20	275	394	542	0	83								
136.71	43	21	12	0	176	20	385	383	463	0	62								
136.81	64	6	0	0	215	77	212	503	424	0	82								
136.90	71	16	6	0	196	44	229	457	465	93	79								
137.00	51	6	11	66	174	106	206	315	482	0	80								
137.11	84	0	0	0	171	110	82	184	538	93	82								
137.20	60	0	20	0	254	49	182	263	524	46	63								
137.31	97	21	8	20	196	82	224	267	539	200	73								
137.40	44	11	0	41	186	46	189	288	499	210	62								
137.51	60	0	0	6	195	99	166	298	516	175	103								
137.60	37	0	17	7	213	111	169	242	552	293	77								
137.71	41	5	0	0	274	75	106	234	584	0	35								
137.81	51	0	0	36	194	48	279	300	580	0	86								
137.91	57	8	0	24	230	95	196	229	512	223	84								
138.01	44	0	8	0	213	68	305	329	548	64	84								
138.11	41	13	0	0	186	87	156	165	569	119	116								
138.21	51	0	0	39	193	73	233	269	533	282	106								
138.31	64	31	0	21	279	60	207	113	564	355	134								
138.41	51	16	0	16	216	78	119	257	613	295	135								
138.50	36	23	0	12	155	71	175	297	543	360	92								

138.61	44	0	0	12	247	101	216	237	582	192	113								
138.70	51	5	0	9	210	68	280	223	598	183	82								
138.80	59	12	0	0	223	136	159	206	671	191	81								
138.91	57	17	0	9	204	90	162	227	583	353	111								
139.01	51	0	18	0	189	89	218	259	626	273	88								
139.10	61	17	0	0	186	88	210	309	681	126	87								
139.21	62	19	0	26	258	69	171	272	676	255	88								
139.31	54	20	9	30	235	50	290	267	709	121	131								
139.41	31	0	0	18	268	31	334	347	753	0	98								
139.50	52	41	9	0	250	0	442	277	642	0	83								
139.61	47	7	0	45	216	55	300	402	577	0	89								
139.70	52	0	16	0	207	24	278	242	530	74	97								
139.81	58	37	0	22	168	48	391	459	476	0	64								
139.91	46	0	7	0	182	62	251	382	457	127	118								
140.01	30	0	0	0	190	14	273	387	465	0	42								
140.10	55	0	22	20	192	52	190	269	483	0	107								
140.21	50	27	8	8	179	15	287	410	479	0	73								
140.31	43	11	0	9	196	55	97	392	461	0	97								
140.41	35	0	0	10	175	50	231	277	472	96	72								
140.51	93	0	0	13	184	67	207	296	507	124	93								
140.61	53	0	0	15	223	89	271	283	546	0	66								
140.71	40	5	8	37	196	69	205	255	509	57	75								
140.81	52	13	16	0	143	70	267	244	493	119	83								
140.91	51	30	5	21	176	37	229	151	612	316	107								
141.01	49	25	0	0	250	124	217	287	610	190	86								
141.11	61	0	0	28	248	81	109	258	616	303	99								
141.20	68	6	0	11	258	71	278	352	597	94	108								
141.31	43	23	0	0	246	109	306	311	651	139	91								
141.40	45	0	0	0	210	88	309	211	645	132	128								
141.50	55	18	7	71	197	93	114	250	644	234	84								
141.60	51	14	0	25	170	83	264	181	599	91	95								
141.71	82	7	17	6	230	162	225	253	597	91	88								
141.81	63	5	12	0	221	120	269	233	637	0	86								
141.91	57	16	0	0	223	150	188	293	597	257	101								
142.00	53	10	0	28	204	49	201	352	611	214	85								
142.11	45	18	0	18	221	61	249	263	576	0	81								
142.21	78	29	0	18	175	54	145	348	589	89	106								
142.30	54	0	16	19	147	14	299	254	575	287	98								
142.40	48	18	0	0	221	78	312	238	593	106	107								
142.51	48	21	0	31	209	32	249	200	618	187	89								
142.60	62	0	0	12	191	73	265	220	620	150	95								
142.71	61	0	12	19	232	73	232	288	580	69	82								
142.81	43	16	7	0	235	80	326	210	635	226	53								
142.91	42	13	0	8	217	70	294	316	669	106	92								
143.01	34	6	0	0	208	47	237	392	689	62	110								
143.11	49	9	0	0	195	93	227	316	632	0	101								
143.21	39	10	0	44	244	39	257	252	767	116	109								
143.30	65	0	12	68	276	5	160	422	693	0	118								
143.41	59	28	0	0	240	0	342	454	662	214	98								
143.51	39	25	8	0	202	45	305	340	601	0	107								
143.61	51	33	11	29	198	60	184	354	612	0	110								
143.71	53	9	26	0	159	14	256	458	539	0	105								
143.81	54	28	17	5	200	16	257	472	543	0	79								
143.91	48	20	11	33	162	0	338	441	496	0	76								
144.01	81	37	33	0	228	0	324	441	494	120	69								
144.11	63	27	9	0	119	37	253	372	503	0	74								
144.21	18	34	18	26	144	35	294	359	459	0	85								
144.31	55	22	6	0	175	43	282	399	456	0	93								

144.41	27	19	5	23	137	0	236	377	466	0	97								
144.51	64	23	13	26	199	42	294	351	456	0	69								
144.60	57	24	0	0	188	63	271	363	516	0	83								
144.71	34	0	0	24	167	37	221	346	528	18	59								
144.81	62	17	0	0	238	74	243	227	525	144	113								
144.91	69	9	0	0	237	89	167	254	574	0	92								
145.00	56	28	5	50	240	83	220	338	626	0	114								
145.10	59	11	12	0	275	61	282	345	650	0	73								
145.20	40	15	10	10	209	83	262	359	672	195	105								
145.30	87	8	0	33	227	46	238	240	662	0	101								
145.40	36	6	0	27	260	121	211	312	744	165	88								
145.51	63	0	26	0	212	115	324	377	698	93	83								
145.61	74	11	13	0	282	94	200	219	682	156	91								
145.71	64	0	0	11	195	96	269	295	671	0	111								
145.81	61	5	16	5	229	46	279	310	682	184	99								
145.91	57	6	0	35	234	25	311	276	658	187	110								
146.00	74	0	9	14	197	84	258	360	634	0	104								
146.10	43	15	15	27	236	120	204	297	695	252	132								
146.20	43	0	0	18	205	153	266	233	685	149	87								
146.31	52	21	0	25	254	82	273	346	731	325	95								
146.40	29	0	0	45	238	61	172	217	696	216	116								
146.50	43	0	0	0	223	80	287	290	708	87	107								
146.61	64	0	0	43	248	76	302	207	663	299	115								
146.70	58	7	0	32	284	92	209	446	694	171	124								
146.81	69	28	0	13	283	83	247	302	691	83	74								
146.90	83	18	32	0	286	19	303	278	767	122	130								
147.00	63	0	15	0	286	62	364	293	764	73	127								
147.10	75	15	0	21	244	0	383	340	768	95	116								
147.21	50	11	8	22	250	11	320	358	634	39	104								
147.31	55	10	0	0	251	12	335	393	684	13	76								
147.41	57	24	0	11	236	25	210	467	631	0	101								
147.50	41	18	0	0	255	16	277	401	621	0	113								
147.60	57	0	0	0	214	0	240	380	633	0	111								
147.70	67	7	23	18	272	72	352	512	605	0	108								
147.81	46	8	10	0	235	0	225	374	552	0	105								
147.91	66	21	5	0	215	12	230	282	514	0	99								
148.01	52	34	11	7	206	14	386	366	570	0	93								
148.10	69	23	20	11	230	38	265	304	603	0	82								
148.21	33	15	0	44	230	107	293	257	623	15	94								
148.30	66	21	19	0	240	10	175	258	545	0	80								
148.41	51	0	0	43	209	76	229	379	588	169	81								
148.51	36	24	0	26	268	10	218	212	599	0	83								
148.61	67	8	0	0	202	25	250	337	573	64	88								
148.71	63	0	0	11	245	60	194	266	595	0	71								
148.80	39	18	0	45	237	5	344	387	688	138	70								
148.91	56	13	0	0	248	64	344	380	645	66	99								
149.00	36	40	15	0	242	64	204	174	663	67	78								
149.11	56	14	0	27	256	27	316	270	671	52	80								
149.21	74	6	4	11	252	89	261	302	652	205	105								
149.30	51	0	0	35	210	93	312	216	624	140	87								
149.40	60	14	9	7	220	73	209	286	633	61	108								
149.51	64	21	7	51	256	55	181	301	672	49	96								
149.61	43	15	8	0	256	70	296	239	703	31	99								
149.71	49	23	7	0	228	62	281	233	691	83	109								
149.81	62	31	0	0	268	60	213	259	675	194	119								
149.91	58	5	0	0	288	70	354	330	660	154	124								
150.01	21	0	0	53	263	37	332	223	705	235	102								
150.10	53	22	0	22	203	75	242	298	693	53	99								

150.20	27	8	0	0	288	24	134	167	726	107	107								
150.31	43	0	0	59	310	83	294	269	782	78	107								
150.41	58	29	27	16	271	33	287	342	775	179	126								
150.51	58	34	10	0	337	50	282	348	736	6	123								
150.61	50	16	0	0	352	89	279	284	715	68	87								
150.71	67	0	0	11	296	139	250	206	792	88	115								
150.80	61	9	9	0	264	110	183	372	801	238	111								
150.91	59	0	6	0	266	30	310	323	804	189	138								
151.01	60	18	7	19	354	53	180	312	806	44	125								
151.11	33	21	7	25	354	55	245	333	755	37	127								
151.21	77	26	11	20	311	18	346	340	752	141	129								
151.30	59	21	17	31	290	25	233	357	793	121	117								
151.41	84	15	14	10	263	27	279	427	732	179	117								
151.50	46	7	0	20	268	6	365	332	735	33	99								
151.60	64	19	12	0	347	0	273	377	709	0	122								
151.70	44	24	24	0	265	43	388	377	764	0	100								
151.80	44	16	0	21	324	30	362	389	675	37	127								
151.90	37	34	7	15	319	46	285	428	733	50	91								
152.00	66	0	31	0	255	88	326	396	696	0	116								
152.11	43	15	0	0	319	49	279	365	621	0	90								
152.21	76	22	12	10	217	39	272	244	684	76	105								
152.31	69	23	12	19	246	21	194	358	718	87	114								
152.41	51	22	16	17	265	83	249	381	709	0	109								
152.51	61	8	18	43	259	95	241	288	697	0	104								
152.61	39	0	5	0	278	75	327	243	680	71	99								
152.70	60	16	0	0	235	0	323	327	634	0	100								
152.81	43	15	0	47	291	64	250	266	687	0	96								
152.91	29	18	10	25	315	79	253	271	720	0	95								
153.01	32	17	0	0	269	74	186	241	769	0	98								
153.11	64	0	0	14	291	99	213	220	818	0	112								
153.21	26	11	0	0	256	65	183	252	746	140	109								
153.30	36	0	7	0	271	50	201	257	648	0	94								
153.40	42	0	0	20	284	95	200	378	760	82	125								
153.50	48	11	0	20	257	23	175	312	683	287	104								
153.61	59	0	0	25	283	65	123	204	667	102	114								
153.71	62	12	0	0	256	109	198	338	751	227	78								
153.81	34	0	26	0	281	71	275	275	728	198	110								
153.90	64	19	16	0	260	73	287	335	788	81	77								
154.01	64	23	0	18	251	30	189	256	782	208	122								
154.11	45	15	0	45	283	54	281	274	744	0	93								
154.21	63	8	17	0	264	113	122	195	749	198	109								
154.31	37	18	0	18	212	70	242	197	756	220	95								
154.41	53	5	0	0	350	97	250	425	769	208	123								
154.50	41	18	0	25	277	74	235	301	810	176	110								
154.60	56	19	13	0	268	82	331	360	837	217	131								
154.71	52	20	12	23	285	82	232	271	815	242	117								
154.81	69	5	0	0	246	77	201	352	821	195	123								
154.91	45	0	8	15	359	53	275	233	849	169	113								
155.01	61	15	21	0	275	108	236	345	907	100	149								
155.11	35	0	0	18	281	140	300	304	884	70	130								
155.20	69	21	9	29	326	43	173	270	842	0	131								
155.31	66	0	0	0	365	89	348	277	873	158	112								
155.41	58	34	0	25	336	25	234	365	938	231	162								
155.51	59	18	0	0	309	38	431	267	992	28	133								
155.61	53	30	25	25	279	84	123	239	973	0	128								
155.70	58	16	13	0	310	47	180	275	1003	12	138								
155.81	50	0	0	12	350	57	337	379	950	0	152								
155.91	79	10	0	4	354	68	247	420	880	50	119								

156.01	74	0	6	0	284	54	202	297	946	178	130								
156.10	46	0	0	30	370	104	230	338	800	0	91								
156.21	61	31	13	8	368	116	280	440	937	87	105								
156.31	61	0	0	0	351	72	468	378	864	8	154								
156.41	59	12	0	19	339	68	205	403	878	0	132								
156.51	58	17	23	27	376	114	312	397	830	139	115								
156.60	34	20	0	10	327	38	321	599	823	0	109								
156.70	53	14	25	0	330	0	272	331	779	17	123								
156.81	45	19	26	12	390	0	250	414	811	64	91								
156.91	54	0	0	16	332	111	279	422	791	0	87								
157.01	80	0	22	0	285	34	328	307	732	0	118								
157.10	46	11	0	12	211	46	208	365	736	0	98								
157.20	65	7	15	24	323	49	383	418	795	0	127								
157.30	56	0	0	0	272	61	196	415	764	0	106								
157.40	70	27	5	34	264	131	371	379	680	0	112								
157.50	68	10	0	0	300	45	267	407	772	124	129								
157.61	43	7	4	4	310	89	238	293	751	172	97								
157.70	38	17	6	4	304	49	236	392	750	0	89								
157.81	51	7	0	11	287	59	215	368	728	0	104								
157.91	76	26	12	17	243	95	330	394	709	0	126								
158.01	74	0	0	0	351	122	333	411	766	0	91								
158.11	54	13	0	6	260	14	352	490	730	0	109								
158.21	36	5	12	0	267	54	196	398	699	0	94								
158.31	71	17	20	0	320	74	240	349	732	53	107								
158.41	74	0	0	24	297	57	248	379	754	77	125								
158.50	56	0	13	0	341	85	209	428	805	38	120								
158.61	75	35	13	7	286	77	281	447	875	91	92								
158.70	39	31	0	0	271	117	236	328	840	68	120								
158.81	65	0	0	90	326	79	265	382	797	135	95								
158.91	83	44	30	42	396	69	209	248	789	137	109								
159.00	47	23	0	0	329	16	176	367	824	195	124								
159.11	51	0	10	9	332	61	248	379	865	27	126								
159.21	72	0	0	0	291	91	222	361	890	39	124								
159.30	51	14	8	0	320	82	220	481	900	142	101								
159.41	54	10	7	0	324	109	323	428	884	112	121								
159.51	52	0	0	24	376	65	319	341	863	0	114								
159.61	57	12	0	0	344	159	354	331	863	277	124								
159.71	48	26	0	0	303	101	258	527	911	75	131								
159.81	46	9	5	17	378	105	266	249	894	0	94								
159.90	52	7	19	33	372	91	312	355	885	0	126								
160.01	68	4	6	18	361	121	279	321	944	104	136								
160.11	36	0	0	35	360	112	277	323	976	99	141								
160.20	48	20	19	0	373	103	266	464	943	73	135								
160.30	59	17	5	0	376	86	302	317	991	0	157								
160.40	61	6	7	44	415	56	302	338	986	255	111								
160.51	92	5	0	33	334	46	344	310	1032	131	133								
160.61	63	29	12	0	358	97	428	309	1014	0	149								
160.71	35	7	0	0	411	61	276	376	1019	45	136								
160.81	37	16	7	0	380	90	286	353	1021	49	105								
160.90	75	0	14	25	362	25	306	396	1012	0	118								
161.00	68	20	23	11	343	119	201	465	989	50	131								
161.11	39	14	0	25	407	89	254	356	959	0	162								
161.21	51	19	0	41	356	59	317	385	967	0	127								
161.31	66	0	0	0	359	80	293	408	1007	163	121								
161.41	65	20	0	14	292	48	261	396	984	71	97								
161.50	55	0	0	22	350	107	156	331	942	0	152								
161.60	61	26	20	26	293	39	293	358	956	0	140								
161.70	71	8	0	0	357	70	303	365	945	40	118								

161.81	35	0	0	0	366	74	211	451	909	0	138
161.91	71	19	8	12	279	122	252	407	886	65	111
162.00	48	8	28	0	294	138	293	252	912	161	134
162.10	51	17	0	0	303	62	374	344	971	0	130
162.21	64	0	13	14	355	117	248	289	917	228	135
162.30	54	4	0	0	383	102	279	320	924	0	111
162.40	60	23	0	13	345	109	208	434	821	0	130
162.50	52	13	28	26	311	32	294	280	888	0	123
162.61	39	15	0	12	350	79	304	366	859	82	98
162.71	48	13	18	44	321	64	226	434	952	26	113
162.81	56	12	11	0	351	99	256	298	907	123	92
162.90	50	5	4	9	310	79	423	283	930	92	116
163.01	48	30	25	0	345	86	264	407	896	46	93
163.11	68	20	12	0	288	122	223	419	980	0	143
163.21	52	22	12	18	398	50	277	322	890	88	120
163.30	53	0	0	49	335	151	243	493	904	49	139
163.40	73	7	16	14	343	124	229	323	912	33	143
163.51	65	0	0	0	333	51	174	345	914	0	155
163.61	54	0	13	0	272	56	274	283	905	130	115
163.71	37	0	0	18	328	43	212	309	883	17	132
163.81	77	19	29	19	336	56	261	306	903	0	106
163.90	41	5	0	0	374	139	277	442	846	120	104
164.01	56	22	0	0	367	59	268	254	928	101	117
164.11	54	0	10	18	376	59	278	409	921	9	119
164.21	59	0	9	49	355	28	278	426	952	0	117
164.31	34	7	0	9	334	50	294	424	953	0	125
164.40	27	25	9	8	346	69	402	430	981	172	109
164.50	56	20	8	49	348	108	248	274	1040	0	135
164.61	44	18	6	5	349	0	256	377	941	58	132
164.71	48	32	0	34	359	115	396	372	1031	0	146
164.80	90	26	8	10	398	114	277	393	999	286	146
164.90	50	0	0	21	319	56	202	319	992	0	168
165.01	34	51	7	17	390	117	236	336	1033	62	127

Scots pine #1. After HCl extraction.

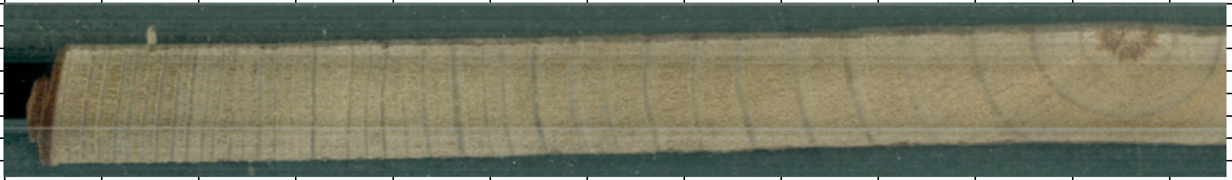

position (mm	Al	P	S	Ti	Mn	Fe	Cu	Zn	Ca	Sr	K
91.91	61	0	0	14	145	76	107	115	68	73	22
92.01	52	8	0	16	62	59	0	18	65	126	0
92.11	50	0	0	0	129	87	87	35	26	121	0
92.21	35	0	0	0	56	41	0	99	31	161	6
92.31	52	6	22	0	95	36	46	69	31	230	5
92.40	48	0	0	0	82	91	49	148	24	221	0
92.51	54	14	0	43	43	0	10	155	76	0	4
92.61	66	4	0	0	77	48	68	140	60	272	0
92.71	39	0	0	17	93	100	153	183	21	191	0

92.80	28	0	9	0	70	67	91	98	39	270	25								
92.91	32	0	0	6	83	61	88	0	37	213	13								
93.01	61	0	0	41	113	94	28	9	48	189	26								
93.10	33	0	0	0	65	56	100	49	57	294	18								
93.21	33	10	0	9	92	56	55	80	73	286	0								
93.30	37	0	0	19	38	99	148	112	46	0	15								
93.41	58	15	0	0	44	35	138	289	32	29	10								
93.51	35	7	8	22	57	76	54	177	74	0	0								
93.60	13	0	0	0	55	78	125	145	41	63	0								
93.70	48	0	0	13	30	89	79	136	35	278	0								
93.81	23	0	0	44	43	46	113	99	64	294	0								
93.90	45	0	0	0	72	59	84	13	45	349	16								
94.01	28	0	0	18	90	73	124	62	46	229	6								
94.11	28	0	0	0	84	65	145	56	62	240	8								
94.20	37	12	0	30	93	28	92	160	17	308	8								
94.31	30	11	0	0	64	57	249	166	48	322	0								
94.41	56	0	0	16	14	75	91	139	40	15	12								
94.50	83	4	0	10	60	61	202	244	35	42	0								
94.61	41	0	0	0	69	57	179	219	39	109	13								
94.71	19	0	0	0	75	21	105	97	56	102	14								
94.80	49	0	0	38	58	42	89	142	32	216	0								
94.91	38	0	0	20	54	105	85	132	61	177	27								
95.01	55	18	0	10	79	87	67	52	56	261	0								
95.10	28	0	0	0	127	75	71	96	31	433	0								
95.21	67	0	0	58	45	50	88	31	52	372	0								
95.30	62	0	0	0	61	34	20	81	18	161	0								
95.40	45	0	0	0	71	41	62	85	47	130	25								
95.50	57	6	0	17	57	52	99	55	38	17	21								
95.60	54	28	0	16	105	68	168	206	48	155	0								
95.71	48	0	0	34	36	66	45	172	52	0	0								
95.80	65	0	0	4	58	32	167	111	61	149	0								
95.91	48	9	0	22	49	69	0	69	22	62	5								
96.01	66	0	8	11	101	22	57	31	34	164	0								
96.11	44	0	0	30	101	83	115	76	47	235	17								
96.21	58	22	7	0	90	80	56	120	34	154	0								
96.30	37	0	0	20	48	28	104	169	38	132	0								
96.41	43	0	10	17	42	105	122	175	56	0	0								
96.51	25	11	0	24	70	65	138	252	38	18	0								
96.60	87	23	4	21	50	44	0	185	56	14	14								
96.71	60	0	0	34	115	78	146	188	20	123	21								
96.81	30	0	0	14	73	56	13	134	50	115	5								
96.91	44	0	0	25	57	103	137	25	37	143	0								
97.01	41	0	0	7	70	76	24	63	25	380	0								
97.10	35	6	4	27	126	72	127	0	39	294	12								
97.21	50	0	0	12	73	135	50	87	22	214	20								
97.31	66	15	0	26	58	55	106	0	70	141	0								
97.40	42	0	16	7	66	0	27	227	64	129	0								
97.51	19	0	0	16	45	45	59	154	34	73	0								
97.61	71	11	0	25	87	77	141	230	48	206	0								
97.71	27	0	0	32	26	67	71	124	53	147	0								
97.81	45	0	0	17	91	78	67	20	29	144	0								
97.90	29	0	0	12	118	119	0	43	34	212	22								
98.01	27	0	0	24	96	39	70	73	36	245	0								
98.11	42	9	0	22	75	76	0	92	65	223	8								
98.21	42	0	0	0	72	60	116	201	42	84	0								
98.31	58	4	0	37	57	12	47	134	28	78	8								
98.41	36	0	0	11	46	28	206	273	34	212	16								
98.51	23	0	0	52	99	45	0	221	74	31	22								

98.61	69	19	0	0	91	38	137	246	46	114	34								
98.71	40	0	0	37	20	32	76	108	26	109	0								
98.80	36	13	0	0	63	79	65	14	58	217	17								
98.91	32	13	0	37	98	88	0	0	40	255	0								
99.01	44	0	0	26	81	33	7	86	59	344	0								
99.10	28	5	0	16	122	68	69	29	22	328	0								
99.21	24	16	0	0	96	46	11	40	44	250	13								
99.30	35	0	0	7	51	40	194	137	22	202	15								
99.41	73	7	0	52	36	53	119	98	45	178	0								
99.51	44	18	0	10	41	0	148	259	74	51	8								
99.61	71	23	5	6	73	33	91	319	20	10	0								
99.71	68	0	14	0	52	70	29	133	6	11	0								
99.81	78	15	0	0	99	77	122	132	29	154	0								
99.91	50	0	0	16	85	55	10	27	54	195	29								
100.00	30	0	0	17	103	51	0	130	57	407	20								
100.11	63	0	0	24	92	98	19	42	39	410	23								
100.20	50	0	0	9	58	41	0	38	37	110	0								
100.31	32	10	0	28	51	69	148	133	59	244	4								
100.40	49	0	0	0	48	61	100	305	47	0	5								
100.51	32	22	11	14	17	60	158	201	42	0	4								
100.61	83	14	17	19	40	14	129	236	31	57	11								
100.70	58	6	10	0	40	50	146	155	37	16	0								
100.81	25	0	0	9	92	92	109	137	49	142	22								
100.90	46	0	0	13	44	79	13	0	46	320	14								
101.01	29	0	0	0	94	82	16	18	32	295	15								
101.11	48	0	0	0	100	39	36	0	39	411	23								
101.20	39	0	4	13	40	7	56	53	23	194	0								
101.31	38	0	0	29	25	24	56	85	18	28	0								
101.40	35	0	0	0	106	13	68	233	31	140	5								
101.50	48	44	0	0	35	22	237	123	53	123	12								
101.61	62	24	13	0	65	17	37	226	42	179	7								
101.71	63	11	0	0	54	74	0	133	43	131	0								
101.80	46	0	0	16	89	43	99	168	24	163	5								
101.90	54	0	0	13	83	64	83	104	40	284	19								
102.01	38	0	0	10	66	67	59	54	28	349	16								
102.10	50	0	0	0	67	40	70	0	18	274	6								
102.20	38	6	0	27	50	61	39	83	72	189	11								
102.31	28	0	0	33	51	92	158	116	26	137	0								
102.41	55	8	0	41	12	40	139	208	27	146	17								
102.50	37	12	0	0	40	63	81	148	23	0	0								
102.61	45	0	0	0	78	46	116	0	35	184	0								
102.70	47	0	0	47	75	89	101	62	61	145	14								
102.81	70	0	0	45	47	188	110	40	29	274	10								
102.91	54	0	0	32	76	150	0	44	47	281	0								
103.01	66	0	0	23	93	105	73	70	40	84	0								
103.11	0	0	0	15	65	94	36	92	22	282	11								
103.21	50	0	0	18	64	86	37	23	36	247	34								
103.31	49	0	0	10	58	0	87	160	22	0	0								
103.41	49	0	0	16	28	66	50	191	42	131	0								
103.50	36	15	0	21	40	17	127	261	33	132	0								
103.60	22	19	0	31	91	72	132	62	35	204	21								
103.71	85	0	0	40	105	55	0	86	32	362	0								
103.80	26	0	0	40	45	67	5	71	58	485	0								
103.91	39	0	0	0	89	109	140	30	44	381	0								
104.01	54	0	0	36	92	36	68	171	47	126	20								
104.11	28	0	0	13	80	91	186	189	34	62	12								
104.20	47	12	0	0	6	64	55	89	27	254	0								
104.31	44	0	0	30	63	56	111	69	24	19	8								

104.41	7	0	0	20	79	94	0	0	43	187	0								
104.51	30	0	0	0	63	92	89	95	20	238	0								
104.61	45	0	0	0	91	81	144	99	45	377	0								
104.71	30	0	0	19	59	55	73	108	41	223	15								
104.80	40	0	0	19	111	66	86	81	58	163	11								
104.91	60	32	0	0	88	66	169	266	41	48	21								
105.01	54	11	0	0	49	35	151	183	32	0	0								
105.11	66	12	0	10	67	15	93	150	48	0	20								
105.21	39	0	4	22	60	12	184	148	45	0	20								
105.31	18	0	0	0	71	72	84	141	45	41	0								
105.41	37	0	0	0	109	34	8	207	44	247	9								
105.51	24	0	0	16	69	83	0	0	29	332	0								
105.61	37	0	0	12	62	62	25	103	26	287	12								
105.71	19	0	0	0	63	39	85	47	51	374	0								
105.81	55	0	0	22	61	90	113	0	33	200	10								
105.91	37	0	0	16	44	70	171	59	51	173	13								
106.01	42	13	13	51	44	31	148	274	64	12	9								
106.10	41	5	6	0	78	15	9	249	36	29	0								
106.21	31	6	0	0	55	45	126	66	29	325	18								
106.31	41	0	0	27	105	90	19	0	63	309	12								
106.40	49	0	0	39	101	74	72	0	54	298	19								
106.51	34	0	0	0	64	114	40	0	46	156	5								
106.61	58	4	10	0	47	125	99	139	39	212	0								
106.71	46	0	0	6	114	0	127	117	50	106	15								
106.81	28	0	0	27	19	30	37	45	41	212	23								
106.91	40	0	0	40	88	50	62	88	46	287	0								
107.01	30	0	0	0	77	66	64	29	45	300	6								
107.11	57	10	0	16	91	81	59	60	72	393	12								
107.21	32	0	0	46	67	45	0	172	46	214	22								
107.31	25	24	0	0	77	0	0	141	44	164	12								
107.41	49	0	0	0	55	45	106	116	25	120	0								
107.50	40	0	0	42	108	52	34	33	18	232	0								
107.61	30	0	0	13	98	45	212	159	47	129	17								
107.71	50	19	0	19	97	28	10	178	9	105	0								
107.80	47	0	0	17	75	73	83	128	18	249	9								
107.91	42	0	0	0	94	44	36	37	37	357	19								
108.01	43	0	0	7	63	101	17	31	54	183	0								
108.10	28	0	0	26	108	115	21	56	42	247	8								
108.21	47	0	0	11	57	62	96	20	44	233	13								
108.30	39	0	0	5	74	94	25	111	56	212	12								
108.41	38	0	0	0	75	47	0	132	45	332	31								
108.51	59	0	0	41	53	56	53	149	46	99	0								
108.60	66	32	11	10	33	0	109	172	32	0	0								
108.71	37	7	0	15	79	0	68	184	66	0	7								
108.80	40	0	0	36	61	37	123	115	43	0	5								
108.91	38	0	0	22	20	70	85	137	58	92	0								
109.01	56	15	0	33	74	67	109	143	28	160	0								
109.11	57	0	0	24	81	64	82	62	30	366	0								
109.20	33	0	0	5	25	52	42	39	33	267	0								
109.31	36	0	0	0	77	57	153	84	42	422	0								
109.41	29	0	0	11	75	72	0	64	52	224	16								
109.51	43	8	0	0	97	78	93	5	57	237	12								
109.60	30	0	0	16	81	90	65	118	74	216	13								
109.71	29	0	0	17	115	77	0	0	41	436	15								
109.81	37	0	0	8	72	57	137	168	67	423	4								
109.91	26	0	0	0	101	28	101	227	64	79	22								
110.01	6	0	0	0	49	18	119	137	34	22	14								
110.10	46	0	0	0	82	68	94	150	35	157	0								

110.21	17	0	0	27	92	33	6	116	52	265	14								
110.31	54	0	0	30	81	101	45	65	50	318	0								
110.41	24	7	0	0	81	57	10	110	47	321	0								
110.51	67	0	0	0	79	0	125	66	63	124	0								
110.60	40	0	0	32	84	62	46	93	52	350	10								
110.71	37	7	0	27	80	55	133	17	36	414	16								
110.81	28	0	0	0	31	102	87	80	62	376	12								
110.91	71	5	10	7	71	45	98	177	102	161	7								
111.00	52	12	4	0	105	42	220	307	97	29	0								
111.10	49	15	4	8	50	36	105	257	54	0	0								
111.21	52	0	0	12	58	85	159	232	49	222	18								
111.30	58	0	0	0	102	11	136	268	44	0	14								
111.41	56	0	0	18	110	64	29	83	69	193	14								
111.50	50	0	0	17	88	66	0	96	23	174	37								
111.61	44	9	0	0	92	111	79	91	64	343	9								
111.71	30	0	0	8	79	93	119	0	72	186	0								
111.81	54	7	0	18	70	65	72	127	32	339	0								
111.91	34	0	0	30	77	75	45	96	38	172	0								
112.01	20	23	0	29	63	100	102	139	61	407	18								
112.11	70	0	0	5	51	4	47	148	37	44	31								
112.21	32	0	11	0	47	52	147	202	67	0	30								
112.31	80	15	0	15	86	55	147	80	64	163	32								
112.41	66	0	0	19	103	118	27	104	48	191	15								
112.51	53	0	0	27	74	107	115	99	64	222	0								
112.61	20	11	0	0	42	98	0	49	64	395	9								
112.70	28	0	0	0	37	112	78	0	61	181	23								
112.81	44	0	0	12	109	89	169	19	40	258	0								
112.91	21	0	0	0	76	146	31	75	60	476	26								
113.01	27	5	0	31	66	60	149	102	57	320	0								
113.11	57	0	0	30	42	70	88	192	72	212	5								
113.20	46	16	7	40	49	10	56	275	48	0	0								
113.30	57	26	0	0	69	18	0	274	49	0	0								
113.41	30	0	0	8	119	38	78	158	54	71	15								
113.50	51	0	0	25	83	73	0	142	32	133	0								
113.61	64	14	0	0	90	0	0	98	24	204	9								
113.71	21	0	0	52	101	96	21	81	41	126	0								
113.81	32	0	0	0	107	132	4	95	59	387	8								
113.90	14	0	0	34	60	111	12	169	50	291	0								
114.01	13	0	0	0	125	108	67	0	40	188	16								
114.11	51	0	0	0	57	80	103	89	31	232	18								
114.20	52	0	0	0	95	105	176	143	50	462	0								
114.31	31	0	0	28	105	20	119	60	60	443	0								
114.40	41	0	0	56	65	92	56	97	29	315	0								
114.51	69	32	5	49	74	15	117	138	76	143	13								
114.60	38	0	0	0	39	56	166	146	77	0	14								
114.70	44	9	0	0	95	36	136	111	50	53	8								
114.80	55	0	0	0	71	0	120	128	47	126	22								
114.91	26	6	0	56	86	86	79	46	30	126	18								
115.00	107	0	0	0	71	63	133	5	49	142	16								
115.11	29	0	0	7	110	74	0	91	32	236	22								
115.21	55	0	0	9	87	80	69	56	37	250	0								
115.31	48	0	0	32	82	98	41	59	51	449	26								
115.40	25	0	0	0	70	103	102	76	49	285	12								
115.50	48	0	8	12	84	79	159	132	51	334	32								
115.61	24	11	0	11	20	84	0	137	43	292	10								
115.71	50	0	0	30	87	46	10	161	62	172	45								
115.81	52	10	0	28	80	45	92	216	64	0	15								
115.91	53	0	0	31	61	61	142	232	51	33	20								

116.01	60	20	0	5	82	91	215	338	50	164	13								
116.11	28	15	0	12	52	15	91	175	77	0	18								
116.21	50	4	17	7	41	26	0	73	39	13	15								
116.31	44	0	0	0	71	0	79	149	43	218	17								
116.40	42	0	0	37	55	32	55	0	53	149	0								
116.51	21	0	0	16	91	79	27	70	62	284	20								
116.60	35	0	0	11	74	111	121	21	47	431	10								
116.71	25	0	0	0	52	66	21	152	63	348	9								
116.81	23	0	0	26	97	76	40	0	38	378	10								
116.91	51	0	13	0	95	58	80	73	26	268	0								
117.01	23	0	0	33	57	92	95	36	53	254	0								
117.10	56	0	0	21	58	45	106	0	46	282	20								
117.20	37	0	0	31	116	48	63	36	48	213	0								
117.31	50	0	0	36	96	17	158	20	36	355	19								
117.41	15	0	0	18	126	75	83	39	61	361	14								
117.51	49	0	0	0	121	75	37	35	44	194	11								
117.61	36	0	4	16	59	93	52	35	52	260	35								
117.71	8	0	0	14	106	85	51	23	51	302	27								
117.81	50	0	0	29	55	28	143	150	15	22	19								
117.90	83	10	18	18	44	13	97	214	66	92	0								
118.00	61	20	0	0	38	41	139	185	66	0	21								
118.11	51	0	0	0	18	43	93	152	54	0	0								
118.21	50	0	10	15	69	41	98	131	56	0	0								
118.31	50	28	0	6	91	46	115	256	7	110	11								
118.40	68	12	16	17	47	22	180	153	45	141	0								
118.51	17	6	0	0	68	44	48	101	36	246	7								
118.61	31	0	0	0	110	83	94	91	56	140	12								
118.70	54	0	0	18	127	98	111	69	64	293	13								
118.80	26	0	0	0	51	62	75	25	53	262	0								
118.91	62	0	0	0	81	65	59	103	71	413	18								
119.01	54	10	0	26	77	102	99	0	42	269	20								
119.10	61	0	0	18	102	60	81	52	44	330	15								
119.21	16	0	0	0	114	94	119	120	67	402	18								
119.31	45	0	0	0	76	79	88	47	22	163	0								
119.41	31	0	0	22	101	102	177	95	53	274	7								
119.51	57	0	0	7	75	32	58	122	56	296	0								
119.61	23	0	0	33	67	41	64	73	54	260	0								
119.71	41	17	0	0	113	25	35	0	58	304	0								
119.80	39	0	0	41	80	74	238	48	43	413	10								
119.91	42	0	8	36	59	41	119	162	76	239	0								
120.00	71	0	7	15	55	11	175	171	88	0	6								
120.11	15	7	0	52	58	50	141	149	52	0	16								
120.20	57	6	0	0	31	36	65	188	64	102	36								
120.31	40	17	0	29	86	80	159	86	51	333	5								
120.40	27	0	0	30	81	49	51	11	72	225	10								
120.51	50	13	0	7	75	57	66	86	33	305	13								
120.61	16	0	0	6	71	123	121	132	50	251	0								
120.70	39	0	0	22	78	91	36	0	50	312	0								
120.81	40	0	0	16	125	9	33	35	74	348	39								
120.91	7	0	0	0	72	44	124	114	78	273	13								
121.01	42	8	0	21	110	110	0	190	76	131	0								
121.11	33	0	0	21	76	16	83	137	96	106	5								
121.21	51	0	0	0	28	76	103	143	71	0	0								
121.31	45	10	23	0	73	29	168	228	72	51	0								
121.41	57	0	0	50	74	75	140	111	72	5	13								
121.50	49	0	0	15	51	77	55	287	56	147	26								
121.60	37	0	0	6	89	94	68	99	48	140	10								
121.70	42	0	12	9	62	50	116	147	58	209	0								

121.80	73	0	0	0	92	74	0	13	64	171	0								
121.90	36	0	0	0	78	37	99	68	78	180	0								
122.01	32	0	0	0	111	112	42	23	50	0	0								
122.10	65	0	0	17	83	77	181	106	59	271	19								
122.21	45	0	0	11	85	66	189	78	51	400	0								
122.31	50	0	0	46	88	15	53	109	64	323	0								
122.40	41	0	0	7	93	54	0	120	74	146	14								
122.50	30	0	0	0	81	81	54	112	63	314	20								
122.61	37	0	0	40	60	87	73	120	32	227	17								
122.71	56	0	0	0	67	51	21	154	36	148	12								
122.81	38	0	0	40	71	79	105	200	74	440	6								
122.91	36	0	0	18	121	109	100	71	43	384	7								
123.01	56	0	0	20	113	59	97	96	63	227	9								
123.10	40	0	0	0	60	19	50	139	68	134	0								
123.21	50	12	18	0	101	76	87	177	67	0	22								
123.30	61	20	0	12	75	10	190	132	73	172	0								
123.41	42	24	11	32	70	0	175	266	46	175	11								
123.51	62	17	0	0	46	25	91	232	57	51	19								
123.60	57	0	0	0	58	0	83	207	58	50	18								
123.71	26	0	6	0	78	34	0	82	41	55	0								
123.81	29	0	0	6	86	50	16	109	60	86	25								
123.90	50	0	0	12	112	85	0	108	62	176	7								
124.01	40	0	0	0	74	60	62	123	56	271	0								
124.10	52	9	0	0	39	29	74	121	67	131	0								
124.21	34	0	0	0	102	122	56	185	49	419	6								
124.30	39	0	0	0	112	16	26	75	32	174	0								
124.41	31	0	0	17	45	65	63	60	71	215	10								
124.51	39	0	0	31	90	97	78	126	55	198	12								
124.61	52	0	0	0	91	93	34	76	57	391	8								
124.71	26	0	0	30	98	75	135	44	75	207	19								
124.80	19	0	0	0	81	51	60	64	44	370	8								
124.91	28	20	0	0	61	104	50	76	38	450	10								
125.01	24	0	0	0	93	45	108	11	71	406	0								
125.11	44	0	0	42	47	52	37	122	54	368	17								
125.21	44	0	0	30	100	20	119	93	61	192	0								
125.31	33	0	0	42	119	72	136	44	34	353	0								
125.40	66	14	5	12	72	51	0	92	53	237	13								
125.50	47	0	0	0	95	93	172	196	47	213	0								
125.61	67	16	22	6	57	23	185	132	46	328	13								
125.71	35	12	18	31	96	0	105	176	47	111	8								
125.81	45	29	0	19	59	6	70	252	69	0	15								
125.91	45	8	0	0	57	0	0	266	60	54	7								
126.01	42	0	0	0	96	77	49	195	37	0	28								
126.11	35	0	0	9	91	21	107	170	61	45	32								
126.21	58	0	0	0	100	20	101	226	68	0	0								
126.31	44	0	0	10	83	20	0	192	51	9	0								
126.41	30	0	0	29	74	41	97	144	60	181	25								
126.51	32	0	13	17	56	57	0	83	62	194	0								
126.60	31	14	0	41	109	30	95	28	34	198	0								
126.70	35	12	0	0	58	67	36	79	84	247	8								
126.81	33	0	0	10	57	90	39	57	42	185	7								
126.90	61	11	18	6	100	78	138	61	40	426	0								
127.01	33	0	0	14	44	75	21	33	42	271	0								
127.10	51	0	0	40	96	57	37	76	60	446	30								
127.21	40	9	0	0	41	84	170	62	49	307	11								
127.31	22	0	0	14	55	83	158	114	68	347	0								
127.41	65	12	0	27	72	59	105	0	39	233	0								
127.50	25	0	0	0	84	58	77	100	48	248	13								

127.61	61	0	0	35	98	85	40	106	50	227	6								
127.71	35	0	0	14	94	58	6	55	24	446	24								
127.80	53	23	0	15	83	37	79	76	36	235	16								
127.91	39	0	0	25	39	37	128	119	39	144	0								
128.01	60	0	0	0	99	54	105	249	46	0	9								
128.10	51	0	4	10	74	59	213	180	48	0	11								
128.21	43	9	0	18	61	28	156	135	52	0	29								
128.30	40	0	0	0	83	38	54	257	54	189	0								
128.41	57	0	0	49	63	51	81	146	71	138	13								
128.51	52	14	12	0	68	16	120	140	43	100	26								
128.61	33	0	0	5	66	70	174	119	79	106	19								
128.71	42	0	0	20	71	74	106	24	57	163	23								
128.81	55	0	0	12	102	42	94	108	33	241	12								
128.91	20	0	0	24	72	71	168	81	40	103	10								
129.01	55	0	0	14	70	44	103	79	54	255	6								
129.11	47	0	0	6	65	55	27	52	46	359	5								
129.20	40	0	0	25	36	79	154	93	47	168	10								
129.31	50	0	0	6	65	112	72	58	36	227	0								
129.41	60	0	0	37	52	121	92	56	49	150	0								
129.51	29	0	0	34	91	93	120	163	68	318	0								
129.61	38	0	0	66	97	58	47	137	66	245	21								
129.71	25	0	0	0	95	83	41	99	51	348	23								
129.81	62	0	0	10	79	111	37	104	46	361	15								
129.90	24	0	0	30	47	86	7	32	78	333	6								
130.00	42	0	0	0	95	233	95	93	74	355	17								
130.10	45	0	0	0	94	101	101	33	53	94	0								
130.20	64	0	0	8	88	83	24	25	52	99	0								
130.30	25	14	0	20	62	28	62	71	79	134	27								
130.41	53	0	0	13	103	77	60	56	91	209	9								
130.51	56	0	8	25	50	76	108	27	52	240	27								
130.60	53	8	0	42	56	43	171	118	74	250	0								
130.71	44	8	23	0	83	60	78	67	83	160	23								
130.81	39	0	0	0	42	0	165	219	93	0	4								
130.90	78	35	10	0	42	30	35	242	52	0	0								
131.01	67	7	0	9	65	0	30	174	46	218	11								
131.11	32	8	10	32	38	16	186	197	34	86	0								
131.21	35	7	0	9	36	0	77	172	49	93	18								
131.31	51	0	0	39	66	69	107	230	53	181	20								
131.40	64	0	0	0	71	37	44	80	32	68	33								
131.50	41	0	0	5	94	63	0	62	76	70	6								
131.60	24	0	0	17	105	66	0	85	48	310	8								
131.70	24	0	0	22	82	116	168	66	72	284	0								
131.81	39	0	0	12	34	40	8	235	57	142	10								
131.91	50	0	0	55	100	77	122	62	41	400	19								
132.00	32	0	0	0	55	84	51	52	56	194	0								
132.11	54	0	0	0	49	65	118	72	80	298	23								
132.20	23	0	0	0	48	89	79	14	68	135	11								
132.31	47	0	0	0	78	77	0	41	51	312	0								
132.41	19	0	0	12	80	81	79	62	32	409	35								
132.51	25	0	0	37	71	69	86	112	48	381	24								
132.61	36	0	0	43	60	84	42	87	64	239	11								
132.70	53	0	0	0	82	87	23	189	71	219	9								
132.81	46	4	0	13	109	134	58	18	69	315	6								
132.91	23	0	7	0	90	115	101	5	48	268	0								
133.01	44	0	0	0	46	109	110	93	62	193	12								
133.10	39	0	0	22	102	89	135	109	70	202	12								
133.21	50	0	0	4	90	66	92	177	33	328	0								
133.31	41	0	0	0	53	88	29	74	65	278	8								

133.41	40	0	0	32	129	87	129	82	54	383	36								
133.51	28	0	0	39	84	53	29	46	52	297	36								
133.60	19	10	0	0	66	92	74	56	44	285	11								
133.71	51	0	0	0	68	23	102	45	55	257	7								
133.81	50	22	0	0	86	68	145	215	61	15	14								
133.91	45	0	0	0	89	41	141	13	49	0	10								
134.01	58	4	18	57	78	58	150	220	67	96	0								
134.11	40	0	0	33	26	42	31	189	33	0	21								
134.21	48	0	0	31	41	77	83	138	69	43	12								
134.30	31	6	0	53	69	89	67	127	65	102	21								
134.41	49	15	0	0	69	40	59	109	47	72	16								
134.51	43	0	0	22	49	74	55	111	49	103	13								
134.61	57	0	0	26	81	35	48	54	61	85	4								
134.71	35	0	0	8	88	54	0	87	75	332	0								
134.81	50	0	0	0	40	96	105	108	55	252	23								
134.90	31	0	0	14	57	83	94	121	50	347	25								
135.00	34	0	9	0	56	50	5	200	69	277	0								
135.10	48	0	0	10	51	62	165	25	53	340	7								
135.20	41	0	0	8	77	79	71	165	74	212	13								
135.31	48	0	0	0	103	162	0	121	60	541	4								
135.41	35	0	0	0	44	116	92	175	72	384	0								
135.51	29	8	0	13	83	67	73	107	66	244	0								
135.61	38	0	0	21	103	55	78	109	42	97	38								
135.71	44	0	0	4	61	40	58	26	85	249	0								
135.81	32	0	0	0	33	20	28	0	87	307	26								
135.90	46	0	0	19	48	108	57	100	62	279	31								
136.01	41	0	0	31	74	86	107	62	55	389	0								
136.11	56	8	0	34	71	68	77	17	73	151	22								
136.21	43	0	0	8	66	85	83	38	69	268	16								
136.30	32	0	0	0	106	96	107	126	86	275	8								
136.41	20	0	0	37	88	40	114	50	74	330	0								
136.51	43	0	0	9	73	44	168	33	51	218	14								
136.61	34	0	0	15	88	82	189	67	56	239	6								
136.71	32	0	0	15	79	93	116	172	34	310	0								
136.80	35	17	0	22	61	73	81	144	76	74	10								
136.91	30	6	8	0	131	124	197	241	60	80	5								
137.01	25	9	0	25	65	0	97	126	53	0	18								
137.11	69	15	0	41	48	19	71	75	54	0	20								
137.21	47	30	12	9	75	0	23	109	72	0	9								
137.31	36	0	0	0	13	53	48	187	40	187	9								
137.40	49	0	0	0	79	25	5	195	69	0	11								
137.51	48	7	0	0	46	44	47	122	6	0	30								
137.61	40	0	0	20	20	43	119	108	64	27	23								
137.71	61	0	4	0	44	64	0	47	21	6	16								
137.81	34	5	0	26	70	62	101	62	41	134	5								
137.90	45	0	5	44	65	55	68	57	55	198	9								
138.00	47	0	0	18	85	36	74	43	78	99	17								
138.11	50	0	0	32	73	113	144	104	22	249	32								
138.21	37	21	0	28	64	77	63	18	74	288	19								
138.30	33	0	0	0	66	70	83	130	58	153	7								
138.41	16	0	0	20	119	75	20	138	60	201	11								
138.51	38	15	0	19	67	70	46	37	63	159	23								
138.61	36	0	0	39	58	63	120	153	41	187	0								
138.71	35	0	0	17	58	79	8	109	48	209	17								
138.80	15	5	0	9	67	57	160	138	63	145	9								
138.90	54	0	8	0	57	111	12	86	49	156	17								
139.01	39	0	0	28	57	87	46	58	56	78	0								
139.11	71	0	0	33	38	78	101	182	62	213	5								

139.21	39	0	0	14	64	103	23	87	49	229	0								
139.31	30	0	0	24	26	90	131	73	45	322	6								
139.41	22	0	10	0	65	69	189	117	69	198	25								
139.51	34	0	0	17	68	29	70	74	52	130	0								
139.61	31	0	5	0	81	36	122	83	34	227	0								
139.70	34	0	0	32	56	111	35	137	61	298	0								
139.81	44	26	0	20	64	32	78	16	50	279	13								
139.91	36	0	0	5	71	65	122	69	60	294	6								
140.01	68	0	0	28	101	43	208	25	79	344	0								
140.11	43	0	0	28	59	87	143	44	48	217	4								
140.20	35	0	0	20	70	63	97	40	77	307	18								
140.31	21	0	0	31	58	70	128	105	67	394	0								
140.41	65	0	0	22	120	103	136	115	50	72	10								
140.50	44	0	0	12	44	76	38	194	74	253	15								
140.61	44	12	0	0	81	54	129	145	49	365	0								
140.71	59	0	13	18	70	85	69	51	48	123	16								
140.81	70	0	0	0	32	61	169	143	61	201	11								
140.91	45	0	0	8	98	28	163	153	77	263	27								
141.01	72	13	16	0	59	70	105	85	74	189	16								
141.11	67	22	0	33	73	76	196	113	50	0	15								
141.20	50	0	0	6	96	74	175	130	55	0	5								
141.31	75	7	0	0	53	64	157	160	55	118	15								
141.41	37	0	0	9	89	7	87	231	61	84	7								
141.50	54	0	0	0	86	35	124	82	61	69	4								
141.61	63	11	0	23	56	80	214	43	54	253	34								
141.71	54	5	0	0	110	36	34	175	49	69	0								
141.81	39	0	0	0	62	0	0	0	60	163	11								
141.90	48	5	0	0	67	70	79	42	38	172	0								
142.01	64	0	0	38	87	118	53	78	43	166	15								
142.10	90	0	0	4	62	118	0	25	61	15	0								
142.21	54	7	0	19	29	41	10	106	90	131	8								
142.31	20	13	0	0	78	102	105	72	57	216	18								
142.40	44	0	0	0	72	74	40	203	51	183	4								
142.51	52	6	0	32	78	119	178	165	52	101	15								
142.60	36	0	0	33	79	127	52	99	63	170	14								
142.71	41	0	0	28	68	57	57	43	54	186	31								
142.80	56	0	0	19	123	86	119	133	51	138	0								
142.90	60	0	7	27	77	77	0	242	69	153	18								
143.01	30	0	0	0	95	119	0	144	65	279	0								
143.10	46	0	0	14	30	94	158	102	49	301	0								
143.21	37	10	0	0	85	114	28	47	80	270	18								
143.31	37	0	0	8	61	99	30	44	79	275	0								
143.41	41	0	0	0	81	83	151	166	51	185	7								
143.51	42	0	0	0	73	112	94	38	47	166	30								
143.61	50	0	0	16	78	66	76	0	63	290	16								
143.71	30	0	0	0	75	95	124	117	77	260	6								
143.81	55	0	0	23	91	51	112	161	55	342	0								
143.91	11	0	0	29	61	54	108	89	43	260	18								
144.01	34	6	0	0	57	95	28	76	65	260	9								
144.10	41	6	8	0	96	94	43	104	50	157	6								
144.21	56	0	0	0	95	95	32	183	114	201	11								
144.30	74	0	9	7	86	50	79	80	70	0	21								
144.41	47	10	0	9	72	40	73	120	68	238	9								
144.50	54	17	27	0	69	76	109	141	80	217	0								
144.61	36	0	15	0	70	38	160	104	73	107	40								
144.71	56	0	0	0	124	60	187	129	89	126	0								
144.81	70	8	0	27	66	68	176	102	83	9	13								
144.91	42	15	8	12	18	56	91	166	57	71	9								

145.00	57	19	0	0	63	87	43	227	89	135	48								
145.11	41	0	0	31	30	102	96	240	75	221	7								
145.21	35	9	0	23	0	39	68	76	51	188	14								
145.31	48	0	33	15	81	71	68	138	65	0	24								
145.41	58	0	0	8	77	78	138	52	77	155	0								
145.51	72	0	0	24	25	45	93	89	56	0	50								
145.61	55	19	0	8	112	77	140	120	96	125	27								
145.71	41	0	0	22	67	76	61	77	115	113	18								
145.81	55	12	0	24	65	40	101	79	83	225	0								
145.91	32	25	11	13	82	39	179	102	58	46	0								
146.00	52	8	0	8	90	43	62	165	86	89	11								
146.10	34	0	0	26	51	59	147	69	70	254	0								
146.20	47	0	0	7	108	76	195	42	82	148	10								
146.31	58	0	7	0	85	82	96	55	83	154	17								
146.41	57	0	0	25	86	61	89	166	79	179	0								
146.50	22	0	0	0	100	50	68	139	65	237	20								
146.61	58	11	0	21	136	121	267	92	67	80	8								
146.70	34	0	0	21	84	112	50	174	94	112	18								
146.81	34	0	0	35	27	126	70	29	71	129	16								
146.91	36	7	0	28	21	41	78	59	77	263	21								
147.01	65	5	18	14	103	85	14	0	92	328	4								
147.11	63	0	0	18	64	65	124	86	80	272	0								
147.20	38	26	0	31	78	96	90	30	78	144	0								
147.31	19	0	0	0	122	102	207	54	68	223	13								
147.40	52	0	0	15	74	85	162	87	76	256	18								
147.51	34	0	0	19	71	49	52	72	72	195	13								
147.61	46	0	0	30	82	41	148	129	86	353	0								
147.71	55	11	0	30	84	82	0	16	61	115	33								
147.81	36	7	0	13	51	64	59	71	56	153	0								
147.91	58	0	0	87	73	68	95	140	58	306	0								
148.01	41	0	4	0	74	86	81	25	61	180	20								
148.11	34	0	0	0	57	114	161	120	77	197	18								
148.21	58	4	0	0	79	86	25	89	72	205	0								
148.31	61	17	5	19	62	50	126	14	97	0	0								
148.41	59	17	0	0	89	53	134	219	70	100	7								
148.51	42	16	0	24	58	32	109	118	76	132	0								
148.60	76	13	0	12	47	60	42	209	66	130	18								
148.70	59	0	0	34	43	47	104	167	88	74	0								
148.80	48	14	0	15	48	78	129	59	58	32	8								
148.91	46	0	0	11	84	25	154	248	68	167	0								
149.00	65	13	8	0	54	64	142	218	86	0	0								
149.11	37	11	0	7	65	0	132	164	74	16	24								
149.21	44	19	8	0	58	55	47	115	71	0	17								
149.30	42	0	0	4	15	28	49	66	67	29	0								
149.40	66	7	0	0	87	42	61	180	58	0	20								
149.51	74	17	0	36	63	44	61	180	70	57	10								
149.60	75	7	0	24	31	18	101	45	73	310	0								
149.70	57	5	0	0	76	68	27	85	45	40	29								
149.81	42	0	0	17	0	50	40	64	96	258	0								
149.90	31	0	0	12	69	86	89	267	78	123	17								
150.00	59	0	0	30	128	76	0	131	74	212	10								
150.11	45	0	0	0	86	66	102	143	108	180	0								
150.21	49	9	0	27	94	77	77	85	90	41	12								
150.31	60	0	0	5	114	81	130	130	76	316	0								
150.40	29	0	0	34	71	65	0	94	71	101	15								
150.51	48	0	0	5	92	88	146	137	71	228	0								
150.60	41	0	0	0	64	68	62	127	87	424	26								
150.70	25	0	0	0	42	35	126	127	82	123	0								

150.81	61	7	15	5	64	104	171	45	81	122	9								
150.90	36	8	0	10	62	77	12	153	68	212	12								
151.01	50	25	0	0	73	48	37	73	85	352	15								
151.11	10	0	0	0	56	82	15	26	55	272	37								
151.21	42	0	0	27	75	111	0	108	75	230	40								
151.31	49	0	0	23	109	58	69	10	75	66	0								
151.41	27	0	0	35	118	139	131	219	102	114	12								
151.51	58	5	0	9	62	58	43	130	63	281	11								
151.61	37	0	0	17	64	54	156	116	57	269	19								
151.71	26	0	0	6	127	76	24	108	100	226	13								
151.81	40	0	0	10	75	74	54	45	59	194	10								
151.90	62	0	0	40	70	51	119	131	77	341	35								
152.01	48	0	0	6	30	82	93	25	90	357	14								
152.11	22	0	0	17	87	70	164	114	86	346	0								
152.21	40	0	0	33	60	150	114	69	103	122	15								
152.30	70	0	0	11	55	113	161	120	74	204	6								
152.40	32	0	0	0	51	58	107	79	98	114	15								
152.51	62	0	0	0	21	32	102	13	117	279	0								
152.61	55	0	0	31	56	84	147	57	94	271	0								
152.70	51	0	0	29	62	27	78	8	102	171	0								
152.81	53	10	0	4	63	71	19	207	110	292	14								
152.91	29	0	0	0	67	81	103	77	80	161	4								
153.00	44	19	0	30	37	57	59	147	84	198	13								
153.11	46	0	0	14	88	109	160	86	38	133	0								
153.20	29	0	0	14	63	37	152	100	92	57	32								
153.31	37	0	0	0	66	85	97	156	73	388	19								
153.41	44	20	0	58	51	39	0	130	104	118	22								
153.51	44	0	7	20	71	117	155	244	90	109	37								
153.61	61	16	0	8	78	107	50	94	79	15	0								
153.71	66	0	9	45	97	54	52	106	86	273	30								
153.81	54	0	0	40	41	9	9	103	101	0	30								
153.90	86	20	0	29	14	17	26	67	103	163	6								
154.00	64	0	0	0	89	38	96	220	88	18	14								
154.11	49	0	0	10	67	61	72	173	101	120	31								
154.21	43	0	0	0	74	63	79	171	65	176	0								
154.31	53	0	0	24	48	77	158	183	54	282	9								
154.41	52	14	0	35	66	108	134	127	101	135	0								
154.51	57	0	12	25	87	47	84	133	88	0	22								
154.61	33	12	0	16	22	71	133	144	73	62	0								
154.70	39	0	0	30	71	59	36	57	92	211	36								
154.81	39	0	0	10	52	87	196	120	89	162	0								
154.90	55	0	0	0	81	54	161	153	54	195	15								
155.01	61	0	0	4	112	72	90	247	67	46	0								
155.11	59	0	0	0	39	80	199	182	61	60	0								
155.20	73	0	0	7	82	112	108	207	88	7	11								
155.31	39	13	0	53	52	82	92	232	105	253	6								
155.40	39	9	0	27	88	81	129	230	98	0	16								
155.51	52	0	0	23	81	0	58	109	75	65	18								
155.61	41	11	0	24	73	71	187	145	88	256	0								
155.71	49	0	0	0	68	75	56	192	89	260	0								
155.80	46	0	0	0	35	142	223	71	78	211	18								
155.91	35	12	6	0	119	46	154	178	108	109	11								
156.01	40	25	0	0	77	111	57	127	104	92	0								
156.10	65	5	9	9	69	68	161	87	75	128	13								
156.21	46	0	0	0	79	94	23	106	83	244	35								
156.31	28	29	0	25	113	116	91	117	88	128	26								
156.40	43	0	0	15	109	56	155	166	87	97	0								
156.51	40	0	0	42	80	77	48	169	73	184	0								

156.61	33	0	0	7	45	71	137	156	84	123	0								
156.70	36	0	0	0	66	75	69	119	77	151	21								
156.81	45	0	0	0	107	126	160	130	83	83	14								
156.91	80	0	0	7	74	112	123	43	78	396	8								
157.01	28	7	0	19	74	97	93	91	85	63	40								
157.11	63	0	0	0	122	97	153	98	37	42	31								
157.21	50	10	0	27	106	95	44	160	92	270	23								
157.31	51	0	0	10	56	87	98	4	80	157	5								
157.40	57	0	0	26	34	105	68	232	92	225	8								
157.50	40	0	7	0	68	87	26	172	88	62	18								
157.61	63	0	0	14	55	141	38	96	102	206	0								
157.70	47	12	0	0	42	43	51	97	90	105	0								
157.80	42	0	9	39	49	63	91	84	91	197	21								
157.90	55	7	0	0	104	86	170	136	83	201	19								
158.01	50	9	7	0	75	110	104	72	93	160	0								
158.11	39	15	0	0	59	118	87	149	114	239	28								
158.21	43	8	0	49	72	105	94	271	110	125	8								
158.31	50	7	0	36	85	135	73	148	76	191	0								
158.41	52	11	0	20	20	149	170	130	110	282	11								
158.50	53	19	8	18	67	85	173	86	76	280	0								
158.61	44	11	0	26	44	121	119	80	81	124	10								
158.71	25	0	0	0	44	121	189	137	109	187	36								
158.81	40	0	9	39	104	68	172	149	103	201	0								
158.91	20	0	0	38	54	102	158	168	72	0	25								
159.00	54	19	0	16	66	86	89	109	72	0	26								
159.11	39	11	0	22	58	62	45	106	87	86	21								
159.20	46	0	0	29	22	47	40	218	78	306	7								
159.30	77	35	13	34	84	78	37	134	84	0	0								
159.41	46	0	0	0	67	94	117	175	107	194	27								
159.51	68	13	0	45	49	91	53	87	79	165	28								
159.61	54	0	0	0	54	97	50	138	82	245	0								
159.71	25	0	0	29	77	115	82	121	111	48	7								
159.81	32	19	0	12	64	89	101	0	81	276	17								
159.91	38	0	0	26	51	130	113	147	94	162	6								
160.00	54	0	0	21	53	79	33	155	75	0	0								
160.11	27	0	0	0	67	82	58	156	84	200	17								
160.20	48	19	0	45	62	85	216	209	55	157	22								
160.31	31	7	0	0	75	104	62	204	106	198	32								
160.40	34	8	0	0	50	103	0	154	66	122	6								
160.51	27	0	0	0	101	80	105	169	82	159	0								
160.61	10	0	0	25	45	44	140	182	84	0	20								
160.71	15	0	10	17	88	79	218	100	99	29	26								
160.80	54	5	0	17	24	113	217	56	121	80	0								
160.91	35	8	0	0	26	159	71	67	92	85	0								
161.00	48	0	0	24	71	103	103	186	75	174	0								
161.11	47	0	0	16	92	73	93	149	87	94	27								
161.21	58	6	0	0	80	42	19	82	101	198	0								
161.30	37	10	0	7	47	132	53	121	83	158	23								
161.41	38	0	6	12	87	130	173	127	101	86	10								
161.50	35	20	0	11	98	86	65	145	98	109	0								
161.61	42	13	0	9	38	102	170	189	98	83	39								
161.71	26	0	0	0	69	92	16	158	105	244	22								
161.81	59	10	0	35	132	72	113	238	136	31	0								
161.91	37	7	0	0	90	99	157	124	122	208	16								
162.01	67	0	0	17	43	118	61	181	130	270	35								
162.10	41	35	0	0	63	139	125	169	97	131	9								
162.21	62	0	0	26	100	111	39	163	99	56	12								
162.30	35	21	0	28	97	80	44	103	111	71	0								

162.40	55	0	0	6	58	99	89	139	107	142	19								
162.51	32	0	0	0	70	128	182	36	102	199	24								
162.60	65	22	0	11	99	103	106	138	108	184	0								
162.71	41	0	0	0	33	146	199	183	108	59	8								
162.80	57	0	0	11	45	113	166	188	127	119	7								
162.91	46	10	10	17	69	71	66	65	102	161	0								
163.01	34	0	0	39	66	67	71	100	133	170	0								
163.10	61	5	0	0	44	98	182	168	115	165	0								
163.21	29	0	5	28	79	147	76	97	94	57	0								
163.30	51	0	0	25	72	128	89	140	123	186	10								
163.41	61	0	0	0	93	143	166	155	96	211	0								
163.51	61	0	13	18	115	131	151	128	118	0	0								
163.61	78	0	0	31	81	135	0	127	84	141	22								
163.71	13	5	0	0	44	145	76	176	98	56	13								
163.81	41	0	0	13	65	145	183	147	103	124	25								
163.91	39	15	0	26	32	104	49	211	107	181	0								
164.01	62	0	0	35	97	105	139	117	103	288	14								
164.11	46	0	0	0	80	110	82	69	118	167	20								
164.21	36	0	0	10	98	124	31	109	86	387	0								
164.31	59	32	0	28	49	149	53	102	128	149	0								
164.41	61	0	0	47	54	90	95	49	92	165	11								
164.51	39	5	4	26	61	77	80	14	139	263	17								
164.61	41	0	0	8	77	135	84	130	111	247	18								
164.70	64	16	0	14	93	110	93	30	65	159	9								
164.81	62	37	5	19	41	121	34	65	99	312	0								
164.91	58	0	0	0	81	80	24	95	104	290	6								
165.00	71	0	8	17	51	141	39	162	78	8	28								
165.11	68	5	0	45	86	127	111	110	154	187	0								
165.21	51	0	12	26	58	100	148	131	94	107	17								
165.31	61	15	0	4	97	114	84	47	105	69	11								
165.41	31	6	0	28	59	109	52	101	103	275	32								
165.50	61	0	0	0	35	31	98	260	101	286	8								
165.61	43	15	0	0	56	111	145	151	61	179	0								
165.70	49	0	0	0	65	115	74	89	98	135	25								
165.80	54	10	0	0	27	111	79	149	106	73	0								
165.91	34	0	0	14	81	81	18	147	89	283	5								
166.00	53	19	5	0	28	72	103	110	67	75	25								
166.10	48	13	0	59	29	34	81	153	111	173	35								
166.21	36	0	0	45	76	155	55	102	117	84	0								
166.30	54	16	0	6	61	27	37	95	76	77	14								
166.40	46	37	25	0	101	121	143	154	87	230	12								
166.51	63	9	0	0	41	16	179	115	107	143	9								
166.61	51	0	0	0	88	95	121	131	106	263	13								
166.71	46	0	0	0	59	77	71	166	83	161	19								
166.81	79	0	0	0	84	117	146	155	91	130	20								
166.91	41	0	0	0	73	147	203	238	92	219	31								
167.01	37	33	9	0	68	69	164	115	113	174	11								
167.10	47	13	0	17	57	154	53	83	93	166	10								
167.21	69	4	7	14	91	58	79	126	124	348	7								
167.31	38	0	0	0	100	99	148	89	82	97	35								
167.41	36	0	0	13	72	37	177	129	73	28	18								
167.51	70	5	0	49	66	124	63	161	117	131	8								
167.60	58	0	0	0	72	79	63	114	96	272	31								
167.71	40	5	0	44	82	99	59	140	124	97	12								
167.81	55	0	0	9	79	107	45	218	100	260	13								
167.91	18	0	0	21	87	92	173	156	101	164	0								
168.01	27	0	0	12	47	77	176	219	113	59	14								
168.10	43	9	0	7	36	81	69	245	105	107	6								

168.21	49	12	0	16	52	105	162	66	105	159	10								
168.31	49	7	0	0	50	122	103	201	107	76	24								
168.41	35	13	0	0	116	125	22	193	120	168	8								
168.50	49	20	23	8	79	91	103	97	97	165	0								
168.61	49	0	0	0	46	50	144	100	154	139	0								
168.70	76	9	0	0	114	51	9	96	101	0	6								
168.81	46	0	0	0	68	111	43	120	116	213	4								
168.91	46	0	0	32	75	99	32	116	98	150	10								
169.00	57	10	0	0	77	119	41	0	114	122	4								
169.11	43	0	0	26	54	84	78	95	93	152	0								
169.21	41	6	0	16	92	125	114	108	96	74	5								
169.30	38	0	0	11	108	112	32	99	158	115	0								
169.41	17	0	0	0	76	62	0	138	84	528	19								
169.51	45	0	0	0	95	85	72	131	116	141	25								
169.61	46	0	0	0	97	124	62	75	127	19	39								
169.71	77	0	0	0	89	112	114	101	101	185	23								
169.81	48	8	0	17	115	107	160	84	76	100	14								
169.91	46	0	0	0	54	106	81	138	122	34	7								
170.01	40	0	0	24	97	183	77	243	121	134	37								
170.11	57	0	0	19	68	108	237	298	125	440	0								
170.21	62	0	0	35	65	81	131	62	113	231	9								
170.30	53	30	23	0	34	110	214	133	123	0	10								
170.41	66	12	0	33	61	76	83	135	126	109	8								
170.50	44	26	6	9	74	140	4	186	126	0	6								
170.61	69	0	0	0	71	101	125	176	139	295	15								
170.71	44	12	9	0	43	0	69	43	112	100	4								
170.80	22	0	0	0	69	79	152	123	116	67	22								
170.90	43	0	0	62	87	78	175	122	121	42	15								
171.00	37	22	0	7	0	109	183	105	101	123	0								
171.11	70	0	15	0	80	61	144	200	127	52	0								
171.21	22	0	0	14	29	82	102	149	94	154	0								
171.31	56	0	0	7	51	82	199	272	139	179	5								
171.41	26	5	26	0	0	96	109	136	105	88	0								
171.50	44	0	0	0	14	5	55	174	99	48	20								
171.60	48	5	0	28	67	113	134	183	127	20	28								
171.71	65	0	0	6	51	107	122	191	145	60	7								
171.80	72	12	0	21	68	62	52	154	103	40	0								
171.91	34	0	0	33	28	108	127	240	132	187	0								
172.00	57	0	0	10	64	173	36	208	155	173	16								
172.10	43	8	0	12	70	142	130	162	125	243	27								
172.20	42	0	0	0	54	91	167	169	127	119	0								
172.31	65	25	0	16	83	21	77	228	91	158	10								
172.41	67	16	0	31	51	69	249	191	142	242	24								
172.51	53	11	21	0	70	109	24	164	120	24	0								
172.60	71	0	0	31	37	93	92	196	143	363	14								
172.71	62	0	11	0	92	70	80	182	109	379	0								
172.80	31	9	0	6	39	10	115	189	98	222	18								
172.91	44	10	0	0	120	106	15	107	141	87	12								
173.01	62	0	0	33	61	60	0	124	159	263	0								
173.10	52	0	0	58	68	74	174	0	112	184	12								
173.21	29	7	0	12	112	69	83	243	125	170	0								
173.30	61	0	0	36	67	39	111	257	126	362	0								
173.40	27	0	0	40	58	137	0	160	128	282	0								
173.51	55	0	0	18	33	122	110	96	167	343	21								
173.60	31	12	0	36	93	128	76	113	109	99	5								
173.71	29	0	0	28	30	108	59	106	140	159	0								
173.81	31	0	0	22	48	116	148	161	130	47	0								
173.90	64	0	0	0	105	105	131	146	118	210	10								

174.01	23	0	0	18	24	104	78	82	133	345	0								
174.11	43	4	0	19	77	69	79	58	150	157	13								
174.21	58	6	0	0	50	116	78	103	134	307	17								
174.31	33	0	0	22	16	20	34	96	143	226	16								
174.40	72	0	0	24	64	84	159	56	132	346	17								
174.50	66	15	0	49	63	54	146	187	89	310	11								
174.61	27	0	0	0	45	88	125	190	104	0	16								
174.71	51	7	0	24	48	71	0	183	91	171	0								
174.81	67	7	0	0	37	86	122	124	118	107	13								
174.91	46	14	0	17	47	84	39	155	139	198	5								
175.01	61	9	11	0	36	93	143	72	133	57	0								
175.11	52	0	0	0	44	12	125	108	139	246	6								
175.21	54	0	0	38	86	89	98	62	103	114	38								
175.31	44	14	0	0	44	72	149	149	142	0	17								
175.41	30	4	14	20	77	94	105	224	112	203	33								
175.50	39	10	8	68	86	79	148	106	137	122	42								
175.61	57	7	0	30	61	107	76	258	128	155	0								
175.71	43	12	13	27	28	74	47	229	130	44	16								
175.81	39	12	9	0	98	109	191	129	124	0	12								
175.91	39	0	6	11	101	28	186	202	107	96	0								
176.00	71	0	0	0	38	36	114	156	139	0	11								
176.11	43	0	8	0	94	87	131	207	176	201	26								
176.21	39	0	0	16	93	12	95	132	126	89	36								
176.30	51	33	0	0	31	79	0	170	102	198	24								
176.40	51	0	0	0	89	55	129	217	124	82	31								
176.50	46	7	14	33	71	57	122	182	107	107	15								
176.60	57	0	0	6	37	27	99	156	127	0	5								
176.71	63	12	0	50	60	109	92	135	91	0	0								
176.81	82	17	16	22	139	76	96	224	126	253	0								
176.91	36	0	0	0	63	48	91	182	118	0	10								
177.01	34	0	0	13	124	90	99	171	97	109	15								
177.11	54	22	5	38	57	29	44	235	111	124	21								
177.21	48	10	0	32	56	20	70	61	110	83	18								
177.30	32	11	0	16	50	100	39	136	97	258	15								
177.41	44	7	0	20	61	141	139	137	127	125	25								
177.51	60	5	0	17	64	37	0	41	92	233	15								
177.61	52	0	0	9	97	120	108	32	105	121	29								
177.71	58	0	0	31	60	102	0	136	127	306	5								
177.80	24	0	4	40	42	58	156	167	140	173	0								
177.91	50	0	0	40	98	133	31	249	127	260	17								
178.01	77	8	0	0	67	51	77	150	146	141	11								
178.10	77	0	0	7	118	107	66	129	123	197	7								
178.21	68	0	0	35	99	64	94	173	109	156	23								
178.30	41	0	0	0	87	139	90	128	108	369	9								
178.41	88	10	0	0	69	57	129	154	166	122	19								
178.51	51	7	10	21	77	79	64	135	127	260	27								
178.61	50	17	0	6	77	97	112	124	125	311	24								
178.71	83	18	18	0	74	85	77	222	146	0	45								
178.81	58	17	0	0	60	81	105	180	108	203	0								
178.90	46	0	7	38	73	60	101	138	132	219	0								
179.01	76	0	0	16	42	87	58	147	117	42	17								
179.11	74	0	0	44	66	27	115	172	130	156	8								
179.21	51	11	7	16	64	33	96	126	139	187	17								
179.31	57	0	0	21	74	81	99	344	107	0	15								
179.40	47	5	5	26	46	73	139	143	183	0	12								
179.50	55	0	0	5	39	47	72	373	182	0	5								
179.61	44	14	11	25	82	73	212	157	117	79	14								
179.71	47	0	0	65	61	39	132	180	105	140	0								

179.81	45	10	0	34	95	16	248	225	114	104	0								
179.90	46	19	4	0	65	53	152	208	110	0	16								
180.01	45	18	0	0	69	68	135	228	120	0	23								
180.11	45	5	0	15	51	66	83	206	103	0	16								
180.20	39	8	18	0	96	98	48	208	105	8	0								
180.31	36	20	6	0	91	63	91	36	138	247	0								
180.40	55	10	0	0	130	60	32	89	139	304	5								
180.51	44	10	0	36	60	69	54	88	92	133	0								
180.61	25	5	0	25	71	33	81	81	125	158	19								
180.70	40	0	0	0	88	120	122	131	124	335	9								
180.81	52	0	0	0	63	60	121	114	159	200	0								
180.91	49	6	0	0	48	77	14	0	119	334	25								
181.00	67	6	0	20	85	112	39	133	103	341	21								
181.11	35	13	0	0	29	124	170	213	111	230	6								
181.20	13	6	0	23	78	122	54	57	109	50	18								
181.30	56	0	0	12	46	41	170	142	118	68	23								
181.41	55	0	0	0	84	77	150	98	133	196	19								
181.51	45	0	0	34	86	107	75	107	90	223	13								
181.60	58	0	0	19	114	84	69	248	142	209	0								
181.71	14	0	0	0	34	76	109	35	128	247	16								
181.81	21	11	0	13	72	72	192	96	109	110	10								
181.90	31	5	0	9	110	75	181	138	144	209	0								
182.01	47	28	0	19	80	66	77	201	117	125	0								
182.11	58	0	9	17	86	61	89	138	145	255	0								
182.21	56	14	0	33	74	82	52	161	135	140	0								
182.31	43	0	0	0	42	83	65	144	111	82	8								
182.40	45	14	0	24	55	56	103	183	109	119	5								
182.51	63	13	0	0	93	53	157	125	134	32	0								
182.61	66	6	0	46	59	156	139	39	135	227	12								
182.71	97	14	0	0	99	113	59	163	125	0	14								
182.81	26	7	0	18	108	136	196	241	133	0	34								
182.90	67	21	0	51	31	76	166	220	128	0	13								
183.01	47	0	0	11	80	100	78	238	119	0	34								
183.11	79	28	8	23	61	47	139	239	123	20	21								
183.20	64	32	7	0	48	42	177	209	140	0	5								
183.31	78	0	0	28	79	33	127	176	134	0	7								
183.41	54	0	0	0	70	68	161	117	102	118	23								
183.51	58	12	0	34	23	57	149	138	90	51	19								
183.61	49	11	0	14	93	95	19	84	141	0	26								
183.70	22	0	0	24	70	186	0	142	140	146	17								
183.81	41	11	0	0	90	45	61	64	116	480	45								
183.91	78	19	0	19	95	156	70	102	116	421	0								
184.01	25	0	0	4	56	98	124	0	122	296	8								
184.11	48	0	0	20	72	81	43	21	107	210	0								
184.20	32	0	0	47	81	133	78	24	122	466	0								
184.30	34	0	0	23	92	108	119	96	113	285	0								
184.41	31	4	0	17	74	108	0	52	122	184	15								
184.51	23	0	0	37	83	59	0	105	160	180	24								
184.61	31	0	0	39	35	77	94	20	140	220	9								
184.70	45	0	0	31	35	64	89	65	103	51	10								
184.81	48	0	0	18	47	70	63	73	107	13	0								
184.90	63	0	0	5	38	81	107	95	150	136	9								
185.01	34	8	0	9	50	61	0	72	127	80	20								
185.11	52	6	5	0	53	64	106	96	112	296	0								
185.20	58	0	0	16	70	83	115	77	156	331	11								
185.31	63	0	0	0	74	32	49	93	151	234	17								
185.40	38	0	0	0	83	56	41	113	158	312	0								
185.50	38	0	0	15	56	65	62	184	137	249	0								

185.61	61	13	9	45	48	83	75	113	144	233	0								
185.71	69	10	0	0	39	70	107	205	149	280	0								
185.81	39	0	0	27	39	72	150	142	126	95	13								
185.91	30	7	0	0	30	122	57	106	132	96	17								
186.01	47	9	0	40	94	70	193	102	118	151	29								
186.11	39	0	0	0	0	80	119	111	133	238	10								
186.20	57	0	0	8	66	87	0	198	136	175	15								
186.30	50	0	0	0	65	27	55	245	148	164	16								
186.41	54	10	0	0	99	81	150	188	122	206	7								
186.51	83	0	0	0	54	23	54	101	153	100	0								
186.61	66	8	10	9	91	125	205	152	99	213	26								
186.71	46	0	0	29	56	77	122	195	132	156	8								
186.80	69	11	13	13	61	45	182	56	95	43	29								
186.90	74	6	5	29	131	46	166	162	92	0	23								
187.01	47	12	0	17	59	59	64	193	119	0	0								
187.10	71	30	19	0	31	55	84	321	109	220	0								
187.21	42	0	0	12	63	57	97	183	90	40	4								
187.30	44	6	0	0	65	138	108	205	111	159	22								
187.41	48	15	24	6	54	64	22	114	104	62	12								
187.51	61	7	0	17	68	46	25	61	129	193	10								
187.61	50	0	0	41	74	96	13	0	89	240	0								
187.71	37	5	0	26	67	95	52	81	119	103	0								
187.81	20	0	0	0	57	90	28	80	92	313	0								
187.91	38	0	0	19	99	135	111	26	136	365	27								
188.00	53	0	0	14	107	76	0	51	98	194	19								
188.11	63	13	10	51	58	106	75	161	128	377	21								
188.21	41	0	0	30	102	122	40	125	138	343	9								
188.31	51	8	0	66	89	85	40	103	130	280	29								
188.41	50	0	0	24	68	131	87	6	101	301	0								
188.50	65	0	0	14	67	56	75	91	132	255	13								
188.61	33	8	0	0	66	116	0	123	100	254	0								
188.71	64	0	9	7	108	71	189	102	128	52	0								
188.80	44	0	0	0	83	69	102	12	115	285	0								
188.91	53	8	0	13	39	124	125	127	125	148	33								
189.01	66	0	0	0	71	122	85	78	124	207	5								
189.10	46	0	0	9	46	40	72	56	123	148	31								
189.21	61	6	0	13	106	100	163	133	140	133	27								
189.31	59	0	0	0	88	100	100	74	119	270	8								
189.40	44	5	13	11	83	90	83	110	104	181	20								
189.51	40	0	0	29	98	88	76	62	126	172	19								
189.61	55	23	12	21	45	109	69	143	127	238	0								
189.71	54	35	16	31	77	30	35	150	105	165	0								
189.81	43	7	15	5	47	103	66	189	111	111	5								
189.91	28	16	11	0	13	143	79	144	137	109	31								
190.01	44	0	0	0	71	21	64	331	94	0	0								
190.11	67	23	6	7	11	51	62	274	98	77	14								
190.21	36	0	0	8	37	109	26	267	114	133	0								
190.30	58	16	0	5	29	53	111	154	141	0	0								
190.41	53	7	0	32	85	89	72	188	99	65	7								
190.51	28	5	0	13	80	53	0	57	97	341	5								
190.60	39	12	0	39	73	117	0	52	91	160	12								
190.71	43	0	5	42	70	132	83	75	122	196	12								
190.80	56	0	0	0	84	77	128	25	97	181	7								
190.90	35	0	0	27	109	131	113	30	113	369	0								
191.01	46	0	0	0	58	119	56	17	115	332	23								
191.10	32	0	0	0	137	99	118	31	128	373	14								
191.21	53	21	0	33	65	127	85	51	91	320	9								
191.31	31	0	0	0	96	90	0	63	91	416	0								

191.41	31	0	0	0	22	100	150	0	112	286	4								
191.51	61	18	0	30	60	98	136	30	122	326	0								
191.61	29	0	0	0	84	115	93	38	134	353	0								
191.71	48	0	0	17	0	73	137	66	96	293	0								
191.81	45	0	0	34	51	105	119	86	137	176	15								
191.90	45	5	0	13	153	114	84	71	116	240	32								
192.01	40	20	8	31	117	156	24	0	100	146	8								
192.11	61	9	0	7	104	98	123	151	104	293	6								
192.21	63	0	0	0	42	61	91	226	97	133	17								
192.31	57	0	0	0	63	100	81	120	135	204	0								
192.41	46	6	0	10	65	71	194	169	95	46	0								
192.51	33	7	0	52	78	78	131	129	128	247	24								
192.60	62	12	0	41	41	62	86	140	105	47	0								
192.71	67	0	20	0	102	69	109	194	82	144	12								
192.81	67	0	0	0	103	64	25	121	103	130	22								
192.91	39	6	0	0	95	87	158	34	84	49	12								
193.01	47	0	0	0	19	91	116	112	102	137	0								
193.10	38	19	5	49	80	53	119	147	94	0	0								
193.21	76	28	0	0	29	127	151	257	120	0	0								
193.31	62	28	12	10	44	71	73	304	105	8	0								
193.40	76	22	16	7	66	95	163	153	104	0	15								
193.51	42	7	15	12	47	69	104	165	79	0	10								
193.60	64	19	4	0	71	94	0	170	73	0	17								
193.70	70	0	0	12	72	56	70	283	101	0	0								
193.81	44	0	10	0	25	56	37	31	101	123	0								
193.91	57	0	0	19	110	103	142	5	115	451	19								
194.01	59	0	0	0	118	143	79	150	92	103	8								
194.11	35	0	0	15	60	100	105	85	105	390	0								
194.21	19	8	0	21	156	109	13	122	99	162	0								
194.31	51	20	0	40	86	79	19	32	113	428	10								
194.41	65	0	0	0	89	106	46	0	121	465	11								
194.50	14	0	0	48	95	118	0	42	79	103	29								
194.61	18	0	0	25	95	109	0	36	82	198	34								
194.70	16	0	0	29	57	82	38	125	97	173	0								
194.80	50	9	0	30	98	77	48	84	75	328	0								
194.90	40	16	0	14	39	107	6	54	76	233	13								
195.00	58	0	0	16	52	49	59	94	87	342	17								
195.10	79	0	0	55	81	121	59	138	91	145	13								
195.21	59	0	0	0	92	134	211	131	103	118	12								
195.30	43	0	0	0	63	67	190	175	52	200	0								
195.40	43	0	0	7	51	86	143	131	82	370	10								
195.50	38	0	0	70	26	84	236	124	108	240	0								
195.60	17	0	0	0	59	116	107	167	118	214	0								
195.70	61	10	0	8	65	98	147	159	76	0	22								
195.81	55	23	0	17	68	47	133	126	79	0	16								
195.90	65	0	9	0	0	60	118	260	89	0	0								
196.01	41	6	4	6	17	71	116	228	74	56	0								
196.10	78	0	0	0	58	51	174	360	86	7	0								
196.21	33	18	10	10	13	51	74	133	91	132	7								
196.30	64	29	7	0	62	32	23	156	82	254	18								
196.41	67	0	0	0	138	51	104	0	57	137	4								
196.50	61	0	0	51	67	88	9	19	69	147	21								
196.60	12	0	0	0	76	79	40	104	74	294	0								
196.70	57	0	0	27	86	112	80	92	88	204	10								
196.81	48	0	0	25	73	105	58	115	88	389	12								
196.91	45	18	0	0	30	104	125	79	66	61	0								
197.00	72	22	23	0	85	71	175	108	74	191	21								
197.11	32	0	0	0	96	104	108	125	83	243	0								

197.21	51	16	0	0	91	92	67	54	83	337	23								
197.31	76	17	6	0	88	119	73	173	59	154	34								
197.40	41	11	0	10	145	101	126	76	51	231	7								
197.51	68	14	0	10	35	32	108	261	87	73	0								
197.61	83	17	0	21	10	57	175	275	69	0	14								
197.71	57	25	14	25	41	86	48	296	63	0	18								
197.81	67	0	10	28	40	58	28	197	61	0	0								
197.90	54	24	8	0	41	46	82	182	74	250	20								
198.01	35	15	0	28	24	38	86	199	54	25	32								
198.10	65	0	0	31	94	73	56	22	47	338	8								
198.21	28	7	0	28	45	73	80	58	53	354	4								
198.31	31	0	0	30	81	103	68	44	69	318	0								
198.40	23	0	0	35	67	116	68	40	63	451	16								
198.50	48	0	0	0	56	80	86	93	61	257	29								
198.61	46	0	0	29	107	174	68	44	92	161	0								
198.71	12	0	0	5	85	75	5	43	66	227	0								
198.80	66	0	17	0	92	95	52	118	46	187	0								
198.90	40	0	0	24	61	82	114	45	55	381	32								
199.01	42	16	0	0	90	66	0	102	55	191	0								
199.10	52	0	12	49	87	107	43	126	75	243	17								
199.21	62	0	0	8	45	61	36	237	77	131	14								
199.31	71	0	20	18	57	67	148	33	68	241	9								
199.41	60	0	0	5	78	77	54	196	53	86	16								
199.51	50	30	15	17	90	104	201	105	70	143	45								
199.60	42	7	0	0	51	81	68	309	65	0	5								
199.71	60	15	10	58	60	64	97	262	80	0	9								
199.81	68	0	8	19	57	94	132	352	66	0	7								
199.90	54	21	12	0	23	77	61	220	66	0	0								
200.01	45	30	0	43	27	118	87	207	73	72	5								
200.11	34	0	0	33	110	76	39	85	59	0	10								
200.21	58	0	0	19	98	51	21	41	63	353	9								
200.31	40	0	0	4	97	101	12	49	70	236	12								
200.40	46	0	0	14	76	107	59	73	87	344	9								
200.51	57	9	0	20	102	116	81	8	90	241	0								
200.61	28	0	0	32	43	147	99	24	106	273	8								
200.70	50	5	12	15	53	130	44	77	53	235	8								
200.81	69	29	0	14	47	59	50	36	56	497	0								
200.91	44	0	0	12	98	105	45	103	73	186	28								
201.00	13	0	0	35	110	50	63	36	84	242	14								
201.11	50	0	0	36	136	96	149	78	67	284	0								
201.21	51	0	8	0	95	101	94	66	60	288	0								
201.31	29	0	0	4	54	124	236	110	61	228	35								
201.40	33	9	10	44	72	114	52	117	55	38	27								
201.50	54	8	11	14	47	109	179	187	69	56	0								
201.61	71	8	0	25	106	73	150	151	50	14	0								
201.71	69	22	15	13	39	65	191	161	81	73	0								
201.80	61	14	0	9	61	85	134	281	46	0	19								
201.90	58	0	0	0	37	114	211	217	75	0	9								
202.01	62	0	0	22	62	99	66	192	49	0	39								
202.10	66	0	0	12	18	78	92	192	51	251	0								
202.21	46	0	0	0	45	10	48	106	28	270	0								
202.31	29	9	0	52	70	95	105	66	67	315	12								
202.41	57	0	0	20	104	104	42	136	60	283	13								
202.51	50	0	0	0	15	33	88	68	40	479	9								
202.60	30	0	0	0	56	79	53	89	63	316	0								
202.70	53	0	0	0	53	85	0	27	109	117	0								
202.81	33	8	0	20	89	73	104	42	50	109	0								
202.90	68	0	0	0	52	104	115	239	63	72	23								

203.00	57	11	18	32	33	69	137	203	56	0	18								
203.11	50	29	0	19	0	77	165	224	60	0	0								
203.21	60	0	0	0	119	87	45	57	79	152	8								
203.30	35	0	0	0	34	85	241	54	70	229	9								
203.41	19	6	0	13	59	41	130	76	67	313	0								
203.51	48	0	9	34	117	110	30	152	71	423	0								
203.60	41	0	0	29	48	89	147	87	70	248	28								
203.70	54	8	0	29	102	71	133	104	76	451	26								
203.80	16	0	0	35	31	68	75	35	80	287	9								
203.91	48	4	0	44	49	114	22	21	34	403	15								
204.00	25	0	0	12	89	82	110	86	71	244	11								
204.11	36	0	0	22	95	99	194	0	89	395	0								
204.20	59	17	0	0	95	129	53	135	48	111	22								
204.31	62	18	0	0	65	70	115	142	60	272	0								
204.40	20	0	0	26	54	67	52	142	44	216	51								
204.51	58	7	0	8	53	5	54	142	71	0	11								
204.60	56	0	0	0	44	119	127	125	75	81	5								
204.71	54	13	14	7	0	0	116	213	68	0	17								
204.81	39	6	0	0	0	0	122	213	59	0	26								
204.90	47	11	0	0	55	90	179	269	51	0	48								
205.00	51	16	11	17	0	52	113	210	55	0	0								
205.11	76	34	33	0	86	39	0	197	90	0	7								
205.21	43	7	0	25	72	56	57	235	47	15	0								
205.31	32	0	0	0	87	103	58	9	50	109	0								
205.41	33	0	0	17	74	109	23	0	17	253	17								
205.50	34	0	0	0	60	59	0	57	72	347	20								
205.61	39	14	5	0	103	68	29	8	55	213	0								
205.70	21	0	0	0	120	123	151	0	58	196	0								
205.80	27	0	0	26	65	64	55	0	70	377	0								
205.91	34	0	0	41	115	99	174	9	47	219	0								
206.01	29	0	0	0	75	72	124	39	67	296	22								
206.11	32	0	0	0	80	116	77	106	60	377	18								
206.21	28	0	0	0	78	72	89	55	85	218	11								
206.31	33	0	5	27	124	76	104	101	34	132	19								
206.41	56	0	0	0	25	150	0	58	88	56	28								
206.51	60	18	0	7	93	140	77	0	87	416	16								
206.61	49	0	13	10	46	57	46	64	79	295	0								
206.71	66	0	0	27	122	58	107	222	58	170	5								
206.80	55	9	8	9	15	60	174	249	56	31	0								
206.90	34	30	0	11	0	34	89	231	56	0	0								
207.00	59	12	9	9	23	56	93	329	62	0	0								
207.10	49	0	0	0	65	52	172	283	62	27	0								
207.21	51	6	0	0	99	62	51	59	59	245	7								
207.31	13	14	0	24	61	47	0	0	55	297	19								
207.41	61	0	0	19	70	109	139	122	73	269	8								
207.51	18	0	0	17	74	82	193	115	61	174	0								
207.60	37	0	0	19	82	70	0	190	50	328	0								
207.70	36	0	0	22	124	77	109	162	55	345	0								
207.81	46	9	0	32	143	60	110	171	51	171	25								
207.90	40	5	7	0	124	102	85	43	55	111	19								
208.00	41	0	0	0	58	63	103	45	59	239	35								
208.10	60	4	0	25	30	101	141	103	72	0	8								
208.21	55	17	23	56	119	54	125	137	99	177	8								
208.31	63	29	10	18	23	48	154	293	106	49	18								
208.40	49	18	0	38	47	28	256	228	67	0	23								
208.51	53	0	0	0	150	82	177	356	61	0	0								
208.61	37	9	0	0	17	67	106	161	43	16	35								
208.70	56	0	0	18	63	73	151	56	57	119	22								

208.81	49	0	0	8	78	133	51	137	29	181	14								
208.91	48	0	0	0	105	138	116	60	94	312	6								
209.01	50	0	0	9	56	99	108	74	101	331	24								
209.11	65	0	0	8	75	117	80	0	58	315	15								
209.21	32	0	0	0	109	141	33	96	86	449	19								
209.31	53	6	0	8	94	137	122	75	84	325	11								
209.40	53	8	0	42	101	102	0	34	88	310	26								
209.51	22	0	0	29	138	127	241	75	79	245	0								
209.60	57	4	0	31	64	127	109	127	94	436	0								
209.71	36	0	11	7	85	71	48	52	73	225	15								
209.80	32	8	0	41	73	129	55	115	117	0	20								
209.90	68	17	0	0	57	34	177	109	108	157	24								
210.00	55	36	0	0	52	29	251	334	76	0	5								
210.11	51	0	0	6	38	36	173	143	77	0	10								
210.20	58	13	18	47	84	70	119	202	59	0	21								
210.31	41	0	0	0	65	89	0	106	75	81	0								
210.41	48	0	0	6	82	12	110	29	87	170	39								
210.51	37	0	0	0	40	88	58	116	58	371	26								
210.60	38	0	0	4	110	81	62	176	79	121	4								
210.71	20	0	0	42	62	131	166	176	74	154	5								
210.81	74	7	0	43	84	119	60	0	81	137	5								
210.91	41	0	0	24	100	49	197	87	80	0	9								
211.00	54	15	0	0	109	95	7	101	73	0	13								
211.11	41	27	0	0	22	159	104	149	69	58	9								
211.21	69	14	0	0	33	38	363	250	84	0	0								
211.31	56	11	0	0	139	35	259	196	65	0	6								
211.40	72	0	0	13	33	97	0	154	55	55	39								
211.50	35	0	0	0	126	94	0	145	94	272	13								
211.60	53	0	0	47	94	116	97	110	77	103	15								
211.70	40	0	0	0	85	80	0	63	69	267	25								
211.81	50	6	0	0	46	109	100	102	92	298	17								
211.91	43	0	0	19	33	97	120	84	56	323	10								
212.00	49	0	0	28	98	52	114	110	88	297	12								
212.10	62	0	9	0	57	87	167	197	60	197	31								
212.20	38	17	0	50	14	126	50	109	83	0	7								
212.31	79	28	20	41	86	17	93	219	54	39	0								
212.41	52	9	6	5	63	62	25	248	93	25	53								
212.51	41	22	11	0	59	42	108	247	62	0	30								
212.61	33	0	0	0	66	87	18	92	70	116	22								
212.71	29	0	0	10	58	68	0	59	68	337	8								
212.81	36	0	0	15	19	64	50	16	71	369	15								
212.90	80	13	0	0	91	95	118	71	89	293	24								
213.01	23	0	0	0	96	71	153	21	64	212	46								
213.10	47	4	0	0	60	70	224	59	85	161	22								
213.20	69	21	5	38	124	114	33	121	60	170	40								
213.30	65	8	0	10	63	60	162	219	68	168	29								
213.40	66	9	0	0	62	27	89	290	72	0	10								
213.50	44	0	0	0	89	73	74	202	54	40	0								
213.61	77	18	4	25	74	79	111	54	64	91	0								
213.71	52	0	0	6	106	61	53	35	30	210	5								
213.81	35	0	0	29	45	51	116	101	67	216	0								
213.90	67	0	0	17	95	80	170	118	76	214	24								
214.00	28	18	0	20	70	108	188	179	89	306	12								
214.10	55	0	0	14	111	106	128	71	76	287	11								
214.20	47	0	10	0	52	112	90	65	79	185	16								
214.30	34	0	7	15	60	59	180	247	62	56	28								
214.40	62	20	0	0	69	0	161	249	91	0	0								
214.50	52	7	6	13	44	30	241	237	72	0	37								

214.61	63	22	9	23	64	42	105	171	64	0	14								
214.71	42	0	0	17	27	21	23	128	39	89	0								
214.81	43	16	0	0	119	103	116	69	61	280	14								
214.91	46	0	0	15	51	82	27	102	53	358	0								
215.00	27	0	0	9	68	103	71	32	69	297	14								
215.10	37	0	0	12	96	73	102	7	83	240	27								
215.20	27	0	0	0	90	78	65	30	65	79	26								
215.31	76	0	0	0	31	89	126	237	72	0	10								
215.40	57	6	0	11	118	89	158	175	44	57	0								
215.50	39	0	0	0	68	321	57	76	63	19	8								
215.60	61	0	0	16	59	100	179	0	56	0	26								
215.71	63	8	0	23	70	132	149	123	72	124	13								
215.81	76	0	14	0	86	98	104	227	70	0	45								
215.91	86	17	0	0	23	34	198	137	50	0	17								
216.01	65	4	0	0	83	115	94	112	41	200	0								
216.10	9	0	0	13	62	72	154	41	52	378	37								
216.21	54	0	0	37	96	54	72	96	50	180	26								
216.31	32	0	0	0	14	118	60	51	84	286	32								
216.41	51	0	0	0	76	119	169	37	94	286	27								
216.51	0	0	0	27	71	58	236	135	82	166	20								
216.61	63	10	0	46	74	97	77	148	80	276	21								
216.71	48	22	19	20	42	92	248	193	90	41	21								
216.81	32	18	0	21	26	105	136	281	86	0	14								
216.90	55	5	0	61	55	94	84	154	78	0	33								
217.00	73	0	0	40	80	82	96	97	88	239	25								
217.10	58	7	0	20	31	85	99	12	56	285	42								
217.20	55	0	0	4	111	76	37	0	59	166	42								
217.30	46	0	0	19	143	124	238	136	106	93	34								
217.40	51	23	0	0	66	108	55	177	119	0	22								
217.51	45	0	0	0	47	60	98	163	94	0	39								
217.61	42	0	0	7	55	77	89	236	74	0	0								
217.70	34	0	0	35	102	150	137	58	105	240	24								
217.80	51	6	0	0	87	135	86	9	104	281	22								
217.91	59	0	0	24	80	116	72	95	109	246	35								
218.01	23	9	0	5	80	150	140	0	141	172	26								
218.11	49	33	19	19	113	132	129	62	164	118	9								
218.21	78	15	8	19	39	148	64	178	167	0	14								
218.30	52	0	0	5	50	87	231	305	170	0	37								
218.41	50	4	0	60	80	183	92	173	125	0	7								
218.51	46	0	0	6	46	101	74	0	81	161	0								
218.60	33	0	0	17	58	95	0	0	28	476	21								
218.71	23	0	0	32	82	66	42	0	41	646	14								
218.81	0	0	0	0	46	91	0	0	23	628	16								
218.90	26	0	0	6	34	63	0	0	34	595	11								
219.00	10	0	0	0	64	52	0	0	15	424	0								
219.11	0	0	0	8	51	89	0	0	37	528	6								
219.21	0	0	0	34	34	80	31	0	38	686	0								
219.31	9	0	0	0	71	78	0	0	33	690	0								
219.41	28	0	0	19	65	81	10	0	33	721	13								
219.50	13	0	0	0	67	63	24	0	24	694	0								
219.60	0	0	0	14	74	93	0	0	27	519	0								
219.71	0	0	0	12	87	49	0	20	38	579	0								
219.81	0	0	0	20	46	90	0	0	32	643	0								
219.90	0	0	0	15	79	78	0	0	28	580	0								
220.01	15	0	0	15	53	101	5	0	38	714	0								
220.10	22	0	0	0	41	102	6	0	30	727	0								
220.20	37	0	0	13	62	75	0	0	43	766	0								
220.30	15	0	0	16	59	49	11	0	32	647	6								

220.41	8	0	0	49	68	76	0	0	14	628	0								
220.51	6	0	0	0	95	62	0	0	17	698	11								
220.61	7	0	0	54	67	75	0	15	22	634	0								
220.71	0	0	0	21	49	83	0	0	26	604	0								
220.81	18	0	0	37	62	47	0	0	22	696	0								
220.90	15	0	0	34	87	60	6	0	26	811	0								
221.01	29	0	0	14	70	52	0	0	38	533	0								
221.11	29	0	0	30	53	81	0	0	47	772	5								
221.21	26	0	0	9	75	64	0	0	20	700	0								
221.31	0	0	0	15	63	71	0	0	43	453	0								
221.40	59	0	0	14	73	95	0	0	32	670	12								
221.51	9	0	0	25	77	30	8	0	25	628	0								
221.61	0	0	0	27	76	74	0	0	10	496	0								
221.70	0	0	0	22	61	77	0	0	10	539	12								
221.81	27	0	0	48	84	39	0	0	50	567	18								
221.91	17	0	0	24	77	77	0	0	47	625	17								
222.01	0	0	0	11	71	70	0	0	32	492	8								
