# Supplementary material for: Dendrochemical Challenge in Climate Science: Whether Chemical Elements in Wood Reflect the Fluctuations in Weather Parameters
Source: Plants (Basel). 2022 Nov 25;11(23):3240. doi: 10.3390/plants11233240 (PMC9740880; doi:10.3390/plants11233240)
Supplement: Supplementary file 1 [file plants-11-03240-s001.zip › Table S26.pdf]

Table S26. Scots pine #2. Initial core. After alcohol extraction. After HCl extraction.

(green) inner border of the bark
(blue) pith

Scots pine #2. Initial core.

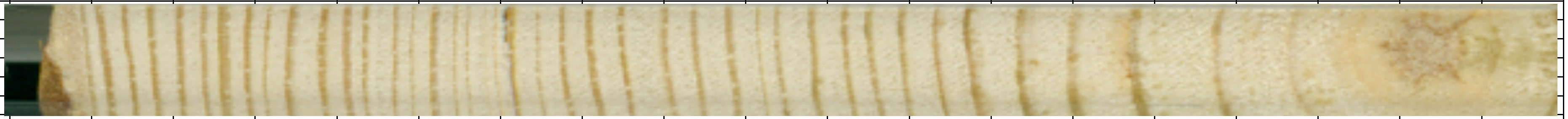

position (mm	Al	P	S	Ti	Mn	Fe	Cu	Zn	Ca	Sr	K
151.01	14	8	27	0	77	77	0	0	48	799	24
151.1	0	0	5	5	95	60	0	0	40	831	10
151.21	11	0	0	0	52	90	0	0	50	949	15
151.31	0	0	0	0	61	111	0	0	56	756	11
151.41	18	0	17	11	72	113	0	0	76	723	22
151.51	0	7	18	0	80	132	0	0	3646	1081	55
151.61	27	0	39	0	131	160	0	0	15493	695	252
151.71	18	0	29	0	139	133	0	0	18855	662	348
151.81	5	0	19	4	188	145	0	55	12739	755	513
151.91	20	39	50	0	208	125	0	45	12883	764	1655
152.01	0	67	72	0	312	113	0	59	19527	661	3794
152.11	35	68	115	0	392	168	89	81	26403	404	6024
152.21	19	55	127	0	530	180	188	283	25986	390	7120
152.31	20	82	93	0	642	197	99	385	18929	224	7202
152.4	24	60	108	22	539	142	195	372	15472	125	7024
152.51	36	62	94	0	546	141	176	324	15322	422	7023
152.6	6	70	91	6	537	145	97	325	14324	404	7396
152.71	28	68	110	0	549	165	176	443	13543	277	7068
152.8	18	65	108	0	495	144	95	408	15222	147	7401
152.91	24	68	113	16	495	198	140	362	15749	388	7836
153.01	5	79	117	0	525	168	135	347	16860	372	8400
153.11	16	53	126	0	591	169	119	303	18984	303	9118
153.2	23	41	117	0	626	216	166	290	22346	403	9462
153.31	7	49	115	0	733	148	163	399	25680	264	10116
153.41	23	90	128	0	642	92	167	429	24046	170	10430
153.51	29	67	116	0	642	83	131	401	19776	56	9471
153.61	29	54	64	0	425	111	181	392	13663	0	7779
153.71	8	29	61	0	324	87	106	361	7031	143	4849
153.81	6	25	38	0	183	52	186	137	3078	231	2701
153.91	22	22	50	0	170	89	166	122	1624	422	1649
154.01	0	21	28	0	195	124	82	126	1299	113	1213
154.11	25	19	30	0	156	94	138	70	1250	256	1242
154.21	44	37	39	0	154	83	112	97	1302	0	1255
154.31	6	25	37	0	146	111	150	146	1285	219	1141
154.41	18	32	65	7	137	65	351	38	1252	0	1070
154.51	0	9	17	0	137	61	226	152	1293	65	981
154.6	4	19	10	0	142	47	205	60	1257	88	875
154.71	0	7	39	0	165	52	119	139	1404	78	883
154.81	0	12	32	0	181	66	232	75	1486	199	924
154.91	32	11	57	0	193	87	26	8	1594	380	876
155.01	15	31	58	0	203	128	164	135	1715	249	905
155.1	21	29	47	0	175	94	194	130	1689	179	863

155.21	0	10	27	0	165	65	148	148	1852	440	870								
155.31	11	18	34	10	162	92	320	151	1685	211	864								
155.41	6	15	45	0	173	81	227	140	1662	0	855								
155.5	14	24	52	16	183	89	264	153	1549	96	859								
155.61	0	20	36	0	126	153	317	188	1647	388	863								
155.71	12	25	31	0	164	162	274	232	1555	375	856								
155.81	15	22	35	0	146	26	207	157	1680	245	781								
155.91	14	14	44	0	177	124	219	21	1660	351	784								
156	0	19	40	0	123	158	276	255	1749	303	785								
156.11	12	4	26	0	209	158	214	115	1601	370	740								
156.21	25	18	51	47	207	79	269	168	1717	466	817								
156.3	0	0	41	0	208	144	223	148	1934	321	916								
156.41	23	37	37	0	238	103	274	266	1769	59	950								
156.5	0	7	11	0	172	42	331	292	1612	17	801								
156.61	6	12	37	0	141	57	333	299	1412	0	739								
156.71	9	16	30	0	141	116	304	138	1473	56	760								
156.81	8	17	42	0	187	105	194	221	1556	140	785								
156.9	13	16	39	13	215	115	177	44	1630	288	736								
157.01	12	26	21	24	193	153	189	88	1736	395	721								
157.1	7	19	32	0	231	128	125	196	1818	499	781								
157.21	4	17	60	0	168	113	220	98	1999	339	787								
157.3	9	19	26	0	210	193	198	114	2028	351	809								
157.41	6	5	23	0	230	156	319	139	2059	313	848								
157.51	0	0	67	0	205	125	248	192	2210	363	930								
157.6	26	0	31	0	201	67	253	173	2176	0	965								
157.71	12	15	28	0	185	62	351	317	1668	0	873								
157.81	26	0	38	0	114	84	253	316	1391	0	803								
157.91	0	19	46	0	176	0	339	177	1369	21	675								
158	14	17	24	0	199	67	166	191	1437	132	551								
158.11	6	5	19	0	190	86	166	67	1578	253	628								
158.21	32	0	38	11	187	115	159	0	1602	196	682								
158.3	10	18	43	0	149	117	120	94	1730	161	666								
158.41	5	5	36	0	220	136	159	123	1951	365	779								
158.51	0	5	46	0	200	135	138	0	1916	320	829								
158.61	12	9	35	0	201	151	167	124	2214	178	871								
158.71	26	19	43	0	197	106	235	84	2239	0	981								
158.81	17	13	14	0	167	18	229	307	1765	107	837								
158.91	33	17	42	0	174	86	264	147	1703	8	754								
159	16	14	44	0	157	267	322	186	1713	0	758								
159.11	23	32	39	0	216	223	59	63	1870	74	787								
159.2	0	25	40	0	260	107	130	115	1916	109	789								
159.31	13	24	58	0	236	97	174	56	2041	388	750								
159.4	10	33	37	0	235	62	151	88	2132	49	840								
159.51	0	0	23	0	110	60	222	322	1849	36	839								
159.61	0	11	12	0	90	23	242	215	1442	90	674								
159.71	0	31	34	0	189	21	217	225	1295	0	604								
159.81	25	4	7	0	148	95	299	256	1345	0	601								
159.91	5	17	23	0	158	62	240	284	1685	10	719								
160.01	11	5	39	0	166	31	118	82	1958	218	816								
160.11	14	33	53	0	216	55	95	61	1990	261	733								
160.21	10	15	25	0	234	79	102	138	2042	336	686								
160.31	0	7	34	0	182	128	153	125	2029	303	721								
160.41	0	12	62	0	276	102	106	122	2134	172	775								
160.51	4	12	31	0	245	113	122	71	2210	265	839								
160.61	20	17	44	0	212	76	276	154	2304	0	860								
160.7	19	0	31	51	209	37	279	399	1928	0	780								
160.81	27	6	51	0	167	48	205	236	1513	0	728								
160.91	19	32	51	0	216	24	222	247	1578	0	753								

161.01	16	12	27	30	226	115	146	118	1780	116	660								
161.11	13	31	32	0	262	77	166	197	2036	129	688								
161.21	29	9	30	0	206	98	121	121	2117	303	706								
161.31	0	5	31	0	222	83	122	152	2118	442	746								
161.41	17	0	48	0	286	60	98	183	2123	185	829								
161.51	6	14	34	0	215	31	276	170	1876	125	804								
161.61	12	29	36	0	156	105	192	271	1822	0	784								
161.71	12	6	32	0	185	64	140	120	1806	221	754								
161.81	0	11	31	0	214	169	145	112	1836	237	762								
161.91	0	0	21	0	191	118	218	98	1843	414	634								
162.01	15	20	42	0	261	66	45	109	1746	376	588								
162.11	0	20	36	0	204	95	35	110	1866	368	652								
162.21	18	0	17	0	215	85	159	265	2027	383	753								
162.3	44	29	44	0	156	138	166	118	2357	294	864								
162.41	0	0	17	0	267	110	221	281	2402	109	931								
162.5	28	16	44	0	178	90	219	357	2204	0	798								
162.61	0	16	40	0	180	27	298	283	1705	0	699								
162.71	0	25	37	0	116	0	278	331	1703	0	725								
162.81	23	15	35	0	217	109	167	181	1653	0	740								
162.91	21	9	21	0	172	70	192	177	1780	221	697								
163	0	5	20	0	196	78	157	191	1837	311	732								
163.11	0	12	39	0	243	112	71	172	1978	343	719								
163.21	0	28	33	0	156	91	173	218	2065	98	667								
163.31	0	0	13	8	160	98	222	319	2124	443	646								
163.4	0	0	37	0	205	83	91	94	2050	155	657								
163.51	12	14	36	20	214	50	151	36	2121	270	732								
163.61	0	8	20	0	235	93	60	172	2179	504	709								
163.7	14	31	23	0	237	86	173	113	2245	402	750								
163.81	0	0	28	0	240	55	170	294	2447	211	810								
163.91	17	28	42	0	183	74	321	281	2248	0	801								
164.01	15	10	38	0	205	55	255	339	1969	0	821								
164.11	0	7	0	0	153	14	156	282	1679	0	648								
164.21	0	16	21	0	236	78	261	232	1841	138	604								
164.3	27	13	33	0	236	93	81	224	2047	115	639								
164.41	0	18	44	0	220	105	55	173	2024	323	636								
164.5	11	45	43	0	161	99	98	120	2247	209	625								
164.61	19	16	40	0	200	109	112	172	2309	303	633								
164.71	7	11	34	7	186	101	112	55	2170	310	680								
164.8	7	5	38	0	230	109	141	134	2334	204	710								
164.91	0	16	20	0	226	101	246	168	2331	259	754								
165.01	10	7	0	0	139	47	234	228	1946	0	709								
165.11	13	19	26	0	204	80	196	257	1632	0	673								
165.21	21	0	44	0	141	23	193	169	1622	0	672								
165.31	5	15	26	0	159	74	254	212	1642	251	596								
165.41	6	0	13	0	201	63	126	109	1966	316	615								
165.51	12	4	17	6	216	87	138	12	2083	418	581								
165.61	0	8	48	0	208	95	86	127	2114	195	585								
165.71	12	5	32	0	226	74	85	168	2332	259	704								
165.8	11	45	30	0	235	47	289	340	2296	0	882								
165.91	0	13	24	0	135	80	230	271	1908	0	804								
166.01	0	6	29	0	145	60	205	278	1628	88	614								
166.11	0	19	27	0	201	60	221	165	1564	137	573								
166.21	10	10	49	0	204	88	180	220	1716	197	596								
166.31	9	18	15	0	182	101	93	88	1778	386	567								
166.41	24	9	40	0	179	139	151	35	1870	318	589								
166.51	0	13	54	0	118	96	100	127	1967	327	584								
166.61	8	0	44	9	204	107	24	88	2069	475	605								
166.71	0	0	33	10	234	140	51	96	2094	428	635								

166.81	14	9	19	0	218	145	71	237	2227	552	610								
166.91	20	17	35	0	192	182	113	256	2312	473	728								
167.01	0	0	23	0	260	85	118	126	2484	290	748								
167.11	0	35	33	18	241	98	173	320	2574	115	889								
167.2	0	10	19	0	195	68	218	364	2310	0	900								
167.31	0	12	36	0	175	82	281	177	1980	0	723								
167.4	27	25	39	0	219	48	160	234	1791	158	574								
167.51	11	17	46	0	160	126	134	131	2001	274	578								
167.61	0	10	16	0	194	110	168	93	2105	459	613								
167.7	0	6	33	0	168	138	120	130	2283	472	620								
167.81	0	0	16	0	255	45	109	133	2582	398	675								
167.9	5	15	28	0	210	89	238	213	2503	136	837								
168.01	0	0	33	0	211	85	191	242	2311	0	836								
168.1	0	0	29	0	174	99	209	191	2164	297	769								
168.21	0	0	26	0	198	74	200	200	2288	125	780								
168.31	24	11	20	0	296	75	162	62	2119	119	719								
168.41	25	5	25	34	205	128	144	243	2173	348	672								
168.5	16	13	49	0	260	84	95	154	2335	350	686								
168.61	26	17	39	29	241	70	171	186	2330	438	694								
168.71	14	9	17	0	235	43	177	147	2610	153	763								
168.8	21	17	50	0	208	82	167	314	2127	145	783								
168.91	19	6	39	0	228	70	227	289	1688	0	684								
169.01	14	23	22	0	136	21	214	301	1643	0	672								
169.1	0	27	17	0	186	53	140	199	1708	54	618								
169.21	0	8	37	0	213	92	82	156	1807	160	602								
169.3	0	17	16	0	248	96	102	99	1943	307	547								
169.41	0	17	33	0	237	74	0	174	2338	251	570								
169.51	0	0	44	0	240	76	43	173	2269	460	576								
169.61	17	15	43	30	199	121	102	88	2360	406	554								
169.7	0	0	27	0	269	65	14	13	2524	422	679								
169.81	0	24	28	0	202	99	168	171	2535	113	797								
169.91	0	6	22	0	175	13	170	234	2217	35	728								
170.01	0	20	28	0	230	146	125	178	2097	0	633								
170.11	0	0	36	0	208	107	104	156	2267	504	628								
170.21	0	14	10	0	249	144	210	102	2404	254	632								
170.31	10	5	25	0	182	110	287	178	2542	132	803								
170.41	24	7	51	0	236	73	269	202	2560	0	913								
170.5	4	15	20	0	259	58	180	234	2288	0	733								
170.61	13	7	26	0	276	72	177	307	2281	317	660								
170.7	22	0	19	0	259	124	69	5	2266	420	616								
170.81	13	11	21	0	247	74	102	27	2286	432	532								
170.9	14	16	16	0	247	137	120	54	2203	579	569								
171.01	0	24	29	0	209	374	101	46	2414	471	652								
171.11	0	0	40	0	228	267	177	154	2910	396	779								
171.2	20	0	27	0	248	104	202	245	3130	190	894								
171.31	22	7	21	0	273	99	115	289	3013	133	790								
171.4	0	6	47	0	295	87	176	147	2828	272	673								
171.51	0	0	40	0	302	167	200	121	2731	56	692								
171.6	7	25	52	0	269	86	278	215	2738	75	799								
171.71	0	17	32	0	246	59	215	204	2592	107	806								
171.81	26	24	26	0	242	83	152	151	2502	40	688								
171.91	14	0	40	0	267	121	156	167	2327	210	604								
172.01	21	24	34	0	296	123	76	130	2075	335	477								
172.11	12	0	37	0	178	114	193	117	1684	539	404								
172.2	18	20	35	0	198	134	63	105	1757	572	441								
172.31	0	11	33	0	222	104	111	226	2801	211	789								
172.41	16	24	30	0	313	134	396	270	2875	58	892								
172.51	18	10	25	0	259	84	237	419	2322	0	682								

172.61	0	15	18	0	227	143	227	318	2043	0	577								
172.71	29	7	40	0	218	90	187	209	2171	236	572								
172.81	0	0	37	0	280	58	45	110	2237	213	589								
172.91	0	5	32	0	267	84	178	43	2288	363	604								
173.01	0	0	28	0	292	100	158	68	2309	316	571								
173.11	10	0	25	0	286	91	115	176	2467	477	585								
173.21	0	10	17	0	266	159	190	114	2648	188	639								
173.3	0	0	24	0	340	187	139	240	2680	382	670								
173.41	0	0	31	0	303	119	175	236	2746	279	685								
173.51	20	7	24	0	246	59	285	269	2821	258	810								
173.61	5	0	33	0	347	70	178	351	2692	55	783								
173.71	0	30	29	0	228	95	197	334	2346	0	740								
173.81	32	8	42	0	261	52	246	135	2208	176	557								
173.91	5	15	33	0	273	57	226	168	2410	357	623								
174	12	8	41	13	278	99	201	160	2461	365	662								
174.11	8	7	39	0	281	88	187	159	2489	338	703								
174.21	20	23	41	0	267	107	98	63	2593	481	686								
174.3	0	10	6	0	266	63	145	169	2729	373	677								
174.41	14	7	41	0	280	156	89	91	2724	200	655								
174.51	0	5	36	0	289	74	192	233	2901	384	701								
174.6	0	21	42	13	293	40	186	264	2856	121	780								
174.71	10	14	42	0	290	57	262	454	2531	0	752								
174.8	0	11	8	0	291	89	232	207	2286	72	692								
174.91	0	0	41	0	305	93	237	198	2327	176	606								
175	14	7	9	0	301	21	207	195	2492	296	578								
175.11	0	21	25	0	297	86	76	150	2615	179	595								
175.21	0	0	12	0	325	39	158	195	2650	322	602								
175.31	0	10	20	21	308	68	164	163	2727	409	553								
175.41	12	19	11	0	365	107	100	217	2713	477	618								
175.51	0	25	38	0	302	140	182	167	2703	528	616								
175.61	8	0	27	0	269	148	117	198	2989	408	681								
175.71	18	31	39	0	280	68	177	169	2980	105	695								
175.8	0	7	20	0	328	23	184	292	2736	0	766								
175.91	7	23	17	9	337	100	268	268	2447	76	748								
176.01	12	20	47	0	284	49	279	338	2236	0	663								
176.1	0	14	56	0	240	44	194	285	2196	77	633								
176.21	0	0	39	0	294	60	211	231	2251	29	597								
176.31	0	5	17	11	266	95	153	169	2340	313	566								
176.41	32	5	15	0	299	69	67	228	2476	325	534								
176.5	10	9	28	0	305	170	71	178	2641	383	594								
176.61	25	22	38	0	334	100	84	108	2607	318	649								
176.71	15	19	33	0	328	83	46	47	2753	384	664								
176.81	0	0	16	6	381	174	35	88	2841	457	622								
176.91	20	5	30	5	370	159	151	158	2905	429	643								
177	0	23	63	22	371	111	204	146	3055	282	630								
177.1	0	24	47	17	378	160	157	154	3349	302	743								
177.21	0	0	15	0	349	79	221	245	3247	417	810								
177.31	28	0	34	0	382	60	225	358	3204	162	732								
177.41	10	28	14	0	326	68	197	271	2841	0	776								
177.51	4	22	30	0	322	0	180	204	2452	0	725								
177.6	0	6	38	4	350	76	186	222	2445	0	660								
177.71	15	23	38	0	327	43	228	209	2268	166	562								
177.81	10	0	27	0	291	82	172	171	2261	283	526								
177.91	18	4	36	0	290	89	105	148	2297	300	554								
178	0	12	28	0	317	70	67	177	2446	230	630								
178.11	13	18	39	0	358	117	140	232	2646	476	686								
178.21	7	8	43	0	337	137	8	157	2807	264	638								
178.3	27	6	35	0	329	80	50	116	2595	536	587								

178.41	0	23	22	0	327	88	106	122	2725	442	572								
178.5	10	0	33	0	360	92	119	131	2874	267	677								
178.61	0	0	27	0	384	102	53	68	3011	292	624								
178.71	0	14	28	18	359	103	113	164	2843	547	655								
178.8	12	0	18	0	330	61	63	210	2908	354	659								
178.91	0	9	57	0	415	156	113	124	3152	211	685								
179.01	8	0	24	0	368	104	229	157	3042	218	789								
179.11	20	14	30	0	328	108	262	346	2679	40	669								
179.21	14	0	32	0	393	87	285	267	2477	0	633								
179.31	40	32	37	0	356	30	202	299	2179	0	649								
179.41	0	12	24	0	298	11	213	288	2080	65	652								
179.51	18	12	39	18	343	57	246	230	2199	63	507								
179.6	14	27	33	0	396	63	135	68	2382	145	554								
179.71	0	6	25	0	342	75	103	206	2651	173	673								
179.8	14	0	26	0	357	101	118	192	2858	279	731								
179.91	8	30	15	0	351	90	126	101	2898	399	699								
180.01	8	26	18	5	373	121	115	306	2947	230	717								
180.11	0	0	27	0	372	132	45	21	2839	340	686								
180.21	0	32	29	0	356	63	115	28	2956	361	647								
180.31	13	21	38	0	428	48	171	195	3033	280	734								
180.41	4	25	43	0	434	124	116	202	3020	178	774								
180.51	0	0	33	0	319	118	202	274	2644	125	661								
180.6	0	0	19	0	359	39	133	238	2394	0	650								
180.71	0	24	26	0	328	42	149	247	2043	0	683								
180.81	0	17	32	0	318	64	286	245	1941	28	568								
180.91	18	38	38	0	302	63	120	209	1904	63	540								
181.01	14	21	24	0	280	89	190	179	1943	357	545								
181.11	8	10	25	0	300	50	35	169	2295	110	603								
181.21	27	10	42	0	278	81	118	185	2379	289	653								
181.31	5	0	13	0	360	134	81	164	2476	332	697								
181.41	27	12	15	0	359	88	117	95	2656	315	684								
181.51	0	32	33	0	401	121	98	115	2687	233	665								
181.6	18	31	55	0	413	54	88	173	2747	233	616								
181.71	0	0	53	17	431	118	88	171	2829	315	633								
181.8	0	32	51	0	374	64	132	89	2983	392	645								
181.91	11	0	53	25	360	55	112	282	2810	212	711								
182	0	0	0	0	389	18	222	321	2466	116	663								
182.11	0	6	18	0	320	133	198	271	2236	0	676								
182.2	18	6	29	0	302	8	250	361	2039	0	666								
182.31	5	12	36	0	290	59	210	301	2086	0	662								
182.4	12	0	13	0	306	73	271	253	2118	82	690								
182.51	39	18	58	0	320	80	108	315	2379	168	663								
182.61	0	0	21	11	345	50	104	122	2296	220	649								
182.71	8	20	39	0	312	113	52	132	2318	209	659								
182.81	10	23	38	0	386	59	103	114	2588	381	602								
182.91	11	21	40	17	321	81	72	106	2855	608	707								
183.01	8	0	22	0	386	45	90	65	2941	332	708								
183.1	0	13	27	0	408	22	102	17	3010	309	700								
183.21	19	16	41	0	389	94	119	116	3206	366	726								
183.31	25	21	24	0	425	38	167	258	3340	457	824								
183.4	0	6	9	0	458	59	183	268	3176	233	813								
183.51	0	10	38	0	366	57	252	283	2985	64	739								
183.6	4	32	23	5	348	75	254	235	2868	131	752								
183.71	0	0	8	0	432	101	255	198	2713	208	694								
183.81	11	24	32	5	441	74	221	169	2763	375	684								
183.91	11	8	19	0	390	93	177	193	2902	212	681								
184.01	9	22	49	0	444	100	150	60	3021	347	692								
184.11	0	22	26	15	402	97	79	162	3069	316	787								

184.21	0	0	37	0	411	64	172	219	2811	84	826								
184.31	6	43	40	0	314	27	185	311	2436	75	769								
184.41	15	27	34	0	333	43	266	288	2272	0	646								
184.51	30	30	23	0	339	56	160	296	2144	265	727								
184.61	11	17	15	25	352	83	198	310	2412	261	661								
184.71	20	16	32	0	356	85	183	202	2526	275	775								
184.81	15	0	30	0	357	71	137	126	2626	308	726								
184.91	0	0	28	0	371	65	112	169	2589	276	705								
185.01	0	0	46	0	319	130	107	146	2577	246	714								
185.11	25	0	34	0	400	127	180	105	2671	333	700								
185.21	24	12	48	0	415	118	118	109	2785	451	702								
185.3	20	6	22	0	402	74	136	372	2823	309	718								
185.41	17	27	39	0	408	138	94	232	2956	358	629								
185.51	12	39	46	0	436	55	129	111	2986	465	708								
185.61	0	0	18	0	431	80	179	185	3225	180	722								
185.71	4	8	20	0	407	66	137	157	3057	231	777								
185.81	10	35	26	0	376	65	302	357	2823	0	755								
185.91	0	0	30	0	403	16	260	282	2289	17	647								
186.01	4	19	32	0	370	88	257	272	2193	0	620								
186.1	0	0	26	0	354	66	275	242	2065	119	620								
186.21	22	7	34	27	338	118	257	258	2330	291	589								
186.3	0	0	22	0	363	113	191	218	2411	400	624								
186.41	13	0	16	0	394	165	131	97	2512	275	693								
186.5	0	10	39	0	388	70	145	313	2601	237	692								
186.61	18	10	48	0	337	134	197	168	2633	483	738								
186.7	13	25	36	30	398	98	127	71	2828	270	666								
186.81	16	24	35	0	407	62	101	84	2658	177	756								
186.91	10	45	30	0	377	110	97	152	2771	321	725								
187.01	23	21	30	0	344	91	84	108	2932	101	740								
187.11	15	18	24	0	422	110	169	181	2984	35	844								
187.21	11	0	35	0	413	99	201	278	2803	89	884								
187.31	5	25	40	0	400	11	213	236	2654	0	795								
187.41	9	18	38	0	376	83	102	363	2418	0	859								
187.51	10	0	15	0	421	102	279	197	2475	104	797								
187.61	0	14	31	0	316	59	138	143	2511	178	755								
187.71	0	0	16	0	382	47	119	234	2607	69	736								
187.81	16	6	22	0	377	57	197	146	2738	316	661								
187.91	0	21	43	0	370	81	24	85	2696	489	735								
188.01	18	8	55	0	379	94	220	62	2919	408	774								
188.11	0	27	28	0	405	105	161	84	3026	377	844								
188.21	0	9	21	0	375	58	225	219	2873	204	820								
188.3	5	0	14	0	345	26	157	329	2853	0	808								
188.41	14	8	14	0	408	18	244	312	2784	109	747								
188.51	10	27	25	0	386	59	283	253	2556	230	648								
188.61	5	23	49	0	360	23	187	242	2431	64	713								
188.71	0	4	29	0	360	25	151	307	2589	68	778								
188.81	12	15	28	0	350	99	248	227	2717	307	759								
188.91	0	12	29	0	393	68	113	182	2812	73	668								
189.01	0	0	34	0	376	61	159	255	2660	415	659								
189.11	0	0	35	0	374	118	170	83	2502	425	720								
189.21	7	0	52	0	377	106	96	74	2633	391	690								
189.31	0	0	29	0	356	92	121	22	2617	398	753								
189.41	7	22	48	0	398	83	83	107	2840	318	750								
189.5	0	24	25	0	392	55	50	90	2983	333	728								
189.61	8	0	20	0	368	110	130	227	3092	269	868								
189.71	0	4	24	0	429	51	243	280	3088	0	795								
189.81	21	9	32	0	391	23	279	228	2793	116	696								
189.91	29	24	22	25	379	57	246	344	2442	0	592								

190.01	0	17	34	0	334	58	297	269	2232	0	561								
190.11	30	8	24	0	344	84	54	209	2310	49	634								
190.21	17	0	31	0	226	95	121	192	2409	210	716								
190.31	0	0	47	0	376	97	135	116	2503	406	779								
190.41	7	28	45	24	263	42	127	182	2478	389	662								
190.51	0	19	40	0	345	56	134	178	2559	392	651								
190.61	16	5	11	0	359	69	106	86	2574	485	662								
190.71	0	4	30	0	363	77	166	103	2865	353	685								
190.81	0	16	18	4	375	84	111	193	2853	434	710								
190.9	10	8	40	0	365	58	127	227	3071	308	660								
191.01	31	24	56	9	424	101	124	126	3066	330	640								
191.11	23	7	32	0	411	124	143	83	3185	219	718								
191.21	21	14	30	0	416	107	199	210	3307	329	659								
191.3	0	15	33	0	532	64	183	293	3136	0	642								
191.41	25	24	21	6	436	26	261	357	2833	122	625								
191.51	6	19	14	0	411	122	282	212	2580	0	540								
191.61	4	0	28	0	401	50	227	239	2517	104	558								
191.7	0	12	31	0	368	95	128	221	2461	307	567								
191.81	24	18	26	0	325	42	88	164	2562	185	458								
191.91	26	9	30	40	359	89	137	193	2704	357	481								
192	11	0	28	0	371	52	151	50	2676	303	499								
192.11	8	5	32	0	392	84	100	61	2771	334	511								
192.2	0	9	38	0	397	80	127	156	2886	292	503								
192.31	33	0	30	0	473	88	171	37	2986	237	506								
192.41	8	22	30	0	339	62	137	106	3039	278	555								
192.51	14	23	25	0	466	146	126	171	3157	482	508								
192.61	30	34	37	0	395	81	190	131	3171	268	564								
192.71	0	0	37	0	430	36	222	253	2979	0	539								
192.81	0	6	25	0	368	34	300	354	2835	0	518								
192.91	9	10	34	0	392	33	263	325	2688	0	448								
193	10	8	11	12	335	68	294	278	2569	0	505								
193.11	0	0	0	0	363	0	222	321	2572	22	450								
193.21	7	9	28	0	387	38	291	321	2644	0	454								
193.31	0	0	15	0	395	54	205	181	2777	117	497								
193.41	17	7	24	0	363	64	205	194	2704	179	486								
193.51	7	0	40	0	419	122	101	234	2809	259	535								
193.61	9	12	24	0	466	119	67	111	2871	211	510								
193.71	12	5	9	0	452	144	137	150	3023	264	512								
193.81	21	11	33	19	412	28	99	133	3026	320	506								
193.91	0	0	9	0	395	125	151	102	3136	599	497								
194.01	10	11	22	0	444	76	51	174	3059	285	566								
194.11	0	20	20	0	437	107	139	295	3114	231	527								
194.21	0	0	42	0	413	90	168	97	3206	332	532								
194.3	0	9	21	0	392	98	133	153	3329	366	493								
194.41	14	17	29	0	498	100	106	122	3349	404	543								
194.51	14	5	20	0	457	87	107	150	3285	436	550								
194.61	0	16	54	0	384	82	190	146	3603	291	584								
194.71	0	0	26	0	481	82	96	165	3540	149	502								
194.81	24	0	38	0	459	73	97	198	3673	235	581								
194.91	0	0	13	0	488	118	192	311	3556	171	557								
195.01	15	0	43	9	425	14	215	411	3147	0	536								
195.11	5	0	13	0	459	91	308	362	2780	0	471								
195.21	0	6	28	0	424	56	242	401	2589	60	473								
195.31	0	10	4	0	434	27	260	426	2491	157	477								
195.41	12	0	38	0	371	94	255	210	2728	127	523								
195.5	4	15	0	0	420	50	118	218	2872	24	492								
195.61	23	24	26	0	408	68	203	273	3071	107	474								
195.7	23	23	24	0	431	72	222	218	2988	164	527								

195.81	23	0	29	0	441	109	170	105	3264	0	519								
195.91	0	0	17	0	486	131	185	166	3321	224	524								
196.01	17	7	17	0	450	70	117	385	3468	348	556								
196.11	5	9	43	0	446	76	209	264	3571	351	541								
196.21	31	31	29	20	469	91	273	253	3487	203	518								
196.31	21	0	21	5	480	123	140	129	3635	285	545								
196.41	0	0	22	0	494	89	283	226	3902	142	558								
196.51	10	0	28	0	440	115	153	198	3725	422	559								
196.6	27	15	9	0	505	129	175	223	3845	337	479								
196.71	11	6	18	19	444	79	157	83	3764	200	548								
196.81	31	29	40	0	515	88	190	120	3579	264	524								
196.91	19	27	42	0	510	75	234	238	3692	400	584								
197.01	0	9	34	0	487	68	156	189	3796	239	603								
197.1	30	11	42	0	543	56	111	220	4114	82	622								
197.21	0	20	37	0	536	123	226	250	3885	117	609								
197.31	5	16	21	0	504	39	354	353	3484	95	472								
197.41	0	25	48	0	504	11	320	288	3239	0	527								
197.5	6	18	33	0	385	14	288	334	3120	0	540								
197.6	27	8	36	0	480	25	285	328	2990	0	492								
197.71	25	22	44	0	467	108	252	208	3138	0	505								
197.81	0	18	27	0	461	40	205	188	3309	195	542								
197.91	21	17	33	0	470	107	292	207	3395	139	555								
198.01	5	24	43	8	395	52	283	212	3646	247	568								
198.11	17	0	23	0	465	7	285	295	3833	158	593								
198.21	23	0	40	0	510	86	253	86	3907	256	615								
198.3	0	0	19	0	486	111	158	221	4108	352	614								
198.41	5	25	14	8	589	137	178	132	3937	218	616								
198.5	0	16	30	0	542	102	218	200	3965	450	687								
198.61	0	0	19	0	543	32	154	142	4107	319	663								
198.7	21	6	44	0	584	47	215	261	4352	357	719								
198.81	0	0	0	0	633	76	214	293	4475	158	802								
198.9	15	6	28	0	572	120	175	318	4635	250	817								
199.01	17	0	57	4	629	77	285	221	4686	371	823								
199.1	32	13	40	0	613	85	263	301	4884	303	846								
199.21	8	8	49	0	656	66	136	162	5022	121	822								
199.31	0	21	59	0	621	33	237	292	4976	187	859								
199.4	26	15	44	17	653	76	281	285	4946	66	917								
199.51	0	0	34	0	625	130	172	362	5049	59	880								
199.61	8	5	51	0	635	0	278	377	5345	71	892								
199.71	10	4	55	0	659	86	327	376	6444	114	764								
199.81	33	0	78	0	746	0	306	418	7893	97	792								
199.91	30	15	99	0	902	60	246	568	7731	166	750								
200.01	8	0	67	0	774	110	241	423	5502	130	696								
200.11	22	12	40	0	666	69	296	328	4318	98	598								
200.2	17	25	48	0	605	77	310	359	3648	0	607								
200.31	5	26	16	0	500	43	256	384	3296	21	467								
200.4	0	6	7	0	422	62	172	375	3183	19	504								
200.51	18	0	38	0	427	37	182	205	3269	116	501								
200.61	7	20	32	0	421	89	191	262	3422	223	544								
200.7	16	0	30	0	432	9	115	87	3481	79	496								
200.81	0	0	16	0	408	67	145	184	3382	349	516								
200.91	8	23	27	0	465	75	63	162	3691	158	545								
201.01	16	0	19	0	464	91	131	240	4021	370	524								
201.11	19	0	44	0	533	120	214	115	3834	351	538								
201.21	6	13	43	0	438	147	174	174	3499	358	500								
201.3	0	8	49	0	514	139	272	128	3433	498	491								
201.41	0	9	7	14	417	133	118	120	3519	162	514								
201.51	7	23	24	0	458	86	202	194	3632	388	481								

201.61	16	16	16	0	431	85	157	104	3469	201	515								
201.7	0	8	43	0	461	63	83	151	3526	471	470								
201.81	0	0	11	0	456	105	187	154	3526	251	478								
201.9	0	16	30	0	436	118	173	47	3507	305	526								
202.01	0	0	26	0	401	85	137	150	3678	460	527								
202.11	15	12	40	0	483	45	33	189	3700	563	578								
202.21	18	21	44	0	484	94	98	154	3793	470	611								
202.31	7	16	43	0	521	123	72	154	3648	468	532								
202.41	14	10	35	0	481	91	120	140	3878	449	543								
202.51	0	0	9	0	489	110	172	184	3974	386	549								
202.61	23	9	36	0	492	59	8	41	4135	292	642								
202.71	11	6	31	0	512	110	121	109	4175	396	631								
202.81	23	40	42	0	557	87	161	170	4314	506	642								
202.91	9	10	23	0	533	160	72	142	4494	325	703								
203.01	8	0	44	0	543	110	162	229	4513	417	603								
203.11	13	10	19	0	584	69	100	70	4735	240	783								
203.21	0	4	50	0	618	130	128	244	4815	292	752								
203.31	4	0	24	0	634	56	93	223	4926	518	799								
203.4	13	11	15	0	646	52	199	279	4692	239	723								
203.51	0	6	32	0	583	100	54	189	4809	231	739								
203.61	0	0	27	0	616	108	167	179	5030	17	760								
203.7	0	19	43	0	592	0	268	301	4871	67	735								
203.81	13	17	17	9	635	0	295	350	4233	109	671								
203.9	8	6	20	0	594	15	247	359	3765	122	618								
204.01	0	0	12	0	487	27	287	404	3452	72	532								
204.11	0	12	32	0	493	27	278	351	3494	64	559								
204.21	0	24	23	0	488	64	140	174	3472	243	541								
204.31	13	0	18	0	471	74	206	264	3514	401	534								
204.41	0	8	33	0	466	64	143	264	3726	160	513								
204.51	0	4	25	16	505	126	102	225	3781	288	577								
204.61	20	0	22	0	526	96	167	176	3745	252	563								
204.71	10	9	27	0	515	93	159	74	3834	155	525								
204.81	31	10	37	0	490	113	101	180	3937	445	559								
204.9	0	7	38	0	541	43	140	101	4208	238	549								
205.01	0	30	35	0	533	159	115	174	4476	181	551								
205.1	16	14	33	0	549	99	165	221	4215	88	559								
205.21	12	24	32	0	523	72	129	162	4027	355	566								
205.3	0	0	25	0	499	105	212	242	4101	270	563								
205.41	19	22	49	0	489	93	158	189	4154	372	583								
205.51	24	26	38	0	486	93	183	126	3971	152	575								
205.61	0	0	33	0	563	53	149	149	4066	466	597								
205.71	0	22	31	0	596	45	136	155	4010	587	551								
205.81	0	0	13	0	470	79	110	143	3922	192	569								
205.91	0	14	25	4	534	89	145	35	3969	336	647								
206	0	0	0	0	549	49	73	101	4125	383	629								
206.11	0	5	52	0	513	77	71	269	4116	228	604								
206.2	0	7	18	0	528	97	148	153	4192	394	621								
206.31	13	6	56	0	567	113	69	125	4286	224	620								
206.41	8	24	22	0	594	160	87	88	4471	305	623								
206.5	32	10	50	9	612	125	103	250	4356	434	606								
206.61	4	0	19	0	527	105	217	242	4556	416	667								
206.7	13	16	38	18	602	117	168	193	4801	360	663								
206.81	0	0	22	0	605	60	204	236	5069	571	789								
206.91	10	0	27	0	643	60	233	198	5259	236	753								
207.01	6	5	47	13	647	60	36	263	5346	70	806								
207.11	19	19	40	0	712	113	253	311	4692	101	659								
207.21	22	0	11	0	639	23	296	286	4495	146	680								
207.31	0	19	5	0	593	37	202	354	4237	144	671								

207.4	15	0	17	0	578	23	175	254	3884	95	613								
207.51	9	0	24	0	492	100	233	260	3809	232	636								
207.6	9	0	32	0	516	33	266	136	4038	152	612								
207.71	0	0	38	0	506	107	188	166	4166	483	628								
207.81	0	7	35	0	548	78	100	160	4574	145	601								
207.91	0	28	21	0	563	95	151	249	4687	231	602								
208	0	11	34	0	566	65	160	214	4592	368	649								
208.1	27	12	34	0	547	127	156	93	4413	337	671								
208.21	13	10	26	0	546	107	142	305	4353	297	645								
208.31	14	19	24	0	607	65	239	326	4369	421	686								
208.4	13	15	33	0	511	85	218	264	4320	380	652								
208.51	0	0	26	10	572	92	142	297	4243	288	645								
208.61	11	12	23	0	623	93	106	149	4365	137	706								
208.7	15	0	43	0	583	110	84	211	4586	256	785								
208.81	19	0	18	0	599	51	125	299	4653	572	784								
208.91	13	0	15	0	580	80	140	86	4811	262	805								
209.01	0	0	45	5	591	68	192	95	4974	246	885								
209.11	8	0	24	0	621	89	121	202	4904	213	873								
209.21	23	0	16	0	584	105	159	239	4890	412	832								
209.3	21	17	35	0	603	55	245	139	4824	364	856								
209.41	24	11	26	0	614	43	159	186	4819	284	778								
209.5	9	27	42	0	605	86	164	241	4931	374	830								
209.61	5	0	46	12	532	37	234	238	5265	218	814								
209.71	12	0	20	0	673	125	200	280	5425	240	802								
209.81	12	6	36	0	658	66	144	348	5166	81	807								
209.91	22	0	28	36	667	53	245	271	5281	325	813								
210.01	27	21	47	0	582	70	191	206	5166	154	761								
210.11	10	10	19	0	653	93	200	166	5161	261	746								
210.21	0	16	38	65	592	47	101	140	5495	211	727								
210.3	0	27	34	0	626	84	152	236	5456	142	699								
210.41	0	0	49	0	605	87	164	165	5292	191	647								
210.51	10	11	50	0	606	67	218	344	5405	232	696								
210.61	4	0	37	0	649	66	187	316	5458	380	693								
210.71	0	18	42	0	589	45	92	159	5114	242	704								
210.8	0	9	36	0	614	86	174	101	5188	329	674								
210.91	6	0	32	0	609	83	140	317	5300	192	681								
211.01	31	12	33	0	592	86	274	279	5247	267	685								
211.1	8	17	28	0	583	98	303	217	5170	290	620								
211.21	6	0	28	0	595	122	219	263	5317	212	672								
211.3	0	0	24	0	608	51	133	245	5723	345	633								
211.41	8	0	46	0	616	86	152	267	5921	74	721								
211.5	0	17	55	0	679	82	105	239	5834	339	678								
211.61	0	0	26	0	658	74	178	215	5888	344	661								
211.7	33	26	51	0	574	49	232	247	5907	371	622								
211.81	6	0	6	0	589	104	160	325	5825	389	714								
211.9	4	21	52	0	570	112	181	187	6109	475	729								
212.01	14	0	25	0	566	120	56	294	5858	447	700								
212.11	11	12	33	0	598	64	168	203	5716	611	659								
212.21	16	0	50	0	566	122	129	191	5821	364	645								
212.31	0	0	55	0	544	97	192	163	5620	345	700								
212.41	32	16	53	0	668	104	87	146	5554	357	663								
212.5	13	0	25	0	481	35	102	159	5638	376	705								
212.61	34	10	19	0	577	68	94	140	5909	441	745								
212.71	7	8	36	0	547	78	104	177	5781	416	777								
212.81	38	19	42	0	633	95	164	181	5819	590	673								
212.91	23	0	53	0	530	86	49	155	5948	482	754								
213.01	13	22	51	0	596	147	128	245	5897	641	706								
213.11	8	7	44	19	625	101	75	203	5810	371	689								

213.2	11	10	44	0	559	134	171	231	6067	402	690
213.31	13	0	59	0	622	90	141	142	6286	566	760
213.4	33	16	28	15	572	112	132	291	6207	502	726
213.51	7	19	42	0	612	79	117	232	5817	361	741
213.61	8	14	46	0	606	116	139	173	6058	414	741
213.71	0	11	43	0	584	67	90	186	6236	529	729
213.8	12	6	46	0	643	122	132	183	6294	328	765
213.91	16	14	30	0	604	54	153	203	6399	419	762
214.01	17	16	49	0	604	50	188	240	6422	363	727
214.11	12	17	39	0	635	91	198	155	6202	291	751
214.2	10	11	24	0	691	104	193	215	6314	460	743
214.31	11	0	38	0	638	74	161	303	6849	508	739
214.4	11	0	44	0	654	80	200	275	7319	235	777
214.51	19	25	52	0	683	63	223	304	7168	23	749
214.61	23	5	84	0	664	107	301	327	6321	386	696
214.71	7	28	40	0	634	51	218	321	5503	281	621
214.81	18	25	51	0	597	99	232	355	5061	99	638
214.91	11	17	41	0	515	59	118	246	4926	146	678
215.01	6	12	46	17	529	99	168	181	5132	42	630
215.1	38	23	29	0	593	90	219	327	5007	0	633
215.21	0	0	25	0	542	131	201	252	4952	182	635
215.31	9	0	10	21	548	94	229	313	4747	164	649
215.41	27	0	50	28	501	51	253	301	4377	77	553
215.5	36	30	52	0	468	56	291	344	4454	110	574
215.61	0	0	20	0	554	105	257	384	4397	189	602
215.7	17	4	42	0	502	78	209	314	4278	0	555
215.81	0	0	33	0	437	41	276	292	4388	110	563
215.9	16	0	42	0	469	54	233	375	4369	178	622
216.01	0	10	37	0	420	144	316	346	4040	0	631
216.11	15	15	53	0	511	104	288	337	4248	31	576
216.21	24	8	40	0	483	24	236	332	4169	276	660
216.31	8	0	49	0	497	71	277	356	4181	65	711
216.41	26	11	34	0	450	90	244	288	4101	151	739
216.51	16	11	16	0	493	131	219	289	4141	134	641
216.61	7	0	34	0	430	39	133	298	4291	260	712
216.71	0	20	31	0	516	132	242	284	4160	200	725
216.81	10	8	52	0	592	121	258	287	4262	307	774
216.91	19	10	38	0	521	47	144	170	4277	171	679
217.01	13	0	40	0	500	134	180	223	4178	99	677
217.11	6	0	26	50	525	93	258	121	4130	357	695
217.21	0	11	39	7	479	102	183	154	3952	339	606
217.3	7	20	49	0	427	93	283	241	3868	248	589
217.41	0	20	33	0	423	121	330	194	3721	134	597
217.51	11	0	23	0	403	55	271	202	3627	77	557
217.61	0	12	35	0	501	90	188	160	3688	313	544
217.71	16	8	15	0	375	82	213	232	3726	341	576
217.8	10	11	25	0	361	76	157	165	3943	165	583
217.91	0	0	52	0	449	115	210	158	3930	71	572

Scots pine #2. After extraction in alcohol.

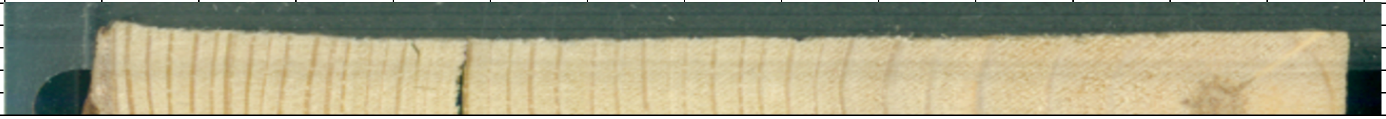

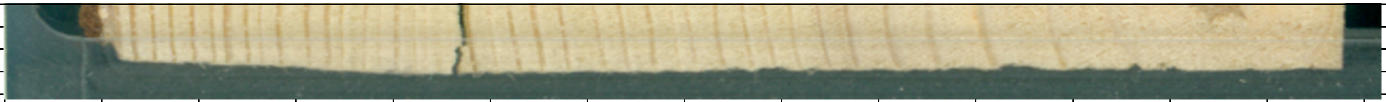

position (mm	Al	P	S	Ti	Mn	Fe	Cu	Zn	Ca	Sr	K
20.01	57	0	15	0	632	275	236	638	4109	163	782
20.11	77	10	25	10	456	413	246	630	2510	28	613
20.21	55	19	0	0	270	94	101	344	913	0	328
20.31	60	27	15	0	137	26	11	274	294	327	175
20.41	50	0	0	0	90	120	170	144	243	306	118
20.50	49	0	0	31	149	60	135	106	243	145	106
20.60	41	21	0	6	154	98	157	150	260	97	87
20.71	39	33	0	0	118	62	98	186	260	226	119
20.81	51	32	8	8	108	24	244	183	177	0	109
20.91	57	0	0	0	138	0	214	349	179	0	97
21.01	72	30	14	20	88	8	149	230	156	60	61
21.11	57	9	0	11	146	79	173	237	168	98	76
21.21	31	0	8	0	120	113	114	111	240	135	86
21.31	57	12	0	51	154	90	158	264	254	369	32
21.41	54	5	0	11	209	108	35	171	254	314	61
21.51	52	11	18	17	183	147	45	94	323	155	98
21.61	47	0	0	32	138	169	64	122	270	367	114
21.71	81	0	0	51	152	95	101	149	291	237	83
21.81	74	0	11	0	131	115	59	207	281	144	80
21.91	55	0	0	11	187	83	222	254	253	130	77
22.01	37	0	0	33	108	37	93	228	229	52	76
22.10	60	12	0	18	189	72	176	297	216	0	83
22.20	41	0	0	23	163	105	69	183	239	223	83
22.31	35	0	0	36	156	59	21	151	253	206	99
22.41	62	13	0	14	171	101	88	88	282	311	70
22.51	72	0	0	27	151	159	159	240	274	112	108
22.61	51	8	0	34	194	116	164	153	329	294	68
22.71	17	7	0	19	196	166	92	194	266	235	65
22.81	78	10	17	25	141	49	156	222	302	286	88
22.91	44	12	9	0	242	38	212	372	305	147	93
23.01	61	21	11	0	116	48	178	322	227	0	78
23.11	47	7	0	11	184	55	224	408	192	0	60
23.21	56	0	7	0	180	66	213	168	181	6	71
23.31	52	0	0	10	169	33	78	222	270	262	62
23.41	57	0	0	7	204	165	186	153	268	249	42
23.50	59	0	6	24	183	105	174	108	322	312	91
23.61	78	17	6	34	174	134	143	96	325	384	69
23.71	57	11	0	30	177	143	225	146	305	178	68
23.81	37	18	0	41	172	111	129	205	325	184	49
23.91	57	0	0	9	158	66	200	155	327	424	61
24.01	36	0	0	0	219	79	167	318	316	188	74
24.11	65	20	5	0	158	54	213	349	299	0	87
24.20	56	12	0	20	140	52	343	341	198	0	62
24.31	68	18	0	24	143	0	242	341	181	0	46
24.41	53	32	10	31	162	71	140	279	196	0	63
24.51	34	8	0	0	170	84	167	146	251	147	56
24.60	38	0	0	0	192	127	258	75	252	154	80
24.71	45	0	0	5	151	51	64	130	273	222	92
24.81	9	0	0	34	219	179	320	189	288	239	60
24.90	58	0	0	8	216	114	177	15	292	165	41
25.01	41	0	12	31	169	76	206	176	342	257	102

25.10	45	20	0	11	171	85	280	181	351	167	85								
25.20	53	6	13	46	158	47	170	400	309	0	98								
25.30	44	0	0	0	201	26	382	348	195	0	51								
25.40	51	17	0	0	160	58	222	295	200	89	54								
25.51	60	0	0	0	186	15	188	175	248	143	80								
25.60	43	0	0	9	175	146	195	232	280	86	37								
25.71	41	0	0	32	160	134	195	152	284	282	71								
25.81	31	0	0	12	145	125	131	105	288	107	54								
25.91	50	22	22	44	166	120	217	262	280	0	85								
26.00	54	10	10	0	206	13	188	251	233	0	56								
26.10	40	0	0	0	134	0	125	251	198	0	92								
26.21	39	20	6	0	186	27	132	320	134	0	43								
26.30	55	5	14	0	206	36	172	307	227	192	62								
26.41	52	15	6	37	145	17	169	204	219	112	42								
26.51	58	0	0	38	191	78	235	227	274	300	78								
26.61	63	0	0	12	145	98	127	231	340	253	59								
26.71	59	5	0	29	175	79	102	155	249	316	57								
26.81	38	0	0	25	190	95	198	295	318	182	55								
26.91	87	5	0	0	190	91	165	146	346	312	46								
27.00	44	26	12	0	209	104	176	379	313	244	88								
27.11	52	0	11	0	228	104	234	364	292	46	56								
27.21	64	48	5	0	217	14	188	199	230	0	83								
27.30	52	0	0	0	111	58	133	310	236	0	60								
27.40	49	0	0	0	163	50	227	90	216	43	83								
27.51	40	0	0	0	211	44	99	120	272	362	51								
27.61	43	0	0	0	174	109	108	190	308	293	47								
27.70	54	7	0	7	164	183	182	215	317	392	55								
27.80	69	0	0	36	202	122	264	122	337	388	60								
27.90	61	0	0	0	138	120	166	283	291	183	71								
28.00	54	23	34	39	154	64	236	322	286	0	79								
28.11	43	0	0	0	216	35	195	220	256	224	73								
28.21	67	11	0	11	142	87	163	256	283	188	46								
28.31	54	0	0	26	195	78	203	134	273	311	31								
28.41	30	21	0	33	182	103	204	108	280	149	84								
28.51	59	0	0	0	217	90	103	208	312	44	47								
28.61	53	0	0	41	170	124	137	54	277	281	112								
28.71	26	0	0	24	228	210	251	251	275	136	89								
28.80	67	21	0	5	167	69	219	239	346	82	59								
28.90	58	10	0	0	177	80	253	406	286	67	89								
29.00	55	0	21	0	156	22	386	157	236	74	81								
29.10	54	18	0	0	172	66	186	270	212	68	76								
29.21	66	36	0	13	153	31	227	289	267	0	51								
29.30	32	16	0	0	205	63	163	240	242	89	89								
29.41	58	0	0	22	191	92	113	202	263	107	78								
29.51	50	0	0	17	173	126	170	134	293	113	66								
29.61	27	0	0	32	156	171	107	59	285	315	45								
29.70	42	7	7	0	208	219	197	245	324	328	64								
29.81	42	0	0	0	195	144	108	118	303	275	53								
29.90	47	4	0	0	209	109	179	41	354	265	88								
30.01	14	9	9	0	222	180	130	304	373	219	65								
30.11	47	8	0	0	194	168	345	208	446	299	55								
30.20	64	0	0	40	189	61	228	199	340	134	70								
30.30	70	26	19	20	178	58	292	306	356	159	94								
30.41	51	9	6	12	210	0	127	374	270	0	86								
30.51	37	20	0	42	230	69	251	373	266	126	70								
30.61	50	0	0	14	166	8	186	430	241	0	77								
30.71	48	10	0	47	203	75	224	133	244	148	37								
30.81	44	29	18	0	138	95	162	215	356	33	90								

30.91	71	0	0	18	208	64	172	114	282	7	62								
31.01	56	0	0	23	139	94	197	190	335	277	46								
31.11	41	8	0	14	188	98	82	149	384	127	82								
31.21	57	20	0	11	167	165	155	236	346	217	83								
31.31	74	0	0	12	72	114	171	216	361	56	82								
31.41	34	0	11	29	160	104	176	375	325	0	58								
31.50	66	7	26	13	162	63	243	226	265	0	47								
31.61	62	9	0	60	155	35	285	364	222	0	68								
31.70	34	0	0	4	137	62	25	342	202	22	67								
31.80	57	0	0	0	165	70	202	120	308	185	75								
31.90	43	0	0	15	160	73	110	129	346	340	79								
32.01	54	10	0	30	174	98	106	237	315	333	64								
32.10	51	0	0	54	149	176	137	210	339	196	69								
32.20	48	46	0	25	187	58	156	212	318	0	68								
32.31	78	25	19	18	157	0	249	379	305	157	83								
32.40	53	0	0	0	115	44	177	220	257	55	83								
32.50	34	16	0	0	227	74	153	303	246	246	68								
32.61	46	10	0	0	155	125	217	234	220	188	60								
32.71	35	14	0	30	161	138	165	60	246	323	53								
32.81	20	8	0	0	146	130	164	203	286	150	48								
32.91	23	0	0	33	191	157	212	114	356	328	45								
33.01	69	0	0	0	146	195	96	89	296	190	59								
33.11	75	0	0	0	158	231	174	230	388	287	37								
33.21	65	16	0	10	200	145	78	237	369	181	61								
33.31	40	0	0	42	189	70	200	116	344	280	34								
33.41	47	0	0	0	245	124	231	29	354	301	76								
33.51	60	9	7	18	146	152	141	153	327	291	60								
33.61	57	0	0	0	198	130	246	399	296	26	69								
33.70	57	37	13	15	163	78	303	399	324	113	86								
33.81	39	0	0	22	159	55	233	276	217	39	39								
33.91	41	20	0	12	162	95	185	107	295	0	44								
34.01	58	0	0	0	192	138	173	224	326	185	78								
34.11	28	4	0	17	197	182	126	93	310	281	84								
34.21	28	0	0	35	177	246	120	217	337	142	73								
34.31	67	0	0	0	175	157	233	274	350	363	71								
34.41	51	8	24	23	254	163	172	323	323	0	50								
34.51	52	0	0	10	198	73	257	336	302	160	45								
34.61	65	25	12	0	166	70	147	205	340	191	54								
34.71	50	11	0	26	147	132	189	256	302	259	49								
34.81	44	0	0	0	182	38	154	178	317	135	69								
34.90	26	0	0	0	199	135	152	263	361	341	51								
35.00	37	0	0	13	206	162	133	201	325	255	42								
35.11	59	28	21	0	208	135	125	94	339	252	64								
35.21	41	9	22	17	186	123	168	302	338	79	61								
35.31	58	0	0	0	153	78	225	333	238	0	62								
35.40	45	15	0	23	206	59	189	256	217	0	54								
35.51	49	5	0	37	167	87	227	191	212	196	35								
35.61	50	23	12	38	191	48	148	217	226	188	72								
35.70	21	0	0	0	121	59	87	182	248	182	67								
35.80	20	0	0	25	196	149	177	141	309	282	67								
35.91	54	0	0	21	206	70	133	152	303	360	69								
36.01	22	0	0	0	201	86	5	170	345	403	40								
36.10	38	0	0	0	220	101	174	113	290	259	55								
36.20	56	23	24	33	219	82	211	195	326	216	53								
36.30	64	12	5	18	204	37	222	212	322	0	75								
36.41	38	0	0	0	211	123	249	298	258	51	47								
36.50	57	27	0	0	146	60	122	180	253	45	69								
36.61	61	0	17	14	165	97	132	280	290	183	51								

36.71	48	17	0	66	196	116	174	261	348	381	76								
36.81	57	26	0	0	174	35	138	187	345	87	66								
36.91	57	14	13	22	216	79	223	172	368	120	75								
37.01	53	13	0	46	155	39	134	179	370	119	60								
37.11	28	0	0	10	177	153	158	134	308	287	43								
37.21	32	0	0	6	176	115	75	159	374	168	81								
37.30	36	0	0	7	216	139	155	222	339	407	73								
37.41	47	0	0	0	211	149	236	207	282	236	67								
37.51	69	0	0	21	244	138	199	139	332	252	43								
37.60	42	0	0	26	319	149	252	147	402	50	91								
37.71	46	0	0	25	269	116	243	312	409	153	64								
37.80	36	14	0	0	216	71	172	290	429	24	99								
37.90	42	11	0	0	195	55	171	260	376	211	59								
38.01	51	0	0	11	250	143	138	360	358	157	67								
38.11	46	0	0	35	195	25	189	333	362	242	102								
38.21	36	0	0	0	248	83	212	274	360	33	84								
38.31	36	0	0	0	208	143	174	358	352	100	47								
38.40	51	0	0	53	243	217	155	143	403	344	61								
38.50	33	0	0	38	209	311	168	297	412	175	78								
38.60	53	0	0	26	218	497	185	53	431	273	58								
38.71	19	0	0	17	90	469	48	156	338	405	30								
38.80	5	0	0	0	124	258	0	18	153	549	0								
38.91	26	0	0	31	116	176	12	79	69	649	0								
39.01	33	0	0	0	202	217	136	229	307	227	56								
39.11	22	0	9	0	257	113	255	355	519	0	115								
39.20	37	21	11	19	191	47	244	469	390	0	94								
39.31	60	0	0	0	183	81	194	332	297	0	60								
39.41	56	0	9	24	198	77	246	194	246	27	83								
39.51	52	7	0	23	175	57	204	224	256	168	49								
39.61	53	9	0	0	182	111	117	206	297	243	70								
39.71	42	0	0	0	205	141	180	198	341	36	66								
39.80	43	0	0	0	159	52	121	132	341	252	51								
39.91	68	0	0	0	256	97	166	130	324	232	73								
40.00	42	0	8	23	192	149	183	223	365	341	58								
40.10	46	5	9	0	251	86	78	187	310	253	74								
40.20	43	8	14	0	213	83	159	232	387	195	78								
40.30	57	35	6	0	203	46	278	331	342	0	70								
40.41	35	8	0	21	251	31	306	300	282	24	59								
40.51	39	0	13	11	198	68	171	223	292	206	52								
40.61	44	0	0	0	158	50	133	110	340	260	37								
40.71	77	0	0	26	250	139	94	183	339	318	84								
40.81	54	0	15	44	261	159	123	286	349	233	66								
40.90	62	0	0	0	215	128	75	181	313	193	44								
41.00	39	19	0	68	203	127	275	310	383	392	64								
41.10	40	8	0	34	242	130	107	140	359	11	66								
41.21	27	25	0	0	217	87	143	284	361	317	76								
41.30	45	25	0	25	180	64	246	240	346	213	65								
41.41	56	8	6	6	261	34	130	320	315	0	53								
41.51	67	0	0	9	199	50	149	436	319	0	80								
41.60	41	10	0	40	210	26	188	255	309	27	81								
41.70	0	5	0	7	233	125	145	243	297	298	17								
41.81	49	0	0	45	203	43	162	192	329	43	41								
41.91	43	0	0	10	226	73	174	254	357	442	65								
42.01	48	0	0	20	268	23	188	126	355	239	43								
42.11	32	0	0	0	184	102	108	205	349	462	63								
42.21	52	9	0	16	227	124	177	266	325	238	78								
42.30	29	0	0	33	250	56	192	244	408	137	54								
42.40	38	8	0	7	275	44	180	190	386	192	41								

42.50	39	5	0	30	268	79	290	349	348	0	60								
42.61	76	24	26	0	288	63	372	446	300	0	76								
42.71	46	0	0	13	204	80	159	437	250	0	84								
42.81	33	0	0	17	207	63	198	385	284	0	52								
42.91	26	0	0	27	176	67	153	312	274	162	54								
43.00	49	0	0	0	203	66	150	122	320	242	55								
43.10	47	0	0	13	209	175	164	126	286	188	44								
43.21	51	0	0	0	285	98	157	156	361	528	60								
43.31	37	0	0	18	263	123	183	170	374	282	69								
43.40	31	13	0	23	200	127	46	122	392	296	77								
43.51	35	0	0	0	235	59	227	171	335	389	56								
43.60	42	0	0	0	268	87	169	225	355	181	29								
43.71	77	10	0	25	274	144	229	167	368	289	50								
43.80	69	0	0	14	323	127	226	231	359	33	58								
43.90	75	0	0	32	275	61	179	259	405	43	68								
44.00	25	0	0	7	274	62	175	383	355	152	100								
44.10	48	0	0	15	264	41	206	441	325	119	98								
44.20	48	0	0	6	203	64	221	365	364	0	70								
44.31	50	13	0	0	243	76	172	301	274	143	37								
44.41	64	0	0	0	247	60	267	220	274	177	68								
44.51	65	0	0	33	221	51	133	188	300	295	52								
44.61	62	7	16	6	220	105	190	152	325	186	73								
44.70	37	0	0	37	271	92	232	154	346	306	83								
44.81	35	0	0	0	204	121	197	133	332	312	60								
44.90	48	11	0	44	238	139	266	207	359	426	59								
45.00	37	13	10	8	270	116	77	147	369	416	49								
45.11	49	0	0	34	228	146	199	169	377	267	46								
45.21	45	0	11	0	243	110	226	193	384	299	74								
45.31	48	0	0	0	217	88	194	86	397	366	52								
45.41	44	12	0	7	267	167	215	208	428	191	52								
45.51	44	0	7	64	223	119	135	225	412	251	86								
45.61	28	0	0	11	262	104	127	181	383	90	86								
45.71	39	18	0	0	267	98	271	375	381	102	59								
45.80	46	10	5	0	289	115	217	315	397	15	88								
45.91	60	0	0	31	308	35	311	419	363	0	27								
46.01	65	26	0	27	281	47	270	374	275	0	63								
46.11	71	4	8	45	260	47	252	348	280	0	81								
46.21	53	0	0	10	276	112	193	224	274	0	45								
46.31	43	0	10	19	210	31	47	215	306	370	52								
46.40	47	0	0	0	282	128	109	201	347	318	66								
46.50	26	0	0	0	321	69	137	153	374	153	96								
46.61	44	0	0	18	301	82	102	169	417	289	66								
46.71	38	0	0	9	270	95	114	188	405	278	65								
46.81	39	12	0	5	243	76	123	205	415	252	47								
46.91	67	0	0	16	246	54	219	187	392	213	72								
47.01	59	0	0	0	282	84	245	171	393	0	75								
47.11	79	19	0	26	319	114	198	401	409	198	55								
47.20	54	5	0	10	338	53	253	351	337	38	70								
47.30	51	0	17	57	301	48	156	372	294	0	44								
47.40	28	16	9	0	269	31	216	453	287	0	65								
47.50	28	17	0	11	254	44	283	303	264	0	50								
47.61	53	0	0	0	236	89	121	213	263	140	52								
47.71	45	0	0	41	197	51	218	172	337	129	20								
47.81	51	0	8	16	269	98	174	239	377	316	70								
47.91	35	0	17	0	258	112	169	165	357	202	85								
48.01	52	0	7	0	252	126	188	178	314	293	50								
48.10	53	0	0	14	243	138	214	149	352	160	61								
48.20	39	19	14	0	270	66	178	192	352	345	61								

48.30	74	18	19	18	295	52	193	165	373	190	74								
48.41	60	8	0	12	259	47	222	61	405	203	68								
48.51	60	4	12	0	306	133	229	282	374	205	86								
48.61	25	0	9	36	224	139	88	159	370	101	79								
48.71	61	8	0	29	283	97	272	267	307	40	91								
48.81	47	13	11	0	243	35	204	330	318	0	60								
48.91	75	0	0	44	215	44	153	250	291	64	87								
49.01	49	21	0	49	224	40	240	299	260	18	70								
49.11	39	8	0	67	216	86	235	333	260	41	58								
49.21	61	24	13	0	270	96	224	231	313	136	77								
49.31	60	11	0	0	307	105	66	145	352	319	62								
49.41	46	0	0	10	261	111	173	139	316	212	36								
49.51	45	10	0	10	277	123	274	157	344	269	54								
49.60	55	9	0	0	305	130	164	292	415	220	52								
49.71	32	0	23	0	313	103	167	219	403	213	40								
49.80	64	0	0	20	277	123	143	242	398	333	59								
49.90	75	0	0	0	271	108	227	227	370	220	45								
50.01	58	20	0	0	301	56	123	316	462	299	89								
50.11	58	0	21	0	331	15	209	342	431	0	91								
50.20	49	10	0	16	282	57	231	410	411	47	47								
50.30	56	21	22	0	323	62	189	225	373	36	59								
50.41	59	16	0	31	362	71	174	213	353	88	37								
50.50	48	27	0	20	295	101	153	181	371	209	89								
50.61	66	10	0	0	306	50	89	186	348	107	100								
50.70	43	13	0	19	312	47	200	264	383	205	70								
50.81	52	23	12	0	330	136	230	187	407	199	42								
50.91	49	0	25	47	321	57	235	373	385	0	70								
51.01	60	0	0	10	327	63	314	367	324	0	87								
51.11	55	0	20	17	240	79	239	374	298	29	89								
51.21	46	13	0	30	227	61	113	292	299	84	50								
51.31	34	0	0	52	279	85	177	234	308	255	67								
51.40	51	0	0	5	303	81	166	175	351	242	62								
51.51	24	6	0	28	260	104	275	331	358	42	82								
51.60	10	0	0	47	249	101	160	159	379	189	41								
51.71	35	0	0	19	241	122	169	217	389	455	62								
51.81	41	0	13	40	281	121	208	198	393	166	89								
51.91	40	0	0	7	273	101	254	255	373	353	89								
52.00	27	0	0	15	251	146	119	234	406	314	85								
52.10	36	0	0	0	336	117	184	220	440	316	39								
52.21	49	0	6	0	260	47	69	102	336	168	56								
52.30	76	5	15	0	295	82	166	197	421	132	76								
52.40	64	26	6	42	322	24	67	192	372	0	100								
52.51	57	5	22	23	342	51	229	312	386	0	103								
52.61	62	29	4	42	348	31	304	303	338	0	86								
52.71	62	7	0	20	288	21	159	330	285	35	50								
52.80	55	26	20	0	270	51	192	156	283	0	92								
52.91	62	0	0	21	311	62	189	234	332	159	60								
53.01	43	0	21	40	271	57	169	169	301	204	70								
53.11	42	0	0	6	292	53	188	133	335	265	77								
53.20	26	6	0	16	308	72	241	317	354	374	41								
53.31	45	6	21	0	293	101	150	298	399	91	80								
53.40	33	0	0	29	354	61	169	191	420	59	37								
53.50	49	0	0	22	298	21	157	207	392	287	34								
53.60	44	4	10	19	302	75	304	283	405	230	81								
53.71	24	16	0	23	317	99	117	200	416	120	70								
53.81	37	0	22	18	329	57	194	323	425	223	108								
53.90	37	16	9	0	393	44	245	318	395	0	82								
54.00	55	14	0	0	363	60	302	467	332	0	86								

54.11	68	11	0	29	258	61	325	322	330	0	56								
54.21	29	0	4	74	292	24	158	239	337	152	62								
54.31	51	9	0	0	320	72	189	240	322	138	68								
54.40	28	0	5	0	339	103	171	185	390	142	60								
54.51	30	0	0	0	347	145	265	312	404	360	55								
54.60	50	12	0	20	310	67	195	220	408	370	51								
54.71	64	8	0	0	297	71	206	200	385	179	49								
54.81	62	5	0	50	326	72	231	526	396	180	91								
54.91	55	23	0	7	334	71	165	764	434	0	70								
55.01	71	34	11	0	332	47	151	830	439	158	75								
55.11	26	9	0	0	340	93	335	625	359	0	70								
55.21	58	28	24	34	326	103	211	440	305	0	95								
55.31	32	0	0	0	323	105	284	341	318	170	46								
55.41	54	26	0	0	295	35	158	269	401	0	64								
55.50	26	9	5	0	309	70	257	263	418	306	93								
55.60	58	6	14	39	277	64	188	234	364	155	104								
55.70	50	14	0	34	302	52	109	164	389	434	67								
55.81	69	28	11	30	328	122	184	109	351	171	71								
55.90	77	15	0	0	310	101	325	207	414	107	75								
56.00	21	0	0	27	304	68	87	252	398	168	63								
56.10	43	12	0	7	286	88	276	221	432	340	98								
56.21	45	7	0	6	294	78	177	340	383	355	57								
56.31	37	5	0	0	381	107	295	241	399	211	91								
56.41	62	25	5	24	369	128	291	344	459	144	92								
56.51	40	19	17	34	296	38	253	291	393	27	76								
56.61	72	17	0	18	305	0	379	449	397	0	86								
56.70	63	6	26	5	279	0	300	382	264	0	91								
56.81	53	6	0	0	272	88	213	280	282	212	56								
56.90	61	5	9	25	250	44	203	290	329	204	61								
57.00	63	9	0	0	316	128	244	141	347	209	96								
57.11	48	21	0	0	250	61	183	101	359	304	79								
57.21	50	0	0	38	278	72	256	209	335	68	43								
57.31	62	0	0	17	316	93	156	225	369	66	65								
57.41	31	0	0	0	318	51	159	173	353	175	118								
57.51	18	0	0	48	291	105	230	257	387	298	87								
57.61	29	0	0	34	352	114	265	275	404	282	74								
57.71	58	0	0	70	340	133	192	272	421	196	72								
57.81	28	0	0	0	352	146	178	258	411	250	48								
57.91	45	0	0	0	329	83	217	273	448	121	92								
58.01	58	0	9	11	413	88	155	259	448	0	104								
58.11	54	0	22	0	352	35	382	395	432	0	86								
58.21	55	6	12	0	339	56	212	344	323	0	92								
58.31	31	5	0	10	315	36	233	276	353	160	72								
58.40	52	39	0	17	271	108	231	249	406	130	53								
58.51	74	0	16	9	258	62	197	208	369	67	60								
58.61	50	0	0	36	290	113	90	241	370	165	56								
58.71	26	0	0	13	274	78	214	263	406	339	42								
58.81	32	8	0	0	289	89	193	197	355	254	63								
58.91	42	16	0	0	299	108	132	216	400	259	60								
59.01	54	0	0	0	310	94	85	117	398	143	116								
59.11	12	0	0	9	396	74	163	220	428	151	67								
59.20	27	0	0	42	313	107	204	176	447	210	65								
59.30	38	14	0	34	309	103	195	279	468	98	69								
59.41	56	0	0	0	337	44	328	389	476	0	61								
59.50	64	26	15	0	344	34	345	532	418	0	75								
59.61	50	30	8	0	402	42	302	333	363	0	54								
59.71	53	0	0	6	406	59	270	329	375	0	85								
59.81	60	0	0	32	268	90	242	364	341	0	81								

59.91	54	0	0	12	370	39	228	301	351	0	52								
60.01	46	6	4	13	355	99	218	255	427	0	38								
60.11	60	9	0	0	348	32	277	294	382	199	67								
60.21	47	0	0	0	353	38	205	286	356	221	42								
60.30	47	0	7	21	278	57	133	321	385	104	41								
60.40	71	13	0	0	356	67	168	293	408	282	85								
60.51	23	0	0	0	358	89	258	176	456	465	52								
60.61	29	0	0	10	313	97	90	157	436	234	63								
60.71	42	7	0	21	366	75	223	215	418	278	69								
60.80	40	0	0	35	337	78	79	141	437	326	100								
60.90	36	0	0	0	312	51	193	184	462	279	53								
61.00	33	0	0	33	357	63	235	249	431	104	48								
61.10	56	5	0	9	366	21	172	108	431	339	62								
61.20	42	0	0	27	385	126	217	177	466	435	74								
61.31	33	0	0	0	374	53	77	202	452	311	63								
61.41	66	7	0	70	358	80	268	147	507	242	70								
61.50	24	0	0	0	368	97	193	285	491	156	44								
61.60	78	24	23	40	357	79	295	337	512	0	95								
61.71	75	0	0	0	410	0	259	532	465	144	81								
61.81	82	0	0	0	358	36	316	448	448	0	65								
61.91	77	21	17	0	391	45	226	443	393	0	50								
62.01	82	0	12	38	356	69	259	432	381	0	63								
62.11	80	32	11	0	379	44	215	266	363	213	63								
62.21	44	10	0	5	330	85	113	326	394	161	72								
62.31	45	20	0	12	331	66	174	313	388	0	58								
62.41	52	24	0	38	348	79	251	355	457	0	97								
62.50	53	0	0	15	351	52	130	384	467	149	52								
62.61	55	8	6	13	433	74	276	332	411	36	65								
62.71	70	17	0	25	319	65	140	206	550	31	82								
62.81	45	9	15	20	418	99	211	275	506	144	103								
62.91	86	0	0	0	361	0	190	264	502	149	58								
63.01	41	0	0	10	434	70	260	336	546	0	81								
63.10	59	15	7	19	399	14	162	299	505	160	32								
63.21	50	31	0	0	394	29	186	335	504	120	100								
63.31	37	10	0	12	415	59	232	296	494	71	74								
63.40	36	16	0	0	439	123	350	345	494	302	69								
63.51	6	17	0	0	377	95	227	152	493	98	73								
63.61	53	0	12	18	410	76	263	364	533	96	70								
63.71	66	34	0	19	389	78	339	170	575	196	81								
63.81	62	20	0	19	426	80	324	316	550	141	60								
63.91	43	9	0	0	465	60	209	358	575	156	108								
64.00	52	0	0	0	393	31	258	350	519	202	90								
64.10	44	27	19	30	448	52	369	346	524	0	53								
64.21	54	10	12	0	470	70	226	393	517	87	94								
64.31	71	16	36	16	401	0	273	381	455	63	93								
64.41	66	17	16	18	405	18	306	282	439	53	69								
64.51	83	28	26	28	363	42	159	299	423	242	65								
64.61	59	11	0	32	384	47	187	336	466	167	83								
64.70	40	8	15	0	375	125	241	356	508	237	61								
64.81	27	0	0	46	359	82	313	284	551	155	78								
64.90	28	0	0	8	396	117	162	278	524	115	83								
65.00	39	0	4	0	435	49	229	291	581	0	85								
65.11	68	0	0	17	387	92	144	366	563	64	89								
65.21	52	0	5	23	478	84	243	438	574	104	103								
65.31	63	0	0	20	421	93	346	342	511	250	85								
65.41	55	0	0	5	396	133	293	329	549	88	85								
65.51	61	19	14	39	488	114	210	334	635	105	115								
65.61	14	0	0	16	485	72	329	354	634	57	113								

65.70	48	13	7	14	434	41	294	271	660	0	132								
65.80	35	6	12	6	502	86	237	425	607	227	132								
65.91	51	12	0	0	535	81	249	438	699	88	144								
66.00	53	15	0	8	504	58	215	370	672	82	142								
66.10	50	0	0	0	549	27	356	273	685	0	124								
66.21	58	7	0	12	566	75	304	372	646	0	146								
66.30	61	0	0	16	575	84	330	361	753	55	151								
66.40	68	13	0	21	551	22	371	432	787	100	139								
66.51	55	51	28	0	690	50	271	378	1027	238	132								
66.61	48	9	0	0	701	48	286	605	1108	239	148								
66.71	58	13	21	49	625	86	276	462	1005	138	143								
66.81	43	0	0	6	498	82	310	457	756	61	120								
66.91	49	13	6	0	483	144	392	318	655	0	89								
67.01	25	27	0	0	386	56	305	399	506	247	69								
67.11	57	10	0	0	407	35	293	316	516	0	74								
67.20	66	0	0	32	379	30	250	350	527	159	59								
67.30	53	0	19	14	392	68	109	431	496	140	86								
67.40	61	9	0	15	368	127	216	212	517	370	86								
67.51	38	0	8	0	382	75	192	256	550	96	83								
67.61	33	0	0	0	383	126	150	208	555	220	104								
67.71	51	0	0	0	406	121	161	299	568	344	84								
67.81	61	8	0	0	404	117	179	209	590	270	82								
67.90	22	0	0	19	405	84	156	219	615	281	91								
68.00	49	0	0	0	363	112	193	194	522	174	92								
68.11	50	0	0	18	339	146	112	288	471	245	93								
68.21	45	0	12	13	403	153	228	199	525	311	84								
68.31	48	0	0	18	364	50	199	199	486	236	85								
68.40	42	0	0	24	346	98	105	179	525	140	77								
68.51	68	0	0	41	328	132	219	181	483	399	90								
68.61	62	24	0	0	356	118	187	251	592	330	91								
68.71	30	17	0	0	392	113	184	117	531	402	66								
68.81	55	0	0	22	353	93	136	136	570	355	97								
68.90	55	6	6	61	367	90	191	174	543	328	86								
69.00	21	0	0	24	366	171	124	190	587	281	81								
69.11	54	0	0	19	422	118	250	80	550	234	67								
69.21	37	0	0	7	401	80	150	203	552	440	101								
69.31	50	0	0	0	396	98	187	293	634	424	86								
69.41	54	0	0	26	451	139	175	243	595	226	91								
69.50	49	0	0	31	433	102	215	177	605	277	77								
69.61	32	0	13	37	441	65	159	262	648	253	113								
69.70	53	0	0	8	392	90	300	191	641	150	108								
69.80	57	0	0	0	462	145	177	191	635	182	114								
69.91	35	0	0	21	453	139	286	312	715	250	165								
70.01	56	6	6	0	458	110	170	290	758	219	129								
70.11	35	10	0	20	487	117	288	425	742	234	140								
70.21	60	12	0	0	486	83	269	371	721	128	100								
70.31	52	24	12	10	487	72	179	402	786	138	122								
70.41	74	5	0	13	572	26	121	300	808	0	126								
70.51	61	19	0	0	521	59	293	438	698	0	101								
70.61	65	13	0	64	455	47	286	486	559	40	94								
70.71	23	0	0	14	467	15	272	383	547	25	111								
70.81	39	10	0	0	413	43	227	302	514	271	73								
70.91	97	49	28	0	438	80	329	255	533	189	97								
71.01	34	0	23	23	381	42	205	283	517	147	92								
71.11	41	0	0	11	471	43	266	205	568	35	106								
71.21	63	10	0	35	412	92	108	190	568	220	92								
71.31	44	0	0	0	374	71	121	220	502	148	73								
71.41	60	6	0	0	397	84	243	241	567	189	107								

71.51	61	0	0	8	365	61	193	246	567	356	96								
71.61	26	0	0	30	406	145	242	204	593	258	89								
71.70	40	0	0	0	417	77	242	318	704	173	101								
71.80	81	17	28	26	427	131	256	347	725	169	73								
71.91	53	0	0	16	427	100	259	281	611	26	111								
72.01	55	10	0	35	435	56	239	399	614	299	112								
72.11	46	16	24	6	395	109	245	245	603	218	88								
72.21	57	13	0	39	424	52	174	249	602	150	106								
72.30	38	0	0	13	362	117	200	208	585	308	92								
72.41	36	0	0	31	425	92	225	187	565	50	97								
72.51	65	0	0	36	494	58	238	134	571	92	82								
72.61	65	0	9	12	410	64	265	160	563	412	107								
72.70	64	0	0	30	426	96	213	199	602	460	100								
72.80	36	7	7	27	400	207	131	255	624	421	118								
72.91	73	5	0	8	434	138	249	274	615	243	114								
73.01	33	6	0	0	470	43	185	271	637	407	100								
73.11	40	5	0	40	453	77	248	371	615	264	104								
73.21	57	0	0	8	439	80	255	214	679	214	120								
73.31	67	8	0	15	427	141	242	283	632	244	103								
73.40	48	0	0	12	500	131	130	211	656	118	95								
73.51	42	0	4	0	498	155	144	218	755	43	92								
73.61	43	0	0	26	465	88	224	312	777	53	145								
73.71	51	0	12	24	530	71	235	368	802	77	148								
73.81	76	16	0	0	511	82	362	408	764	49	115								
73.91	58	11	0	14	512	60	237	487	665	42	103								
74.01	54	23	0	13	482	73	291	393	581	136	102								
74.11	53	20	17	19	498	52	249	243	585	0	101								
74.21	51	0	0	27	423	26	269	282	526	214	93								
74.30	63	15	23	15	448	60	315	320	572	213	69								
74.41	16	0	0	0	433	148	298	357	589	0	87								
74.51	23	0	0	15	436	78	206	190	629	209	106								
74.60	42	0	12	34	434	75	234	294	724	249	97								
74.70	61	12	16	18	428	73	351	341	701	234	117								
74.81	60	17	5	0	511	125	231	234	639	206	92								
74.91	52	4	6	45	415	75	249	231	642	80	111								
75.00	22	11	0	59	459	70	346	290	634	86	108								
75.11	50	11	21	18	408	112	161	296	679	306	100								
75.21	31	5	15	0	425	108	343	300	649	282	86								
75.31	36	17	0	24	474	104	222	227	723	280	78								
75.41	67	0	0	24	412	123	293	296	687	361	112								
75.51	63	9	0	29	454	103	231	343	624	234	108								
75.61	26	20	16	10	467	95	219	303	642	193	96								
75.70	34	0	0	28	494	52	288	232	717	177	125								
75.80	52	0	0	40	528	98	158	339	715	244	115								
75.91	44	10	0	12	441	44	269	328	711	11	125								
76.00	60	5	0	0	519	81	302	322	682	0	152								
76.11	26	0	7	0	516	88	287	274	708	251	110								
76.20	68	0	0	13	484	67	231	319	735	293	162								
76.31	38	0	0	28	522	68	273	317	743	285	137								
76.41	26	0	6	5	548	38	245	302	801	116	117								
76.51	79	0	0	0	520	22	178	300	778	280	111								
76.61	34	0	0	28	563	82	283	241	804	21	106								
76.71	72	14	17	39	497	42	236	303	771	90	118								
76.81	50	0	0	21	450	96	260	255	767	104	130								
76.91	19	0	0	11	539	62	223	410	861	176	95								
77.01	63	15	0	10	543	26	371	299	835	531	129								
77.11	47	0	4	0	496	62	251	353	786	92	112								
77.21	41	5	0	0	546	54	229	246	852	104	129								

77.31	56	0	16	19	564	21	282	255	813	359	117								
77.41	41	11	0	0	500	69	254	299	884	0	135								
77.50	38	0	0	28	534	12	250	149	783	107	117								
77.60	42	7	0	0	460	7	195	394	796	203	112								
77.71	42	34	0	26	487	34	247	318	856	242	106								
77.81	66	35	0	0	569	54	210	325	790	145	121								
77.91	35	8	0	21	463	96	269	253	811	212	115								
78.01	63	0	0	31	565	79	246	365	842	230	105								
78.11	60	0	0	0	575	95	274	247	886	138	128								
78.20	31	0	0	25	511	125	216	332	914	203	114								
78.30	58	0	0	0	547	33	247	269	933	271	93								
78.40	43	7	0	23	562	123	260	283	898	209	138								
78.50	33	0	0	0	496	90	253	255	860	510	151								
78.61	49	0	0	21	548	57	179	294	892	306	110								
78.71	53	0	0	0	468	144	198	234	954	228	142								
78.81	55	0	0	31	495	150	210	244	875	223	141								
78.91	37	9	0	39	455	98	210	312	837	382	156								
79.00	33	0	8	17	442	67	141	259	954	337	116								
79.11	42	0	0	0	471	104	132	220	827	389	125								
79.21	38	5	7	6	501	155	190	349	861	465	151								
79.31	53	9	0	34	469	103	195	258	916	283	121								
79.40	32	0	0	0	439	55	156	254	929	200	149								
79.50	37	0	0	37	445	80	240	265	897	380	131								
79.60	8	0	0	26	455	58	183	250	882	191	144								
79.71	34	0	0	23	437	131	227	260	903	437	121								
79.81	48	0	0	9	449	120	175	245	891	286	141								
79.91	47	0	19	13	507	102	145	222	871	375	132								
80.01	53	0	0	14	465	144	302	290	931	167	136								
80.11	55	0	30	22	424	99	183	249	947	209	137								
80.21	39	0	0	6	521	63	261	313	893	245	128								
80.31	57	5	6	17	487	55	196	252	862	305	132								
80.41	44	0	0	15	511	81	153	324	944	250	120								
80.51	41	0	7	8	468	56	219	305	1012	436	133								
80.61	61	18	28	0	462	42	239	273	970	347	111								
80.71	16	15	0	9	524	125	248	346	891	302	124								
80.80	34	0	0	31	516	104	125	368	959	308	138								
80.90	80	0	0	30	523	99	222	290	974	342	153								
81.01	57	0	0	31	667	133	130	194	1071	237	134								
81.11	24	4	0	12	570	118	176	261	1194	370	157								
81.21	55	0	5	40	604	81	261	340	1147	399	135								
81.30	17	10	9	42	518	97	227	270	1104	38	124								
81.40	60	15	8	0	491	114	291	227	969	140	91								
81.51	49	0	7	24	501	80	252	435	861	364	127								
81.61	52	0	16	20	469	56	171	244	817	44	103								
81.71	62	9	7	21	418	129	188	316	824	228	115								
81.80	37	0	0	16	504	55	224	248	836	87	103								
81.91	17	0	0	0	525	80	270	254	771	448	100								
82.01	49	0	9	43	479	71	272	357	851	192	126								
82.11	53	20	16	19	527	118	311	257	847	273	119								
82.20	60	0	0	30	501	111	320	312	813	308	105								
82.30	54	9	5	33	494	12	261	310	809	406	119								
82.40	51	8	0	0	440	112	307	263	833	0	137								
82.51	41	0	0	42	539	152	262	426	855	337	107								
82.61	42	0	11	28	510	124	272	332	825	159	148								
82.70	59	0	0	0	492	83	204	295	744	265	106								
82.81	77	0	0	0	458	88	232	309	764	253	94								
82.91	33	7	0	0	453	69	266	354	754	38	124								
83.01	42	4	0	0	445	55	243	247	708	226	97								

83.11	47	0	0	21	435	91	192	198	690	33	129								
83.21	54	11	0	32	510	52	223	318	690	7	123								
83.30	52	0	0	26	465	141	192	250	690	285	144								
83.40	58	0	5	13	497	69	236	260	765	97	140								
83.51	41	0	7	30	519	134	243	333	772	380	155								
83.61	71	22	5	21	433	63	208	268	724	150	173								
83.71	52	22	0	0	364	124	141	294	796	270	134								
83.81	29	6	0	36	448	91	257	340	712	0	118								
83.91	49	0	9	0	427	140	200	260	727	68	108								
84.01	45	0	0	29	413	73	256	278	668	222	140								
84.11	42	0	0	0	414	61	193	278	674	186	96								
84.20	64	11	0	22	388	97	220	211	668	194	119								
84.30	49	19	7	50	458	106	316	311	690	360	119								
84.40	30	0	0	12	420	148	289	165	655	93	107								
84.51	53	15	9	0	413	131	209	198	657	248	74								
84.61	31	0	0	0	410	95	321	224	681	148	119								
84.71	49	0	0	0	384	40	202	360	687	315	99								
84.81	52	0	0	8	361	74	289	320	637	97	83								
84.91	44	0	0	29	450	95	261	330	746	268	130								
85.00	48	0	0	37	373	68	354	229	757	215	113								
85.11	48	0	0	5	381	106	217	293	818	237	99								
85.21	82	17	13	40	398	81	324	370	689	15	55								
85.31	73	0	0	22	411	149	281	259	652	33	91								
85.41	24	16	10	0	417	127	199	301	704	80	108								
85.50	65	9	19	30	425	110	336	324	659	268	118								
85.60	58	5	0	0	449	106	323	395	635	84	102								
85.71	46	11	0	16	431	72	348	298	680	58	118								
85.81	40	17	8	0	491	75	249	272	648	207	84								
85.91	49	14	7	5	407	48	374	271	706	189	135								
86.01	48	34	5	8	413	136	223	265	634	206	85								
86.11	50	0	0	0	469	83	249	141	617	134	100								
86.20	49	0	11	16	349	123	121	224	635	160	68								
86.30	50	0	0	68	347	96	282	338	631	242	87								
86.40	23	0	9	0	392	254	199	187	654	347	78								
86.51	42	7	0	0	323	422	212	139	690	232	101								
86.60	20	0	0	0	172	421	107	44	508	564	73								
86.70	7	0	0	22	143	220	74	0	223	496	29								
86.81	28	0	0	47	60	63	0	0	47	688	12								
86.90	23	0	0	5	49	102	65	0	23	680	0								
87.01	23	0	0	0	65	59	88	0	26	731	0								
87.11	36	0	0	45	74	69	103	21	28	528	0								
87.21	23	0	0	42	92	54	95	0	24	684	0								
87.31	23	0	0	37	59	88	117	86	37	658	0								
87.41	29	0	0	8	41	78	40	6	15	867	0								
87.51	27	0	0	19	37	72	22	0	13	576	0								
87.60	10	0	0	5	88	67	42	59	30	565	0								
87.70	13	0	0	14	62	67	66	0	52	541	0								
87.80	0	0	0	0	59	99	57	0	56	688	0								
87.91	21	0	0	25	84	89	66	26	57	760	0								
88.00	0	0	0	21	54	98	11	60	26	643	16								
88.10	16	0	0	0	55	78	0	15	32	622	0								
88.20	21	0	0	12	74	40	0	0	19	632	0								
88.31	13	0	0	19	64	94	73	61	41	656	0								
88.40	22	0	0	7	57	104	65	43	52	696	0								
88.50	17	0	0	33	35	27	60	32	15	611	0								
88.60	19	0	0	14	50	65	24	141	20	706	0								
88.71	25	0	0	34	86	78	58	34	58	643	18								
88.81	18	0	0	22	74	41	45	66	47	566	12								

88.90	21	0	0	13	103	61	0	84	46	416	15
89.01	7	0	0	17	81	59	75	26	38	806	15
89.11	4	0	0	0	58	60	6	0	59	550	6
89.21	23	0	0	12	72	82	41	25	37	689	6
89.31	0	0	0	0	74	121	90	21	36	431	0
89.41	10	0	0	0	59	59	57	63	22	468	28
89.50	7	0	0	25	72	93	8	158	41	564	0
89.60	0	0	0	28	69	71	38	144	59	689	0
89.70	6	0	0	0	63	76	69	114	22	680	0
89.81	23	0	0	15	68	67	37	113	45	755	0
89.90	0	0	0	0	49	66	36	15	41	716	13
90.01	7	0	0	18	59	58	41	54	27	541	0
90.10	51	0	0	19	45	51	24	47	41	575	4
90.21	26	0	0	8	80	80	43	69	33	630	0
90.31	35	0	0	0	44	88	25	31	32	760	8
90.40	21	0	0	7	81	58	0	0	36	446	0
90.51	45	0	0	0	78	48	0	20	34	712	0
90.61	8	0	0	9	95	100	33	109	37	646	9
90.70	34	0	0	20	43	61	0	44	54	667	13
90.80	23	0	0	26	71	89	46	0	39	574	15
90.91	23	0	0	39	55	41	0	0	105	731	0
91.00	54	0	0	14	92	63	71	6	976	694	72
91.11	49	0	0	0	117	93	197	124	2873	627	195
91.20	29	0	0	32	147	125	163	100	3589	477	330
91.30	60	0	0	26	144	151	299	132	2974	496	360
91.40	18	0	0	14	165	136	229	252	2461	355	479
91.50	41	0	0	0	181	117	242	117	2283	388	523
91.61	34	0	0	55	244	144	260	226	2214	400	649
91.71	39	0	0	0	245	76	192	264	2147	290	678
91.80	46	0	0	16	342	133	199	199	2065	521	603
91.91	42	0	0	33	146	284	255	256	2440	170	647
92.00	41	0	0	23	201	632	273	295	2370	176	613
92.10	52	20	0	22	229	206	357	272	2668	345	553
92.21	48	0	16	0	185	96	184	398	2432	23	547
92.31	87	5	0	10	154	87	212	272	2265	193	458
92.41	47	11	0	23	158	74	125	328	2098	318	509
92.51	52	0	0	18	230	89	188	234	2044	219	504
92.61	66	0	0	14	221	109	200	352	1881	222	552
92.71	46	0	0	18	219	108	357	405	1787	287	528
92.81	43	9	0	0	245	91	263	299	1920	273	532
92.91	36	0	0	22	341	123	236	360	2414	355	638
93.01	57	0	16	30	378	117	223	343	2899	469	724
93.11	28	0	0	26	434	134	340	513	3204	166	890
93.21	29	0	21	0	437	137	310	445	3169	32	792
93.31	49	40	12	10	347	128	265	461	2748	0	738
93.41	80	12	11	0	301	128	226	500	2222	57	541
93.50	73	0	0	4	316	92	335	347	1546	93	371
93.60	57	21	5	0	196	96	278	390	1040	0	272
93.71	57	0	0	0	140	82	198	416	579	45	213

Scots pine #2. After extraction in HCl.

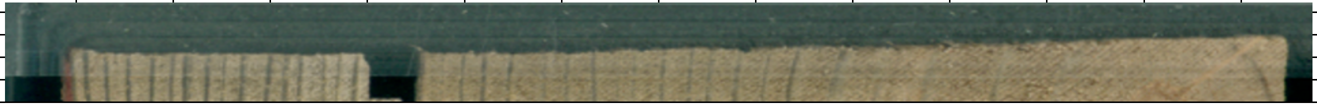

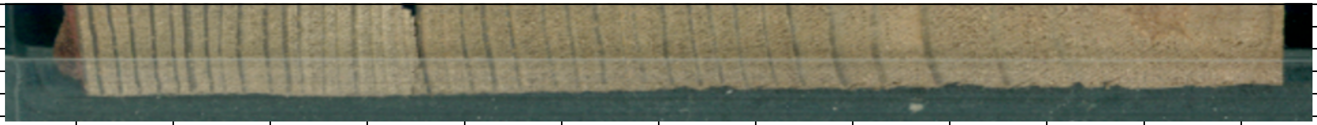

position (mm	Al	P	S	Ti	Mn	Fe	Cu	Zn	Ca	Sr	K
20.01	9	0	0	0	66	74	0	0	23	580	10
20.10	9	0	0	30	73	69	0	0	0	784	0
20.21	22	0	0	0	41	56	0	0	52	669	0
20.30	27	0	0	31	61	38	0	0	73	591	0
20.41	0	0	0	8	52	109	0	0	91	538	8
20.50	14	0	0	0	54	48	12	0	66	662	13
20.61	34	0	0	44	48	74	0	19	97	557	11
20.71	40	0	0	22	108	99	0	46	111	572	0
20.81	19	0	0	21	61	112	0	44	90	522	10
20.91	31	0	0	0	75	77	0	34	268	466	0
21.01	23	0	0	32	88	102	0	52	322	374	14
21.10	27	0	0	0	88	75	0	55	185	359	21
21.21	42	0	0	20	61	130	0	173	144	104	17
21.31	23	0	0	21	74	69	0	165	179	135	0
21.41	28	0	0	0	70	50	0	110	159	272	11
21.50	46	7	0	29	62	88	0	121	103	180	16
21.60	16	0	0	6	70	64	23	102	69	208	0
21.71	37	0	0	0	89	55	0	81	53	399	0
21.80	20	0	0	0	121	27	55	29	59	399	5
21.91	64	0	0	0	80	56	0	58	35	330	0
22.01	29	0	0	30	104	80	0	0	41	274	0
22.11	47	0	0	24	73	31	0	101	22	105	17
22.21	33	0	0	15	58	67	0	75	34	321	0
22.31	39	0	0	32	37	87	69	0	32	382	0
22.41	64	0	0	0	104	0	54	71	33	335	11
22.51	31	0	0	0	100	57	0	42	22	279	7
22.61	47	0	0	21	86	75	0	66	69	296	11
22.70	55	0	0	0	54	27	0	0	23	350	0
22.80	22	0	0	0	84	40	29	0	28	261	21
22.91	9	0	0	14	80	47	19	0	18	312	14
23.01	0	0	0	9	103	56	81	105	39	505	0
23.11	39	0	0	9	69	54	0	50	47	312	11
23.21	33	0	0	16	60	26	37	83	8	88	0
23.30	40	0	0	21	51	26	42	0	19	274	9
23.41	22	0	0	10	98	133	68	71	35	206	11
23.50	44	0	0	8	60	19	52	0	36	369	15
23.61	38	0	0	23	69	91	34	38	42	391	0
23.71	28	0	0	0	74	27	19	0	37	276	0
23.81	21	0	0	32	89	59	70	12	44	384	24
23.91	32	0	0	8	106	73	58	60	57	339	0
24.01	20	0	0	0	99	61	81	31	18	487	11
24.11	37	0	0	0	129	80	181	0	19	404	0
24.20	38	0	5	0	41	16	100	47	20	263	9
24.30	46	0	0	5	45	26	50	32	33	193	11
24.41	36	0	0	41	64	52	212	152	25	172	0
24.51	36	0	0	6	55	17	24	97	14	266	0
24.61	33	0	0	26	69	53	41	35	18	243	17
24.71	41	0	0	0	47	82	105	0	57	225	6
24.81	37	0	0	0	83	54	18	0	32	310	16
24.91	0	0	0	14	26	66	49	0	15	434	0

25.01	15	0	0	0	76	99	67	14	5	415	0								
25.11	24	0	0	0	56	81	47	46	18	197	10								
25.21	41	0	0	28	82	52	112	0	43	509	0								
25.30	23	13	0	0	83	69	55	80	30	240	0								
25.40	51	0	0	0	52	13	178	79	42	241	5								
25.50	26	0	0	0	81	25	61	58	27	177	0								
25.61	49	0	0	32	85	4	104	95	36	326	21								
25.70	48	0	0	0	81	0	16	88	38	174	16								
25.81	56	0	0	0	96	65	23	15	50	266	8								
25.91	26	0	0	11	81	27	66	15	31	438	0								
26.00	22	0	0	13	63	99	53	0	38	420	10								
26.11	19	0	0	21	64	38	56	37	34	320	4								
26.21	28	0	0	14	88	52	81	0	28	420	0								
26.31	38	0	0	0	74	32	119	14	44	362	26								
26.41	41	0	0	10	77	60	89	76	32	348	27								
26.50	30	0	0	28	80	0	41	112	17	194	14								
26.61	38	0	0	8	54	12	84	175	20	146	0								
26.70	40	0	0	0	87	33	70	118	17	271	0								
26.81	36	0	0	16	46	52	48	64	32	294	0								
26.91	60	0	0	24	72	63	36	27	16	540	0								
27.01	56	0	0	9	76	63	77	19	39	401	7								
27.11	32	0	0	0	57	59	0	47	28	266	0								
27.20	68	0	0	36	87	65	33	0	37	258	11								
27.30	52	0	0	0	71	17	52	159	44	146	0								
27.41	46	0	0	0	70	0	0	48	45	155	5								
27.50	54	0	0	32	89	55	27	99	21	135	0								
27.61	40	0	0	0	79	34	15	100	30	251	17								
27.70	17	0	0	0	85	13	28	12	30	0	0								
27.81	56	0	0	0	109	61	0	31	40	417	0								
27.90	48	0	0	23	50	59	73	0	23	347	0								
28.00	23	0	0	28	91	93	66	0	30	356	25								
28.11	32	0	0	10	100	91	0	0	40	359	0								
28.21	44	0	0	22	93	69	33	35	51	403	4								
28.31	8	0	0	6	93	58	116	0	15	413	0								
28.41	33	0	0	17	87	29	88	109	18	13	0								
28.51	29	0	0	27	132	0	0	157	31	92	33								
28.61	41	0	0	20	96	41	102	33	17	220	0								
28.71	24	0	0	19	70	49	0	57	43	327	12								
28.81	38	0	0	16	63	94	9	0	35	190	0								
28.90	38	0	0	0	101	53	85	106	37	492	15								
29.01	49	0	0	0	77	97	0	131	62	390	26								
29.11	47	0	0	0	99	104	140	8	41	383	22								
29.20	49	0	0	21	74	46	95	37	31	180	0								
29.31	55	0	0	20	75	74	67	49	47	341	23								
29.41	50	0	0	10	72	33	27	52	40	235	7								
29.51	34	0	0	0	72	49	0	7	31	311	0								
29.61	29	0	0	16	89	70	110	57	32	375	13								
29.71	15	0	0	0	90	79	130	0	31	497	9								
29.81	49	0	0	16	83	79	71	59	24	276	18								
29.90	31	0	0	0	69	55	0	0	27	306	0								
30.00	42	0	0	0	110	81	0	43	38	470	11								
30.10	29	0	0	29	86	47	0	103	26	331	0								
30.21	64	0	0	25	85	70	0	184	25	61	0								
30.31	43	0	0	0	110	33	56	37	40	130	0								
30.41	36	0	0	0	87	61	66	214	31	241	0								
30.51	41	0	0	16	84	21	15	112	28	290	14								
30.61	49	0	0	17	79	107	47	23	31	347	18								
30.71	49	0	0	0	90	86	73	0	30	416	0								

30.80	24	0	0	13	84	101	0	0	42	342	11								
30.91	21	0	0	43	123	95	23	0	24	190	22								
31.01	19	0	0	15	75	71	0	51	46	572	10								
31.11	22	0	0	0	96	58	0	0	32	519	0								
31.21	35	0	0	22	92	97	96	46	46	506	10								
31.31	26	0	0	27	99	66	0	43	34	305	25								
31.40	29	0	0	31	106	106	0	18	33	374	11								
31.50	19	0	0	23	69	23	66	99	30	152	7								
31.60	34	0	0	14	84	48	0	0	24	293	18								
31.70	40	0	6	9	93	28	0	94	25	198	19								
31.80	42	6	0	33	104	71	0	103	6	0	8								
31.90	12	0	0	32	66	73	63	29	30	208	0								
32.00	25	0	0	5	86	90	0	55	33	291	31								
32.11	46	0	0	7	97	77	0	0	32	274	4								
32.21	50	0	0	25	92	83	99	90	53	265	9								
32.31	28	0	0	6	45	49	5	0	33	245	23								
32.41	39	0	0	29	91	17	0	0	28	258	21								
32.50	29	0	0	0	106	64	16	99	31	195	6								
32.60	47	0	0	27	97	54	26	61	41	237	0								
32.71	55	0	0	0	71	29	88	116	0	312	17								
32.81	27	8	0	0	90	57	73	32	31	205	0								
32.91	54	0	0	0	121	42	0	49	11	342	17								
33.01	33	0	0	41	101	89	0	39	41	275	0								
33.11	31	0	0	0	58	65	17	6	63	252	9								
33.21	40	0	0	22	79	83	67	23	0	317	0								
33.31	34	0	0	46	42	125	0	39	9	254	0								
33.40	29	0	0	32	97	117	69	49	31	108	0								
33.51	27	0	0	19	61	43	43	37	31	239	31								
33.61	49	0	0	15	69	51	0	97	53	174	7								
33.71	45	0	0	8	73	42	0	0	30	194	0								
33.81	21	0	0	19	97	43	33	30	33	415	0								
33.91	39	0	0	8	96	79	35	0	29	526	0								
34.01	33	0	0	27	93	81	31	99	47	469	6								
34.11	44	0	0	44	91	59	7	65	27	431	5								
34.21	23	0	0	24	87	104	25	41	30	376	28								
34.31	25	0	0	0	103	110	73	32	11	533	0								
34.41	20	0	0	27	69	104	118	0	45	467	0								
34.51	29	0	0	16	95	97	22	0	32	395	5								
34.60	22	0	0	0	92	76	86	20	33	286	0								
34.70	68	0	0	0	77	15	0	67	46	305	12								
34.80	39	26	0	0	96	38	67	54	39	146	0								
34.91	35	0	0	21	58	38	83	26	33	211	9								
35.01	39	0	0	0	58	72	73	11	31	249	7								
35.11	54	0	0	26	54	112	0	67	39	292	0								
35.21	32	0	0	0	81	60	65	7	20	478	0								
35.30	46	0	0	12	98	77	0	0	42	351	0								
35.41	26	0	0	15	67	58	0	78	13	226	0								
35.50	30	0	0	6	43	31	43	47	27	139	18								
35.61	47	0	0	30	75	51	73	12	31	119	0								
35.71	53	0	0	0	75	101	120	49	38	181	17								
35.81	60	0	0	0	56	66	128	97	16	354	4								
35.90	27	0	0	0	107	66	20	0	37	408	5								
36.00	29	0	0	0	103	86	0	24	23	293	0								
36.11	59	0	0	19	80	65	120	6	54	356	8								
36.21	20	0	0	13	17	75	56	94	30	300	0								
36.30	41	0	0	8	44	29	13	40	18	449	11								
36.40	43	0	0	42	122	101	197	158	9	237	16								
36.51	55	12	0	0	63	25	0	109	40	125	14								

36.61	25	0	0	0	87	31	72	74	14	26	33								
36.70	29	0	0	19	82	107	47	0	37	117	22								
36.80	39	0	0	47	92	88	12	0	56	574	7								
36.91	29	0	0	6	88	89	5	40	55	457	0								
37.00	32	0	0	19	63	42	41	43	46	389	10								
37.10	28	0	0	0	69	100	101	0	33	287	15								
37.21	46	0	0	5	93	92	46	123	46	423	0								
37.31	19	0	0	0	59	59	98	7	46	369	4								
37.40	27	0	0	0	74	68	0	44	31	318	17								
37.51	30	0	0	34	86	86	76	0	63	350	0								
37.61	9	0	0	39	73	111	76	11	55	592	29								
37.70	0	0	0	23	82	138	28	0	19	346	0								
37.80	35	0	0	11	78	104	0	53	20	505	4								
37.91	20	0	0	24	78	57	29	62	9	357	0								
38.00	15	0	0	27	49	44	132	0	43	534	24								
38.11	34	0	0	25	72	71	7	13	36	432	23								
38.21	34	0	0	43	83	87	58	0	0	509	8								
38.31	23	0	0	26	47	73	0	0	36	554	0								
38.41	0	0	0	27	90	123	0	0	41	578	5								
38.51	24	0	0	0	65	91	20	9	46	570	0								
38.61	41	0	0	7	70	87	14	0	28	382	16								
38.70	38	0	0	0	82	92	31	0	33	217	0								
38.81	20	0	0	0	71	84	0	0	40	339	11								
38.91	34	0	0	0	50	88	44	25	27	161	0								
39.01	41	0	0	11	53	74	0	0	58	238	0								
39.11	24	0	0	12	84	56	90	0	19	535	4								
39.21	42	0	0	0	95	47	0	15	26	307	11								
39.31	0	0	0	31	69	79	13	18	34	391	9								
39.41	30	0	0	7	79	95	0	0	28	673	0								
39.51	0	0	0	20	75	128	57	30	41	268	14								
39.61	7	0	0	29	72	133	0	0	45	395	0								
39.70	5	0	0	5	62	133	48	0	38	454	4								
39.81	39	0	0	16	57	149	0	21	50	411	0								
39.90	11	0	0	32	67	115	32	58	37	393	18								
40.01	26	0	0	33	81	41	88	61	65	331	34								
40.11	45	0	0	6	62	69	80	205	68	173	0								
40.21	44	0	0	0	36	17	120	145	48	184	0								
40.31	24	0	0	0	113	91	18	95	48	218	17								
40.41	60	0	0	20	84	83	0	55	27	333	0								
40.51	39	0	0	17	82	67	29	62	38	307	0								
40.60	7	0	0	0	106	102	0	17	41	154	0								
40.71	30	0	0	0	67	24	0	71	16	289	0								
40.81	24	0	0	16	44	98	0	56	33	222	0								
40.90	17	0	0	11	71	53	27	7	40	219	6								
41.01	45	0	0	20	58	57	38	0	40	284	0								
41.11	65	0	0	48	73	44	0	61	38	323	0								
41.20	56	0	0	19	59	39	0	146	34	130	23								
41.31	37	0	0	28	101	49	88	42	28	151	5								
41.41	65	12	0	0	95	64	70	110	23	163	0								
41.50	31	0	0	9	87	63	96	0	50	258	0								
41.61	36	0	0	0	57	78	106	71	25	442	0								
41.71	21	0	0	19	73	96	0	0	38	308	0								
41.81	26	0	0	11	78	100	18	124	25	525	0								
41.91	8	0	0	24	80	100	83	54	35	399	23								
42.01	14	0	0	48	87	41	21	36	55	135	8								
42.11	12	0	0	62	82	75	78	0	49	496	15								
42.20	36	0	0	14	111	44	0	83	38	65	0								
42.31	67	4	0	0	85	86	20	127	28	10	15								

42.40	0	0	0	0	47	28	70	124	13	0	0								
42.50	29	0	0	7	74	67	45	166	48	259	0								
42.60	5	0	0	8	95	60	93	102	17	425	43								
42.70	25	0	0	17	80	93	110	48	27	477	11								
42.81	37	0	0	0	72	69	0	0	25	249	0								
42.91	12	0	0	0	58	57	0	0	49	197	9								
43.00	11	0	0	0	105	48	65	82	16	526	6								
43.10	17	0	0	10	91	65	76	17	86	393	0								
43.21	49	0	0	29	86	116	103	0	171	332	12								
43.30	29	5	0	0	102	90	103	81	142	228	0								
43.41	38	0	0	18	85	57	47	93	51	105	16								
43.50	27	0	0	0	78	31	0	188	34	15	0								
43.61	49	0	0	0	93	13	0	28	26	0	21								
43.71	45	0	0	0	81	25	99	67	38	265	0								
43.80	44	0	0	12	93	88	40	62	24	0	0								
43.90	62	0	0	20	59	83	122	31	43	442	7								
44.01	24	0	0	36	75	55	50	0	34	262	0								
44.10	17	0	0	0	92	36	31	0	35	445	0								
44.20	32	0	0	39	111	91	37	11	51	292	17								
44.31	26	0	0	23	56	130	67	0	44	210	4								
44.41	48	0	0	15	64	81	81	13	36	289	0								
44.51	29	0	0	12	86	119	68	77	17	400	0								
44.60	21	0	0	0	71	76	0	0	16	351	12								
44.70	25	0	0	6	84	98	0	0	37	249	7								
44.81	17	0	0	0	128	43	0	97	37	187	0								
44.90	42	0	0	46	89	65	141	134	27	223	13								
45.01	55	0	0	13	45	16	96	156	9	152	6								
45.10	47	0	0	0	59	69	151	107	36	237	0								
45.21	17	0	0	22	70	62	97	0	50	30	0								
45.31	10	0	0	15	63	28	24	0	40	468	12								
45.41	13	0	0	24	74	82	0	52	40	293	0								
45.51	39	0	0	6	109	66	14	0	20	204	0								
45.60	30	0	0	24	114	20	83	4	15	422	0								
45.71	36	4	0	0	114	74	90	51	49	333	12								
45.81	20	0	0	34	96	51	81	46	51	503	18								
45.91	10	0	0	26	109	69	77	0	34	363	18								
46.01	0	0	0	11	56	129	0	75	25	459	0								
46.11	12	0	0	25	70	47	71	0	36	314	0								
46.20	42	0	0	9	91	141	69	0	62	310	14								
46.31	50	0	0	29	91	94	89	56	34	137	6								
46.40	34	5	0	24	63	72	0	55	38	491	14								
46.51	54	0	0	17	85	64	128	108	50	258	18								
46.61	20	0	0	0	100	71	109	122	34	190	5								
46.71	59	6	0	0	111	77	58	148	48	80	0								
46.81	45	0	0	24	69	7	0	115	35	58	0								
46.90	71	19	0	20	69	62	104	50	21	193	5								
47.01	51	0	0	7	70	18	0	58	42	124	5								
47.10	38	0	0	19	87	59	96	17	6	486	0								
47.21	37	0	0	19	104	53	6	0	46	288	0								
47.31	32	0	0	13	90	57	19	12	23	283	5								
47.41	14	0	0	27	50	54	38	0	16	371	25								
47.51	32	0	0	8	47	56	0	78	47	151	17								
47.61	36	0	0	23	89	73	45	56	41	419	0								
47.70	20	0	0	10	89	75	0	0	30	357	5								
47.81	38	0	0	24	96	49	47	0	18	321	29								
47.90	53	0	0	16	69	24	38	57	43	295	20								
48.00	26	0	0	0	84	38	102	123	28	299	13								
48.11	42	0	0	13	121	8	52	82	50	124	0								

48.20	61	0	0	5	79	52	131	202	16	231	0								
48.31	52	0	0	18	63	69	20	44	36	276	0								
48.41	45	0	0	9	89	70	64	0	29	331	26								
48.51	54	0	0	17	100	42	0	109	36	315	27								
48.61	40	19	0	0	94	93	9	0	31	347	0								
48.71	38	0	0	0	70	114	103	0	43	332	16								
48.81	20	0	0	22	88	73	52	0	28	424	9								
48.91	37	0	0	38	74	68	33	64	31	194	0								
49.00	53	0	0	19	86	55	78	11	27	354	0								
49.11	36	0	0	33	73	49	89	0	32	275	11								
49.20	34	0	0	0	92	49	11	99	17	473	18								
49.31	31	0	0	40	80	83	49	0	15	472	11								
49.41	27	0	0	36	99	56	113	90	35	280	19								
49.51	54	14	0	30	93	41	36	78	33	280	10								
49.61	44	0	0	12	28	45	0	91	18	0	0								
49.71	32	0	0	13	65	35	84	70	46	129	16								
49.80	17	0	0	16	91	69	109	85	13	34	24								
49.90	37	0	0	6	37	34	78	24	21	194	0								
50.01	11	0	0	24	58	54	6	88	50	271	5								
50.10	27	0	0	25	60	72	0	25	18	161	15								
50.20	58	0	0	18	96	57	20	50	0	256	0								
50.31	34	0	0	43	75	65	26	15	44	119	22								
50.41	43	0	0	25	95	93	41	65	51	341	0								
50.50	31	0	0	5	80	46	0	71	21	393	0								
50.61	51	0	0	10	68	22	43	36	21	350	0								
50.70	38	0	0	0	81	113	74	37	36	223	8								
50.81	51	0	0	42	58	50	66	33	47	444	0								
50.91	56	0	0	0	101	13	0	45	63	253	15								
51.00	67	0	0	25	100	71	55	123	34	392	0								
51.11	0	0	0	7	83	57	117	116	42	175	15								
51.21	11	0	0	20	107	78	0	83	57	246	10								
51.31	26	0	0	0	69	63	124	31	52	355	27								
51.41	8	0	0	0	81	93	15	99	37	383	0								
51.51	34	0	0	0	58	70	7	17	60	435	15								
51.61	49	0	0	0	65	22	79	94	39	183	20								
51.71	56	4	0	11	67	62	104	172	34	186	0								
51.80	58	0	0	37	64	63	57	76	28	183	0								
51.91	38	0	0	0	61	54	0	175	19	195	0								
52.01	37	13	0	0	49	36	8	173	37	150	4								
52.11	26	0	0	21	83	76	34	60	34	423	5								
52.21	31	0	0	0	91	62	0	51	50	373	17								
52.31	17	0	0	0	102	60	0	39	29	224	0								
52.41	15	0	0	0	74	62	22	0	53	347	0								
52.51	50	0	0	7	115	48	35	0	29	391	0								
52.60	36	0	0	0	51	70	72	39	55	203	10								
52.71	35	0	0	10	49	79	83	41	51	221	0								
52.80	19	0	0	8	75	83	36	0	23	36	0								
52.90	25	0	5	0	112	81	73	0	20	261	19								
53.01	37	0	0	22	140	33	140	38	54	443	0								
53.10	42	0	0	0	90	0	63	34	64	329	19								
53.21	34	0	0	5	98	44	48	122	56	111	0								
53.31	37	11	0	40	56	28	0	94	44	29	25								
53.40	25	0	0	0	69	36	32	91	57	194	0								
53.51	13	0	0	25	51	63	53	60	11	438	8								
53.60	31	0	0	13	48	79	89	39	23	482	7								
53.70	27	0	0	27	81	21	0	15	39	312	7								
53.81	45	0	0	0	86	37	41	50	39	435	8								
53.90	28	0	0	0	91	60	103	79	44	494	7								

54.00	46	0	0	0	72	79	76	108	28	292	8								
54.10	34	0	0	32	83	54	35	68	43	303	0								
54.20	43	0	0	23	91	61	39	17	39	364	18								
54.31	32	0	0	0	126	58	75	0	36	314	9								
54.41	36	0	0	25	71	73	33	9	7	146	0								
54.50	16	0	0	0	46	37	90	97	50	334	0								
54.61	44	0	0	16	81	57	110	72	41	208	0								
54.70	51	0	4	48	117	49	75	275	42	115	11								
54.81	22	0	0	0	83	68	42	626	44	50	0								
54.91	41	0	0	0	82	23	38	712	35	30	0								
55.00	13	0	0	33	71	23	4	290	23	233	0								
55.10	24	0	0	21	90	87	0	0	42	424	0								
55.20	37	0	0	0	80	44	83	20	32	301	0								
55.31	48	0	0	30	70	26	0	20	26	336	7								
55.41	25	0	0	42	83	46	83	0	22	323	0								
55.50	28	0	0	38	63	71	5	124	21	277	13								
55.61	51	0	0	6	95	60	75	0	53	127	8								
55.71	42	0	4	0	47	34	58	89	42	104	6								
55.80	32	9	0	14	81	31	135	66	20	61	0								
55.91	40	0	0	19	84	75	51	122	37	215	16								
56.01	45	0	0	0	92	83	135	74	41	211	0								
56.11	36	0	0	0	91	13	37	92	22	221	0								
56.21	31	0	0	26	64	52	0	16	46	242	0								
56.30	40	0	0	0	59	77	78	107	17	411	6								
56.41	53	0	0	6	69	51	0	60	43	328	0								
56.51	34	0	0	16	62	90	112	0	51	361	20								
56.60	24	0	0	0	46	90	94	0	33	450	19								
56.71	38	0	0	0	77	86	12	0	32	290	0								
56.81	42	0	0	29	52	44	99	77	35	268	19								
56.90	50	0	0	30	107	55	87	4	48	295	17								
57.00	39	0	0	14	85	67	103	98	23	173	9								
57.11	6	0	0	19	66	76	152	166	41	120	0								
57.21	34	5	0	38	87	94	127	266	45	134	0								
57.31	63	0	0	33	85	62	119	52	33	0	0								
57.41	56	0	0	0	50	9	33	58	28	346	28								
57.51	61	0	0	30	33	75	12	82	48	58	14								
57.61	21	6	0	11	70	80	64	63	0	334	23								
57.70	37	0	0	0	86	36	39	81	22	282	0								
57.81	28	0	0	0	134	52	7	34	32	568	25								
57.91	47	5	0	0	94	66	104	65	41	325	0								
58.00	26	0	0	27	96	65	138	71	31	393	7								
58.11	27	0	0	24	68	78	82	110	29	197	11								
58.20	42	0	0	0	69	41	0	0	28	229	0								
58.31	48	0	0	15	75	68	69	61	22	216	18								
58.40	28	0	0	21	71	72	94	78	57	471	0								
58.50	36	0	0	51	68	80	86	0	58	265	14								
58.61	24	0	0	30	133	10	95	33	61	212	0								
58.71	32	0	0	7	101	78	0	115	37	67	6								
58.81	26	0	0	59	55	48	83	0	27	166	11								
58.91	48	20	0	0	86	48	45	24	71	374	0								
59.00	45	0	0	7	75	0	18	72	32	258	13								
59.11	32	0	0	0	85	56	13	53	64	256	11								
59.21	32	0	0	24	71	84	34	0	39	362	0								
59.30	41	0	0	0	74	70	0	40	36	308	10								
59.41	28	0	0	15	86	35	28	0	46	424	0								
59.50	58	0	0	37	49	101	45	11	43	383	0								
59.61	40	0	0	16	63	55	54	119	56	345	15								
59.71	20	0	0	0	117	84	77	31	33	280	20								

59.81	9	0	0	20	78	70	0	38	50	400	0								
59.91	34	0	0	0	79	27	90	10	46	124	0								
60.01	22	10	5	0	76	80	85	124	52	261	24								
60.11	36	0	0	6	79	69	143	145	43	105	26								
60.20	38	18	0	44	55	50	32	108	63	49	8								
60.31	28	0	0	0	59	12	0	96	49	128	7								
60.41	59	9	0	5	71	53	24	119	26	153	20								
60.50	32	0	0	19	69	72	96	158	0	242	0								
60.61	37	0	0	0	74	93	0	73	44	177	18								
60.70	41	0	0	0	104	11	17	59	36	103	7								
60.80	41	0	0	17	85	109	144	101	29	129	5								
60.91	44	0	0	0	71	82	60	62	36	249	0								
61.01	42	0	0	16	104	30	21	45	26	414	0								
61.11	22	0	0	0	86	92	132	146	21	341	0								
61.21	13	0	0	0	65	104	0	33	21	522	6								
61.30	38	0	0	17	61	86	37	118	39	466	0								
61.41	37	0	0	14	52	71	69	49	55	219	29								
61.51	37	0	0	25	91	122	55	53	68	221	19								
61.61	0	0	0	5	102	50	0	86	68	249	0								
61.71	25	0	0	0	115	38	81	98	29	254	15								
61.80	41	0	0	19	89	84	76	0	39	177	0								
61.91	58	0	0	15	91	33	46	93	49	294	0								
62.00	52	0	0	7	108	55	0	66	40	469	35								
62.11	35	0	0	33	106	57	49	150	48	140	0								
62.21	41	0	0	50	94	56	53	105	53	155	15								
62.30	56	6	0	24	68	22	173	148	43	98	7								
62.41	39	0	0	12	59	0	63	119	37	229	23								
62.51	28	0	0	25	94	13	60	185	48	98	26								
62.60	56	0	0	17	95	39	131	113	39	315	7								
62.71	51	0	0	41	120	22	71	135	38	144	11								
62.80	14	0	0	4	69	41	0	157	35	177	0								
62.91	35	0	0	6	78	26	79	141	49	0	23								
63.01	23	9	0	0	78	88	55	127	54	143	0								
63.11	77	0	0	0	70	24	68	55	70	341	16								
63.21	19	5	0	35	65	54	15	58	27	297	15								
63.30	53	0	0	43	63	59	67	137	27	470	0								
63.41	48	0	0	0	75	14	94	131	25	104	0								
63.51	44	22	0	0	84	29	70	37	43	310	22								
63.61	46	0	0	38	97	107	0	25	39	326	13								
63.71	40	0	0	0	52	69	0	69	45	357	9								
63.81	35	5	0	21	76	48	124	41	42	250	17								
63.90	41	0	0	24	61	65	120	14	44	219	8								
64.01	33	0	0	23	62	46	111	30	44	310	0								
64.11	44	0	0	25	109	66	35	107	16	334	9								
64.21	37	0	0	60	81	72	0	43	48	102	4								
64.31	53	7	0	15	41	63	0	23	47	282	19								
64.40	15	0	0	6	32	48	17	63	19	373	20								
64.50	40	0	0	25	70	102	85	188	54	308	37								
64.61	51	0	0	11	91	97	146	193	57	145	5								
64.70	39	0	0	15	48	52	34	247	38	108	0								
64.80	20	0	0	31	61	35	60	151	56	91	6								
64.91	31	0	0	6	75	48	185	242	51	189	15								
65.01	24	0	0	0	40	22	96	149	38	206	0								
65.10	39	0	0	35	77	56	44	17	39	287	23								
65.21	40	0	0	0	75	33	83	40	66	299	0								
65.30	34	0	0	18	91	184	115	162	42	340	10								
65.40	44	0	0	0	117	111	67	74	49	500	0								
65.51	53	0	0	13	88	80	10	78	62	112	17								

65.61	33	0	0	0	108	64	121	16	59	151	11								
65.71	48	6	0	27	80	70	26	79	65	283	0								
65.81	40	5	0	41	76	115	11	58	61	253	24								
65.90	22	0	0	20	66	73	0	55	41	111	0								
66.01	61	0	0	22	86	57	65	79	46	298	10								
66.10	27	12	0	32	99	57	23	8	37	294	0								
66.20	36	0	0	32	55	93	80	0	66	453	20								
66.31	43	0	0	6	56	0	54	106	71	348	5								
66.40	41	0	0	4	63	111	9	21	52	188	5								
66.50	29	0	0	0	49	114	35	43	76	231	16								
66.61	42	19	0	5	78	51	54	25	74	208	37								
66.70	55	0	0	21	53	54	110	0	45	341	0								
66.81	50	0	0	22	53	50	62	133	42	441	0								
66.91	64	0	0	24	76	68	81	175	63	221	6								
67.00	27	0	0	0	121	62	27	91	87	114	16								
67.11	34	8	0	11	62	76	52	113	34	367	31								
67.21	45	0	0	0	43	59	89	148	63	197	12								
67.31	64	0	0	21	55	27	50	143	66	185	5								
67.41	26	5	0	31	84	33	109	138	96	19	6								
67.50	50	5	0	17	90	54	95	169	87	109	11								
67.61	37	0	0	0	70	67	61	99	64	197	0								
67.71	57	0	0	24	62	108	8	134	84	146	13								
67.80	24	0	28	10	74	78	88	87	101	245	19								
67.91	30	11	0	0	50	78	0	103	75	176	0								
68.01	25	0	0	0	66	85	76	80	65	344	0								
68.11	56	0	0	33	108	84	66	73	54	141	20								
68.20	32	0	0	41	107	83	50	135	35	466	0								
68.30	25	0	0	34	67	63	0	44	43	238	0								
68.41	30	0	0	0	71	65	36	56	26	276	20								
68.51	23	0	0	11	86	106	35	65	74	382	14								
68.60	64	0	0	0	80	70	86	0	42	359	16								
68.71	30	0	0	0	91	104	13	66	58	181	7								
68.80	50	0	0	41	97	80	9	62	54	421	23								
68.91	28	0	0	0	74	69	104	0	60	322	34								
69.01	32	0	0	11	110	61	81	35	113	217	25								
69.10	48	0	0	25	65	110	57	31	64	324	13								
69.21	36	5	0	0	91	110	74	64	59	392	7								
69.30	9	0	0	0	58	98	90	48	56	409	0								
69.40	20	0	0	0	121	44	15	108	72	335	15								
69.50	36	0	0	0	65	114	9	87	67	453	12								
69.60	14	0	0	33	102	137	36	61	82	325	9								
69.71	30	0	0	29	75	167	64	188	87	448	20								
69.80	37	0	0	0	107	120	37	125	70	366	7								
69.91	49	0	0	0	74	106	65	53	34	368	0								
70.01	62	0	0	25	61	44	107	183	72	377	33								
70.11	52	0	0	18	85	110	85	157	66	256	40								
70.20	17	5	0	23	93	100	55	72	94	292	10								
70.30	32	0	0	21	89	61	66	79	63	207	25								
70.41	24	0	0	24	101	100	85	36	109	447	40								
70.51	25	0	0	0	64	109	123	51	69	460	0								
70.61	38	0	0	12	79	97	102	145	50	463	9								
70.71	50	0	0	28	58	41	141	91	54	358	13								
70.80	33	0	0	0	77	67	81	121	54	295	13								
70.91	43	9	0	14	78	86	140	168	83	116	18								
71.01	56	0	0	15	61	40	166	176	55	216	0								
71.10	31	0	0	0	23	51	53	116	45	198	9								
71.21	13	0	0	17	35	118	10	103	66	273	33								
71.30	21	0	0	29	70	58	42	79	47	312	18								

71.41	32	0	0	19	88	10	0	107	33	293	15								
71.51	38	0	0	18	83	62	50	82	40	278	0								
71.60	33	0	0	22	73	88	31	94	46	395	0								
71.71	17	0	0	0	57	107	92	149	58	201	12								
71.81	38	21	0	0	73	94	0	73	73	206	18								
71.90	62	0	0	0	58	64	0	105	41	275	0								
72.01	58	0	0	33	58	57	0	32	60	263	13								
72.10	50	0	0	28	46	64	27	0	35	327	17								
72.21	28	0	5	8	116	138	60	62	62	388	16								
72.30	29	0	0	0	108	63	143	25	78	526	38								
72.41	70	0	0	8	55	58	45	144	58	284	8								
72.51	35	0	0	21	83	77	20	138	41	75	21								
72.60	29	6	0	47	73	63	42	86	53	503	18								
72.71	51	0	0	6	139	67	17	39	46	278	11								
72.81	23	0	0	12	60	59	89	109	34	411	0								
72.91	0	0	0	20	87	120	82	24	55	371	0								
73.01	30	0	0	20	27	96	10	65	36	357	0								
73.11	79	0	0	0	76	49	0	0	49	521	14								
73.21	56	0	0	0	87	31	63	118	43	338	0								
73.30	41	0	0	5	71	76	18	51	44	388	0								
73.40	30	0	0	0	49	83	150	11	52	286	13								
73.51	33	0	0	12	110	59	69	114	42	344	13								
73.61	60	11	0	0	83	84	74	52	62	426	0								
73.70	15	0	0	9	28	70	63	137	65	420	0								
73.81	35	0	0	38	69	115	0	35	31	425	0								
73.90	33	0	0	0	42	76	38	6	82	359	27								
74.01	33	0	0	7	49	27	94	123	42	327	14								
74.11	39	0	0	21	71	60	33	126	37	215	7								
74.21	48	0	0	0	75	86	183	154	44	269	0								
74.31	34	0	0	0	61	60	136	88	41	217	9								
74.40	40	0	0	0	106	93	125	121	62	324	9								
74.50	43	0	0	16	104	53	139	178	29	145	0								
74.61	72	0	0	21	85	60	16	119	54	235	7								
74.71	68	0	0	0	74	11	167	53	25	223	0								
74.80	34	0	0	0	78	90	97	22	31	153	8								
74.91	47	0	0	0	80	41	110	5	47	256	0								
75.01	52	0	0	26	84	48	125	82	52	275	0								
75.10	45	0	0	30	38	48	45	41	44	350	21								
75.21	44	9	0	31	106	62	5	42	56	389	10								
75.30	36	0	0	58	61	50	119	131	40	298	0								
75.41	10	0	0	12	68	166	20	0	38	217	6								
75.51	44	0	0	0	52	112	64	89	39	212	8								
75.60	43	20	0	30	58	65	116	6	32	388	38								
75.71	31	0	0	37	88	79	116	121	48	357	19								
75.81	34	0	0	0	42	67	50	76	45	238	27								
75.91	32	0	0	0	116	72	34	40	28	372	5								
76.01	22	0	0	0	46	78	80	0	41	267	11								
76.10	29	0	0	0	70	65	0	121	60	194	10								
76.21	32	0	0	28	59	65	62	73	55	160	0								
76.30	42	0	0	0	54	71	178	17	54	326	0								
76.40	42	0	0	45	91	29	129	91	59	305	0								
76.51	51	0	0	42	62	72	118	72	48	244	0								
76.60	49	0	0	35	51	121	194	53	40	132	0								
76.71	16	0	0	0	66	107	131	58	63	227	0								
76.81	20	0	0	0	87	62	164	104	78	259	25								
76.90	24	0	0	21	65	98	103	84	48	338	0								
77.01	41	0	0	0	109	87	0	18	48	243	15								
77.11	47	0	0	43	66	141	14	114	15	318	11								

77.21	30	8	0	0	76	39	97	0	47	142	0								
77.31	34	0	0	0	75	76	122	81	40	319	0								
77.41	50	0	0	5	84	76	119	0	55	470	0								
77.51	47	0	0	18	82	67	81	0	43	356	5								
77.61	28	0	0	46	79	62	60	38	46	509	0								
77.70	48	0	0	49	75	81	108	85	56	304	31								
77.81	64	0	0	0	93	84	106	191	40	382	8								
77.91	48	0	0	15	66	113	124	136	75	336	0								
78.01	41	0	0	5	54	80	107	49	48	417	0								
78.11	33	0	0	0	41	92	84	117	70	298	7								
78.21	49	0	0	0	116	63	85	180	60	251	21								
78.31	51	0	0	57	38	79	83	140	39	460	0								
78.41	40	0	0	10	89	58	94	0	64	235	11								
78.51	5	0	0	0	67	110	50	109	41	286	6								
78.61	37	0	0	0	110	56	82	23	40	442	15								
78.71	0	0	0	24	87	63	6	0	69	336	8								
78.81	44	0	5	18	89	65	115	49	47	307	34								
78.91	46	0	0	25	44	100	42	27	48	416	21								
79.00	9	0	0	47	52	77	160	41	50	374	19								
79.11	48	0	0	0	73	127	28	95	76	390	0								
79.20	27	0	0	38	83	112	54	9	63	438	0								
79.31	12	0	0	19	33	80	68	59	72	415	0								
79.41	47	0	0	61	82	111	102	63	48	180	0								
79.50	40	0	0	6	104	66	54	53	50	292	14								
79.61	18	0	0	47	103	89	13	68	48	343	22								
79.71	39	0	0	0	69	47	69	0	61	91	11								
79.81	18	0	0	0	107	95	50	0	52	368	5								
79.91	46	0	0	38	75	115	18	47	44	464	13								
80.01	30	0	0	0	60	83	71	61	59	349	37								
80.11	48	0	0	0	9	16	149	30	49	319	0								
80.21	36	0	0	27	59	78	0	95	49	363	0								
80.31	39	0	0	5	60	61	28	0	49	366	14								
80.40	45	0	0	46	51	50	129	11	57	333	0								
80.50	34	0	0	0	86	62	76	43	84	438	13								
80.61	34	0	0	14	82	86	154	0	49	276	11								
80.70	36	0	0	47	98	81	108	109	51	519	8								
80.81	17	0	0	28	67	39	118	102	57	480	17								
80.91	47	0	0	8	33	35	58	60	49	387	6								
81.00	31	0	0	35	119	80	46	162	41	247	0								
81.11	42	0	0	14	67	31	51	0	40	195	19								
81.21	48	0	0	0	64	106	169	28	52	367	14								
81.31	38	0	0	25	78	82	43	85	95	407	6								
81.40	65	0	11	0	58	72	57	12	45	231	18								
81.50	15	0	0	11	69	75	58	68	31	278	0								
81.60	45	0	0	31	77	32	74	101	57	399	13								
81.71	36	0	0	45	43	78	97	110	58	180	12								
81.80	15	0	6	8	89	85	115	36	51	334	20								
81.90	57	0	0	15	65	98	95	26	35	403	5								
82.00	26	0	0	20	49	109	71	114	77	200	11								
82.11	42	0	0	41	102	95	71	61	26	374	23								
82.21	61	0	0	5	35	18	85	26	53	336	21								
82.31	60	0	0	11	78	52	45	63	75	124	0								
82.40	25	0	0	0	82	56	71	60	61	194	0								
82.51	45	0	0	0	81	69	92	134	45	159	0								
82.61	22	0	0	21	78	127	0	25	49	207	0								
82.70	49	0	0	7	61	79	133	146	53	226	5								
82.81	38	0	0	10	67	131	55	34	57	414	45								
82.91	35	0	0	42	16	96	155	0	48	230	7								

83.01	45	0	0	44	46	48	147	95	18	246	0								
83.10	34	0	0	43	66	91	111	92	47	294	0								
83.20	30	0	0	38	75	45	85	92	43	173	7								
83.31	15	0	0	0	76	35	59	63	50	264	0								
83.41	22	0	0	8	68	102	120	39	35	469	0								
83.50	41	0	0	19	54	54	74	121	35	242	11								
83.61	64	0	0	12	126	70	69	70	45	272	0								
83.71	52	0	0	42	103	79	132	90	81	119	25								
83.81	37	0	0	0	80	31	0	76	48	297	5								
83.91	26	0	0	0	86	111	119	69	15	365	8								
84.01	46	0	0	0	82	69	78	30	40	375	8								
84.11	37	8	0	36	101	78	131	72	39	228	0								
84.20	22	0	0	15	97	82	191	74	31	280	14								
84.31	30	0	0	17	93	63	0	81	34	353	8								
84.41	15	0	0	31	82	77	219	11	58	358	10								
84.51	28	0	0	0	111	114	189	126	36	329	0								
84.61	32	0	4	0	93	102	177	31	42	339	0								
84.71	53	0	0	0	69	54	143	96	41	344	0								
84.81	19	0	0	6	68	111	11	20	41	378	0								
84.91	30	0	0	6	81	64	59	71	53	323	18								
85.00	33	0	0	38	115	89	64	54	53	262	28								
85.11	6	0	0	36	58	96	73	63	73	204	9								
85.21	24	0	0	13	84	28	110	79	54	229	10								
85.31	63	0	0	29	107	39	110	58	24	210	19								
85.41	34	7	0	10	50	59	71	34	44	139	0								
85.51	41	0	0	0	77	123	137	49	62	260	28								
85.60	42	0	0	7	63	30	59	65	64	100	20								
85.71	44	0	0	24	123	64	188	89	61	245	25								
85.80	52	0	0	15	44	81	126	126	54	237	19								
85.91	36	0	0	0	76	79	164	61	35	283	4								
86.01	26	0	0	0	53	87	109	179	38	278	0								
86.11	15	0	0	42	61	51	66	77	70	225	0								
86.21	16	0	0	0	61	91	40	92	50	287	0								
86.31	54	0	0	0	61	78	131	0	56	236	7								
86.40	27	0	0	31	137	89	35	59	34	413	15								
86.51	31	0	0	9	70	94	106	82	75	497	16								
86.61	39	0	0	19	94	106	61	107	47	230	0								
86.71	46	0	0	19	107	201	109	97	114	253	0								
86.81	0	0	0	16	82	137	72	41	81	655	5								
86.90	16	0	0	21	65	97	66	0	0	623	0								
87.00	9	0	0	30	67	74	0	0	9	723	6								
87.11	12	0	0	22	93	73	81	17	56	633	0								
87.21	7	0	0	37	84	49	46	0	19	722	0								
87.30	15	0	0	87	42	79	0	60	40	640	5								
87.40	14	0	0	5	51	61	46	0	27	670	0								
87.51	0	0	0	7	62	51	0	22	26	600	0								
87.61	21	0	0	40	85	85	53	0	29	691	5								
87.71	0	0	0	7	70	92	0	0	57	739	0								
87.81	14	0	0	29	69	96	0	50	20	576	12								
87.91	0	0	0	17	54	90	53	0	35	724	0								
88.01	4	0	0	6	64	38	0	0	34	538	35								
88.11	36	0	0	0	47	76	24	0	41	668	0								
88.21	15	0	0	21	54	97	91	15	24	768	0								
88.31	0	0	0	37	57	56	0	5	27	557	0								
88.40	18	0	0	31	69	76	15	0	38	684	0								
88.51	38	0	0	28	74	71	86	98	31	487	0								
88.61	15	0	0	5	61	75	62	0	43	621	0								
88.70	15	0	0	17	76	69	0	28	29	619	22								

88.81	36	0	0	26	44	33	0	0	50	567	36								
88.90	5	0	0	9	67	34	0	0	15	671	0								
89.01	21	0	0	0	62	72	0	29	11	661	0								
89.11	0	0	0	0	54	23	40	45	64	533	0								
89.20	13	0	0	51	68	75	41	43	45	717	0								
89.31	0	0	0	0	96	60	54	81	109	415	14								
89.40	37	0	0	36	54	74	71	0	948	525	0								
89.50	8	0	0	0	62	132	19	0	1442	478	16								
89.61	0	0	0	0	90	94	18	126	1430	465	20								
89.71	15	0	0	0	51	121	46	79	1158	453	33								
89.81	34	0	0	22	89	99	28	61	697	358	6								
89.91	35	0	0	0	78	117	171	0	556	316	0								
90.00	25	0	0	13	86	89	10	5	318	354	14								
90.11	27	0	0	49	84	64	84	126	180	489	13								
90.20	15	0	0	17	60	89	0	135	175	239	14								
90.31	42	0	0	13	62	243	0	82	352	288	12								
90.41	36	0	0	17	60	191	49	53	480	373	11								
90.50	44	0	0	5	68	91	0	21	519	257	0								
90.61	18	0	0	0	52	65	90	156	478	162	19								
90.71	24	0	0	0	85	116	132	57	396	245	0								
90.80	42	0	0	0	64	84	157	103	316	439	28								
90.91	15	0	0	22	67	90	38	81	257	315	26								
91.00	55	0	0	18	73	86	16	32	312	389	0								
91.10	42	0	0	33	83	97	47	83	388	315	12								
91.21	55	0	0	16	59	154	54	88	498	204	5								
91.30	31	0	9	28	78	90	72	180	467	208	21								
91.41	51	0	5	11	24	46	147	167	400	130	11								
91.51	43	7	0	39	50	98	104	142	381	115	7								
91.60	32	0	11	0	47	108	111	167	352	50	0								
91.71	49	0	0	16	63	86	154	146	185	29	0								
91.81	26	9	0	30	4	84	93	132	82	7	13								
