# Supplementary material for: Dendrochemical Challenge in Climate Science: Whether Chemical Elements in Wood Reflect the Fluctuations in Weather Parameters
Source: Plants (Basel). 2022 Nov 25;11(23):3240. doi: 10.3390/plants11233240 (PMC9740880; doi:10.3390/plants11233240)
Supplement: Supplementary file 1 [file plants-11-03240-s001.zip › Table S27.pdf]

Table S27. Scots pine #3. Initial core. After alcohol extraction. After HCl extraction.

Colour	Meaning
green	inner border of the bark
blue	pith

Scots pine #3. Initial core.

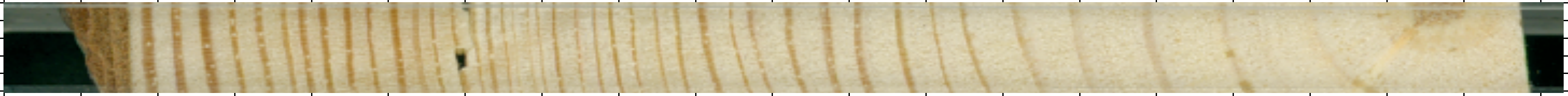

position (mm	Al	P	S	Ti	Mn	Fe	Cu	Zn	Ca	Sr	K
18	17	0	0	11	84	78	0	0	38	1163	21
18.1	5	13	9	0	91	100	0	0	42	1055	33
18.21	8	7	26	7	85	67	0	0	58	1006	32
18.3	4	0	26	0	97	115	0	0	62	1179	21
18.4	9	4	13	0	69	89	0	0	37	1117	17
18.5	6	0	6	0	60	97	0	0	66	1084	48
18.61	0	0	0	0	77	91	0	0	70	848	63
18.7	9	6	22	0	73	100	0	0	77	972	34
18.8	18	13	21	7	75	89	0	0	52	936	24
18.91	0	0	18	0	78	97	0	4	75	1086	38
19	7	0	24	0	58	95	0	0	69	1247	21
19.11	0	0	0	0	93	49	0	0	82	867	13
19.21	20	17	37	0	85	82	0	0	75	956	28
19.31	23	10	8	0	84	110	0	29	45	891	31
19.41	9	5	14	0	74	60	0	41	59	859	12
19.5	0	0	17	0	75	118	0	7	57	1037	46
19.61	21	17	25	0	77	74	0	0	61	1025	61
19.7	27	15	28	0	90	113	0	0	60	890	49
19.81	10	0	0	0	62	76	0	0	85	1007	17
19.91	4	0	8	0	93	96	0	0	58	976	15
20	0	16	24	0	58	110	0	0	99	950	12
20.11	7	0	29	13	60	95	0	44	87	992	30
20.21	0	9	7	21	94	102	0	0	56	1048	18
20.3	0	0	0	0	75	78	0	0	37	963	15
20.41	0	0	5	0	83	66	0	24	45	1075	15
20.51	0	0	9	0	83	105	0	0	44	941	11
20.61	5	0	0	0	106	92	0	0	25	982	9
20.71	17	10	18	0	74	82	0	19	73	1043	32
20.81	0	19	7	4	69	88	0	0	63	979	33
20.91	0	4	16	0	84	92	0	49	29	982	13
21	0	0	29	11	89	86	0	0	75	879	33
21.11	5	0	15	0	99	94	0	73	33	992	5
21.21	8	0	6	0	80	110	0	0	21	964	5
21.31	0	0	5	0	72	89	0	0	28	1004	8
21.41	16	4	0	7	82	80	0	0	44	841	0
21.5	20	0	12	0	113	111	0	0	22	787	13
21.61	14	8	20	0	79	75	0	0	29	1081	0
21.7	0	0	19	0	72	101	0	0	59	1090	33
21.81	14	0	15	0	91	71	0	0	59	1057	20
21.91	0	5	0	0	62	96	0	8	47	1036	17
22	0	0	0	0	78	94	0	0	60	1075	46
22.11	0	14	0	0	96	80	0	0	216	991	52
22.2	8	0	22	0	99	114	0	0	505	1061	80
22.31	16	9	23	0	71	97	0	0	691	867	102
22.41	17	13	27	22	62	172	0	7	1074	960	112
22.51	0	0	44	16	102	187	0	0	1484	920	126
22.61	12	7	22	25	105	143	0	0	1471	816	66
22.71	15	21	42	0	91	140	0	0	1106	819	131
22.81	12	0	16	0	79	122	0	0	1650	696	144

22.91	13	20	40	18	99	194	0	0	3692	908	178										
23.01	28	12	47	0	86	126	0	0	4675	792	206										
23.1	24	27	48	0	121	180	25	33	4247	660	197										
23.21	0	19	45	10	108	155	0	0	4325	724	187										
23.31	0	0	33	12	154	175	11	70	4729	793	187										
23.41	0	32	39	0	194	168	46	62	5477	653	184										
23.51	24	0	27	0	115	125	10	11	9216	636	269										
23.61	16	7	40	0	116	190	118	50	13217	533	364										
23.71	10	7	76	24	193	215	114	142	13554	307	391										
23.81	10	0	37	0	126	169	91	94	14227	550	540										
23.91	0	12	26	0	138	219	148	14	12199	192	585										
24.01	11	26	43	0	71	169	120	155	9626	499	600										
24.11	7	7	53	0	150	149	125	94	9730	438	693										
24.21	28	0	59	0	157	147	136	238	11473	524	731										
24.3	6	0	72	16	143	91	131	103	10650	563	780										
24.41	0	0	12	0	121	111	143	159	9768	710	796										
24.51	24	23	52	17	169	121	150	65	9880	527	815										
24.61	8	20	32	0	140	108	45	59	9829	641	836										
24.71	26	15	57	0	155	136	103	106	9073	635	918										
24.81	14	31	47	0	151	155	149	57	9049	543	1057										
24.91	21	0	58	28	185	111	92	127	10697	530	1048										
25.01	0	20	49	0	211	154	129	239	10908	336	844										
25.11	30	0	33	0	180	201	189	169	10600	275	562										
25.21	21	0	48	0	115	123	206	115	9099	337	411										
25.31	39	26	43	0	111	124	169	44	7402	731	376										
25.41	17	12	46	0	132	84	93	0	6308	533	407										
25.51	13	0	47	0	103	121	0	8	6756	597	468										
25.61	14	16	48	0	143	177	69	0	6874	685	538										
25.71	29	38	66	12	93	85	65	0	10331	477	649										
25.81	22	34	75	0	184	107	118	114	13412	416	2009										
25.91	12	39	87	0	116	139	271	227	16011	270	5013										
26.01	47	10	123	0	231	167	218	152	16415	187	6452										
26.11	28	44	105	0	354	186	145	350	15892	285	5998										
26.21	16	47	101	0	504	176	178	457	14613	310	5915										
26.3	12	81	147	0	399	217	163	301	13451	430	7622										
26.41	7	38	134	0	371	294	188	242	13128	365	9688										
26.5	43	87	104	0	389	189	165	267	8384	288	7549										
26.61	14	39	33	0	332	145	211	323	3332	25	3722										
26.7	8	11	49	0	157	77	217	250	1470	78	1951										
26.81	0	19	44	0	150	42	171	164	1037	168	1297										
26.91	0	9	32	0	133	62	219	177	1108	149	1224										
27.01	9	5	29	0	143	26	163	166	1034	0	1086										
27.11	9	26	61	0	122	71	62	122	967	166	1006										
27.21	16	0	34	0	127	47	146	127	916	182	912										
27.31	27	18	41	0	139	100	192	120	912	234	777										
27.41	16	9	35	0	93	105	119	86	1043	346	816										
27.51	20	18	30	11	156	87	71	139	1188	252	715										
27.6	0	0	41	4	195	154	188	59	1236	420	766										
27.71	15	10	41	0	173	148	106	146	1280	365	763										
27.81	17	32	37	0	145	106	118	17	1237	439	712										
27.91	0	0	32	8	168	105	201	123	1337	432	748										
28.01	14	20	48	0	193	99	149	156	1557	307	788										
28.1	0	0	47	12	133	81	176	41	1865	381	774										
28.21	0	0	24	0	204	46	210	191	1902	129	727										
28.31	0	21	29	0	163	28	277	208	1903	7	725										
28.41	11	22	25	0	137	89	255	219	1836	67	763										
28.51	0	7	38	0	146	44	232	175	1631	0	636										
28.61	7	21	25	0	138	0	242	163	1483	0	618										
28.71	4	14	41	0	176	86	226	163	1296	0	566										
28.81	32	16	22	0	146	126	203	176	1338	128	585										
28.91	11	0	23	11	143	99	156	148	1338	297	551										
29.01	33	27	48	0	126	70	135	234	1351	249	600										
29.11	13	0	40	6	127	128	167	50	1421	463	587										

29.21	9	0	38	0	123	103	186	115	1450	321	603										
29.31	19	12	38	0	182	104	94	86	1487	334	589										
29.41	15	0	52	26	158	147	126	151	1527	487	625										
29.51	8	21	44	41	225	158	142	159	1644	326	643										
29.61	0	0	55	26	131	61	207	164	1989	300	659										
29.71	12	20	25	0	159	95	98	171	1933	215	650										
29.81	0	19	53	0	195	33	126	200	1896	151	600										
29.91	9	7	25	20	126	60	195	229	1764	41	658										
30.01	13	0	28	0	193	56	257	238	1695	0	631										
30.11	0	7	33	0	135	10	334	126	1677	0	621										
30.21	0	10	29	0	100	39	235	231	1635	0	652										
30.31	11	20	34	0	162	21	254	153	1512	136	543										
30.4	13	6	36	0	129	69	109	130	1527	123	521										
30.51	0	12	26	0	142	55	139	137	1498	213	560										
30.6	12	16	33	6	119	79	145	48	1524	63	510										
30.71	0	0	32	0	196	193	157	191	1584	80	604										
30.8	0	14	37	0	167	152	103	89	1440	296	610										
30.91	11	9	37	0	174	132	45	92	1499	267	537										
31.01	0	20	22	0	207	113	165	160	1594	379	624										
31.11	29	13	29	0	231	74	171	67	1724	248	625										
31.2	13	13	20	32	219	100	155	146	1795	427	760										
31.31	16	0	27	0	199	98	151	163	1834	74	851										
31.41	18	17	24	0	149	50	256	264	1807	156	813										
31.51	15	7	21	0	170	83	282	124	1712	0	677										
31.6	0	0	28	12	188	93	208	267	1582	0	581										
31.71	44	28	43	0	149	39	96	187	1521	224	599										
31.81	24	14	49	0	160	77	206	203	1432	52	547										
31.91	14	19	41	0	189	117	131	162	1395	252	555										
32.01	5	0	36	0	169	282	143	242	1614	275	513										
32.11	14	0	28	0	207	583	92	242	1589	225	604										
32.21	0	6	14	27	130	184	73	254	1627	507	591										
32.3	15	0	27	0	178	213	55	97	1634	288	660										
32.41	24	17	36	0	203	178	100	140	1784	400	553										
32.51	0	0	26	0	185	149	139	108	1879	402	636										
32.61	15	10	26	0	241	164	158	162	1955	133	623										
32.71	12	17	19	0	190	115	248	139	1996	170	655										
32.81	0	8	37	0	220	54	142	180	1908	268	719										
32.9	0	9	64	13	248	57	246	174	1834	0	732										
33.01	14	26	32	27	146	49	152	253	1772	75	619										
33.11	6	0	39	0	226	80	202	136	1514	93	566										
33.21	0	9	56	0	203	133	172	210	1503	17	555										
33.31	7	4	38	0	131	147	131	163	1472	93	617										
33.4	14	20	35	11	234	71	57	131	1442	175	432										
33.51	28	16	50	0	171	150	17	119	1516	314	504										
33.6	16	20	31	9	177	182	26	127	1467	109	447										
33.71	0	12	13	0	195	234	0	34	1622	493	460										
33.8	17	29	41	0	185	166	0	9	1830	412	494										
33.91	10	18	49	0	161	154	133	66	1862	292	539										
34	12	8	31	0	183	86	45	159	1825	162	509										
34.11	10	17	50	0	187	126	96	214	1894	248	514										
34.21	18	17	32	10	203	107	144	185	1794	360	535										
34.31	17	0	35	0	214	79	213	244	1636	81	542										
34.41	0	23	15	0	209	140	198	241	1614	84	515										
34.51	0	20	19	0	204	41	242	195	1650	55	494										
34.61	0	10	44	0	252	105	177	169	1624	62	438										
34.7	16	11	40	0	172	111	144	112	1657	303	473										
34.81	10	0	31	0	164	119	78	165	1764	285	475										
34.91	10	0	21	21	213	166	140	92	1884	207	542										
35	10	0	24	0	281	149	45	108	1838	282	518										
35.1	6	16	21	0	236	52	92	71	1793	0	493										
35.21	13	0	20	0	214	81	93	137	1875	131	584										
35.31	14	35	39	0	196	88	165	223	1836	152	587										
35.41	0	32	31	0	222	102	155	164	1603	60	580										

35.51	9	5	27	0	208	54	113	200	1447	100	521										
35.61	5	0	26	11	194	52	159	60	1570	239	394										
35.71	16	17	39	0	190	81	91	114	1478	219	438										
35.81	19	14	26	0	154	91	74	93	1554	482	469										
35.9	27	13	49	0	209	111	70	311	1806	38	480										
36.01	9	0	33	0	215	72	69	97	1973	368	485										
36.11	11	28	22	0	248	36	132	111	2201	417	472										
36.21	0	0	12	0	184	59	151	143	2176	302	491										
36.3	0	19	15	0	211	61	170	252	2121	105	575										
36.41	19	14	31	0	218	20	111	137	1731	59	618										
36.51	23	25	40	0	219	39	164	160	1596	0	544										
36.61	0	12	29	7	165	17	132	189	1445	40	441										
36.71	16	12	38	7	225	99	86	78	1440	6	425										
36.81	11	10	39	0	211	38	25	34	1461	254	411										
36.91	15	0	29	0	254	110	42	94	1606	567	439										
37.01	0	0	23	0	246	112	90	136	1647	359	428										
37.11	0	10	34	14	210	124	28	199	1697	548	401										
37.2	0	0	17	0	207	105	101	16	1913	367	482										
37.31	17	7	37	0	251	124	44	70	2398	363	536										
37.41	0	8	21	0	265	95	235	159	2328	251	554										
37.51	30	20	36	0	246	63	189	205	2155	72	585										
37.61	4	10	53	0	247	33	112	252	1799	0	486										
37.7	0	0	19	0	219	41	183	353	1684	58	436										
37.81	13	28	52	0	214	32	118	335	1527	14	441										
37.9	16	0	14	0	220	91	160	118	1531	296	461										
38.01	0	5	23	20	228	136	154	86	1604	228	387										
38.1	15	28	54	0	195	123	55	24	1645	239	416										
38.21	10	25	33	33	186	167	72	46	1720	371	422										
38.31	9	21	42	0	248	172	15	19	1747	361	447										
38.4	18	0	31	0	206	102	96	202	2033	361	482										
38.51	16	24	32	21	228	187	162	125	2300	464	511										
38.6	13	21	52	0	223	66	116	134	2369	207	557										
38.71	31	8	26	0	218	133	176	278	2303	185	599										
38.81	19	18	38	0	274	113	230	268	2172	0	555										
38.91	13	7	15	0	282	86	126	241	1962	41	568										
39.01	21	0	24	14	188	75	208	175	1864	0	525										
39.11	0	5	36	0	256	97	108	354	1878	114	470										
39.21	0	5	41	15	230	89	166	253	1918	201	412										
39.31	8	21	49	8	169	212	63	188	1901	334	511										
39.41	32	0	55	0	233	184	65	250	1862	473	513										
39.51	7	0	51	0	217	119	112	100	1918	465	492										
39.61	11	17	45	14	163	212	79	163	2005	439	593										
39.7	14	14	31	0	209	285	96	95	2245	525	578										
39.81	23	0	69	0	234	295	205	202	2443	494	572										
39.9	13	0	36	0	274	138	185	285	2453	69	556										
40.01	0	0	36	0	235	75	176	126	2414	155	594										
40.11	0	17	19	0	238	45	177	244	2348	17	552										
40.21	0	0	25	0	246	85	181	277	2248	0	586										
40.31	0	0	23	0	244	8	192	133	2073	80	566										
40.41	0	0	39	0	251	65	156	162	1927	83	566										
40.51	0	0	20	0	241	105	167	161	1981	0	458										
40.61	0	7	46	0	201	92	106	55	2096	321	475										
40.71	4	15	34	0	217	123	102	142	2128	345	515										
40.81	9	17	37	10	224	65	0	6	2425	383	584										
40.91	6	25	31	0	211	297	46	162	2554	501	617										
41.01	7	14	39	0	256	199	158	260	2623	346	613										
41.11	0	0	22	0	270	70	210	167	2256	43	568										
41.21	18	16	36	0	237	90	156	181	2101	34	527										
41.31	25	16	41	0	241	84	177	198	1812	126	460										
41.41	0	17	23	0	218	29	117	191	1716	72	433										
41.5	0	9	25	0	259	119	162	121	1756	115	475										
41.61	13	24	35	16	209	159	136	249	1826	118	480										
41.71	0	0	29	26	176	139	53	186	1835	237	490										

41.81	0	0	28	0	235	157	47	40	1905	370	493										
41.91	0	0	33	0	233	210	116	122	2169	178	596										
42.01	0	11	16	7	284	190	144	266	2190	411	603										
42.11	9	16	16	0	228	130	142	220	2023	156	581										
42.21	24	29	65	0	216	98	146	220	1883	117	577										
42.31	0	0	44	12	168	81	121	262	1849	61	557										
42.41	11	6	26	0	191	197	186	272	1776	310	473										
42.51	0	0	22	0	220	225	167	320	1658	429	422										
42.61	0	0	33	0	156	123	196	138	1507	332	381										
42.71	0	11	39	0	132	98	101	28	1743	379	408										
42.81	13	0	39	0	172	132	56	40	1836	528	441										
42.91	11	38	34	18	259	185	43	98	2063	390	437										
43	5	0	41	0	215	172	75	188	2222	275	574										
43.11	35	21	44	21	224	115	103	28	2296	160	596										
43.2	0	5	0	0	259	67	235	230	2090	99	672										
43.31	14	27	49	0	182	99	157	230	1814	24	603										
43.4	42	28	37	0	202	162	130	24	1407	197	401										
43.51	17	8	31	28	149	155	0	48	1207	495	356										
43.6	4	7	19	5	203	424	73	131	1325	589	378										
43.7	0	0	33	0	161	508	8	162	1589	732	428										
43.81	13	8	24	0	183	268	51	64	1759	624	462										
43.9	38	16	48	41	168	223	106	49	2006	558	508										
44.01	0	5	38	70	191	425	71	206	2011	355	509										
44.11	0	10	36	31	189	306	20	132	1827	477	477										
44.21	13	31	37	0	222	249	82	70	1963	328	535										
44.3	0	14	21	0	161	237	55	102	2065	413	516										
44.41	0	10	34	0	154	79	83	163	2073	203	493										
44.5	13	30	37	0	202	96	158	221	1932	302	500										
44.61	7	0	0	11	155	56	140	200	1844	374	457										
44.7	11	7	20	0	168	125	118	208	2077	438	450										
44.81	9	6	13	0	219	68	128	210	2540	354	442										
44.91	0	6	56	0	164	123	51	85	2320	406	446										
45	0	0	27	0	177	73	54	53	1918	395	457										
45.11	14	0	30	0	202	101	119	158	1759	393	430										
45.21	18	16	44	0	147	38	137	299	1639	125	531										
45.3	0	8	33	0	190	72	138	227	1628	292	508										
45.4	0	22	15	0	192	92	192	180	1481	82	385										
45.51	0	15	22	0	215	49	157	0	1546	253	389										
45.61	0	0	27	0	162	86	0	11	1561	555	416										
45.71	0	16	12	0	143	101	93	173	1814	382	403										
45.8	8	14	16	0	187	106	38	113	1920	506	462										
45.91	0	7	38	0	157	127	175	142	2026	367	572										
46.01	15	0	8	0	143	79	159	176	2244	288	707										
46.11	0	15	45	0	199	61	159	219	2231	204	689										
46.2	0	7	35	0	187	57	159	167	1873	143	473										
46.31	0	0	29	0	188	49	58	158	1799	227	432										
46.41	0	23	39	0	182	81	101	163	1935	429	495										
46.5	0	0	18	0	169	101	100	252	2040	324	502										
46.61	5	16	35	0	163	112	143	185	2038	132	539										
46.7	6	0	4	0	130	86	61	68	1862	193	444										
46.81	18	0	17	0	172	97	98	220	1775	429	438										
46.91	13	6	26	24	181	93	57	0	1828	512	423										
47.01	25	20	34	0	177	123	128	10	1879	682	426										
47.11	9	0	24	0	164	119	24	103	2254	512	538										
47.21	14	4	29	0	209	96	176	159	2573	275	633										
47.31	0	8	19	0	207	92	161	195	2521	225	584										
47.41	11	19	33	9	211	128	91	174	2268	271	500										
47.51	0	21	25	0	275	55	10	223	1997	219	491										
47.61	16	14	33	0	172	34	213	168	2062	214	473										
47.71	10	15	37	0	185	41	37	141	2044	385	575										
47.8	23	37	19	0	179	82	81	97	2092	244	456										
47.91	6	19	26	0	163	63	167	62	2037	334	479										
48	17	20	24	0	204	64	138	140	1895	296	467										

48.11	11	0	8	10	111	97	74	182	1872	427	431										
48.21	0	4	30	8	177	109	112	127	1912	459	437										
48.31	19	25	27	0	230	87	100	0	1970	531	404										
48.41	0	0	20	0	145	136	167	96	2154	520	486										
48.51	0	6	11	0	175	87	183	130	2468	53	532										
48.61	0	7	38	0	140	7	120	236	2355	198	676										
48.71	0	0	23	0	188	51	145	210	2114	45	544										
48.81	32	11	40	0	133	56	105	113	1946	0	478										
48.9	0	4	26	0	211	40	218	202	1801	7	521										
49.01	0	12	21	0	176	110	145	163	1687	195	440										
49.11	0	0	22	0	148	114	146	76	1803	246	409										
49.21	21	24	25	0	182	110	18	40	1880	242	413										
49.31	21	15	24	0	184	111	66	107	2054	425	428										
49.41	0	16	20	0	207	120	280	50	2213	596	471										
49.5	26	18	38	0	202	101	149	53	2215	307	526										
49.61	26	23	28	0	231	116	165	86	2582	475	573										
49.71	15	0	16	0	201	81	46	119	2751	523	664										
49.81	0	24	30	0	217	108	118	213	2762	229	644										
49.91	7	7	24	0	172	24	179	228	2607	158	604										
50	0	20	36	0	174	40	145	72	2433	162	578										
50.11	12	24	31	0	199	35	149	96	2275	296	532										
50.21	32	9	33	0	166	98	54	87	2053	426	443										
50.31	11	10	27	0	188	78	59	91	2138	490	388										
50.41	10	23	51	0	206	82	64	147	2025	463	446										
50.51	16	18	30	0	206	90	95	176	2240	354	509										
50.61	12	18	28	0	190	148	170	103	2520	590	483										
50.7	9	25	42	0	231	129	102	158	2602	296	527										
50.81	10	0	34	0	208	40	189	220	2632	132	567										
50.91	23	25	19	0	223	47	188	264	2478	332	551										
51.01	12	15	27	0	292	35	107	271	2275	300	459										
51.11	12	11	33	0	226	98	181	209	2249	127	434										
51.2	5	14	15	0	181	47	171	144	2216	273	442										
51.31	0	22	24	0	176	103	73	79	2152	335	469										
51.41	19	10	44	0	214	142	169	186	2258	417	459										
51.5	0	0	18	0	249	72	162	133	2372	456	577										
51.61	13	0	21	0	179	104	90	149	2425	155	557										
51.71	22	39	39	0	239	162	141	182	2373	187	508										
51.81	0	9	34	11	194	121	190	195	2331	164	537										
51.9	0	0	22	0	193	68	99	197	2353	183	492										
52.01	28	17	36	0	181	24	185	288	2224	123	431										
52.11	0	0	15	6	169	8	103	275	2149	362	463										
52.2	20	15	57	0	230	128	102	159	2214	323	420										
52.3	0	27	16	29	212	114	80	61	2276	384	415										
52.41	0	0	24	0	205	118	170	0	2393	528	437										
52.51	0	4	22	0	214	144	90	71	2480	672	464										
52.6	0	0	18	0	198	100	72	61	2710	355	528										
52.71	7	29	26	0	265	131	128	86	3011	384	684										
52.81	0	24	29	0	240	28	137	217	3006	188	709										
52.91	16	0	29	0	235	89	173	125	2799	130	649										
53.01	6	13	0	0	230	127	185	225	2472	328	586										
53.11	21	6	17	0	196	103	149	257	2191	305	519										
53.2	16	0	33	0	207	109	35	78	2139	341	424										
53.31	11	0	17	0	166	90	34	36	2010	197	424										
53.41	10	16	47	0	220	133	137	158	2180	424	439										
53.5	0	11	33	0	224	101	115	74	2053	447	443										
53.61	16	28	41	0	194	173	97	51	2142	364	479										
53.71	7	0	33	0	164	110	127	220	2252	361	542										
53.81	0	20	41	0	231	91	108	155	2406	364	484										
53.91	17	26	43	0	241	75	145	199	2669	319	595										
54.01	8	16	22	0	253	47	227	135	2613	274	585										
54.11	7	21	23	0	210	89	133	222	2517	87	626										
54.2	0	24	31	0	240	71	166	249	2343	195	552										
54.31	0	10	28	0	228	63	296	167	2322	207	480										

54.41	14	11	45	0	163	80	197	215	2338	347	496										
54.51	0	8	15	0	272	90	159	154	2244	269	458										
54.61	10	14	27	0	220	75	137	145	2356	262	444										
54.71	0	0	26	0	227	140	81	58	2465	441	449										
54.81	14	18	15	0	219	102	112	101	2469	390	483										
54.91	5	5	20	24	248	123	45	142	2586	478	502										
55.01	0	16	40	0	256	132	100	212	2700	386	600										
55.11	0	19	29	0	248	80	94	147	2822	403	618										
55.21	0	16	27	0	252	92	83	114	2652	0	610										
55.31	0	21	34	0	229	62	178	184	2384	70	596										
55.41	10	0	31	0	186	53	260	114	2263	100	586										
55.51	29	17	28	0	162	76	213	167	2177	0	492										
55.61	0	0	32	0	164	64	121	238	2238	338	539										
55.7	0	17	32	0	287	141	137	212	2555	213	595										
55.81	13	22	40	0	257	96	77	234	2668	296	692										
55.91	0	0	30	0	217	156	93	120	2653	511	639										
56.01	0	0	28	36	235	125	81	62	2653	504	514										
56.11	5	6	47	0	243	139	76	115	2817	470	478										
56.21	0	18	35	6	200	72	55	77	2756	371	514										
56.31	8	5	40	0	174	161	131	55	2799	346	510										
56.41	0	0	35	0	207	80	145	177	2727	186	510										
56.51	9	11	26	0	230	124	208	199	2651	116	525										
56.6	0	25	44	21	236	33	110	250	2581	123	502										
56.71	0	5	20	0	216	68	147	134	2580	132	585										
56.81	27	27	33	0	228	67	219	275	2520	162	575										
56.91	20	0	0	0	216	142	135	221	2334	245	500										
57	19	23	25	0	226	55	174	261	2273	249	499										
57.11	0	0	37	0	233	91	131	148	2266	303	474										
57.21	14	23	52	0	216	117	138	61	2213	295	467										
57.3	16	7	19	0	244	41	82	128	2418	225	448										
57.4	19	10	26	0	199	118	79	145	2503	346	426										
57.51	0	0	10	0	217	81	73	31	2474	80	431										
57.61	25	0	28	0	233	81	139	47	2689	336	498										
57.71	46	25	30	0	225	75	126	9	2808	397	510										
57.81	26	0	34	0	256	91	84	122	2814	153	503										
57.91	7	15	33	0	216	111	73	115	3090	551	541										
58.01	8	21	24	19	259	118	121	189	3130	441	605										
58.11	0	13	33	0	268	79	164	57	3166	361	560										
58.2	0	0	47	0	224	130	153	128	3121	227	587										
58.3	0	11	22	0	275	92	138	82	3025	429	600										
58.41	11	10	26	0	306	121	143	127	2953	300	513										
58.5	18	21	22	0	266	71	116	219	2925	342	535										
58.61	21	20	36	0	275	168	225	264	2713	388	549										
58.71	0	19	23	0	234	124	152	175	2803	145	498										
58.81	4	0	43	0	323	93	116	244	2857	329	571										
58.91	0	10	22	0	236	89	76	188	2957	350	578										
59	0	10	33	0	272	151	99	265	2997	206	570										
59.11	18	25	40	0	306	75	140	186	2909	45	566										
59.2	0	11	44	0	223	156	161	174	2851	386	527										
59.31	0	0	45	28	309	73	126	59	2798	271	554										
59.41	5	6	33	0	260	64	104	200	2699	454	515										
59.51	6	0	8	0	265	38	133	194	2539	317	534										
59.61	26	5	32	0	268	102	159	181	2609	324	577										
59.71	20	0	16	0	269	72	112	122	2579	150	519										
59.81	8	0	22	0	178	72	147	56	2467	397	466										
59.91	19	36	33	0	174	70	111	89	2448	316	451										
60.01	13	5	22	0	169	108	131	89	2509	347	519										
60.11	9	11	24	0	207	127	97	154	2637	296	438										
60.2	5	23	40	0	180	135	61	128	2727	269	479										
60.31	14	17	37	0	209	108	23	64	2619	387	511										
60.4	0	24	42	0	347	151	136	124	2874	454	513										
60.51	24	0	44	0	210	104	92	11	2795	271	508										
60.6	7	14	18	0	247	124	63	9	2812	443	479										

60.71	4	16	22	0	292	146	97	132	2824	480	508										
60.81	20	5	18	0	270	133	172	72	3191	122	541										
60.91	0	28	51	0	303	133	74	193	3245	215	564										
61.01	11	0	30	6	252	75	212	232	3068	164	567										
61.11	0	0	23	0	246	91	124	308	2841	70	590										
61.2	16	16	40	0	233	128	127	267	2728	31	538										
61.31	22	6	19	0	272	56	221	219	2726	327	453										
61.4	10	14	33	0	214	107	206	143	2493	161	421										
61.51	0	0	30	37	276	84	134	180	2497	148	480										
61.6	17	0	23	0	199	99	136	175	2544	120	406										
61.71	12	32	21	0	251	123	155	252	2551	85	466										
61.81	0	0	19	0	240	62	112	114	2592	257	438										
61.91	0	0	12	0	258	48	121	117	2616	229	440										
62.01	10	0	17	0	258	166	89	146	2702	447	493										
62.11	0	10	19	0	214	112	108	81	2824	344	515										
62.21	17	28	47	0	237	147	176	105	2936	172	488										
62.31	0	6	28	0	244	64	16	101	2860	445	469										
62.41	17	0	26	0	227	95	80	65	2955	442	535										
62.5	0	0	40	0	229	131	95	167	3036	285	584										
62.61	0	0	35	0	251	118	155	121	2992	387	514										
62.7	18	15	25	0	229	83	128	117	3146	360	483										
62.81	6	7	40	0	281	55	114	214	3518	234	661										
62.91	9	12	51	0	276	100	192	231	3563	409	607										
63.01	0	22	27	0	295	156	187	235	3374	408	581										
63.1	6	0	17	0	260	42	180	144	3186	184	589										
63.21	24	13	33	0	269	25	251	257	2966	87	570										
63.31	14	0	42	0	208	0	228	180	3149	71	645										
63.41	0	15	23	0	281	39	167	149	2962	231	567										
63.51	12	15	19	0	264	61	190	247	2890	242	569										
63.61	27	8	33	0	272	110	146	185	2774	312	542										
63.7	15	10	24	0	250	99	160	161	2825	186	507										
63.81	27	5	28	0	262	93	65	15	2858	449	512										
63.91	9	8	38	0	289	51	136	57	3042	366	558										
64.01	12	0	33	0	277	157	165	105	3061	280	532										
64.11	12	25	28	0	257	60	96	123	2994	583	527										
64.2	16	15	8	0	283	90	42	0	3059	454	539										
64.31	7	0	19	0	289	91	61	104	3083	379	537										
64.41	5	16	38	0	256	112	30	92	3177	340	643										
64.51	11	6	41	0	267	146	131	198	3730	375	663										
64.61	0	14	46	0	279	27	165	188	3930	258	766										
64.71	0	20	58	0	272	152	262	298	3893	207	793										
64.81	8	0	43	0	272	19	204	207	3571	128	694										
64.91	21	7	27	0	240	38	259	191	3303	107	706										
65.01	8	18	48	0	233	150	211	341	3004	79	626										
65.11	0	14	22	12	243	71	165	130	2672	162	545										
65.21	6	16	41	0	248	99	139	133	2663	399	537										
65.31	27	12	50	0	239	86	136	83	2915	324	583										
65.41	0	21	14	0	248	131	90	62	2911	305	593										
65.51	6	41	28	0	266	112	143	268	3022	446	610										
65.61	17	23	38	0	270	76	176	73	2984	378	566										
65.71	24	18	36	0	252	101	103	73	3054	688	586										
65.81	0	21	29	0	281	116	92	43	3167	338	598										
65.9	7	0	35	0	293	118	99	31	2995	294	596										
66.01	37	15	36	0	302	66	108	70	2962	347	580										
66.1	31	19	19	6	267	97	0	78	3016	527	503										
66.21	15	12	16	7	234	110	119	112	3082	492	548										
66.31	9	38	23	0	302	117	86	180	3209	271	517										
66.41	6	7	35	0	239	106	70	125	3219	364	583										
66.51	0	15	35	0	246	87	137	115	3327	420	550										
66.6	0	0	21	0	263	80	0	139	3357	392	582										
66.71	12	6	21	4	242	85	140	114	3642	138	607										
66.8	12	4	12	0	308	84	234	190	3454	269	603										
66.91	17	12	31	0	288	34	179	305	3244	237	584										

67.01	10	34	13	0	258	49	168	307	3070	107	511										
67.11	5	0	25	0	255	39	214	206	2986	116	522										
67.2	9	0	13	0	214	70	294	238	2639	81	488										
67.31	0	15	40	0	252	72	224	158	2478	49	443										
67.41	6	4	13	0	297	8	152	253	2357	207	410										
67.5	0	0	21	0	169	53	120	197	2487	162	443										
67.6	13	11	35	0	221	76	182	160	2506	340	489										
67.71	26	14	41	0	208	99	153	88	2574	247	513										
67.8	19	0	20	0	238	109	154	137	2568	302	488										
67.91	8	22	18	0	197	201	13	137	2589	418	396										
68.01	24	16	49	0	228	135	130	100	2740	423	447										
68.1	15	16	29	0	208	105	108	230	2997	403	463										
68.21	21	32	32	0	268	109	93	156	2998	456	469										
68.31	10	12	28	0	232	515	145	66	3021	566	476										
68.4	16	0	43	0	304	468	8	179	3126	289	433										
68.51	6	25	32	0	229	135	132	131	3248	361	457										
68.61	30	36	46	0	261	117	116	77	3325	287	471										
68.71	0	0	18	0	264	80	166	106	3570	113	432										
68.81	5	5	24	0	236	43	156	98	3342	358	448										
68.9	19	12	19	0	233	72	69	180	3362	317	487										
69.01	0	9	23	0	240	134	161	174	3162	211	469										
69.11	8	13	19	0	278	44	159	115	3099	246	469										
69.2	0	9	28	0	252	98	104	201	3089	248	402										
69.31	0	18	37	9	209	64	163	192	3086	215	385										
69.4	0	6	0	0	266	120	149	232	2922	226	421										
69.51	0	0	38	0	234	148	92	151	2827	271	418										
69.61	27	12	44	0	202	43	129	140	3036	274	431										
69.71	18	0	41	0	228	80	44	89	2953	460	422										
69.81	0	26	17	16	212	105	77	190	3080	220	411										
69.91	26	13	35	0	277	108	91	95	3107	354	412										
70.01	7	11	31	0	260	35	73	17	3152	462	399										
70.11	0	0	13	0	315	77	63	114	3196	459	422										
70.21	26	0	38	0	268	93	100	27	3383	428	436										
70.31	11	0	16	0	299	49	114	203	3496	234	443										
70.41	6	14	34	0	333	43	159	230	3494	357	463										
70.5	14	0	26	0	304	103	115	218	3414	268	477										
70.61	8	9	11	0	258	39	140	134	3352	190	492										
70.71	0	0	38	0	266	79	185	200	3203	103	424										
70.81	8	19	24	0	296	137	149	124	3157	103	433										
70.91	0	0	14	0	258	63	164	216	3262	93	448										
71	17	6	33	0	240	92	189	65	3204	135	447										
71.11	14	0	16	0	253	124	196	218	3036	101	414										
71.21	0	0	17	0	244	53	131	132	3177	188	417										
71.31	0	9	37	0	258	99	108	176	3222	361	446										
71.41	18	5	12	0	303	116	150	143	3096	234	371										
71.51	7	9	43	0	242	62	156	214	3099	217	431										
71.6	0	15	26	5	237	126	162	112	3225	349	450										
71.71	0	7	20	0	271	110	74	120	3210	325	378										
71.81	0	13	10	0	232	83	134	96	3150	384	390										
71.91	0	0	40	0	240	85	32	101	3206	298	429										
72.01	0	15	13	0	223	117	155	146	3173	513	429										
72.11	0	0	22	0	279	153	164	209	3307	407	471										
72.21	14	18	24	0	328	86	74	119	3255	342	461										
72.31	26	11	31	0	297	77	59	106	3287	470	527										
72.4	8	0	35	0	261	118	96	169	3362	472	483										
72.51	13	6	46	0	250	82	31	159	3523	555	462										
72.6	19	0	26	0	229	120	54	144	3446	327	428										
72.71	21	0	26	0	256	128	37	151	3501	489	497										
72.8	24	28	6	0	270	91	112	32	3582	374	437										
72.91	0	5	21	0	319	80	216	137	3624	452	509										
73.01	24	0	35	0	244	105	69	172	3668	316	457										
73.11	5	8	54	0	276	164	269	273	3574	147	449										
73.2	19	4	27	0	254	76	156	186	3538	258	450										

73.31	30	5	33	15	237	57	182	223	3345	159	465										
73.4	9	12	55	0	285	33	156	105	3497	204	447										
73.51	23	14	58	0	283	109	153	246	3509	0	485										
73.61	0	10	32	0	261	85	176	203	3335	26	498										
73.71	5	0	25	0	272	73	218	72	3165	165	404										
73.81	0	0	23	0	244	89	273	282	3115	203	432										
73.91	11	0	7	0	215	45	167	60	3191	214	427										
74.01	0	24	21	0	230	41	139	133	3269	122	427										
74.11	0	24	34	0	236	88	173	203	3153	216	408										
74.2	0	0	25	0	284	33	185	155	3218	276	425										
74.3	30	8	26	0	253	100	200	205	3279	197	427										
74.41	24	0	23	0	331	50	189	201	3338	225	440										
74.51	0	0	36	10	270	62	142	186	3396	199	423										
74.6	9	0	35	7	321	75	69	102	3445	329	461										
74.71	0	0	20	0	328	70	141	162	3490	413	439										
74.8	0	14	14	54	221	45	128	193	3622	269	432										
74.91	7	13	32	0	272	29	0	131	3569	253	512										
75.01	16	0	0	0	294	76	93	283	3736	667	476										
75.1	19	0	22	0	273	83	92	56	3872	216	419										
75.21	31	18	28	0	223	95	128	155	3775	401	443										
75.3	10	7	43	0	309	103	140	202	4061	347	508										
75.41	10	0	31	0	314	135	153	111	3939	362	454										
75.51	0	11	35	0	328	140	146	98	3903	434	479										
75.61	0	12	5	0	342	78	103	246	3907	348	483										
75.71	10	0	38	0	260	19	174	51	4094	437	483										
75.81	8	21	16	0	331	72	134	123	3890	382	489										
75.91	18	0	8	0	404	42	110	0	3885	296	427										
76.01	13	0	13	0	277	124	122	195	4040	364	493										
76.11	7	31	17	0	276	28	193	139	4035	306	526										
76.21	6	0	23	0	300	49	155	208	3973	234	517										
76.31	0	16	13	0	292	42	198	221	3716	205	542										
76.4	8	11	31	0	320	56	146	237	3779	219	575										
76.51	0	0	22	0	256	64	211	180	3537	55	501										
76.61	0	10	38	8	280	60	151	213	3478	132	512										
76.7	15	0	24	0	279	73	201	213	3332	54	463										
76.81	11	4	26	0	271	20	195	162	3358	197	442										
76.9	0	9	7	0	260	29	253	208	3301	227	470										
77.01	16	30	20	11	282	40	108	204	3403	103	436										
77.11	29	12	21	0	276	36	175	209	3438	283	400										
77.21	0	11	39	0	298	50	198	223	3145	156	393										
77.3	0	23	20	16	252	54	170	62	3100	244	486										
77.4	0	13	38	0	285	67	172	70	3036	333	370										
77.51	0	13	38	0	333	109	94	79	3169	290	467										
77.61	18	9	29	0	285	93	108	285	3377	148	432										
77.7	11	13	26	0	295	108	143	115	3531	377	397										
77.81	8	0	24	19	203	65	49	28	3567	268	404										
77.91	18	12	36	0	300	89	88	146	3512	500	438										
78	0	0	25	0	269	98	202	114	3440	393	471										
78.11	12	7	24	0	249	74	176	146	3606	373	455										
78.2	8	0	13	0	261	145	193	218	3695	417	436										
78.31	9	11	21	0	278	96	170	142	3606	354	427										
78.41	0	0	25	0	285	100	235	105	3666	127	436										
78.5	9	11	21	0	302	88	76	75	3643	159	459										
78.61	20	0	16	0	309	93	61	31	3851	443	449										
78.71	25	28	33	0	331	46	143	0	3962	273	503										
78.8	0	0	24	0	381	20	152	177	4028	231	446										
78.91	0	0	0	0	319	61	84	35	3972	349	513										
79.01	21	7	16	0	344	80	111	57	4057	479	482										
79.11	8	25	36	0	349	88	52	0	4113	449	560										
79.21	5	8	27	0	280	77	44	28	4181	245	526										
79.31	17	5	23	0	334	126	158	132	4167	330	594										
79.4	21	7	30	0	308	139	161	154	4189	468	556										
79.51	8	0	15	0	238	100	102	30	4073	309	561										

79.6	9	6	43	0	371	85	128	109	4116	388	497										
79.71	0	9	19	0	310	60	80	65	4072	371	507										
79.8	0	9	33	0	270	58	185	144	4189	265	559										
79.91	0	0	44	6	308	66	185	153	4353	398	544										
80.01	10	0	34	0	290	82	161	120	4194	303	581										
80.11	16	30	41	0	294	118	275	228	4046	86	588										
80.21	21	14	34	0	293	111	169	215	3992	169	524										
80.3	0	0	35	0	344	24	194	201	3976	106	509										
80.41	9	22	37	0	271	64	174	255	3848	211	588										
80.5	16	0	24	0	330	58	216	164	3689	64	516										
80.61	22	13	33	0	275	129	218	182	3763	125	518										
80.71	10	27	49	0	254	56	58	255	3684	13	534										
80.81	13	21	36	0	275	72	168	208	3666	172	557										
80.91	0	18	36	0	274	76	191	167	3706	209	489										
81.01	13	16	40	0	297	59	243	177	3893	65	486										
81.1	0	9	21	0	247	119	176	176	3781	170	527										
81.21	8	0	20	0	305	27	249	205	3827	193	536										
81.31	30	17	36	24	293	122	178	186	3798	312	555										
81.41	0	10	15	0	265	149	243	190	3937	257	524										
81.5	17	15	25	13	366	63	112	196	3622	144	532										
81.61	0	0	29	0	245	112	118	191	3938	353	521										
81.71	0	0	33	0	302	77	59	26	4129	454	606										
81.81	28	0	21	0	319	125	129	116	4277	336	477										
81.91	20	18	34	0	336	104	130	68	4099	372	537										
82.01	9	0	21	0	284	96	200	134	3884	422	574										
82.1	6	18	29	0	317	133	127	117	3970	372	572										
82.21	14	20	35	0	271	103	13	78	3985	204	522										
82.31	16	4	45	0	279	118	157	159	4092	389	523										
82.4	0	14	29	16	298	75	177	0	4095	289	547										
82.51	9	6	34	15	352	71	112	108	4144	405	604										
82.6	15	22	24	0	332	97	103	61	4094	279	513										
82.71	14	20	31	0	301	74	0	49	4179	500	548										
82.81	0	10	23	0	322	108	154	199	4233	491	586										
82.91	7	10	33	0	329	51	38	101	4497	499	521										
83.01	5	5	20	0	323	142	115	212	4536	286	543										
83.11	11	5	22	0	329	136	70	136	4445	256	555										
83.21	0	0	30	10	340	110	130	219	4362	326	581										
83.31	4	0	13	0	337	61	87	83	4357	424	606										
83.41	6	22	54	0	327	91	230	45	4407	331	589										
83.51	0	0	39	0	321	107	17	55	4430	492	627										
83.61	4	0	34	11	361	99	213	109	4643	597	599										
83.71	0	19	40	0	358	87	128	104	4772	306	656										
83.81	0	0	37	0	332	63	139	71	4848	320	601										
83.9	9	22	19	0	356	55	154	159	5052	247	645										
84.01	5	0	32	0	345	59	140	175	5083	413	624										
84.11	0	7	28	0	340	91	11	169	5035	291	631										
84.2	12	0	32	0	352	44	138	167	4727	228	662										
84.31	12	6	26	0	332	51	139	211	4849	245	627										
84.41	29	4	38	0	304	61	191	91	4681	88	608										
84.5	18	10	34	0	333	55	148	260	4460	269	605										
84.61	11	7	27	0	343	21	183	189	4361	52	493										
84.71	18	5	25	0	313	70	199	214	4267	116	593										
84.8	9	19	37	0	317	70	156	213	4087	387	558										
84.91	13	5	25	0	389	33	193	150	4110	97	521										
85	0	5	14	0	385	25	255	129	4064	73	517										
85.11	7	13	33	0	278	75	207	86	4079	180	559										
85.21	5	6	26	0	390	63	199	289	3848	264	532										
85.3	9	26	41	0	354	74	152	119	3868	310	540										
85.41	38	18	31	0	315	54	85	104	3929	252	533										
85.5	8	12	35	6	322	107	214	160	3892	223	472										
85.61	5	0	18	0	343	87	152	90	3687	220	488										
85.71	6	11	25	0	308	44	77	99	3814	329	479										
85.81	6	21	10	0	274	98	68	76	4042	352	530										

85.91	11	0	41	8	309	78	109	245	4002	227	595										
86	21	0	25	0	344	53	154	94	3815	318	550										
86.11	12	24	25	0	334	95	117	181	3768	284	493										
86.21	16	0	45	0	335	165	61	107	3706	407	554										
86.3	23	34	20	0	289	47	108	142	3790	484	480										
86.41	16	6	9	0	327	90	114	138	4072	518	525										
86.5	0	0	9	33	311	88	127	193	4088	375	588										
86.61	0	0	38	0	296	87	100	111	3988	226	548										
86.71	0	6	22	0	377	47	119	117	4092	173	562										
86.81	24	6	34	0	310	14	166	79	4085	386	553										
86.91	0	0	39	0	330	86	166	131	4317	310	549										
87.01	8	7	58	0	336	63	218	122	4092	399	566										
87.11	19	17	13	0	314	146	173	183	4138	304	532										
87.21	0	9	22	11	329	52	145	252	4132	239	524										
87.31	15	8	35	0	318	46	105	115	4222	331	571										
87.4	0	0	20	0	317	131	133	100	4150	309	509										
87.51	23	6	45	0	368	74	66	85	4214	280	544										
87.6	0	14	38	0	382	51	100	79	4312	431	561										
87.71	0	0	22	0	347	65	160	134	4302	472	547										
87.81	15	6	27	0	357	102	121	152	4300	426	555										
87.91	16	9	57	0	417	105	180	241	4452	428	537										
88.01	22	7	21	0	330	67	156	134	4613	243	585										
88.11	0	11	23	0	344	78	78	123	4622	246	622										
88.21	6	0	32	0	362	74	93	251	4535	504	595										
88.31	0	6	21	0	308	112	96	130	4690	458	594										
88.41	36	0	19	4	363	156	154	150	4648	539	603										
88.51	5	0	31	0	386	145	223	118	4834	243	687										
88.61	19	10	57	0	440	45	83	122	4870	607	679										
88.71	0	8	35	0	430	71	141	209	5005	380	634										
88.81	16	0	35	0	354	68	115	124	5481	504	583										
88.9	10	0	27	0	351	126	149	121	5485	461	636										
89.01	6	23	46	0	343	89	124	200	5269	255	652										
89.1	12	14	35	0	417	42	149	207	5129	220	652										
89.21	16	0	37	0	445	12	61	141	5091	308	646										
89.3	9	18	24	0	355	71	118	205	4872	330	649										
89.41	11	0	47	8	339	102	162	263	4732	499	638										
89.51	16	9	19	5	399	106	125	87	4652	294	607										
89.6	0	24	21	0	369	98	71	181	4789	172	624										
89.71	9	0	29	0	355	95	51	171	4653	248	663										
89.81	13	9	58	0	403	95	131	191	4555	216	613										
89.91	8	10	20	0	344	80	225	250	4642	154	580										
90.01	0	17	31	0	325	147	116	164	4556	356	630										
90.11	0	16	44	0	400	84	145	106	4578	226	593										
90.2	0	18	30	0	359	53	159	115	4523	530	602										
90.31	15	0	31	0	351	95	107	121	4603	169	601										
90.41	8	11	19	0	346	86	106	124	4568	245	549										
90.51	29	10	25	0	337	130	182	203	4598	209	577										
90.61	4	0	39	0	378	61	156	145	4423	475	561										
90.71	16	19	43	0	348	73	121	86	4392	264	653										
90.81	12	22	48	14	348	65	109	197	4419	344	553										
90.91	21	17	53	0	311	123	130	195	4428	394	606										
91.01	11	7	22	0	380	98	148	249	4362	285	571										
91.11	7	0	57	0	336	86	137	148	4344	368	573										
91.21	0	6	19	0	390	32	188	174	4445	241	593										
91.31	7	13	28	0	320	50	111	128	4434	245	563										
91.41	12	0	26	0	364	97	147	221	4403	292	621										
91.51	31	0	25	0	422	115	139	56	4447	326	572										
91.61	9	11	28	0	382	123	90	118	4418	354	636										
91.71	14	12	30	0	335	147	225	195	4296	251	555										
91.81	15	6	54	0	309	61	105	134	4374	204	580										
91.9	0	0	50	0	292	106	131	293	4349	412	618										
92.01	7	9	25	0	373	95	133	56	4511	301	574										
92.11	6	15	26	0	395	110	198	147	4572	83	605										

92.21	0	0	23	0	326	135	189	200	4434	372	537										
92.31	20	12	16	0	374	178	156	110	4488	120	590										
92.4	9	0	28	0	319	65	78	174	4662	367	628										
92.51	5	0	39	0	347	154	136	190	4656	248	582										
92.61	7	10	21	25	372	53	230	159	4710	262	602										
92.7	0	8	26	0	379	50	117	156	4777	57	580										
92.8	0	6	32	0	398	116	124	116	4712	179	611										
92.91	21	6	32	0	375	80	237	66	4709	336	638										
93	8	0	36	0	385	99	162	162	4942	214	688										
93.11	8	16	29	0	344	98	161	234	5032	289	676										
93.21	7	0	54	0	428	68	131	252	5030	241	651										
93.31	24	0	46	0	385	71	236	120	5343	216	698										
93.41	0	0	27	0	437	57	126	260	5584	240	719										
93.5	24	6	24	0	421	75	146	195	5731	336	724										
93.61	19	8	24	0	407	52	248	260	5629	327	694										
93.71	12	0	25	0	431	57	102	259	5521	391	729										
93.81	23	22	40	0	447	77	207	257	5517	235	720										
93.91	9	7	38	0	452	141	257	192	5710	268	674										
94.01	31	11	34	0	428	88	229	175	5877	288	726										
94.11	5	5	42	0	344	121	152	221	5703	152	646										
94.21	18	6	20	0	411	61	108	250	5688	355	717										
94.31	19	0	46	0	359	71	179	151	5592	214	659										
94.41	28	26	40	0	412	70	174	175	5813	194	658										
94.5	0	8	57	0	428	90	148	165	5767	197	676										
94.61	7	0	31	0	397	82	108	122	5866	264	657										
94.71	8	0	18	0	424	36	199	138	5815	560	692										
94.81	0	16	25	0	412	134	123	144	5853	290	659										
94.91	6	18	35	0	450	104	133	144	5809	184	685										
95.01	18	0	41	0	434	67	90	121	5783	343	728										
95.11	9	4	63	0	388	111	130	186	5468	302	673										
95.2	15	21	34	0	420	88	194	168	5490	356	659										
95.31	8	15	30	0	410	128	188	115	5506	434	617										
95.41	11	0	31	0	420	106	29	208	5477	305	612										
95.51	0	12	45	0	423	117	191	192	5372	515	707										
95.61	46	8	55	0	439	117	123	289	5184	329	664										
95.71	6	8	36	0	407	115	140	84	5403	559	702										
95.8	9	0	11	0	411	76	150	141	5376	315	742										
95.91	10	0	16	0	399	67	173	243	5410	523	613										
96	0	9	17	0	413	21	128	35	5346	428	591										
96.11	7	21	45	0	417	69	86	168	5472	514	672										
96.2	5	0	22	29	438	91	86	216	5615	441	668										
96.31	8	0	41	0	477	117	244	183	5756	382	651										
96.41	20	10	23	0	413	110	78	171	5590	245	659										
96.51	20	0	34	0	370	84	149	125	5465	277	655										
96.61	6	0	53	6	394	68	92	154	5481	300	645										
96.7	0	5	33	0	451	109	119	192	5299	370	651										
96.81	0	14	31	12	345	98	108	188	5195	188	715										
96.91	14	0	39	0	442	117	199	176	5576	378	671										
97.01	23	14	42	0	401	97	98	222	5480	316	661										
97.11	0	0	35	0	387	121	107	237	5258	156	655										
97.2	0	7	31	0	379	74	107	175	5355	302	648										
97.31	21	23	25	0	456	128	178	232	5418	310	620										
97.4	10	0	33	0	452	176	83	263	5411	280	683										
97.51	20	5	29	0	433	186	130	144	4898	343	652										
97.61	5	6	48	0	385	140	167	224	4978	338	601										
97.7	15	22	15	0	369	86	199	140	4857	191	597										
97.81	5	0	47	0	372	40	217	166	4736	601	658										
97.91	21	7	48	0	342	145	173	184	4803	445	621										
98	0	10	44	0	401	54	103	217	5020	345	614										
98.11	0	0	51	0	326	126	178	112	5109	316	617										
98.21	0	19	33	0	420	76	160	189	4975	441	613										
98.31	0	9	14	0	373	85	133	83	4882	412	607										
98.41	0	0	33	0	412	68	215	222	4796	287	597										

98.51	0	0	34	0	345	0	156	89	4769	423	625
98.61	20	5	20	0	408	87	119	145	4817	395	623
98.71	9	11	19	0	405	118	104	123	4599	265	618
98.81	16	16	53	0	352	95	133	158	4439	261	526
98.91	0	33	46	0	379	131	59	163	4629	293	593
99.01	15	0	31	0	399	154	56	98	4477	271	596
99.11	23	0	32	0	353	121	196	183	4469	279	535
99.21	26	19	48	0	387	56	116	116	4429	477	594
99.31	7	9	50	0	353	115	99	167	4465	219	573
99.41	5	15	39	0	371	45	159	146	4491	387	524
99.51	12	8	39	0	415	125	222	117	4588	325	543
99.61	0	29	37	0	319	92	102	196	4417	490	549
99.71	0	11	34	0	360	120	90	138	4392	398	582
99.81	17	10	24	37	340	132	114	158	4364	383	586
99.91	0	5	33	9	382	88	104	173	4372	270	650
100.01	7	11	19	0	333	162	152	229	4309	285	598
100.11	5	10	55	0	336	63	160	184	4293	204	538
100.21	18	0	49	0	390	156	102	120	3939	490	553
100.31	6	0	37	23	301	109	59	86	3310	326	477
100.41	15	7	8	0	293	169	33	36	2244	486	319
100.51	23	0	38	0	268	199	0	17	1085	668	180
100.61	18	0	14	0	193	239	11	73	346	854	46
100.71	16	15	6	0	119	146	0	0	72	890	32
100.81	0	0	8	0	68	127	0	0	52	928	16
100.91	0	8	18	0	61	91	0	0	54	1005	18
101.01	14	0	0	6	91	103	0	0	19	996	0

Scots pine #3. After extraction in alcohol.

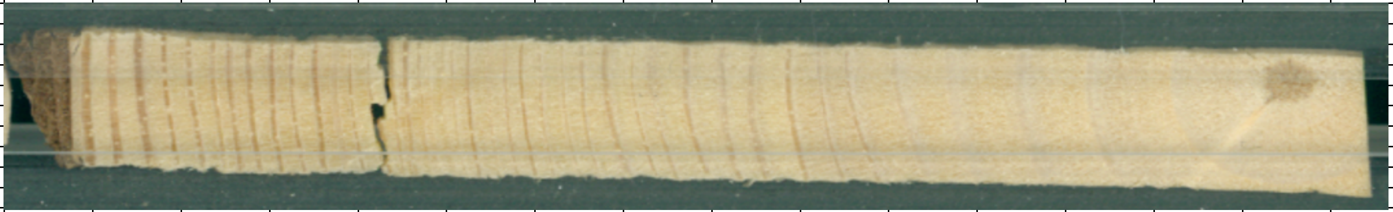

position (mm	Al	P	S	Ti	Mn	Fe	Cu	Zn	Ca	Sr	K
84.41	46	13	0	0	139	16	75	219	234	0	173
84.50	51	6	11	22	145	13	86	272	226	0	182
84.61	56	8	8	0	149	115	161	250	259	0	116
84.70	50	6	22	0	133	52	185	151	233	0	146
84.81	48	23	15	17	160	39	39	167	184	0	132
84.91	17	0	0	0	70	77	205	162	147	0	106
85.01	81	27	0	0	155	83	18	130	197	4	83
85.11	28	0	0	0	151	106	71	165	252	93	94
85.20	29	0	4	7	181	123	130	110	260	247	84
85.31	23	5	0	0	96	143	125	84	237	167	74
85.40	42	0	5	0	176	185	108	257	265	0	106
85.50	60	0	0	9	167	134	136	171	327	5	92
85.61	63	37	0	0	162	124	144	204	313	218	63
85.71	55	15	8	22	151	142	154	149	342	6	125
85.81	61	14	4	0	185	103	73	332	339	0	128
85.91	46	0	0	0	156	81	175	336	289	0	134
86.00	54	0	16	39	154	0	317	297	354	79	135
86.11	42	24	28	18	219	0	161	279	295	0	110
86.21	26	0	10	0	149	32	175	381	273	0	94

86.31	42	0	0	41	101	67	117	220	246	0	98										
86.41	55	35	6	0	141	74	108	231	259	0	68										
86.50	40	0	0	13	128	142	198	118	227	114	69										
86.61	71	0	0	30	158	94	96	219	262	0	91										
86.71	52	19	18	0	95	98	15	224	242	134	78										
86.81	47	0	7	62	172	149	152	176	263	141	100										
86.91	46	18	0	50	108	238	71	126	303	39	92										
87.00	39	0	21	43	153	120	128	217	272	113	70										
87.11	39	0	0	17	120	135	155	138	307	88	104										
87.21	27	0	0	17	175	185	131	193	352	0	127										
87.31	67	22	14	0	175	128	190	281	356	0	111										
87.41	37	39	22	15	175	76	107	262	285	0	100										
87.50	63	9	15	35	135	47	206	369	363	0	80										
87.61	63	0	11	8	138	0	117	217	309	0	140										
87.70	47	0	0	5	135	28	102	280	309	6	92										
87.81	65	30	24	0	196	32	154	218	282	0	115										
87.91	69	39	27	22	132	43	292	194	284	0	96										
88.01	64	12	0	12	145	17	166	182	229	0	91										
88.11	48	17	15	0	179	0	122	141	276	0	94										
88.21	61	0	0	41	134	79	57	227	269	144	76										
88.30	53	0	0	46	155	176	139	161	319	0	62										
88.41	47	0	5	54	158	92	41	82	281	0	95										
88.51	36	0	0	22	154	108	131	38	271	111	76										
88.60	42	6	0	40	129	82	58	154	338	51	93										
88.70	40	19	0	20	139	163	95	167	298	146	80										
88.80	52	9	0	0	175	105	223	84	318	0	67										
88.91	68	9	9	35	179	127	124	128	322	56	87										
89.00	66	18	15	27	170	58	54	351	305	0	114										
89.11	58	25	0	16	163	10	142	314	289	0	75										
89.21	28	26	23	0	221	108	256	262	296	6	80										
89.30	51	29	16	34	205	49	29	220	284	0	103										
89.41	39	11	4	0	177	24	56	206	264	104	78										
89.51	46	30	4	21	175	48	151	258	227	0	86										
89.60	64	31	0	21	167	151	87	151	281	31	59										
89.71	38	13	0	0	212	156	147	110	343	132	50										
89.81	25	15	0	29	202	232	41	175	334	347	47										
89.91	28	0	0	28	186	199	0	169	343	119	87										
90.00	45	5	14	35	217	80	45	141	353	59	74										
90.10	38	9	0	0	182	166	83	194	393	128	55										
90.21	47	13	7	0	219	141	184	192	326	0	81										
90.31	44	0	0	0	207	118	100	184	358	0	103										
90.40	49	0	22	25	168	85	110	198	353	62	94										
90.50	65	5	4	40	198	99	158	260	401	0	120										
90.61	76	6	0	0	178	89	30	285	319	0	132										
90.71	65	32	12	0	130	64	38	264	278	0	88										
90.80	54	0	0	13	141	101	58	220	337	0	63										
90.91	47	19	0	0	214	80	128	290	339	0	95										
91.01	61	0	0	10	185	96	46	249	248	82	67										
91.10	65	0	15	17	173	174	87	197	266	0	62										
91.21	52	0	0	5	174	159	0	161	299	75	81										
91.31	58	16	0	16	173	152	61	42	302	73	57										
91.41	59	11	0	15	154	166	41	101	339	358	83										
91.51	40	0	0	0	194	188	146	167	345	363	78										
91.61	69	13	14	5	184	158	50	152	352	19	78										
91.71	66	19	0	0	188	80	147	241	328	120	77										
91.80	64	22	12	40	227	82	228	282	389	0	103										
91.91	68	0	0	0	200	92	83	199	299	0	73										
92.01	47	30	10	0	127	43	114	352	281	0	72										
92.11	60	14	0	49	153	65	52	305	257	0	67										
92.20	55	47	15	8	151	37	114	237	243	67	38										
92.31	53	28	22	18	129	95	0	186	300	0	85										
92.41	47	24	0	13	235	93	97	231	327	100	95										
92.51	63	8	13	37	177	85	138	110	383	53	66										

92.61	37	20	18	29	209	119	0	87	400	55	101										
92.71	23	17	7	15	232	78	93	181	380	0	85										
92.81	30	7	0	0	235	13	169	318	332	0	93										
92.90	36	33	0	27	209	32	205	296	313	0	64										
93.01	66	16	12	36	187	0	74	337	310	0	63										
93.11	60	18	5	0	182	82	158	249	317	39	106										
93.21	60	7	0	17	232	70	80	212	313	135	67										
93.30	37	12	0	28	229	108	70	212	259	52	65										
93.41	40	0	7	0	180	108	0	207	361	66	65										
93.51	52	0	10	20	180	194	0	184	360	0	57										
93.61	23	28	9	38	231	167	126	171	349	126	63										
93.71	54	10	0	40	215	122	180	291	347	111	81										
93.81	48	12	0	0	231	18	145	201	372	0	90										
93.90	59	24	0	12	254	41	87	299	338	0	83										
94.01	75	23	4	25	215	0	15	378	352	0	79										
94.11	67	32	0	21	230	27	124	218	307	0	100										
94.20	53	0	0	32	191	39	89	190	270	0	55										
94.31	67	0	0	18	168	73	89	211	239	63	70										
94.41	58	0	0	0	206	92	76	303	298	241	77										
94.50	61	20	0	22	191	160	56	140	285	328	57										
94.60	53	0	0	10	190	121	110	45	390	36	85										
94.71	45	11	0	0	107	203	131	189	352	413	87										
94.80	35	0	0	15	189	303	0	134	351	182	63										
94.91	37	13	0	37	210	160	154	122	452	100	72										
95.01	66	15	14	24	242	88	194	224	450	12	87										
95.10	51	17	0	31	182	36	91	277	424	0	113										
95.20	37	10	12	6	243	40	98	281	341	0	51										
95.31	36	6	8	0	191	38	51	353	267	0	68										
95.40	66	19	0	0	192	101	32	331	254	0	43										
95.51	42	11	19	53	167	133	63	277	311	124	58										
95.61	59	0	0	15	138	152	149	101	322	122	79										
95.71	56	0	0	0	214	285	59	43	383	17	62										
95.81	15	0	0	0	142	283	53	119	354	190	95										
95.91	69	0	11	0	149	233	141	122	328	266	62										
96.01	46	0	0	14	249	164	47	62	343	107	54										
96.11	51	0	0	16	196	157	65	43	380	148	95										
96.21	55	9	21	13	203	142	76	248	405	0	84										
96.30	66	8	0	0	160	99	116	266	402	0	70										
96.41	38	7	0	0	246	106	106	219	363	19	68										
96.51	47	18	9	0	230	107	24	328	338	0	72										
96.60	63	36	12	17	174	109	31	186	287	0	79										
96.71	65	18	31	19	216	86	181	275	338	0	80										
96.81	34	0	0	0	180	147	237	213	537	0	61										
96.91	77	16	35	41	201	265	106	73	445	0	60										
97.00	49	0	0	40	150	184	166	209	328	180	53										
97.11	26	0	0	0	151	196	282	78	335	276	55										
97.20	56	30	25	23	204	259	106	257	366	240	62										
97.31	44	0	0	0	219	400	79	139	385	142	77										
97.41	58	15	27	21	237	361	19	237	381	188	76										
97.51	78	8	4	0	236	152	115	327	435	275	85										
97.61	46	0	0	39	200	44	111	285	386	163	70										
97.70	48	39	28	13	292	45	96	357	342	0	56										
97.80	70	0	0	0	188	0	82	307	343	0	55										
97.90	51	0	9	9	224	84	181	307	331	0	46										
98.01	53	16	0	13	189	110	84	377	309	0	58										
98.11	37	12	0	18	166	83	55	316	288	168	105										
98.20	75	14	0	33	189	101	182	265	289	105	82										
98.31	27	0	0	14	144	141	88	185	308	68	41										
98.41	58	0	0	37	255	157	85	72	337	0	30										
98.50	46	13	0	20	202	246	126	177	434	0	67										
98.61	58	21	11	20	248	181	133	220	398	153	59										
98.71	59	16	0	45	219	84	99	212	427	0	51										
98.81	76	26	9	11	195	74	29	188	365	167	35										

98.91	38	17	0	0	200	71	25	264	318	0	100										
99.00	61	0	7	0	191	45	36	440	252	56	53										
99.11	46	7	0	0	204	68	147	278	272	0	66										
99.21	52	32	0	31	208	124	85	163	275	86	52										
99.31	43	8	0	31	209	219	155	142	338	181	53										
99.41	71	0	0	23	178	155	96	123	353	274	38										
99.50	53	0	0	12	226	145	137	177	374	162	29										
99.61	25	0	0	0	239	112	139	169	388	132	87										
99.71	60	21	31	41	155	33	115	228	377	38	99										
99.80	50	25	0	0	230	87	114	220	438	0	86										
99.91	55	6	0	0	218	185	100	191	314	0	53										
100.00	31	15	0	0	218	128	18	223	337	10	80										
100.11	49	7	23	0	197	166	154	213	341	45	49										
100.21	32	9	0	14	104	157	51	165	331	223	85										
100.31	56	0	0	0	157	153	41	229	301	114	39										
100.41	50	12	4	0	182	196	61	234	355	220	61										
100.50	21	0	0	30	193	241	80	128	342	239	65										
100.61	51	0	0	0	126	198	32	58	389	302	63										
100.71	56	0	0	20	187	212	29	124	383	310	59										
100.80	93	0	5	11	178	230	155	156	433	0	76										
100.91	53	0	0	0	159	209	137	68	379	13	53										
101.01	67	0	0	8	132	214	54	175	322	352	56										
101.10	11	0	0	16	185	213	0	28	234	230	57										
101.21	49	0	0	26	147	315	85	0	240	370	28										
101.31	65	0	0	19	153	586	39	126	289	230	32										
101.40	45	0	0	11	183	519	73	159	430	454	64										
101.51	60	0	0	25	187	394	149	123	393	179	62										
101.61	39	0	0	20	223	482	101	185	363	412	70										
101.70	49	0	5	15	118	482	158	32	380	86	91										
101.81	53	0	0	58	234	386	175	18	409	108	76										
101.91	48	11	0	24	144	192	65	98	349	289	77										
102.00	43	10	0	40	165	339	62	154	354	308	61										
102.10	38	27	0	0	194	146	112	254	461	188	81										
102.20	53	0	10	0	159	24	86	245	404	0	76										
102.30	69	21	0	17	158	51	163	285	380	16	90										
102.41	74	13	15	4	169	146	139	288	303	78	82										
102.50	32	0	6	27	171	98	88	241	327	91	57										
102.61	20	0	0	7	122	56	7	76	330	0	64										
102.71	26	8	0	24	153	95	55	168	276	242	57										
102.80	44	22	0	36	168	95	53	152	343	0	75										
102.91	51	23	30	0	171	21	158	265	338	35	81										
103.01	45	20	15	0	126	37	65	178	315	0	97										
103.11	48	21	8	0	166	52	221	278	272	0	58										
103.21	22	0	14	64	136	90	75	188	260	0	43										
103.31	45	0	0	59	172	96	39	97	264	89	89										
103.41	39	0	0	40	194	130	47	224	260	225	64										
103.51	27	6	0	37	172	150	142	156	334	176	56										
103.61	59	10	20	10	189	102	58	204	305	220	72										
103.71	31	0	0	0	206	87	20	243	381	0	48										
103.80	62	27	26	40	169	57	95	242	423	0	85										
103.91	85	12	0	26	191	96	202	296	386	80	91										
104.01	42	0	0	11	99	44	200	230	306	0	60										
104.10	42	0	0	30	181	25	44	285	305	41	85										
104.21	67	9	0	19	125	97	7	151	333	160	71										
104.31	69	11	16	15	206	63	48	172	347	118	60										
104.41	68	12	4	31	183	55	103	235	326	22	84										
104.51	36	0	0	20	200	29	81	175	334	170	85										
104.60	57	17	0	0	205	110	44	258	336	237	41										
104.71	37	0	23	0	162	141	36	13	304	458	58										
104.81	52	0	0	0	178	189	68	84	320	250	88										
104.91	44	0	0	38	166	158	85	119	345	165	69										
105.01	40	0	0	0	141	48	38	263	391	156	75										
105.11	34	0	0	0	250	15	143	246	483	0	90										

105.20	47	0	18	51	175	58	198	196	385	0	65										
105.31	52	0	0	46	245	108	100	212	361	84	59										
105.41	60	0	5	38	200	24	95	247	374	26	57										
105.51	50	20	4	0	158	95	95	204	333	8	51										
105.61	60	24	0	15	169	67	80	226	327	154	100										
105.70	61	6	6	12	148	67	76	368	348	0	101										
105.81	69	15	12	39	162	23	147	123	343	44	72										
105.90	64	0	0	0	168	133	190	282	379	73	76										
106.00	34	0	0	41	219	62	50	222	316	164	94										
106.11	48	0	0	12	192	139	159	174	387	209	51										
106.21	61	27	0	15	166	176	183	45	344	134	82										
106.31	44	11	0	24	209	137	44	179	367	179	79										
106.40	40	5	17	9	172	55	0	206	419	0	60										
106.50	44	30	19	12	186	10	142	333	347	0	104										
106.61	74	21	13	36	181	42	177	253	336	0	53										
106.70	44	5	0	5	187	84	276	313	311	95	72										
106.81	57	5	0	12	153	59	67	232	309	15	75										
106.91	29	0	0	10	141	81	135	153	298	81	63										
107.01	61	0	9	14	190	104	110	81	330	174	50										
107.11	33	0	7	23	158	42	66	144	366	137	55										
107.20	20	0	0	17	179	136	150	113	417	260	66										
107.31	51	0	0	0	166	125	195	96	330	227	59										
107.41	19	0	0	11	178	36	129	101	395	167	67										
107.51	56	0	0	5	237	109	170	224	404	159	96										
107.61	37	0	0	0	190	49	225	169	460	20	69										
107.71	52	7	35	17	193	110	142	301	444	99	58										
107.80	38	28	0	29	200	56	135	232	408	0	77										
107.91	51	0	0	14	172	85	107	158	363	79	52										
108.01	51	0	0	0	192	50	98	141	330	60	71										
108.11	65	0	0	31	182	175	65	135	363	300	70										
108.21	52	7	0	0	175	139	182	107	372	314	64										
108.31	30	0	0	14	193	109	255	100	415	14	20										
108.40	61	0	0	24	189	115	95	199	394	94	41										
108.51	56	10	0	0	227	123	150	296	397	104	66										
108.61	51	26	0	28	264	91	92	318	366	42	111										
108.71	72	27	5	39	190	79	145	258	434	0	64										
108.81	56	12	0	0	147	23	107	228	336	0	49										
108.90	41	35	5	0	196	20	190	230	365	18	52										
109.01	50	4	0	0	198	30	96	161	375	0	56										
109.10	55	16	5	15	185	79	71	139	367	218	51										
109.20	36	7	0	13	189	70	15	136	413	185	41										
109.31	60	0	8	6	205	133	49	122	387	0	82										
109.41	60	0	0	9	186	148	150	224	413	72	46										
109.51	44	5	0	0	211	68	140	84	396	26	62										
109.60	67	35	0	14	237	19	186	284	373	53	63										
109.71	45	0	0	0	249	62	280	392	359	0	71										
109.81	48	10	0	0	206	66	105	251	402	18	112										
109.90	43	15	0	0	230	132	185	281	397	0	61										
110.01	45	9	0	0	165	132	62	71	410	36	67										
110.11	56	7	5	5	165	178	116	111	382	274	73										
110.20	28	0	0	0	142	122	6	150	363	0	77										
110.30	12	0	0	29	202	197	206	209	335	454	63										
110.41	16	0	0	10	208	133	156	207	438	128	70										
110.50	48	25	0	12	259	108	68	201	428	187	62										
110.61	61	0	0	21	217	76	71	279	499	0	103										
110.71	44	33	13	27	219	95	208	300	472	0	83										
110.81	66	17	16	21	221	119	118	317	365	0	81										
110.90	50	8	0	0	202	106	124	269	434	16	79										
111.00	30	0	0	8	147	134	114	151	410	19	76										
111.11	28	0	0	22	212	183	92	146	412	129	37										
111.21	45	10	0	7	141	201	70	60	407	213	46										
111.30	56	0	0	41	255	180	80	17	453	213	52										
111.41	50	10	14	40	184	148	75	58	424	266	67										

111.51	21	8	0	0	162	309	55	142	438	265	77										
111.60	47	0	0	8	209	163	10	155	416	181	74										
111.71	65	26	0	20	220	121	36	222	415	442	70										
111.81	56	0	0	38	252	90	42	218	511	173	75										
111.91	40	9	5	0	200	86	178	220	442	0	28										
112.01	71	19	0	24	177	46	150	311	358	122	88										
112.11	61	42	13	19	224	78	182	218	356	0	56										
112.21	69	0	0	31	214	120	138	212	363	204	61										
112.31	38	0	6	0	167	143	83	167	401	34	79										
112.41	45	0	24	13	204	159	202	186	372	29	56										
112.50	33	0	0	16	212	142	72	205	430	90	72										
112.60	47	6	0	11	156	134	83	164	420	93	78										
112.70	57	0	0	19	164	247	197	92	410	154	92										
112.80	51	0	0	0	224	103	60	93	388	52	82										
112.90	37	0	0	0	182	89	177	238	424	0	87										
113.00	62	0	0	0	218	45	171	228	404	0	63										
113.11	51	31	0	18	182	45	178	200	376	154	67										
113.21	40	7	0	0	209	37	175	291	365	0	93										
113.31	25	0	0	0	239	92	61	189	391	0	63										
113.41	34	0	0	15	174	83	153	283	397	0	48										
113.51	64	22	0	45	163	119	49	194	475	110	89										
113.61	19	0	0	24	244	249	76	261	499	123	94										
113.71	48	0	7	4	165	173	159	189	490	0	75										
113.80	90	7	8	18	165	163	101	222	424	129	52										
113.90	48	0	0	15	224	185	54	114	416	82	65										
114.01	42	0	0	0	196	176	41	147	446	196	74										
114.11	63	0	0	0	194	136	178	35	427	166	47										
114.20	43	0	0	12	146	225	117	196	476	30	77										
114.31	60	23	0	0	203	157	98	280	420	0	70										
114.41	88	19	8	0	256	72	227	276	388	0	55										
114.51	76	35	28	0	196	0	108	246	406	0	60										
114.60	46	6	10	22	234	21	192	116	346	0	55										
114.70	68	24	41	0	202	48	161	234	374	28	53										
114.80	42	0	0	33	208	29	165	275	315	0	67										
114.91	56	11	7	0	225	61	226	148	337	0	62										
115.00	41	0	0	9	166	121	146	193	411	238	53										
115.11	0	4	12	0	159	142	191	211	411	57	58										
115.21	50	0	0	0	243	169	80	88	453	106	44										
115.31	54	0	0	0	186	98	38	123	371	345	95										
115.41	55	16	9	18	168	190	165	261	421	87	61										
115.51	26	0	0	0	216	192	94	161	441	137	54										
115.61	33	0	0	25	278	190	135	104	463	333	75										
115.71	35	0	0	0	219	120	137	139	502	0	53										
115.81	50	6	0	40	279	138	177	159	508	88	113										
115.91	21	0	0	22	248	102	120	222	480	7	59										
116.01	27	0	0	0	260	103	191	171	507	84	76										
116.11	28	19	0	0	230	124	148	249	479	0	59										
116.20	60	19	10	42	160	125	34	175	469	176	53										
116.30	53	16	11	17	206	158	106	226	486	0	78										
116.41	48	16	6	0	195	159	145	225	476	15	72										
116.51	35	0	0	0	189	126	22	151	461	277	63										
116.60	57	37	8	17	222	210	99	224	474	29	81										
116.70	52	6	0	0	243	221	81	134	504	183	63										
116.81	40	11	0	21	285	128	74	167	470	251	74										
116.90	73	0	0	4	173	60	165	153	463	139	48										
117.01	61	22	18	6	201	82	176	195	454	101	100										
117.11	43	0	0	26	203	157	27	159	440	159	82										
117.20	43	0	9	15	184	114	131	220	410	0	91										
117.31	55	0	19	0	216	147	109	148	508	0	87										
117.40	67	0	0	32	206	149	72	173	458	153	70										
117.50	74	15	24	0	174	194	87	128	454	0	62										
117.61	38	12	0	18	257	167	196	140	446	26	52										
117.70	86	18	21	11	231	122	135	232	507	46	59										

117.81	42	0	0	0	188	192	107	184	475	80	40										
117.91	52	16	0	24	142	186	93	185	427	334	48										
118.00	52	22	0	6	204	203	124	171	424	290	76										
118.10	37	4	0	33	128	207	36	148	445	216	42										
118.20	35	0	0	0	225	158	92	109	446	47	75										
118.31	41	0	0	0	258	256	97	56	541	113	35										
118.41	32	10	0	18	223	210	171	97	432	283	60										
118.50	49	10	4	0	236	183	61	256	453	202	70										
118.61	43	0	0	0	221	190	119	206	471	312	53										
118.71	77	26	29	34	183	120	0	186	534	53	55										
118.81	41	0	0	22	181	86	179	380	496	64	70										
118.90	47	23	11	11	237	113	137	262	440	8	60										
119.01	84	0	20	34	204	96	152	222	442	0	89										
119.11	62	0	0	0	216	57	223	255	450	0	53										
119.20	69	0	11	21	192	67	117	101	416	0	53										
119.31	34	13	0	21	205	29	158	213	360	0	54										
119.41	60	21	10	14	173	54	139	232	367	15	88										
119.50	68	11	0	38	229	75	98	253	365	6	56										
119.61	62	12	6	0	209	53	41	129	410	70	50										
119.70	67	26	0	0	184	85	83	182	413	203	74										
119.81	42	5	0	0	213	82	51	175	448	186	39										
119.91	53	0	0	7	187	136	83	166	500	59	84										
120.01	63	18	0	18	235	137	54	91	459	134	56										
120.10	54	22	0	6	206	96	44	39	444	427	87										
120.20	19	0	0	0	200	88	202	167	486	232	63										
120.31	52	0	0	21	205	176	106	168	407	340	75										
120.41	40	0	0	23	195	100	83	87	450	170	52										
120.51	41	4	0	13	205	65	0	165	457	91	46										
120.60	41	0	11	0	195	131	112	171	476	107	104										
120.71	54	0	0	0	255	122	172	311	491	0	80										
120.81	65	0	0	0	226	14	173	340	473	0	90										
120.91	48	15	0	0	205	53	150	299	433	0	51										
121.01	56	6	8	14	267	6	173	323	434	0	75										
121.11	44	13	6	22	220	57	91	211	434	26	68										
121.20	56	25	9	9	197	60	124	179	416	0	94										
121.31	51	12	0	15	233	49	93	293	420	19	54										
121.41	42	0	0	12	185	38	116	196	427	0	70										
121.51	34	0	0	9	231	60	127	187	443	205	73										
121.61	36	0	0	0	167	116	83	69	407	200	68										
121.70	69	0	0	53	255	100	18	204	443	140	37										
121.81	29	8	0	29	227	148	51	136	473	0	76										
121.90	28	0	0	6	243	119	0	143	432	169	65										
122.01	79	0	0	0	193	126	137	183	437	212	76										
122.11	30	0	0	17	158	173	99	220	449	150	70										
122.20	43	0	0	14	239	103	33	172	474	145	68										
122.31	76	15	0	8	222	127	46	212	553	254	91										
122.41	49	0	7	13	295	139	35	355	555	0	101										
122.50	62	6	0	17	333	26	186	270	506	111	76										
122.61	53	10	10	0	274	52	227	194	508	0	90										
122.71	60	23	16	0	220	75	177	262	434	11	75										
122.81	34	6	0	0	228	450	0	279	410	0	54										
122.90	35	0	0	0	225	125	168	242	390	0	70										
123.00	91	9	15	40	203	86	128	92	392	192	70										
123.10	41	9	0	24	164	118	58	133	501	132	54										
123.21	59	0	7	0	198	137	110	188	507	128	83										
123.31	38	7	0	0	203	174	58	58	541	140	66										
123.40	54	0	0	0	225	358	10	264	500	357	82										
123.50	61	0	0	35	229	320	166	159	500	167	46										
123.61	41	0	9	21	204	119	147	138	488	108	89										
123.71	26	0	0	0	236	119	61	177	459	105	79										
123.80	18	0	0	39	219	112	25	207	388	355	62										
123.91	65	5	0	6	218	109	41	56	441	151	116										
124.00	45	0	9	0	253	145	121	185	491	255	57										

124.11	59	9	0	31	218	98	93	208	461	198	69										
124.21	59	5	0	0	244	119	0	11	493	246	70										
124.31	68	0	0	0	179	113	28	112	532	169	113										
124.40	36	28	7	14	193	99	173	143	504	74	67										
124.51	51	0	14	0	241	120	30	154	484	0	78										
124.61	46	0	16	22	237	62	128	331	445	0	76										
124.71	49	8	6	0	277	8	128	249	497	45	83										
124.80	43	27	0	0	214	25	70	259	448	30	57										
124.90	69	8	0	0	210	32	118	411	447	0	76										
125.01	72	5	0	0	212	54	150	441	356	0	34										
125.10	61	5	16	23	184	0	186	178	317	0	46										
125.21	38	0	0	47	181	28	212	151	308	0	53										
125.31	55	14	0	0	191	74	10	227	357	57	51										
125.40	61	8	0	0	213	131	70	251	377	228	31										
125.51	39	6	0	0	224	109	41	201	439	123	32										
125.61	51	17	0	10	159	119	62	241	384	224	66										
125.71	46	9	0	36	233	102	48	149	440	218	56										
125.80	50	14	0	28	217	113	115	173	411	232	64										
125.91	29	6	9	0	208	118	37	97	470	133	80										
126.01	43	0	0	36	168	107	107	149	533	0	48										
126.10	75	11	0	31	236	134	124	113	463	204	78										
126.21	30	13	5	0	188	77	51	152	481	248	64										
126.31	50	0	0	23	193	42	85	89	509	45	88										
126.40	42	0	0	0	267	199	181	146	503	61	74										
126.51	41	0	0	23	211	91	75	188	515	6	60										
126.61	58	20	11	6	236	66	249	307	549	0	79										
126.71	50	0	0	0	248	111	166	350	533	0	69										
126.81	84	8	17	9	247	88	124	219	504	0	67										
126.90	44	0	5	12	223	111	65	350	434	26	74										
127.01	48	10	10	39	165	74	105	235	438	0	59										
127.11	63	0	0	53	244	74	105	263	420	103	62										
127.20	45	0	5	22	200	94	56	200	440	179	41										
127.30	42	16	7	0	180	46	95	211	480	83	71										
127.41	16	0	0	0	187	94	116	196	471	122	73										
127.51	43	0	0	0	192	59	32	110	473	120	39										
127.61	67	0	0	22	254	87	120	98	455	84	57										
127.71	52	0	0	0	229	34	79	90	483	0	58										
127.80	21	0	0	0	222	132	52	85	513	93	77										
127.91	46	0	0	21	244	89	76	74	431	144	49										
128.01	30	0	0	38	211	58	161	69	482	78	88										
128.10	42	0	0	48	171	116	31	123	478	83	88										
128.21	69	21	10	8	177	49	154	150	509	153	58										
128.31	59	28	35	8	278	72	160	183	490	112	72										
128.41	61	0	14	0	237	83	153	233	490	14	92										
128.51	54	24	0	0	252	108	245	268	464	0	42										
128.60	21	0	0	13	232	65	34	169	461	30	46										
128.71	80	0	0	10	201	66	37	255	434	0	49										
128.81	63	7	8	0	222	29	142	186	462	192	79										
128.90	47	38	0	0	207	72	45	200	422	152	95										
129.01	62	0	6	5	181	121	168	280	409	298	64										
129.11	35	0	0	9	223	37	139	184	453	0	77										
129.21	63	29	16	21	222	98	69	221	475	50	40										
129.31	67	0	0	0	224	93	55	206	393	210	51										
129.41	52	0	0	18	236	59	165	151	449	10	34										
129.51	76	0	0	0	183	110	148	99	437	0	44										
129.61	25	0	0	27	216	119	89	176	448	268	54										
129.71	57	0	0	27	208	103	117	166	513	265	52										
129.81	44	10	0	0	213	94	0	108	481	327	60										
129.91	47	0	11	20	224	112	198	59	406	104	39										
130.01	71	0	0	11	217	78	63	9	465	103	51										
130.10	35	0	0	0	252	57	98	191	454	142	68										
130.20	75	9	0	57	182	100	30	114	452	168	73										
130.31	57	11	9	47	196	129	59	203	511	38	66										

130.41	66	8	0	15	230	144	162	204	544	36	74										
130.50	36	0	0	0	229	98	83	174	485	114	60										
130.61	62	0	7	19	204	82	76	170	451	194	62										
130.71	52	0	0	25	266	79	95	96	536	109	95										
130.80	52	16	10	12	226	38	107	253	532	21	69										
130.91	30	0	0	17	288	47	109	239	483	0	90										
131.01	63	35	11	28	251	45	123	200	562	0	50										
131.11	48	0	0	0	199	90	144	270	499	57	80										
131.21	82	22	0	4	207	41	59	103	476	0	64										
131.30	78	0	0	0	189	67	131	160	516	0	56										
131.40	74	0	0	25	189	10	148	249	466	85	95										
131.51	51	0	9	10	238	45	177	266	460	0	45										
131.60	42	0	0	4	191	61	99	140	420	104	85										
131.70	65	9	0	9	203	50	219	281	414	0	60										
131.80	45	0	14	0	220	60	197	341	422	0	34										
131.90	41	18	0	0	235	41	231	117	446	0	56										
132.00	54	28	8	0	269	29	91	129	466	92	72										
132.11	50	0	0	9	228	82	100	137	470	109	59										
132.21	38	16	12	6	251	109	90	260	477	166	58										
132.31	42	4	0	43	251	104	54	61	519	0	73										
132.41	64	0	5	0	260	112	142	126	501	70	61										
132.50	84	0	0	8	259	65	171	209	523	102	60										
132.61	36	16	0	0	227	39	105	114	473	163	85										
132.71	55	0	0	25	215	175	130	206	557	108	67										
132.80	58	0	0	0	224	119	37	128	537	143	79										
132.91	67	10	22	0	176	107	53	148	570	297	57										
133.00	64	10	9	0	252	92	162	70	576	251	66										
133.11	55	0	0	12	209	84	182	128	538	64	95										
133.21	24	0	30	0	265	51	122	91	566	37	46										
133.31	63	0	0	35	247	147	0	148	536	250	50										
133.40	37	18	12	8	258	68	73	121	543	111	67										
133.51	36	5	0	0	239	101	144	190	556	8	83										
133.61	55	0	9	51	253	171	152	136	533	217	95										
133.71	76	17	8	15	257	60	86	229	598	146	100										
133.81	59	0	0	0	260	55	226	155	595	94	61										
133.91	64	16	18	12	243	103	60	177	643	98	91										
134.01	45	0	0	34	271	52	63	200	572	27	86										
134.11	57	0	0	0	246	67	167	367	527	0	92										
134.21	54	0	0	7	218	49	90	151	497	0	71										
134.31	73	26	40	0	257	69	178	236	501	0	61										
134.41	44	22	9	34	226	48	215	256	521	0	82										
134.50	60	7	25	0	247	71	105	234	504	0	102										
134.61	72	12	0	26	190	16	135	205	488	0	85										
134.71	49	9	0	0	204	29	169	201	495	0	60										
134.80	39	0	7	0	231	73	205	209	510	0	84										
134.91	42	20	0	32	250	78	135	213	479	159	72										
135.01	61	22	4	0	247	40	84	90	436	119	92										
135.10	49	0	0	23	216	80	118	255	475	198	66										
135.21	44	0	9	24	195	45	27	90	462	238	89										
135.30	61	13	0	19	281	61	112	134	462	176	65										
135.41	27	12	0	19	265	113	93	155	551	35	90										
135.51	46	22	13	0	318	81	119	195	562	32	85										
135.60	38	7	0	11	200	83	123	210	507	44	87										
135.71	56	0	0	17	243	143	170	139	582	104	72										
135.80	48	5	0	11	271	73	127	203	532	91	45										
135.90	56	10	0	48	239	73	169	122	513	231	63										
136.01	36	16	0	28	249	86	94	193	508	322	35										
136.11	52	7	0	44	222	63	71	172	516	239	52										
136.21	19	6	0	27	196	118	30	70	578	225	81										
136.30	61	0	0	0	236	78	154	116	525	0	59										
136.41	26	0	0	13	242	79	189	161	570	152	53										
136.51	32	0	0	14	255	99	136	92	486	234	59										
136.60	40	0	0	16	227	81	122	14	506	279	71										

136.71	70	15	11	12	253	75	129	66	581	50	66										
136.80	71	8	0	10	229	98	160	178	591	251	54										
136.91	45	0	0	24	255	68	57	76	611	146	94										
137.01	32	0	0	51	253	45	139	149	572	307	98										
137.10	58	0	0	24	231	65	63	184	540	45	86										
137.21	6	0	0	23	293	94	125	157	571	158	78										
137.31	66	0	0	18	226	73	171	121	631	70	113										
137.40	56	0	0	7	243	99	96	196	560	406	84										
137.51	44	0	0	26	296	33	96	154	556	241	72										
137.61	47	25	0	0	206	86	61	158	581	197	74										
137.71	71	0	4	0	234	37	143	180	611	89	69										
137.81	50	0	0	0	229	26	241	260	641	64	54										
137.90	60	22	5	30	301	10	139	256	561	0	50										
138.00	66	13	12	0	270	70	191	194	573	0	78										
138.11	67	25	17	4	211	54	204	249	494	0	52										
138.21	60	12	0	19	230	24	224	252	491	0	76										
138.30	61	17	10	0	207	25	172	366	459	0	68										
138.41	54	4	9	36	211	21	70	202	434	0	98										
138.51	44	26	4	0	217	64	126	287	502	182	94										
138.60	64	19	34	25	225	39	25	233	561	0	78										
138.71	73	17	0	29	245	0	119	136	591	38	90										
138.81	34	36	7	17	316	47	0	229	591	163	63										
138.90	72	15	8	0	239	42	25	155	531	0	48										
139.01	60	0	0	21	246	80	150	126	524	166	59										
139.11	52	12	0	30	295	79	108	79	512	237	65										
139.20	36	20	0	14	295	51	168	172	580	134	82										
139.30	40	6	5	0	198	5	89	129	597	222	88										
139.41	76	0	0	0	265	97	120	115	559	277	109										
139.50	41	0	0	30	235	58	124	105	572	219	84										
139.60	59	0	0	0	231	117	131	97	616	125	98										
139.71	46	10	0	20	203	54	0	124	617	201	76										
139.81	56	10	0	36	236	52	73	64	627	370	65										
139.91	52	9	0	6	279	92	59	210	580	154	55										
140.01	48	19	0	43	283	85	141	238	537	201	101										
140.10	48	17	15	4	282	85	108	92	595	117	64										
140.20	54	0	0	0	244	72	116	34	593	47	65										
140.31	63	0	0	25	245	123	83	86	611	96	56										
140.41	46	0	0	9	281	123	125	97	582	192	95										
140.50	46	0	4	0	239	129	92	51	676	126	86										
140.61	36	0	0	0	289	214	49	76	636	222	94										
140.70	70	0	8	0	243	158	114	171	632	184	96										
140.81	80	27	0	23	267	148	109	18	598	219	95										
140.91	22	0	0	14	308	117	98	121	585	177	53										
141.01	61	0	0	41	269	137	203	207	642	289	92										
141.11	57	0	9	13	270	119	164	244	616	280	103										
141.20	59	0	0	0	210	95	16	80	678	281	63										
141.31	37	20	0	16	294	119	96	145	619	203	96										
141.40	51	0	0	0	306	39	146	238	674	218	88										
141.51	67	0	5	26	266	116	124	323	688	235	109										
141.60	25	0	5	0	295	54	208	299	732	249	100										
141.70	49	0	0	0	301	78	114	223	717	88	80										
141.81	61	11	23	11	372	104	163	302	801	66	77										
141.90	60	31	7	7	336	8	143	305	788	0	113										
142.00	42	0	5	0	319	68	208	260	729	0	89										
142.11	60	27	5	31	342	64	165	340	756	0	107										
142.21	68	21	5	22	286	27	221	195	711	77	64										
142.31	51	11	0	6	283	56	145	354	616	133	81										
142.41	43	0	5	23	345	80	168	108	690	0	71										
142.51	79	46	13	20	308	64	134	184	614	134	101										
142.61	47	17	5	58	277	23	141	248	561	56	106										
142.71	56	10	0	21	266	45	179	138	590	143	90										
142.81	64	10	0	13	271	84	135	241	573	0	74										
142.91	69	16	0	11	277	104	129	187	594	0	65										

143.00	51	0	0	12	298	51	194	187	613	133	89										
143.11	47	0	15	23	281	103	71	187	618	0	105										
143.20	58	0	0	31	350	126	81	194	532	10	74										
143.30	73	7	17	0	209	40	111	198	575	40	86										
143.40	51	7	0	43	302	97	189	145	607	199	97										
143.51	61	32	7	14	219	95	196	170	559	145	73										
143.61	34	18	20	23	239	78	0	179	652	44	76										
143.70	70	7	7	21	295	41	117	83	646	170	82										
143.81	67	0	8	38	283	58	234	261	615	71	76										
143.90	34	0	0	26	260	55	60	98	552	297	57										
144.01	76	26	0	22	260	117	97	176	581	196	85										
144.11	58	0	0	13	244	135	174	165	610	100	74										
144.20	26	0	0	6	318	148	150	180	630	42	96										
144.31	45	0	0	0	235	142	115	307	629	140	102										
144.41	24	0	0	24	285	76	161	149	649	21	87										
144.51	32	0	0	11	257	87	105	192	635	0	80										
144.61	41	22	0	0	310	96	185	184	653	156	107										
144.70	56	5	18	44	290	72	137	270	593	154	90										
144.81	63	6	0	0	274	73	95	124	655	68	81										
144.91	42	0	13	37	309	108	105	135	625	278	111										
145.01	53	0	0	6	309	68	156	28	654	195	88										
145.11	62	0	4	7	234	67	201	88	667	112	113										
145.21	47	11	0	17	380	84	117	227	657	58	83										
145.31	60	6	0	9	283	46	88	95	623	196	88										
145.41	56	19	7	16	271	31	182	134	657	155	85										
145.51	37	0	0	0	274	62	134	104	656	46	105										
145.60	51	14	0	0	209	52	10	184	690	223	88										
145.71	68	19	0	16	251	130	68	48	695	100	89										
145.80	36	11	0	0	262	95	242	204	684	272	97										
145.91	56	18	19	24	360	155	171	157	749	55	81										
146.00	54	0	0	13	285	81	127	143	770	341	96										
146.10	48	11	10	0	292	107	104	207	679	222	84										
146.21	24	0	0	4	360	148	132	241	724	161	170										
146.31	45	0	0	12	290	77	116	124	735	13	90										
146.40	52	0	9	20	355	76	143	166	714	159	114										
146.51	64	21	7	7	272	26	147	128	750	199	112										
146.61	55	16	0	0	298	98	125	237	797	0	130										
146.70	56	0	0	18	361	141	149	213	875	116	143										
146.81	44	0	0	20	371	129	113	179	803	123	113										
146.90	28	22	0	28	308	75	317	258	758	83	122										
147.00	47	0	5	45	318	45	105	285	772	0	105										
147.11	67	13	0	16	319	84	147	232	721	29	83										
147.21	62	0	19	11	325	49	200	211	640	93	88										
147.31	58	11	18	0	310	117	78	140	697	0	118										
147.41	38	0	4	10	323	94	177	251	675	48	81										
147.50	56	0	0	21	291	88	259	228	732	197	128										
147.60	52	0	0	30	315	79	163	207	691	100	118										
147.71	43	0	0	0	328	56	106	187	659	66	90										
147.81	62	25	9	0	255	39	134	231	645	0	101										
147.91	25	24	0	0	325	50	261	165	669	116	85										
148.01	37	0	0	33	315	66	209	134	735	102	95										
148.10	46	27	7	38	339	66	196	157	662	159	82										
148.20	72	0	0	0	286	59	230	279	734	95	97										
148.31	58	0	0	0	233	78	273	252	633	175	96										
148.41	41	15	0	0	331	90	207	209	619	40	98										
148.51	40	0	0	0	256	106	147	185	618	0	99										
148.61	58	0	16	0	353	98	113	162	637	81	66										
148.71	62	7	0	24	282	57	46	234	651	212	89										
148.80	38	0	0	0	298	111	117	208	643	357	68										
148.90	73	0	6	48	319	60	156	217	603	62	83										
149.00	31	0	0	34	273	104	211	166	682	14	124										
149.10	47	0	0	30	326	42	147	208	705	204	104										
149.21	40	35	7	7	301	56	87	175	686	95	93										

149.31	44	16	0	31	318	87	103	244	690	151	75										
149.41	45	0	0	72	281	83	223	123	666	105	73										
149.51	61	12	0	0	262	109	212	117	668	25	84										
149.61	46	0	0	0	281	133	192	251	645	166	76										
149.71	63	18	0	28	290	70	147	205	711	73	102										
149.81	40	9	11	12	348	129	195	227	715	214	96										
149.91	59	11	0	0	340	88	112	201	708	0	66										
150.00	61	14	10	61	345	134	168	127	797	0	97										
150.11	44	0	0	21	320	34	304	190	763	7	120										
150.21	72	0	0	0	365	201	70	270	783	0	82										
150.31	50	5	0	0	348	113	206	307	818	0	124										
150.40	65	0	0	9	369	121	289	285	888	310	150										
150.50	59	25	16	34	324	78	75	250	829	82	135										
150.60	71	0	0	33	333	110	196	229	833	205	105										
150.71	54	0	7	15	371	95	116	179	811	46	125										
150.81	56	16	0	25	370	0	133	206	868	0	104										
150.90	66	0	13	0	394	85	171	279	939	44	138										
151.00	38	6	7	10	443	60	157	299	932	218	120										
151.11	72	21	17	0	377	26	132	130	907	237	156										
151.21	59	10	7	10	399	146	151	205	977	0	135										
151.30	73	21	8	0	444	88	150	262	1042	0	121										
151.41	88	21	10	0	398	25	165	302	1031	100	176										
151.51	55	37	20	16	391	81	180	230	932	17	114										
151.60	87	0	0	6	435	106	154	112	966	20	150										
151.71	73	24	7	19	434	81	163	285	1031	176	146										
151.81	67	8	0	0	398	111	134	216	991	111	151										
151.90	52	0	0	50	408	101	155	311	992	49	134										
152.00	53	30	0	49	467	87	46	147	986	205	133										
152.11	42	16	9	0	383	96	171	229	1056	0	129										
152.21	34	19	7	20	438	97	174	268	1112	17	111										
152.30	54	0	8	0	467	101	25	247	1098	155	151										
152.41	86	23	21	37	432	80	200	140	1066	0	130										
152.51	45	12	0	11	400	96	232	297	1079	100	143										
152.61	56	7	0	4	453	97	107	210	1074	255	148										
152.70	68	4	0	12	448	120	162	159	1123	139	127										
152.81	17	13	0	44	395	63	158	201	1061	28	134										
152.90	65	0	0	18	366	80	162	123	1026	107	127										
153.00	66	44	5	0	412	100	157	207	1154	339	116										
153.11	59	7	8	0	438	81	219	139	1145	29	125										
153.21	24	0	0	41	375	137	174	304	1008	284	172										
153.31	67	21	13	13	419	57	142	215	968	0	133										
153.41	38	8	4	0	312	147	48	206	937	261	106										
153.50	61	38	9	37	436	129	190	108	1002	89	128										
153.60	41	6	0	0	396	130	65	200	962	240	123										
153.71	42	0	0	17	405	78	109	87	1008	112	94										
153.81	56	0	0	0	368	72	194	134	973	196	133										
153.91	34	0	0	26	377	82	111	61	961	164	108										
154.01	56	11	23	0	410	79	259	138	989	218	100										
154.11	62	0	0	30	380	27	105	165	1056	251	115										
154.20	63	9	0	23	456	126	145	182	954	216	143										
154.30	33	0	0	0	402	53	196	157	1063	71	156										
154.41	49	21	4	0	413	47	27	145	1014	0	152										
154.51	21	8	0	0	418	41	211	205	1015	214	143										
154.60	30	0	0	20	434	60	110	159	1014	117	129										
154.71	56	18	9	19	472	66	116	185	1011	154	141										
154.80	35	15	24	17	449	44	182	238	1051	234	132										
154.91	60	24	34	19	429	67	155	186	1031	142	139										
155.00	22	0	9	20	375	68	108	266	1094	0	141										
155.11	51	0	0	0	431	148	225	226	1236	100	106										
155.21	56	26	0	0	359	73	135	255	969	0	142										
155.30	70	5	15	40	392	123	96	207	959	0	141										
155.41	68	0	0	0	415	115	228	277	896	0	107										
155.51	42	0	7	21	418	40	250	171	871	170	108										

155.61	51	0	0	12	376	100	147	306	825	132	128										
155.71	29	0	0	4	355	135	108	202	813	0	114										
155.80	73	0	7	0	389	108	178	209	961	36	146										
155.91	29	0	8	0	405	97	193	256	1023	150	94										
156.01	71	18	12	36	447	71	146	227	984	163	122										
156.10	49	6	18	36	398	114	134	135	905	0	112										
156.20	60	0	0	0	326	144	30	94	884	108	104										
156.31	80	18	16	0	338	149	206	153	892	145	122										
156.41	43	5	12	22	340	126	54	168	786	115	124										
156.51	47	11	0	10	383	90	151	151	815	109	95										
156.61	37	11	0	0	313	80	217	208	828	20	144										
156.70	39	40	10	0	327	93	247	101	805	11	122										
156.80	63	0	0	0	357	102	148	169	799	229	110										
156.91	41	0	0	7	372	117	154	228	774	117	100										
157.01	30	20	10	5	338	147	132	209	786	88	108										
157.11	65	27	0	32	326	31	96	132	753	96	101										
157.21	40	0	0	0	284	53	142	161	742	87	121										
157.31	65	11	15	6	348	58	236	164	735	0	107										
157.41	45	7	13	9	345	99	132	125	738	32	91										
157.50	68	0	0	25	420	118	154	111	759	24	85										
157.60	42	0	0	15	366	115	281	290	725	130	119										
157.71	48	35	15	4	298	60	182	257	707	0	82										
157.81	49	0	0	0	272	142	229	243	710	27	95										
157.90	44	22	0	21	331	193	142	130	711	94	121										
158.00	60	25	20	27	260	333	78	0	592	157	90										
158.10	32	0	0	0	193	289	69	0	308	357	57										
158.20	15	0	0	32	105	149	37	0	62	447	9										
158.31	32	0	0	32	75	67	0	0	24	473	11										
158.41	15	0	0	39	77	72	6	0	12	281	0										
158.51	67	0	0	6	52	100	0	0	32	667	0										
158.61	0	0	0	27	76	77	45	0	44	502	0										
158.70	30	0	0	14	46	28	0	0	43	655	9										
158.80	34	0	0	21	64	75	0	0	38	634	0										
158.90	46	0	0	16	67	89	32	0	57	509	17										
159.01	0	0	0	21	44	82	52	11	20	582	8										
159.11	32	0	0	0	33	92	0	0	44	657	0										
159.21	12	0	0	19	72	89	0	0	61	530	0										
159.31	35	0	0	25	76	35	0	0	36	320	0										
159.40	14	0	0	9	79	68	26	0	24	524	22										
159.51	25	0	0	11	82	72	0	0	50	585	0										
159.61	12	0	0	6	52	65	0	0	27	620	6										
159.71	0	0	0	45	69	73	0	0	21	513	26										
159.81	21	0	0	15	94	335	15	0	229	591	37										
159.91	42	0	45	40	252	1672	110	263	1867	489	473										
160.01	64	18	95	47	598	1917	204	752	4071	168	952										
160.11	69	8	74	62	725	1014	108	953	4025	78	672										
160.20	65	15	31	0	819	776	184	911	3692	98	538										
160.31	62	17	17	39	845	339	111	1105	2743	61	460										
160.41	41	23	0	16	772	101	189	987	2042	34	395										
160.50	51	11	8	33	666	69	155	850	1748	0	335										
160.60	31	23	6	15	672	65	103	808	1710	0	386										
160.71	36	5	18	20	582	106	161	713	1503	0	336										
160.81	33	11	13	0	483	77	130	639	1372	11	323										
160.91	65	0	0	22	493	114	94	592	1416	22	386										
161.01	59	20	13	24	486	117	159	640	1733	16	496										
161.10	54	21	26	10	646	157	188	835	2134	0	764										
161.20	58	14	47	28	746	223	170	907	2143	172	943										
161.31	48	17	40	8	784	183	116	905	1932	41	838										
161.41	49	26	11	41	877	184	92	1185	2027	82	834										
161.51	25	6	25	25	890	252	145	1052	1896	121	1000										
161.61	41	12	43	0	878	163	104	935	1975	219	1039										
161.70	40	0	8	0	851	197	86	1092	1894	92	1036										
161.81	53	14	24	8	874	235	180	1266	1781	313	1167										

161.90	50	10	22	0	664	190	171	1056	1285	0	949
162.01	75	9	23	0	546	203	12	650	728	80	571
162.11	38	0	10	29	217	154	167	379	491	252	359
162.21	37	7	9	17	221	161	96	381	254	58	219

Scots pine #3. After extraction in HCl.

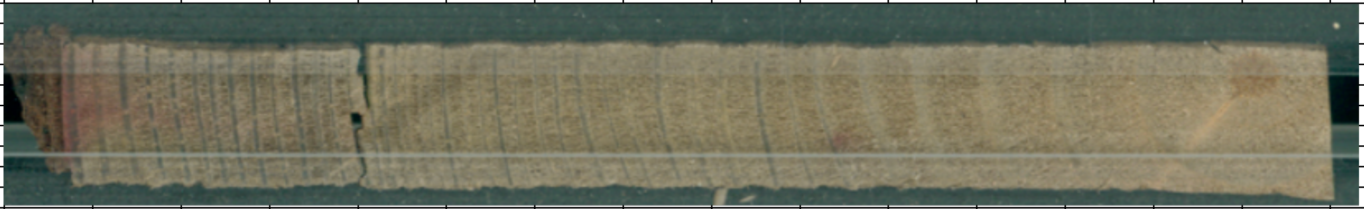

position (mm	Al	P	S	Ti	Mn	Fe	Cu	Zn	Ca	Sr	K
85.91	60	0	0	59	74	67	29	0	21	100	0
86.00	24	14	4	30	124	23	0	0	53	211	10
86.11	48	0	0	18	96	72	32	0	43	284	8
86.21	23	0	0	7	92	76	32	42	33	419	16
86.31	28	0	0	31	115	92	135	30	31	397	0
86.41	27	0	0	19	69	51	48	0	29	146	15
86.50	46	0	0	16	98	163	152	0	19	187	19
86.60	35	0	0	0	98	336	0	8	35	222	16
86.71	70	0	0	23	60	213	94	69	43	257	12
86.80	63	0	0	25	68	28	104	95	52	60	5
86.91	35	0	0	0	29	39	112	222	33	157	13
87.01	46	0	0	0	85	44	11	105	27	0	0
87.10	64	0	0	0	50	0	65	125	44	29	16
87.21	24	0	0	15	92	66	9	135	12	0	0
87.30	50	12	0	8	84	47	108	0	14	137	13
87.40	54	0	0	50	65	69	42	112	20	138	0
87.51	18	0	0	14	100	67	91	62	0	34	5
87.60	38	0	0	14	130	28	69	108	44	373	17
87.71	30	7	0	0	78	103	77	12	24	288	33
87.81	49	0	0	10	96	87	79	131	18	406	25
87.90	41	6	0	0	120	105	95	42	35	123	7
88.01	42	0	0	12	70	31	103	61	48	140	0
88.10	30	0	0	0	59	29	0	84	37	282	17
88.21	50	9	0	16	66	23	98	63	14	54	0
88.30	16	0	0	11	103	26	109	141	33	0	23
88.40	45	0	0	7	79	40	61	128	27	0	0
88.51	41	0	0	0	49	0	0	154	29	171	7
88.61	57	0	0	0	51	53	161	67	23	9	0
88.70	32	0	0	39	27	71	112	147	28	0	0
88.81	32	0	0	0	58	35	106	56	49	186	6
88.90	48	0	0	9	74	24	0	105	54	233	0
89.00	74	0	0	21	54	35	53	31	22	183	0
89.11	24	0	0	30	90	22	0	91	0	149	17
89.21	46	0	0	0	88	71	69	119	0	295	23
89.31	22	0	0	0	130	81	60	56	30	258	41
89.41	24	0	0	30	104	91	106	61	24	218	20
89.50	56	12	0	23	72	78	30	81	16	253	9
89.61	23	0	0	30	84	47	28	76	14	54	0
89.71	51	0	0	10	91	54	51	61	33	96	8
89.80	52	0	0	0	72	36	0	0	60	66	11
89.91	38	0	0	0	84	41	83	162	63	48	0

90.00	61	0	0	53	94	0	100	47	52	0	0										
90.11	49	9	0	15	88	8	19	53	28	36	0										
90.21	37	0	0	30	63	58	27	35	47	0	0										
90.30	69	13	0	22	38	44	103	15	17	27	15										
90.41	53	0	0	35	93	53	0	8	38	343	8										
90.51	21	0	0	39	100	88	20	8	45	77	0										
90.60	6	0	0	0	93	111	46	58	24	269	6										
90.71	20	0	0	53	126	92	0	0	38	244	0										
90.81	50	0	0	15	104	33	73	98	14	309	16										
90.91	39	0	0	0	70	135	46	30	32	186	0										
91.01	25	0	0	12	158	70	0	23	23	293	0										
91.10	49	0	0	5	67	99	62	56	25	85	23										
91.21	30	15	0	0	93	0	0	56	28	0	21										
91.30	57	18	0	0	92	30	133	121	42	255	0										
91.41	64	0	0	6	74	12	90	36	35	77	0										
91.51	49	0	0	0	52	26	65	110	41	266	18										
91.61	46	0	0	0	71	114	79	45	47	53	7										
91.71	47	0	0	0	133	62	0	72	19	187	18										
91.81	46	0	0	10	75	68	0	0	29	214	29										
91.91	26	0	0	24	90	72	16	41	23	383	11										
92.01	14	0	0	0	84	54	112	43	28	417	7										
92.10	47	0	0	30	72	24	20	21	30	331	4										
92.21	35	0	0	0	83	76	171	5	34	382	0										
92.30	46	0	0	52	76	81	21	32	5	197	26										
92.41	35	0	0	0	88	15	0	97	18	229	0										
92.50	63	0	0	0	117	80	66	49	55	108	7										
92.60	69	17	6	10	84	94	136	0	36	145	30										
92.71	48	21	0	0	112	51	138	134	50	0	0										
92.80	60	0	0	0	22	15	41	88	51	149	0										
92.91	33	0	0	17	81	52	61	130	13	170	17										
93.01	56	0	0	46	100	63	24	62	23	122	0										
93.11	50	0	0	0	73	68	67	69	36	77	0										
93.21	34	0	0	22	73	62	10	0	36	317	0										
93.31	51	0	8	0	100	62	0	0	29	0	0										
93.41	44	26	0	16	63	83	0	111	43	49	12										
93.50	49	0	0	28	111	59	55	47	27	178	7										
93.61	0	0	0	22	121	57	145	103	32	0	0										
93.71	46	8	13	0	86	53	14	113	18	27	23										
93.81	64	0	0	56	73	62	53	127	49	171	9										
93.90	37	0	0	5	95	38	5	14	44	137	0										
94.01	50	10	0	0	98	26	45	63	32	221	13										
94.11	11	17	0	27	114	74	150	20	42	94	0										
94.20	17	0	0	34	103	106	6	57	34	226	0										
94.31	27	0	13	0	102	109	34	34	27	321	0										
94.40	53	0	0	20	91	40	40	26	26	200	0										
94.50	37	0	0	28	109	57	85	56	27	56	0										
94.61	67	0	0	19	108	56	0	96	30	0	0										
94.70	46	0	0	5	94	58	69	137	37	18	12										
94.81	45	0	0	0	69	40	88	70	8	0	19										
94.91	31	0	0	20	77	39	5	120	22	174	0										
95.00	40	0	0	11	43	49	0	99	29	196	0										
95.11	45	0	0	0	106	92	65	0	24	309	0										
95.20	20	0	0	7	107	93	0	0	33	414	5										
95.31	26	0	0	46	66	85	36	0	48	342	7										
95.41	44	0	0	5	78	32	59	118	41	392	0										
95.50	26	0	0	0	111	90	0	52	30	437	8										
95.61	42	0	0	21	82	95	72	18	64	379	0										
95.71	43	9	0	28	80	45	55	36	28	201	0										
95.81	40	0	0	0	111	71	25	17	27	104	13										
95.91	64	8	0	0	79	73	70	93	25	25	0										
96.00	33	19	0	0	72	7	126	111	5	0	31										
96.10	36	0	0	0	68	39	46	159	39	37	17										
96.21	36	0	0	0	92	84	68	95	18	172	12										

96.30	35	0	0	14	110	49	58	42	46	252	0										
96.41	42	0	0	13	123	52	0	76	53	315	0										
96.51	38	0	0	0	92	54	0	48	39	281	21										
96.61	20	0	0	33	75	64	0	0	33	340	0										
96.71	35	0	0	34	93	106	56	117	40	253	17										
96.81	53	0	0	8	81	54	0	28	38	408	12										
96.91	42	0	0	0	93	98	25	48	43	290	0										
97.01	62	23	0	0	56	70	57	170	26	225	0										
97.10	40	0	0	48	54	8	64	142	43	91	30										
97.21	47	0	0	32	94	12	0	166	25	0	20										
97.31	32	0	0	27	72	49	52	226	0	123	13										
97.40	44	4	13	0	93	42	17	131	37	179	0										
97.51	43	0	0	21	106	49	44	67	41	351	5										
97.60	22	0	0	0	90	89	39	18	22	217	0										
97.71	45	0	0	0	72	50	0	61	45	474	0										
97.81	41	0	0	7	133	34	120	112	19	421	5										
97.90	40	0	0	28	85	58	32	8	23	241	0										
98.00	27	0	0	23	119	123	140	176	23	265	25										
98.11	59	0	0	12	131	60	127	43	33	19	0										
98.21	45	5	0	26	112	22	32	84	15	173	0										
98.31	42	0	0	0	50	71	48	190	22	138	0										
98.40	58	4	0	0	69	38	127	125	35	0	13										
98.50	52	0	0	0	85	46	0	147	34	72	0										
98.61	60	0	11	14	96	23	92	116	58	246	9										
98.70	44	0	0	59	83	104	64	13	38	0	28										
98.81	35	0	0	73	96	63	136	14	34	346	8										
98.91	34	10	0	30	66	108	0	13	40	234	0										
99.00	44	0	0	11	102	106	0	93	35	294	0										
99.10	36	0	0	20	101	94	0	50	38	389	0										
99.20	41	0	0	26	88	95	77	82	5	218	0										
99.31	51	0	0	32	141	93	35	61	36	164	0										
99.40	74	0	0	0	62	31	0	61	22	178	21										
99.50	50	0	0	0	78	71	112	162	15	0	15										
99.60	39	0	0	0	87	60	0	207	9	0	0										
99.71	54	0	0	0	94	20	11	63	46	225	12										
99.80	46	0	0	0	82	50	41	0	40	207	0										
99.91	39	0	0	34	96	65	0	0	29	202	20										
100.00	48	0	0	45	112	143	23	28	48	310	10										
100.11	31	0	0	0	118	84	16	15	34	380	15										
100.21	48	0	0	19	197	50	47	47	29	127	0										
100.30	43	0	0	16	108	45	82	67	29	278	28										
100.41	31	0	0	28	98	48	60	198	0	170	20										
100.50	29	0	0	34	100	66	115	98	26	114	16										
100.61	41	0	0	4	77	96	82	115	37	108	0										
100.71	34	0	0	13	91	101	8	70	32	285	29										
100.80	19	0	5	0	122	79	83	40	58	311	0										
100.91	30	0	0	20	91	112	0	0	69	539	20										
101.01	29	0	0	0	89	114	0	50	81	410	0										
101.11	44	0	0	29	73	130	41	52	55	477	15										
101.21	57	0	0	40	97	76	0	10	116	285	0										
101.31	40	0	6	0	110	95	8	54	104	253	40										
101.41	24	0	0	8	127	30	40	172	54	256	13										
101.51	43	0	0	0	38	124	0	119	66	276	0										
101.60	43	8	0	17	96	104	63	35	82	279	17										
101.71	20	0	0	45	82	78	100	0	30	270	13										
101.81	19	0	0	40	118	154	24	9	18	524	0										
101.90	0	0	0	6	113	221	0	90	59	468	23										
102.00	25	0	0	0	120	82	14	116	54	349	0										
102.11	0	10	0	11	104	79	0	37	70	286	0										
102.21	26	0	0	0	82	170	0	20	55	481	45										
102.31	30	0	0	11	98	117	0	0	52	438	16										
102.40	31	0	0	0	89	115	0	48	61	544	19										
102.50	32	0	0	5	97	76	42	0	50	280	7										

102.61	49	0	10	24	56	56	0	81	40	104	32										
102.70	26	0	0	10	100	52	23	90	46	223	9										
102.81	36	0	0	42	58	52	34	0	44	316	0										
102.91	41	0	0	5	144	80	113	131	33	191	0										
103.01	40	0	0	6	90	52	0	33	28	375	6										
103.11	31	0	0	14	83	19	0	91	45	293	33										
103.20	46	0	0	20	97	54	75	60	31	403	9										
103.30	51	0	0	0	33	38	0	145	28	209	16										
103.41	54	0	0	18	123	56	141	106	37	257	16										
103.50	14	0	0	19	53	21	73	9	19	286	9										
103.61	22	6	0	0	71	35	86	82	30	143	5										
103.71	12	5	0	33	69	29	0	44	22	207	0										
103.80	36	0	0	0	86	79	0	162	13	390	0										
103.91	61	0	0	46	101	79	0	26	19	393	9										
104.00	49	0	0	28	77	74	0	120	0	188	19										
104.10	50	0	0	14	106	46	0	140	47	210	0										
104.21	29	0	0	33	50	18	22	20	42	254	0										
104.31	41	0	0	0	72	0	0	0	8	216	29										
104.41	45	0	0	0	65	46	126	0	36	333	0										
104.51	17	0	0	0	73	72	36	145	38	407	23										
104.60	77	0	0	10	65	46	0	45	32	478	0										
104.71	18	0	0	19	102	56	0	88	26	229	8										
104.81	70	0	0	0	72	31	12	86	24	184	0										
104.91	35	0	0	27	84	70	0	26	25	288	4										
105.01	18	0	0	0	77	60	0	0	25	530	0										
105.11	31	0	0	0	74	79	0	9	8	344	13										
105.21	20	0	0	0	95	84	52	112	33	411	22										
105.31	37	0	0	17	101	43	29	51	22	419	0										
105.40	15	0	0	14	125	83	0	135	8	311	9										
105.51	33	0	0	4	64	30	98	111	42	238	5										
105.60	57	0	0	0	45	28	0	67	15	247	10										
105.71	51	0	0	10	78	36	51	84	38	342	8										
105.80	45	0	0	6	104	82	0	43	22	334	16										
105.90	31	0	0	29	87	76	149	107	35	311	0										
106.01	29	0	0	0	126	16	58	151	52	200	0										
106.10	27	0	0	32	72	26	102	96	42	296	12										
106.20	38	0	0	0	75	19	125	63	30	375	0										
106.31	14	0	0	19	61	81	71	73	17	312	16										
106.41	0	13	0	0	93	51	18	10	21	252	7										
106.51	39	0	0	16	66	65	50	15	41	194	0										
106.60	25	0	0	35	96	88	19	0	18	339	0										
106.71	38	0	0	22	77	32	75	135	22	214	24										
106.81	28	0	0	0	141	38	79	108	10	151	23										
106.91	48	9	0	0	56	16	15	84	0	179	8										
107.01	45	0	0	0	56	0	0	80	43	159	0										
107.10	47	0	0	17	94	60	57	61	39	92	9										
107.21	63	11	0	26	57	18	8	0	14	402	0										
107.31	27	0	0	6	72	34	115	0	33	353	0										
107.41	26	0	0	9	80	60	29	23	14	384	6										
107.51	36	0	0	11	76	65	0	0	10	495	0										
107.60	53	0	0	0	62	98	68	56	6	179	0										
107.70	31	0	0	49	72	59	23	40	36	354	13										
107.81	15	0	0	10	84	86	119	0	32	352	0										
107.90	63	0	0	35	74	46	130	155	20	228	0										
108.01	51	0	0	12	87	78	59	123	34	149	26										
108.11	49	0	0	17	84	29	97	67	15	279	0										
108.21	29	0	0	20	66	0	34	90	28	235	6										
108.30	38	0	0	4	89	42	109	61	31	302	0										
108.41	36	0	0	0	75	32	0	96	40	252	0										
108.50	41	0	0	0	94	50	12	68	18	333	0										
108.60	12	0	0	4	86	62	40	0	42	138	0										
108.71	42	0	0	27	49	44	77	75	54	232	0										
108.80	50	0	0	26	99	60	73	211	44	188	0										

108.91	34	0	0	0	77	48	0	145	24	183	8										
109.00	36	0	0	24	109	32	84	74	45	226	18										
109.11	65	0	0	0	46	14	183	130	6	104	0										
109.21	11	0	0	14	62	67	18	148	24	293	0										
109.30	53	0	0	10	48	34	106	62	22	348	0										
109.40	55	0	0	25	78	35	5	6	23	147	0										
109.51	44	16	0	24	91	36	0	0	20	261	15										
109.61	38	4	0	6	59	82	69	129	22	313	0										
109.71	25	0	0	33	100	23	171	49	28	367	0										
109.80	55	0	0	0	58	34	0	54	47	120	0										
109.91	65	0	0	30	77	58	49	83	37	59	24										
110.01	28	7	0	47	71	24	49	132	46	199	0										
110.10	37	0	0	0	78	60	14	61	39	403	0										
110.21	49	0	0	5	71	71	31	0	23	306	11										
110.30	32	0	0	22	81	43	91	77	33	451	0										
110.40	11	0	0	0	112	64	66	83	29	401	5										
110.51	29	0	0	23	88	46	22	6	43	258	13										
110.61	37	0	0	18	61	108	108	21	20	398	20										
110.71	43	0	0	0	82	112	21	0	24	315	20										
110.81	40	18	0	0	39	28	16	0	14	361	23										
110.90	41	0	0	41	72	74	91	88	19	78	0										
111.01	42	5	0	0	93	6	62	24	36	174	0										
111.10	49	9	0	0	85	27	0	26	40	130	15										
111.20	38	0	0	10	90	84	61	5	17	257	11										
111.30	35	0	0	0	107	88	35	22	20	211	7										
111.40	45	0	0	52	79	81	10	113	50	318	7										
111.51	26	0	0	26	75	113	19	0	51	376	16										
111.60	55	0	0	10	105	71	65	0	40	375	6										
111.71	23	0	0	0	111	107	0	0	32	341	0										
111.81	6	0	0	50	75	64	78	0	53	489	0										
111.90	11	0	0	4	163	89	79	122	15	515	18										
112.01	10	0	0	0	89	45	131	158	25	292	0										
112.11	33	12	0	41	91	70	46	0	24	185	15										
112.21	32	0	0	0	65	59	271	99	27	122	0										
112.31	50	0	0	0	36	59	119	100	34	249	0										
112.41	30	0	0	0	87	77	118	164	20	131	0										
112.51	35	0	0	0	81	33	89	0	30	354	0										
112.61	48	0	0	9	83	65	55	41	45	455	31										
112.71	5	0	0	10	53	39	0	0	20	239	0										
112.81	25	0	0	24	94	77	32	0	46	402	0										
112.90	26	0	0	22	112	44	7	35	30	221	0										
113.01	53	0	0	5	85	105	75	29	19	217	4										
113.11	0	0	0	32	98	50	141	28	19	200	0										
113.20	67	0	0	18	72	13	15	43	16	219	5										
113.31	45	0	0	0	80	40	0	138	19	269	6										
113.41	56	0	10	21	79	53	123	113	20	328	0										
113.50	18	0	0	0	41	48	116	35	47	196	0										
113.60	34	0	0	20	115	58	39	158	72	215	8										
113.71	49	0	0	7	110	76	0	110	34	309	13										
113.81	46	0	0	8	92	108	91	131	34	348	0										
113.91	29	0	0	31	77	73	0	4	34	340	0										
114.01	36	0	0	22	124	104	111	16	32	409	12										
114.10	47	0	0	33	77	58	0	57	29	303	17										
114.20	48	0	0	6	94	56	35	0	13	320	7										
114.31	48	8	0	4	102	73	0	99	37	366	0										
114.40	19	0	0	15	44	65	52	96	39	320	22										
114.51	36	0	0	0	71	8	48	60	40	61	0										
114.60	29	0	0	9	54	59	47	83	23	291	0										
114.70	39	0	0	0	62	12	57	45	27	175	5										
114.81	39	0	0	14	105	58	75	46	40	153	13										
114.91	36	0	0	0	71	47	85	34	41	241	8										
115.00	40	0	0	0	88	79	88	12	8	312	8										
115.11	21	0	0	17	116	44	86	138	40	260	0										

115.21	13	0	0	0	100	84	66	72	34	207	25										
115.31	14	0	0	0	73	75	10	0	0	265	16										
115.40	43	0	0	15	83	95	55	56	0	287	0										
115.50	42	0	0	12	106	41	9	0	26	509	5										
115.61	38	0	0	18	99	95	14	75	21	388	15										
115.71	41	11	0	0	61	69	0	0	40	517	18										
115.80	39	0	0	21	64	26	34	27	36	312	0										
115.91	18	0	0	0	64	67	23	0	13	260	4										
116.01	50	0	0	0	106	33	123	106	26	250	0										
116.11	55	0	0	58	97	102	88	133	58	39	0										
116.21	32	17	0	18	71	100	66	22	42	160	0										
116.31	47	0	0	21	79	77	143	54	18	318	0										
116.41	30	0	0	10	82	49	10	94	42	204	10										
116.50	21	0	0	0	67	14	17	91	44	282	16										
116.61	60	0	0	11	53	51	139	0	14	76	18										
116.71	45	0	0	18	97	81	17	59	33	408	10										
116.80	27	0	0	18	79	94	97	0	40	309	18										
116.91	30	0	0	40	74	71	150	113	47	314	0										
117.00	20	0	0	20	63	57	71	0	0	166	0										
117.11	44	0	0	28	99	84	66	60	40	44	9										
117.21	61	0	0	27	71	46	89	0	42	207	0										
117.31	16	5	0	5	55	31	69	110	31	280	0										
117.41	39	10	0	27	73	50	87	71	26	151	6										
117.51	15	0	0	13	59	70	33	49	19	213	0										
117.61	31	0	0	0	51	74	0	181	41	238	0										
117.70	53	0	0	0	102	69	78	116	24	237	15										
117.81	50	12	0	8	111	88	54	0	17	305	0										
117.91	26	0	0	50	68	101	0	57	30	367	26										
118.01	29	0	0	25	83	103	153	54	38	519	0										
118.10	36	0	0	0	103	105	36	65	40	77	0										
118.20	24	0	0	36	89	42	63	151	0	390	0										
118.31	33	0	0	32	106	73	28	68	33	264	0										
118.40	26	0	0	14	58	56	158	0	23	201	0										
118.51	34	0	0	9	80	96	59	15	46	376	9										
118.61	40	0	0	0	86	79	22	73	55	284	11										
118.70	44	0	0	0	35	77	121	0	34	201	8										
118.81	26	0	0	41	74	101	39	78	27	227	0										
118.90	51	24	0	0	76	31	156	144	45	180	0										
119.01	56	0	0	0	95	60	185	156	8	179	23										
119.11	20	0	0	30	49	67	27	157	31	120	0										
119.20	46	0	0	0	78	68	127	122	26	107	0										
119.31	43	8	0	0	41	47	92	52	49	200	0										
119.40	52	0	0	26	68	68	104	141	45	27	11										
119.51	55	0	0	23	58	19	60	111	23	282	0										
119.61	36	0	0	18	108	68	65	0	33	146	21										
119.71	68	0	0	14	45	98	0	58	40	267	25										
119.81	65	0	0	13	67	105	19	141	60	273	22										
119.90	40	0	0	12	98	77	113	100	22	241	0										
120.00	41	0	0	17	51	70	48	136	25	351	11										
120.11	36	0	0	26	64	40	0	22	27	186	8										
120.21	0	0	0	0	98	210	23	0	41	284	0										
120.31	44	0	0	24	82	338	47	0	60	206	0										
120.41	57	0	0	55	107	122	105	78	48	486	0										
120.51	32	0	0	6	74	89	95	0	37	296	15										
120.61	33	0	0	16	86	59	133	40	47	209	0										
120.71	61	0	0	13	63	71	106	104	49	269	7										
120.81	37	9	0	50	83	58	25	56	58	225	25										
120.91	22	0	0	12	27	36	20	61	33	248	0										
121.01	38	0	0	0	42	0	0	236	28	275	0										
121.10	56	0	0	20	81	58	146	108	28	128	0										
121.20	50	0	0	5	71	75	133	53	20	0	5										
121.31	64	0	0	0	65	36	14	86	42	163	0										
121.41	49	0	0	36	43	66	41	0	8	205	0										

121.51	13	8	0	7	94	54	54	0	31	139	22										
121.61	31	0	0	0	69	79	162	70	51	260	11										
121.71	23	0	0	0	31	61	43	45	27	191	0										
121.81	19	0	0	41	73	43	63	0	26	353	4										
121.90	48	9	0	11	71	98	52	64	31	504	0										
122.01	40	7	0	13	126	72	61	24	74	338	0										
122.10	45	0	0	6	23	71	51	39	34	345	0										
122.20	43	0	0	5	105	118	149	0	33	440	12										
122.30	27	0	0	0	48	39	97	86	25	231	7										
122.40	22	0	0	0	62	47	114	76	37	237	25										
122.51	46	9	13	20	37	27	19	173	19	83	9										
122.61	72	8	0	0	13	40	174	225	43	153	0										
122.71	52	0	4	0	37	61	69	132	25	141	0										
122.81	53	0	0	16	73	31	124	103	37	116	11										
122.90	50	13	0	0	30	75	93	19	50	0	7										
123.01	44	0	0	40	79	61	68	42	26	357	0										
123.11	15	0	0	0	76	78	73	91	40	193	17										
123.21	35	0	0	39	54	97	12	17	37	305	10										
123.31	26	0	7	37	73	65	0	0	23	274	9										
123.40	27	0	0	6	52	71	0	21	42	372	37										
123.51	33	0	0	0	81	51	83	130	61	241	0										
123.60	38	0	0	21	103	99	57	0	54	297	22										
123.70	33	0	0	0	59	92	88	0	33	358	0										
123.81	59	0	0	45	102	92	94	17	48	339	0										
123.91	34	0	0	14	72	33	62	72	61	453	0										
124.00	33	0	0	10	69	104	0	0	16	214	0										
124.11	34	0	0	0	41	72	11	0	25	303	9										
124.20	56	0	0	15	46	39	0	29	45	258	8										
124.31	37	0	0	30	61	53	36	0	33	164	26										
124.41	48	0	0	13	86	49	25	81	27	269	0										
124.51	73	0	0	10	117	22	152	0	8	408	19										
124.61	48	14	0	6	104	27	95	95	22	284	0										
124.71	30	0	0	10	101	59	134	72	29	214	33										
124.80	32	0	0	21	62	61	185	143	28	94	13										
124.91	46	0	0	0	82	19	50	128	20	105	0										
125.00	50	0	0	17	66	0	78	95	34	0	10										
125.11	30	10	0	0	122	80	73	146	37	225	0										
125.21	42	0	0	0	114	34	52	40	38	273	23										
125.31	51	0	0	19	48	50	13	0	51	228	0										
125.41	23	0	0	0	49	50	70	44	35	340	0										
125.51	18	0	0	29	74	65	53	54	35	198	23										
125.61	41	0	0	23	78	72	42	61	43	361	18										
125.71	28	0	0	28	75	97	0	33	39	424	0										
125.81	23	0	0	6	57	98	60	108	29	368	25										
125.91	38	0	0	9	82	90	84	74	53	267	12										
126.01	26	0	0	16	109	49	109	0	40	272	16										
126.11	34	0	0	41	68	36	61	69	34	315	0										
126.20	46	0	0	30	64	79	86	0	39	306	11										
126.30	20	0	0	11	60	93	85	48	13	332	0										
126.40	40	0	0	0	123	20	42	91	37	324	17										
126.51	31	0	0	0	95	55	154	115	21	164	24										
126.60	45	0	0	28	57	42	132	104	40	229	0										
126.71	37	0	0	12	118	63	161	60	33	89	13										
126.80	35	0	0	25	50	28	28	94	24	256	10										
126.91	21	0	0	6	51	13	62	0	43	335	0										
127.01	61	0	0	7	15	16	11	106	39	335	10										
127.10	38	0	0	41	73	60	0	32	29	236	10										
127.21	22	0	0	0	106	34	119	89	5	395	0										
127.31	45	0	0	9	121	115	0	112	49	275	22										
127.40	31	0	0	9	70	105	0	21	46	204	0										
127.51	33	0	0	29	94	74	57	81	12	213	0										
127.60	39	0	0	0	90	14	0	0	35	359	11										
127.70	40	0	0	32	87	67	44	39	34	407	0										

127.80	42	0	0	51	98	74	57	60	43	514	0										
127.91	51	0	0	16	61	45	11	88	13	128	10										
128.01	56	0	0	0	89	24	15	47	38	130	0										
128.11	29	0	0	0	50	51	9	0	44	123	0										
128.21	41	0	0	32	63	23	140	47	41	164	14										
128.31	23	0	0	9	126	78	94	115	18	357	0										
128.40	30	0	0	0	78	55	47	32	24	67	0										
128.50	11	14	0	14	83	41	131	40	42	207	7										
128.61	33	0	0	37	66	45	82	0	26	74	0										
128.71	37	12	0	5	120	49	147	29	31	302	10										
128.80	38	14	0	0	96	79	69	19	36	208	17										
128.91	38	0	5	0	86	76	88	65	39	179	30										
129.01	30	0	0	0	102	52	88	110	40	369	6										
129.11	68	17	0	22	125	41	91	44	53	311	0										
129.21	34	0	0	13	67	78	0	143	42	187	0										
129.30	28	0	0	26	67	67	0	73	34	344	18										
129.40	43	0	0	19	67	56	68	92	50	442	7										
129.51	18	0	0	16	56	77	0	0	39	30	28										
129.60	22	0	0	43	59	93	110	114	12	277	29										
129.71	26	0	0	0	75	90	22	59	41	298	16										
129.81	37	0	0	0	112	46	76	0	31	413	0										
129.90	47	0	0	47	94	29	26	0	27	342	0										
130.01	43	0	0	30	95	33	87	45	38	470	0										
130.10	71	6	0	12	65	37	93	0	19	342	0										
130.20	57	0	0	0	89	92	37	174	69	311	0										
130.31	38	0	0	6	83	71	68	69	38	174	22										
130.40	11	0	0	9	48	62	41	0	39	273	0										
130.51	39	0	0	0	118	91	91	38	20	230	7										
130.61	18	0	0	13	70	54	42	0	26	343	7										
130.71	47	0	0	12	85	69	48	23	34	209	0										
130.81	20	0	0	29	98	17	87	74	30	229	9										
130.90	31	0	0	0	72	55	111	0	21	79	22										
131.01	67	0	0	19	46	44	108	86	44	113	13										
131.11	41	5	0	0	57	15	38	79	64	0	27										
131.21	23	0	0	0	64	60	132	130	31	177	12										
131.31	25	0	0	20	91	12	161	167	43	211	0										
131.40	43	0	0	12	60	75	124	98	41	183	10										
131.51	51	36	0	7	98	0	123	149	34	138	0										
131.61	27	0	0	41	57	56	91	119	40	118	27										
131.70	45	20	7	17	82	0	0	71	40	154	8										
131.81	39	0	0	29	103	42	178	105	58	29	22										
131.91	34	0	0	0	94	49	28	131	54	202	0										
132.01	25	0	0	0	44	83	45	69	27	223	25										
132.11	49	0	0	46	110	79	39	97	44	336	20										
132.21	45	0	0	32	86	92	0	198	9	377	0										
132.31	57	6	0	0	84	69	109	0	35	373	0										
132.40	9	0	0	0	92	0	10	97	45	464	13										
132.51	50	0	0	0	68	53	77	77	36	250	17										
132.61	41	0	0	24	83	60	65	124	49	235	17										
132.71	42	0	0	67	110	113	84	39	55	423	19										
132.81	24	0	0	0	98	51	27	0	51	250	12										
132.91	69	0	0	12	66	47	84	55	53	364	0										
133.01	33	0	0	64	91	44	27	76	40	258	16										
133.10	39	10	0	18	73	35	103	0	41	268	8										
133.21	33	0	0	23	50	82	59	71	60	185	10										
133.31	41	0	0	21	110	74	112	81	68	162	15										
133.41	42	0	0	12	97	88	91	0	26	342	0										
133.51	64	0	0	33	66	73	0	26	22	293	13										
133.61	64	0	0	54	75	89	97	10	33	261	20										
133.70	31	9	0	29	73	75	0	124	33	278	14										
133.81	35	0	0	0	58	55	0	0	22	226	0										
133.90	34	0	0	25	67	74	84	105	65	310	9										
134.01	50	0	15	13	76	52	67	27	39	274	0										

134.11	63	0	0	4	45	24	120	118	22	290	13										
134.20	60	0	0	0	53	62	153	54	7	168	16										
134.31	49	0	0	21	37	71	21	132	28	218	5										
134.41	59	0	0	21	70	0	119	123	35	155	10										
134.51	24	19	0	45	50	69	15	8	27	209	4										
134.61	37	0	0	0	128	38	90	64	0	317	0										
134.70	18	0	0	26	73	62	90	41	25	327	16										
134.80	51	0	0	10	84	71	36	50	34	193	0										
134.91	56	0	0	43	91	36	47	20	43	311	0										
135.01	21	0	0	0	91	70	71	7	77	231	8										
135.11	35	0	0	42	39	88	27	0	76	271	0										
135.20	48	0	0	0	41	93	52	39	47	289	17										
135.30	9	0	0	47	68	76	6	14	34	251	8										
135.41	16	0	0	26	77	66	46	35	46	286	21										
135.50	34	7	0	51	67	48	124	106	61	328	0										
135.61	46	0	0	0	88	66	0	131	43	230	0										
135.70	31	0	0	20	92	96	49	29	27	448	0										
135.80	53	0	0	4	85	73	82	82	60	329	0										
135.91	0	0	0	23	74	105	93	42	49	420	4										
136.00	38	0	0	0	67	100	101	0	28	262	11										
136.11	7	0	0	8	123	113	4	50	14	258	21										
136.21	44	0	0	26	52	64	48	0	60	457	15										
136.31	44	0	0	0	67	48	30	0	52	506	10										
136.41	4	0	0	32	96	88	10	10	36	315	21										
136.50	0	0	0	0	81	46	89	4	46	367	5										
136.61	34	0	0	25	58	33	48	4	57	290	4										
136.71	23	0	0	0	33	79	68	0	64	267	0										
136.81	22	0	0	0	56	122	23	66	49	162	32										
136.91	25	0	0	24	105	67	88	78	23	365	29										
137.01	55	0	0	17	75	55	105	50	31	341	16										
137.10	30	0	0	22	64	13	138	34	38	292	11										
137.21	17	0	0	11	81	63	96	112	18	361	6										
137.30	55	0	0	9	47	34	127	73	25	316	8										
137.41	31	0	0	14	71	103	70	41	37	158	4										
137.51	39	0	0	11	28	4	61	0	13	232	0										
137.61	51	0	0	31	81	46	102	212	29	238	11										
137.71	51	0	0	0	59	5	46	108	24	156	0										
137.81	55	0	0	35	40	52	49	67	39	55	4										
137.90	58	0	0	7	81	68	162	47	49	269	4										
138.01	34	0	0	14	54	0	89	37	60	115	4										
138.11	40	0	0	5	65	29	26	116	32	192	8										
138.20	30	0	0	25	70	81	131	60	25	161	0										
138.31	55	7	5	8	57	6	59	93	37	158	0										
138.40	50	0	0	32	57	63	0	69	69	296	13										
138.50	48	0	0	23	77	58	131	84	56	173	15										
138.60	40	20	0	18	84	42	22	6	48	66	15										
138.71	63	0	0	18	84	33	135	15	38	235	0										
138.81	48	0	0	20	69	51	57	0	29	292	34										
138.90	23	0	0	31	53	35	98	0	40	336	0										
139.01	54	0	0	26	104	82	136	28	35	285	0										
139.11	38	0	0	0	79	89	108	0	23	369	0										
139.21	29	6	0	15	50	50	43	5	59	209	24										
139.30	46	0	0	11	32	40	32	0	35	345	0										
139.41	26	9	0	0	96	84	141	0	26	453	11										
139.51	33	12	0	0	49	83	71	0	47	466	0										
139.60	51	0	0	21	65	111	139	67	62	362	0										
139.70	47	0	0	7	80	60	0	0	54	262	22										
139.81	4	0	0	0	56	90	0	63	38	249	0										
139.90	29	0	0	10	132	75	102	0	42	368	5										
140.01	48	0	0	12	81	52	212	0	37	174	24										
140.10	28	0	0	0	95	64	47	11	39	176	10										
140.21	6	0	0	0	64	101	17	22	47	415	0										
140.31	17	0	0	18	71	36	158	0	29	438	12										

140.41	34	0	0	0	87	129	76	96	56	404	8										
140.51	53	0	0	0	99	104	37	29	42	421	9										
140.60	33	0	0	8	86	76	15	42	33	339	0										
140.71	27	0	0	30	102	101	83	118	42	479	0										
140.80	32	0	0	0	20	85	127	97	63	319	14										
140.90	25	0	0	13	35	68	74	71	60	355	14										
141.01	29	0	0	14	68	26	108	0	48	302	9										
141.11	24	0	0	21	88	99	121	53	54	127	31										
141.20	46	0	0	26	52	99	127	0	70	345	24										
141.30	46	0	0	0	74	85	100	134	47	239	21										
141.41	54	0	0	0	66	101	54	0	67	288	7										
141.50	44	0	0	16	62	67	7	157	46	193	20										
141.60	21	0	0	33	19	52	114	35	39	361	8										
141.70	26	0	0	0	61	54	103	79	48	197	34										
141.81	20	30	0	0	73	39	53	29	65	225	6										
141.90	19	0	0	0	110	40	134	146	59	45	10										
142.01	42	9	0	0	109	40	108	67	69	124	30										
142.11	31	0	0	11	64	87	91	144	58	221	0										
142.21	57	0	11	21	91	87	211	51	83	10	0										
142.31	44	0	0	0	26	60	80	98	51	56	0										
142.41	58	0	0	0	54	65	131	23	43	323	0										
142.51	33	0	0	13	47	41	129	0	46	70	0										
142.61	68	0	0	16	75	98	57	81	51	203	0										
142.71	33	8	7	27	78	72	0	66	54	96	27										
142.81	18	4	0	0	84	20	35	96	42	376	12										
142.91	26	0	0	50	101	37	103	76	45	426	15										
143.00	16	0	0	0	64	18	131	0	47	256	9										
143.10	47	0	0	0	87	94	152	65	59	206	0										
143.21	54	0	0	0	69	35	39	112	46	308	27										
143.31	40	0	0	37	47	81	63	0	23	198	21										
143.40	62	0	0	19	85	96	168	0	34	200	8										
143.51	47	0	0	36	33	44	37	27	43	349	0										
143.60	26	0	0	0	64	95	165	17	29	291	34										
143.70	37	0	0	0	76	89	214	126	28	269	11										
143.81	25	0	0	0	67	62	154	0	33	314	0										
143.90	60	0	0	47	62	56	52	22	55	243	14										
144.00	45	10	0	0	97	78	49	0	41	298	0										
144.11	50	6	0	19	65	72	63	0	43	173	7										
144.20	12	0	0	35	72	47	98	89	59	336	0										
144.30	48	0	0	19	130	64	66	40	49	274	31										
144.41	33	0	0	0	78	68	54	0	53	287	0										
144.50	35	0	0	0	93	93	36	0	23	403	10										
144.61	28	0	0	22	73	65	41	55	59	336	0										
144.71	61	0	0	50	40	42	100	90	51	437	11										
144.81	37	0	0	7	49	72	68	37	30	226	0										
144.91	6	0	0	0	78	92	0	27	39	222	0										
145.00	35	0	0	30	80	87	74	145	61	343	10										
145.11	36	0	0	31	79	59	44	181	53	267	19										
145.21	20	0	0	0	93	117	165	19	44	255	0										
145.31	74	6	0	0	78	122	40	115	29	264	0										
145.40	48	0	0	6	73	75	111	40	70	273	7										
145.50	29	0	0	14	78	0	48	0	38	444	12										
145.61	42	0	0	34	103	69	179	48	64	372	15										
145.71	62	0	0	22	98	55	5	94	61	222	6										
145.80	42	0	0	0	110	106	190	0	66	340	6										
145.91	40	0	0	35	64	60	125	39	49	211	0										
146.01	42	0	0	42	57	78	133	78	44	242	0										
146.11	49	0	0	17	65	83	86	27	51	334	11										
146.21	7	0	0	13	55	46	160	0	46	313	0										
146.31	34	0	0	24	87	62	36	0	45	380	40										
146.41	66	0	0	17	110	69	143	42	43	233	5										
146.51	44	0	0	0	69	55	229	146	47	195	0										
146.60	38	17	0	31	92	50	196	80	12	161	0										

146.71	42	10	0	22	94	47	102	0	53	207	22										
146.81	39	0	6	0	76	67	60	40	27	59	12										
146.91	51	0	0	0	31	94	152	11	38	113	31										
147.01	35	0	0	14	83	82	100	67	55	201	15										
147.10	30	0	0	26	61	88	14	0	59	393	0										
147.21	42	0	0	14	42	29	123	0	40	153	23										
147.31	54	0	0	0	103	62	162	97	51	413	0										
147.41	38	0	0	0	65	94	118	0	45	320	34										
147.51	22	0	0	4	19	32	105	0	27	282	34										
147.61	52	0	7	12	64	75	73	30	35	202	14										
147.71	30	0	0	23	53	32	90	48	45	138	18										
147.80	20	0	0	43	68	61	140	17	15	185	7										
147.91	23	0	0	31	90	70	81	32	29	238	20										
148.00	35	0	0	33	78	61	65	25	38	121	0										
148.10	21	19	0	0	65	77	128	31	51	341	13										
148.20	26	0	0	0	36	65	194	73	29	320	0										
148.30	52	0	13	19	99	63	139	87	26	340	0										
148.41	30	0	0	0	46	113	56	66	38	364	23										
148.50	54	0	0	18	82	38	83	32	40	277	10										
148.60	18	6	0	21	93	141	42	7	61	234	14										
148.71	37	0	0	26	107	88	19	27	54	464	7										
148.81	50	0	0	29	58	103	0	56	47	131	7										
148.91	43	0	0	18	106	34	114	74	42	292	21										
149.01	43	0	0	0	121	39	33	73	51	187	0										
149.11	62	0	0	14	79	83	110	0	43	200	17										
149.21	19	0	0	6	89	101	81	87	65	390	0										
149.30	57	0	0	36	77	53	55	18	19	202	10										
149.41	44	16	0	0	81	127	50	5	46	417	17										
149.51	66	0	0	18	77	127	159	46	68	287	0										
149.61	21	0	0	18	126	102	94	27	57	257	7										
149.71	55	0	0	20	85	23	132	0	50	240	17										
149.81	28	0	0	5	59	104	105	89	36	243	12										
149.91	34	0	0	0	89	108	220	0	50	296	28										
150.00	33	0	0	0	45	59	145	73	71	294	27										
150.11	8	0	0	0	47	74	56	79	49	243	0										
150.21	34	0	0	32	109	73	63	33	32	186	18										
150.30	46	0	0	24	62	87	89	57	56	347	6										
150.40	43	0	0	0	78	134	81	64	66	324	0										
150.51	40	0	0	9	62	235	173	0	65	185	41										
150.61	67	0	0	0	83	116	9	36	58	495	0										
150.71	65	0	0	30	5	73	170	97	70	378	22										
150.80	55	0	0	0	63	55	78	29	45	269	23										
150.91	44	15	0	39	113	75	131	0	40	269	12										
151.01	54	7	0	0	92	26	144	34	33	248	29										
151.11	20	0	0	0	119	90	196	155	58	287	19										
151.21	37	0	0	0	77	66	175	87	63	220	21										
151.30	43	0	0	0	64	95	212	31	55	279	6										
151.41	39	0	0	6	44	92	107	57	37	416	33										
151.51	46	0	0	0	35	106	124	76	55	254	0										
151.60	18	0	0	18	97	98	105	57	74	303	20										
151.71	18	0	6	0	35	59	145	29	43	213	0										
151.80	37	0	7	24	50	42	250	38	57	241	10										
151.91	43	0	0	19	64	82	79	0	57	202	24										
152.01	38	0	0	0	102	102	123	19	38	408	22										
152.11	43	0	0	12	73	29	98	58	27	155	11										
152.21	30	5	0	0	104	117	100	9	88	424	12										
152.31	45	0	0	21	97	109	7	40	40	422	32										
152.41	39	9	0	35	74	108	97	52	66	326	14										
152.50	34	0	0	30	32	78	46	34	45	365	0										
152.60	31	6	0	13	77	86	87	7	56	415	30										
152.70	46	0	0	0	104	114	170	84	59	352	0										
152.81	49	0	0	10	110	90	52	38	91	220	4										
152.91	36	0	0	56	45	79	8	65	75	482	18										

153.01	24	0	0	0	77	88	106	59	62	425	12										
153.10	26	0	0	0	87	61	130	74	49	238	0										
153.20	45	7	0	0	82	41	88	64	78	289	37										
153.31	39	9	0	0	60	50	64	34	51	220	4										
153.41	20	8	9	0	66	105	41	74	84	54	6										
153.51	34	0	0	5	104	68	130	110	91	383	12										
153.61	26	0	0	9	52	48	20	0	75	324	0										
153.71	45	5	0	28	76	69	161	27	75	518	8										
153.81	53	0	0	20	90	183	122	0	58	381	0										
153.90	22	0	0	8	87	131	122	59	52	452	13										
154.01	28	0	0	36	103	115	80	32	43	408	0										
154.11	37	0	0	26	101	49	17	0	68	346	25										
154.21	43	0	0	17	112	46	108	31	47	289	0										
154.30	39	0	0	9	82	72	84	47	64	303	34										
154.41	36	8	0	24	51	46	7	45	56	383	0										
154.51	25	0	0	33	121	105	58	47	39	342	32										
154.61	89	6	0	9	80	105	121	0	54	281	20										
154.71	40	0	0	0	24	74	111	0	58	140	0										
154.81	44	0	0	0	99	82	162	22	72	454	13										
154.90	31	0	0	19	111	97	121	94	74	268	17										
155.01	27	0	0	42	54	102	98	11	35	283	0										
155.10	46	5	0	0	56	47	92	20	69	163	11										
155.20	56	0	0	0	83	58	37	56	62	331	0										
155.31	36	0	0	0	67	60	65	99	56	415	7										
155.41	51	14	0	66	41	111	92	14	44	350	8										
155.51	47	0	0	30	69	89	43	12	5	394	14										
155.61	57	0	0	19	58	109	115	92	66	301	0										
155.71	35	0	15	17	84	124	44	0	36	283	24										
155.80	79	11	0	0	85	76	86	82	48	343	0										
155.91	46	8	0	27	149	71	118	51	44	55	16										
156.01	52	0	0	0	93	76	59	38	37	197	6										
156.11	29	0	0	44	64	70	88	41	57	276	7										
156.21	52	12	0	0	69	102	102	40	33	439	0										
156.31	25	0	0	50	42	128	73	54	60	215	0										
156.41	50	0	0	26	81	57	174	99	21	69	6										
156.51	57	0	11	0	131	94	126	46	48	161	0										
156.61	42	0	0	0	76	31	108	114	43	246	9										
156.71	42	0	0	28	79	56	141	30	29	257	0										
156.81	26	0	0	23	55	100	165	43	55	225	23										
156.91	34	6	0	0	89	78	89	145	32	222	11										
157.01	15	8	0	21	83	82	86	74	36	187	0										
157.11	67	0	0	0	62	51	132	16	67	344	26										
157.20	35	0	0	20	85	83	56	0	43	394	29										
157.31	42	0	12	0	98	54	124	80	54	260	0										
157.41	7	0	6	13	91	102	185	0	63	294	18										
157.51	24	0	0	9	104	128	128	52	90	420	0										
157.60	57	0	0	25	73	170	0	0	81	540	55										
157.70	0	0	0	36	59	103	23	0	72	512	17										
157.81	0	0	0	33	90	94	0	0	54	725	11										
157.90	21	0	0	12	82	76	0	0	26	683	13										
158.01	11	0	0	16	89	65	0	0	17	501	0										
158.11	5	0	0	35	55	111	81	0	30	544	6										
158.20	17	0	0	11	27	68	51	0	22	676	0										
158.31	11	0	0	0	80	104	0	0	11	544	0										
158.41	22	0	0	38	70	110	23	0	17	591	8										
158.51	0	0	0	26	86	60	48	0	46	732	0										
158.61	18	0	0	8	67	61	0	0	23	664	0										
158.71	0	0	0	6	65	82	0	0	24	609	0										
158.81	12	0	0	11	51	65	0	0	42	742	5										
158.91	25	0	0	20	109	66	84	0	8	555	8										
159.01	11	0	0	0	81	40	30	30	26	550	0										
159.11	12	0	0	35	87	45	26	0	18	638	12										
159.20	14	0	0	17	50	76	49	42	32	624	0										

159.30	24	0	0	9	83	86	0	0	44	576	9										
159.40	15	0	0	31	80	101	29	0	33	633	0										
159.51	19	0	0	15	68	81	53	0	32	461	13										
159.61	0	0	0	26	47	65	43	0	50	778	10										
159.70	0	0	0	41	83	84	13	35	0	649	9										
159.81	16	0	0	0	57	74	25	0	22	623	0										
159.91	7	0	0	18	60	58	0	0	41	784	0										
160.00	39	0	0	11	62	111	0	0	0	551	9										
160.11	12	0	0	0	91	86	33	0	40	627	0										
160.21	15	0	0	0	51	59	66	0	19	570	7										
160.30	6	0	0	9	55	79	71	0	24	553	0										
160.40	50	0	0	0	40	49	0	0	9	663	6										
160.51	34	0	0	29	73	73	0	0	61	705	0										
160.60	9	0	0	15	59	75	74	64	21	687	5										
160.70	16	0	0	0	52	78	39	0	34	509	8										
160.80	0	0	0	18	76	67	11	22	43	580	0										
160.91	16	0	0	15	70	58	0	0	23	611	13										
161.01	16	0	0	28	72	72	59	0	4	697	0										
161.11	17	0	0	9	76	87	29	0	26	672	0										
161.21	27	0	0	26	88	59	0	0	21	574	26										
161.31	19	0	0	18	76	58	45	0	44	540	0										
161.41	24	0	0	32	98	79	0	0	12	684	0										
161.50	7	0	0	56	49	65	20	18	20	791	0										
161.61	18	0	0	19	48	82	20	0	20	545	0										
161.71	0	0	0	24	94	78	12	0	40	494	0										
161.81	0	0	0	35	95	88	0	0	18	542	0										
161.90	22	0	0	19	61	81	0	0	27	483	0										
162.00	35	0	0	29	80	92	27	17	41	571	15										
162.11	43	0	0	20	22	65	11	0	29	549	0										
162.21	18	0	0	18	79	87	9	0	24	494	0										
162.30	18	0	0	31	96	79	30	0	25	591	0										
162.41	0	0	0	18	70	122	93	24	26	545	18										
162.51	27	0	8	78	80	348	70	13	137	458	9										
162.61	41	0	20	40	77	336	208	95	177	335	0										
162.71	33	0	0	6	47	245	113	165	134	254	44										
162.81	43	0	0	15	81	241	220	217	153	63	28										
162.91	25	0	0	24	39	148	149	184	105	177	7										
163.01	48	0	0	0	70	142	137	59	88	151	17										
163.11	38	0	14	0	89	139	124	62	95	174	0										
163.21	50	0	0	4	87	107	44	31	87	265	20										
163.31	33	0	0	51	49	77	126	19	50	130	18										
163.40	49	0	0	15	90	100	92	59	104	110	12										
163.51	34	0	5	30	103	164	151	42	354	103	18										
163.61	33	27	12	0	105	118	121	166	268	96	0										
163.71	38	7	0	33	98	174	137	180	187	151	8										
163.81	30	6	18	21	86	113	0	0	89	84	0										
163.91	42	0	0	9	55	92	103	110	46	163	18										
